# Open Search-Based Proteomics Reveals Widespread Tryptophan Modifications Associated with Hypoxia in Lung Cancer

**DOI:** 10.1155/2022/2590198

**Published:** 2022-04-30

**Authors:** Jinfeng Chen, Lei Zhang, Zhao Sun, Hongyi Li, Jingyi Li, Xinli Xue, Qingqing Zhu, Bowen Dong, Yuanyuan Wang, Yang Yang, Yongqiang Dong, Guangyu Guo, Hongqiang Jiang, An Zhang, Guoqing Zhang, Zhichao Hou, Xiangnan Li, Jing-Hua Yang

**Affiliations:** ^1^Clinical Systems Biology Key Laboratories of Henan, Translational Medicine Center, The First Affiliated Hospital of Zhengzhou University, Zhengzhou, 450052 Henan, China; ^2^Departments of Surgery, The First Affiliated Hospital of Zhengzhou University, Zhengzhou, Henan 450052, China

## Abstract

The tryptophan residue has a large hydrophobic surface that plays a unique role in the folded protein conformation and functions. Tryptophan modifications are presumably to be readily detected in proteins due to the vulnerability of the indole structure to electrophilic attacks. In this study, we report a systematic identification of sequence variations at tryptophan, termed tryptophan variants, from the proteome of patients with nonsmall cell lung cancer (NSCLC). Using shotgun proteomics and a modified open search algorithm, 25 tryptophan variants on 2481 sites in over 858 proteins were identified. Among these, 6 tryptophan variants are previously identified, 15 are newly annotated, and 4 are still unknown, most of which are involved in the cascade of oxidation in the blood microparticle. Remarkably, Trp313 of glyceraldehyde-3-phosphate dehydrogenase (GAPDH) was up-oxidized whereas Trp16 and Trp38 of hemoglobin (HBB) were down-oxidized in NCSLC tissues. The results were further supported by an independent cohort of 103 lung adenocarcinoma samples, reflecting a negative feedback and potential detoxification mechanism against tumor glycolysis and hypoxia. Overall, the study reports a quick approach to explore tryptophan variants at the proteomic scale. Our findings highlight the predominant role of tryptophan oxidation in regulating the redox balance of cancer cells and its potential role as prognostic biomarker for patients with NSCLC.

## 1. Introduction

Proteins are the main functional biomacromolecules in all organisms, which can be modified at the amino acid (AA) side chains, including by addition, subtraction, or alteration of chemical groups [1]. Modified proteins play key roles in many important cellular functions and regulatory processes [1, 2]. To date, more than 1500 various types of posttranslational modifications (PTMs) have been identified, including acetylation, methylation, glycosylation, phosphorylation, and ubiquitylation [3, 4]. The nucleophilic and high reactivity amino acids, cysteine (Cys) and lysine (Lys), are the two most attractive residues to the electrophilic small molecules [5, 6]. A global map of 3,000 covalently liganded cysteines in primary human T cells have been presented, which can impair T cell activation by distinct mechanisms involving the direct functional perturbation and/or degradation of proteins [5]. A new lysine modification, lactylation, was recently identified and shown to act as an endogenous “lactate clock” in bacterially challenged M1 macrophages to turn on gene expression to promote homeostasis [6].

Recently, tryptophan (Trp) has inspired the interest of researchers because of its unique reactivity and specificity. Tryptophan is the rarest amino acid in eukaryotes, accounting for only approximately 1.13% of all amino acids [7]. The average solvent-exposed value of tryptophan residue, calculated for globular proteins, is 0.19, which is much smaller than that of other charged amino acid residues, such as glutamate (0.50) [8, 9]. Therefore, tryptophan has a greater probability of being buried inside the proteins compared with other AA residues [10]. Nevertheless, tryptophan exists in almost all proteins [7]. Additionally, tryptophan residues tend to participate in the recognition of molecules interacting with enzymes and proteins [11–14]. Furthermore, tryptophan affords exclusive site selectivity owing to the presence of a unique indole ring, an electron-rich aromatic ring, possessing distinct chemical reactivity [12]. Since the 1960s, diffuse occurrence of tryptophan residues in heme- and/or metal-coordination sites of proteins has detected widespread metal-catalyzed oxidations [15–17]. On the other hand, the occurrence of accessible tryptophan residues in proteins present in close proximity to reactive oxygen species (ROS) promotes their selective oxidative modifications [18, 19]. Besides, tryptophan residue can also be nitrated [19, 20] or mutated to another amino acid, such as arginine (Arg) [21], glycine (Gly) [22]. Substitution of Arg for Trp at position 37 of hemoglobin greatly reduced its cooperativity and oxygen affinity [21], whereas the mutation of Trp to Gly resulted in a greater oxygen affinity than normal [22]. Collectively, these findings suggest the great importance of identifying tryptophan variants.

Shotgun proteomics, in which proteins are enzymatically digested and analyzed *via* liquid chromatography–high resolution mass spectrometry (LC-HRMS), is one of the most widely used approaches to analyze PTMs [23, 24]. Unlike the restricted search engines, the open search engines enlarge the search space with more types of modifications to retrieve more peptide candidates [23, 25]. To develop a comprehensive list of tryptophan modifications, we carried out a shotgun-based open search proteomic of 139 human tissues and primary cells. Our analysis not only provides the most comprehensive map of the tryptophan modifications to date, spanning nearly the full depth of the tryptophan modifications, but also highlights the predominant role of oxidative modifications in hemoglobin (HBB) and glyceraldehyde-3-phosphate dehydrogenase (GAPDH), mainly associated with tumor hypoxia. An independent cohort was further utilized to evaluate the potential prognostic value of the biomarkers of these oxidative tryptophan modifications, which reflect the hypoxia and metabolic dysregulation involved in lung tumor.

## 2. Materials and Methods

### 2.1. Sample Preparation of NSCLC Tissue for LC-MS/MS

Histologically-confirmed human nonsmall cell lung cancer (NSCLC) tissues and paired noncancerous adjacent tissues from 27 patients (27 × 2 samples) were received from the tissue band of the First Affiliated Hospital of Zhengzhou University, Zhengzhou, China. The protocols for analysis of the samples were approved by the Institutional Research Ethics Review Boards. The tumor and adjacent lung tissues (27 × 2 samples) were ground in liquid nitrogen and homogenized with Dounce in ice-cold RIPA buffer. The soluble proteins were collected by centrifugation, and their concentration was measured using Bio-Rad BCA protein assay kit. Approximately, 100 *μ*g of the soluble proteins was precipitated with -80°C acetone and resuspended in 10 mM dithiothreitol and alkylated with 50 mM iodoacetamide. Proteins were digested with sequencing grade trypsin at an enzyme-to-substrate ratio of 1 : 100 overnight at 37°C. The peptides were desalted with the C18 column (Sigma-Aldrich) and fractionated using high-pH reversed-phase chromatography [26]. Briefly, approximately 50 *μ*g of the desalted peptides was resuspended in 160 *μ*L ammonia water (pH = 10.0) and loaded on the column filled with C18 reverse-phase medium (Agela Technologies). The column was eluted with increasing concentrations of acetonitrile (6−50%) in ammonia water. A total of 10 fractions were collected, lyophilized, and stored at −80°C for further use.

### 2.2. LC-MS/MS-Based Analysis of Peptides

**Figure 1:**
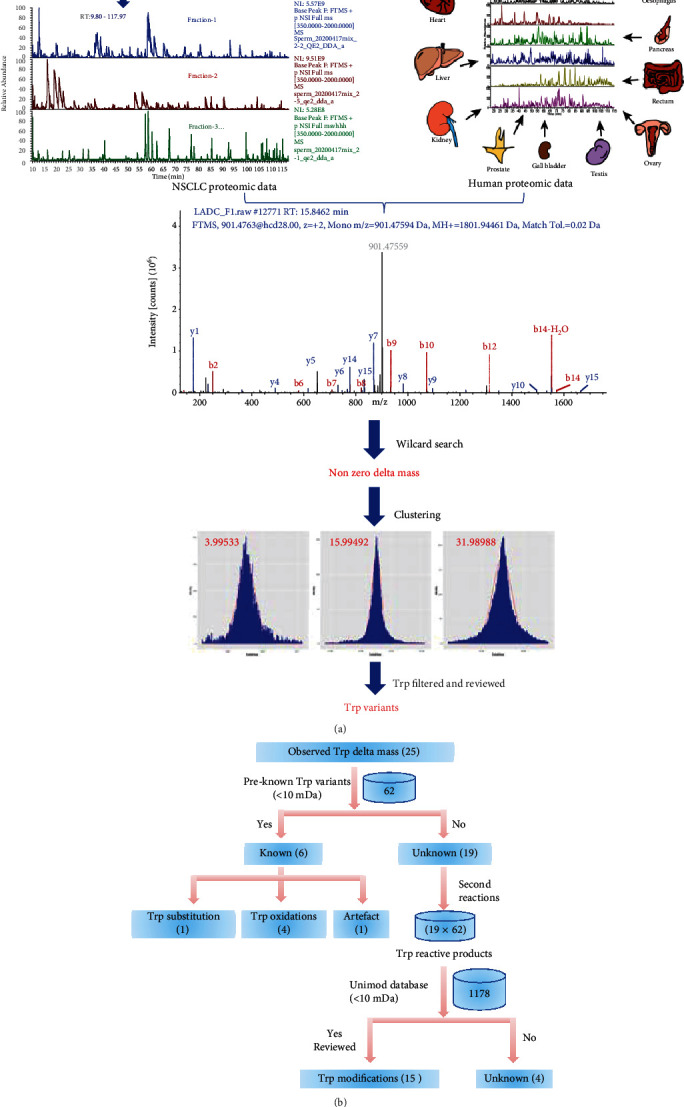
Algorithms for comprehensive identification of Trp variants by wildcard search-based proteomics. (a). NSCLC samples were digested, fractionated, and analyzed on the high-resolution and high-accuracy Orbitrap mass analyser. The human proteomics data (dataset identifier PXD000561, “A draft map of the human proteome” doi:10.1038/nature13302) were downloaded from the Proteome Xchange Consortium (http://proteomecentral.proteomexchange.org). Tandem mass spectrometry data were searched against the human UniProt database (only reviewed entries, human 20350 entries, proteome UP000005640, downloaded April 2020) using Byonic wilcard search algorithm. The obtained nonzero delta mass was clustered (Gaussian mixture model); then the tryptophan modifications with high confident peptide spectrum matches were filtered. (b). Workflow for the tryptophan modification annotation. The 62 preknown Trp variants are shown in Supplementary Table 2.

For label-free proteomic analysis, trypsin-digested peptides were dissolved with 0.1% formic acid and analyzed by EASY-nLC1000 LTQ-Orbitrap Elite mass spectrometer (Thermo Fisher Scientific). The reversed-phase C18 column (Reprosil 3 *μ*m, 250 × *φ*0.075 mm) was eluted with a 180 min linear gradient of 5−35% acetonitrile at a flow rate of 250 nL/min (solvent A: 0.1% formic acid in water; solvent B: 0.1% formic acid in acetonitrile). The flex nanospray was used in positive mode, and the spray voltage was set to 2.30 kV using stainless steel emitters. The temperature of the transfer capillary was 250°C. The HCD collision energy was 27.5 V, and the MS spectra from 350 to 1800 m/z were acquired in the DDA mode with a resolution of 60,000. The 30 most intense ions were selected for MS/MS in the CID mode using ion trap. Normalized collision energies were 35% with 10 ms. The maximum precursor ion injection time was 100 ms. The repeat count was 2, and the dynamic exclusion duration was 90 s. The minimal ion count threshold was 1000. The acquired raw data (NSCLC proteomic data, Figure 1(a)) for 10 fractions of each sample were then analyzed by a wildcard open search using the Byonic software (v3.8) [25].

### 2.3. Identification of the Tryptophan Variants

To compare with normal tissues and cells, a total of 39 HRMS proteomics data (human proteomic data, Figure 1(a)) from 22 histologically normal human cell and tissue types were download from the ProteomeXchange Consortium (http://proteomecentral.proteomexchange.org) via the PRIDE partner repository with the dataset identifier PXD000561 [27]. Both the NSCLC proteomic data [28] and the human proteomic data [27] were analyzed against the UniProt database (20350 entries, human proteome UP000005640, April 2020) by a wildcard open search using the Byonic software (v3.8) [25]. The tolerance was set to 10 ppm for precursor ions, and 0.6 Da for the fragment ions. Two missed cleavages were allowed for trypsin digestions. The delta masses between coding and the observed amino acids were set from -150 to 500 Da and filtered using the criteria: Score ≥ 300; Delta Mod Score ≥ 10; FDR 2D ≤ 0.01. To determine the tryptophan variants, the delta masses were analyzed by the Bayesian Information Criterion (BIC) [29] followed by Gaussian mixture model [30] with 1 Da intervals from -150 to 500 Da. The clustered peaks with the expected delta masses and sufficient values of the goodness-of-fit (*R*^2^) were identified as the qualified mass differences between the coding and the observed amino acids, which were considered different modifications or amino acid variations. A list of peptides containing the confident delta masses at the tryptophan residues were identified and termed tryptophan variants (Supplementary Table 1).

### 2.4. Annotation of the Tryptophan Variants

Based on the Unimod database (http://www.unimod.org/) and reference [31], a tryptophan modification database (62 preknown Trp variants, Supplementary Table 2) was first constructed for the annotation. We initially mapped the 25 tryptophan variants against the tryptophan modification database, resulting in the identification of six known modifications (error < 10 mDa, Table 1). The unmatched delta mass was further annotated according to biological cascade reactions. Accordingly, the obtained 1178 delta mass (19 unknown delta mass minus 62 preknown delta mass) was mapped against the 1514 entries of protein modifications (April 2021) in the Unimod database (error < 10 mDa). The resulted delta mass pair for each unknown Trp variant was reviewed, and the reasonable reactions were finally chosen to annotate the unknown Trp variants.

### 2.5. Gene Ontology (GO) and Network-Based Analyses

Latest GO database (https://www.ebi.ac.uk/QuickGO/) was used for GO enrichment analysis. For functional enrichment analysis, lists of interesting proteins were submitted for enrichment analysis using the UniProt database (20350 entries, human proteome UP000005640, April 2020) as a background dataset. All terms displayed had a *p* < 0.05. ClueGO, a Cytoscape plug-in, was used to decipher functionally grouped pathway annotation networks of the tryptophan-modified proteins [32]. For network-based analyses, protein lists were submitted to the STRING database (http://www.string-db.org/) [33]. The network was exported into a simple tabular output then imported into Cytoscape software (http://cytoscape.org/) for visualization [34].

### 2.6. Statistical Analysis

The R framework (version 4.0.3) was used to perform all statistical analyses of the bioinformatics data. Spearman correlation matrix was generated using “corr.test” function within the package “psych” (2.0.12) and visualized using the “ggcorrplot” function within the package “ggcorrplot” (0.1.3) and “pheatmap” (1.0.12). GO annotations were performed using the R package “clusterProfiler” (3.14.3) with the “enrichGO” function [35].

### 2.7. Survival Analysis

Kaplan-Meier survival curves (log-rank test) were used for overall survival (OS) or disease-free survival (DFS) of the proteomic subtypes and patients with Trp313 oxidation in GAPDH, Trp16, and Trp38 oxidations in HBB. Prior to the log-rank test, survminer 0.4.9 R package was applied to determine the optimal cut-off level for the selected samples. DFS or OS curves were then calculated (Kaplan-Meier analysis and log-rank test) based on the optimal cut-off level.

### 2.8. Molecular Docking

Molecular docking was conducted using MOE v2018.0101 [36]. The 3D structures of the proteins GAPDH and HBB were downloaded from the RCSB Protein Data Bank with PDB ID of 6M61 [37] and 1CBL [38], respectively. The 313W site of GAPDH and W16 and W38 sites of HBB were modified, respectively, and then, the energy of the modified structure was minimized to obtain the modified 3D protein structures. Prior to docking, the force field of AMBER10: EHT, and the implicit solvation model of Reaction Field (R-field) were selected. MOE-Dock was used for molecular docking simulations of the proteins with compounds.

The “induced fit” protocol was selected, wherein the side chains of the binding site in the receptor were allowed to move according to ligand conformations, and a constraint was applied on their positions. The weight used for tethering the side chain atoms to their original positions was 10. Firstly, all docked poses were ranked by London dG scoring function; then, force field refinement was applied on the top 30 poses followed by a rescoring of GBVI/WSA dG scoring function. The conformation with the lowest binding free energy was finally identified as the best probable binding mode.

## 3. Results and Discussion

### 3.1. Widespread Tryptophan Variants Identified by a Modified Open Search Approach

The tryptophan moiety in proteins is presumably reactive to many cellular metabolites that would generate a variety of tryptophan derivatives essential for protein conformation and functions. In this study, the shotgun proteomics and a modified open search approach were adapted to systematically identify any possible variations of tryptophan in the human proteome (termed tryptophan variants) in patients with nonsmall cell lung cancer (NSCLC). Briefly, total proteins of the tumor and adjacent tissues were digested with trypsin, fractionated, and analyzed with high-resolution LC-HRMS. Mass differences between the coding and observed amino acids were identified by a wildcard search using Byonic [25] against human UniProt database (Figure 1(a)). This generated a total of 2,153,367 nonzero delta masses accumulatively from the downloaded human proteomic data and the NSCLC proteomic data. To identify tryptophan variants, these delta masses were subsequently grouped with multivariate clustering followed by Gaussian regression [39], resulting in 25 unique delta mass clusters at tryptophan from 11,769 high-confident MS/MS spectra spread over 2481 tryptophan sites on 858 proteins (Supplementary Figure 1, Supplementary Table 1). These clustered delta masses of tryptophan were reasonably caused by different chemical modifications or structural alterations, reflecting the variants of tryptophan at the protein level.

### 3.2. Tryptophan Variants Largely due to Electrophilic Attacks of the Indole Moiety

**Figure 2:**
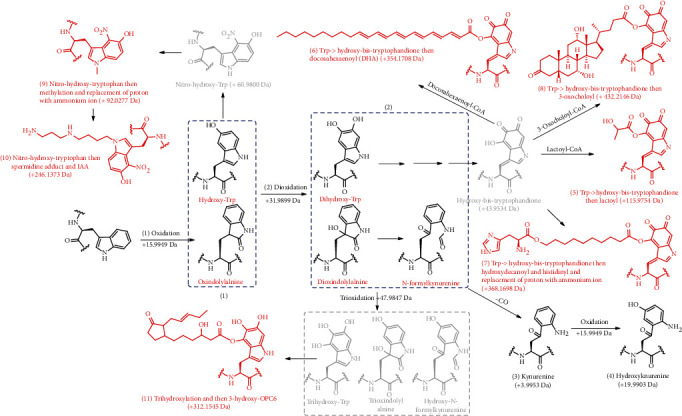
Proposed pathways of reactive species-induced modifications at tryptophan residue. The red-colored structures indicate the potential new modifications at tryptophan residue; the structures in grey color show the intermediates of the tryptophan modification pathway; the structures in black color show the preidentified tryptophan modifications.

As the accuracy of the clustered delta masses was greatly increased by clustering and regression (mass tolerance < 0.005 Da), it enabled more confident annotation simply based on the molecular weights and structure of the putative tryptophan variants (Figure 1(b)). By searching the database for known tryptophan variants (Supplementary Table 2), 6 of the 25 delta mass clusters matched the previously reported tryptophan modifications (Table 1, Figure 2, Supplementary Figure 2), including monooxidation (W+15.9949, 1) and di-oxidation (W+31.9899, 2) of tryptophan, tryptophan->kynurenine substitution (W+3.9953, 3), tryptophan->hydroxy-kynurenine substitution (W+19.9903, 4), tryptophan->Leu/Ile substitution (W-72.9961, 14), and dithiothreitol adduct (W+209.0179, 21). Herein, monooxidation includes keto and hydroxyl modifications of tryptophan, and di-oxidation includes quinonyl and dihydroxyl modifications of tryptophan. Additionally, dithiothreitol adduct was the artefact product derived from the dithiothreitol during the sample preparation.

The unknown delta mass clusters were assumed to be the products of stepwise reactions of the indole sidechain (Figure 1(b)). Because of the electron enrichment by oxidation, the HO-moiety indole moiety was found more vulnerable to attacks by electrophilic metabolites, leading to multiple modification productions of tryptophan (Figure 2). In this line, the clustered delta masses of W+115.9754 (5), W+354.1708 (6), W+368.1698 (7), and W+432.2146 (8) matched several acyl-modifications of oxidized tryptophan. In brief, dihydroxyl-tryptophan (2) might react with docosahexenoic acid (DHA), an essential compound for brain function [40], to generate docosahexenoyl-tryptophandione, suggesting the occurrence of W+354.1708 (6) in the proteome. Similarly, dihydroxyl-tryptophan might react with lactic acid, 10-hydroxydecanoic acid, and 3-oxocholic acid to generate lactoyl-tryptophandione (W+115.9754, 5), histidinyl-decanoyl-tryptophandione (W+368.1698, 7), and 3-oxocholoyl-tryptophandione (W+432.2146, 8), respectively. Notably, more-step reactions might occur after the mono-/trioxidized tryptophan in the human proteome. These included nitro-spermidinyl-tryptophan (W+246.1373, 10) and nitro-methyl-tryptophan (W+92.0277, 9) following monooxidation and hydroxyl-OPC6-tryptophan following tri-oxidation (W+312.1545, 11). Thus, these data agreed with our hypothesis that the oxidation products of tryptophan are the essential intermediates of many unknown tryptophan variants, and the dihydroxyl-tryptophan is apparently more vulnerable to further modifications. Following the same line, the nucleophilic nitrogen of the indole moiety is another potential site for electrophilic attacks. A good example was the clustered delta mass W+493.2657 (Supplementary Figure 2(A), 1(2)), which may be attributable to glycosylation of tryptophan by hexosamine followed by its reaction with levuglandinyl-lactam to contribute to the observed delta mass. Note that these two small molecules have been shown to be important metabolites for the hexosamine and levuglandin pathways [41]. Additionally, certain unknown delta mass clusters may involve amino acid substitutions prior to modifications. For instance, replacement of the tryptophan with histidine, aspartate, cysteine, and tyrosine, followed by histidine oxidation, aspartate benzoylation, cysteine thiobutyroylation, and tyrosine formylation was shown to be responsible for the tryptophan variants of W-59.0506 (15), W+32.9748 (18), W-13.0308 (17), and W+4.9792 (16), respectively. For the same reason, the tryptophan variants of W+88.9942 (19) and W+67.0058 (20) matched multiple reactions after Trp to Thr substitution (Supplementary Figure 2(B)).

Overall, the total frequency of all identified modifications was 11725 (Table 1); among these, 93.16% of tryptophan variants (total frequency: 10923) had resulted from primary and secondary modifications by reactive species, 2.74% (total frequency: 321) was due to amino acid substitutions, 0.32% (total frequency: 37) was due to direct chemical reaction with *in vivo* metabolites, 3.14% (total frequency: 368) was due to unknown reasons (Supplementary Figure 3A), and 0.65% (total frequency: 76) represented the artefact product derived from dithiothreitol during sample preparation. Thus, reactive species-induced modifications (Supplementary Figure 3(B)) were the most common nature of tryptophan variants in human proteomes. Collectively, our data suggested that the mechanisms for tryptophan variants were largely due to the vulnerability of the indole moiety to electrophilic attacks, which was significantly enhanced by single, double, or triple oxidization of indole (Table 1). Additionally, presubstitution of tryptophan by other amino acids followed by further reactions was another mechanism.

### 3.3. Tryptophan Variants Dominantly Found in Hemoglobins in Blood Microparticle

**Figure 3:**
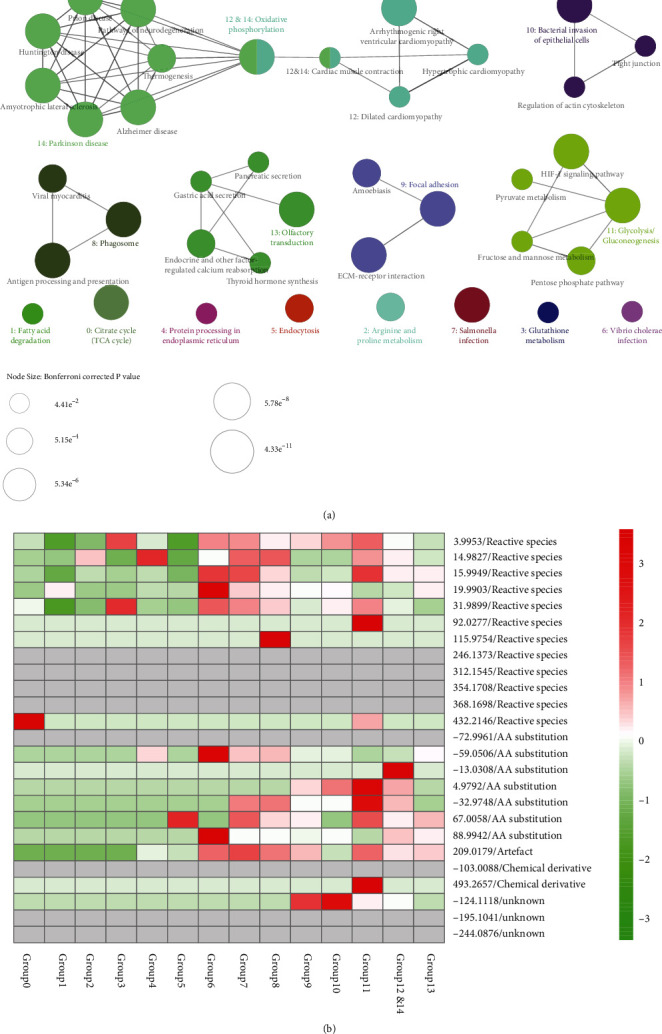
Proteins with tryptophan variants were largely clustered in cytoplasmic glycolysis pathway. (a) Functionally grouped KEGG pathways of the modified-tryptophan-containing proteins with terms as nodes linked based on their kappa score level (≥0.4), where the label of the most significant term per group is shown in color and bold. The node size represents the term enrichment significance. Functionally related groups show partial overlap. (b) Heatmap depicting the relative expression levels of the identified modifications in CC groups.

The Trp-modified proteins were grouped into 14 KEGG pathways (Figure 3(a), Supplementary Table 3) on bioinfomatics analysis using ClueGO of Cytoscape [32]. Most Trp oxidations were clustered in the glycolysis-related pathways (Group 11, Figure 3(b)) but not mitochondrion oxidative phosphorylation-related pathways (Groups 12 and 14), although mitochondria are the major source of cellular ROS in eukaryotic cells. The result was consistent with a previous study in which Cys, Pro, and Tyr, instead of Trp, were found to be predominantly oxidized in mitochondrial proteins [42]. Moreover, most Trp oxidations were clustered in the infection-related pathways, such as vibrio cholerae infection (Group 6), salmonella infection (Group 7), and antigen processing and presentation (Group 8). These results are highly consistent with the fact that ROS production is an important mechanism to counteract bacterial infections [43].

**Figure 4:**
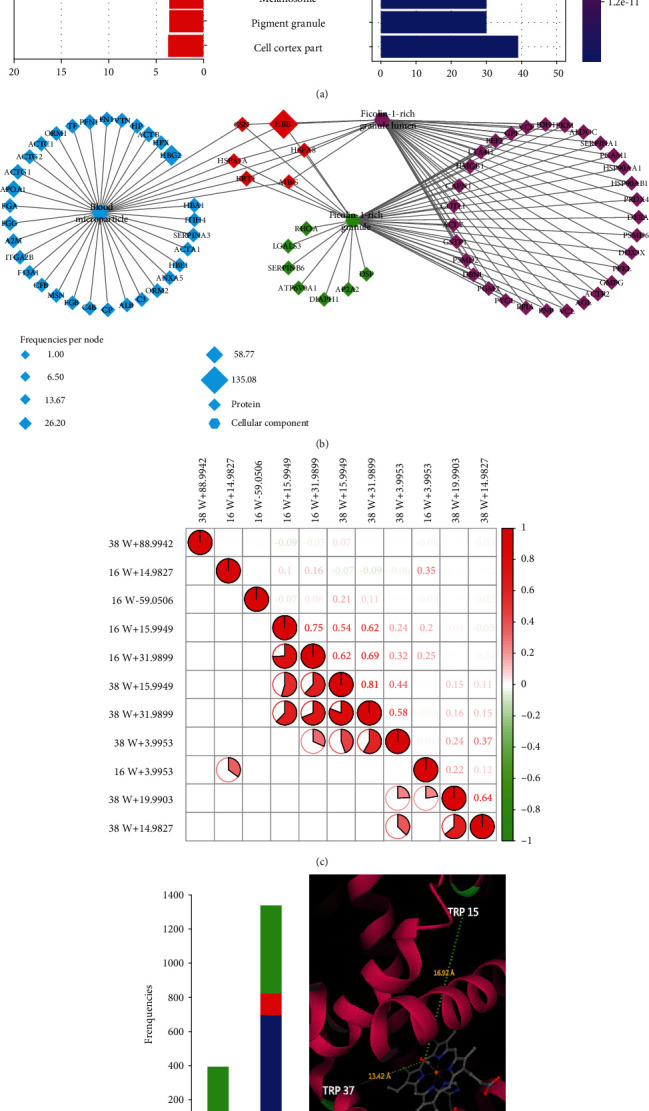
Proteins with tryptophan variants were largely clustered in blood microparticle. (a) Right panel displays the cellular components (CC) from GO enrichment analysis of the modified-tryptophan-containing proteins; left panel shows the frequencies per site of each CC term. (b) Network diagram of the modified-tryptophan-containing proteins mapped to the CC categories. Diamonds represent proteins; octagons represent cellular components. The size of the nodes represents the frequencies per site. (c) Heatmap depicting the Spearman correlation of tryptophan modifications in P68871 (HBB). (d) Color bar represents the relative frequencies of differentially expressed oxidation modification at the 16 W and 38 W sites of P68871 (HBB); the graph shows the overall structure of heme-core in P68871 (PDB: 1HBB). The linear distance between the 15(16)/37(38) Trp sites and heme is shown.

To understand the overall cellular distribution, the proteins with tryptophan polymorphisms were analyzed for their cellular component enrichment. Our data demonstrated that the proteins with tryptophan variants predominantly spanned over blood microparticle and ficolin-1-rich granule (Figure 4(a), Supplementary Table 4). Of note, the iron-containing and oxygen-carrying hemoglobins (HBB and HBG2) within the blood microparticle and ficolin-1-rich granules were the proteins with the highest modification rate (Figure 4(b), >135 per site) and the most abundant tryptophan oxidation (Supplementary Figure 3(C)). For instance, the high frequencies of modifications at Trp16 and Trp38 of HBB (P68871, Supplementary Table 1) and at Trp16, Trp38, and Trp131 of HBG2 (P69892) were presumably due to self-oxidation cascade [44]. Hemoglobin consists of two *α*- and two *β*-subunits containing the heme groups, a reduced ferrous ion in the porphyrin ring [44]. As an efficient O_2_ carrier, the heme group coordinates with four O_2_ molecules reversibly without exchanging electrons. Therefore, the indole moiety of the tryptophan residues in hemoglobin is reasonably vulnerable to self-oxidization by the reduced ferrous ions surrounding the heme-coordination sites [44]. The results indicate that Trp is prone to oxidize due to metacatalysis.

Next, the frequencies of tryptophan variants at different sites of hemoglobins (HBB and HBG2) were analyzed across the human proteome using Spearman correlation. The results demonstrated that the cascade of tryptophan oxidations such as W+3.9953, W+15.9949, W+19.9903, and W+31.9899 were significantly correlated (Figure 4(c) and Supplementary Figure 3(D)). The total oxidation frequencies varied at different tryptophan sites of HBB and were congruent with the sequence of monooxidation, di-oxidation, kynurenine, and hydroxyl-kynurenine (Figure 4(d), left panel), with the highest rates near the heme-coordination site. For instance, HBB at 16 W was detected with higher oxidative rate than HBB at 38 W, apparently due to the distance with the heme group (Figure 4(d), right panel). HBG2 at 16 W and 38 W was more sensitive to oxidation than HBG2 at 131 W for the similar reason (Supplementary Figure 3(E)).

Collectively, tryptophan variants were closely associated with the function of the heme proteins in the blood microparticle and were associated with anti-infection and energy metabolism pathways. Our data consistently suggested that the chemical properties or variants of tryptophan in the heme proteins were mainly regulated by self-catalyzed oxidation. Generally, our data suggested that the self-catalyzed oxidation was prone to target the tryptophan residues in the close proximity of the metal-coordination sites of HBB and HBG2 [14, 18, 19, 44]. The results improved our understanding of the diversity of distribution of Trp modifications, leading to the discovery of many Trp modifications with distinguished localization in specific subtypes of pathways.

### 3.4. Tryptophan Variants Closely Associated with Reactive Species in Lung Cancer

**Figure 5:**
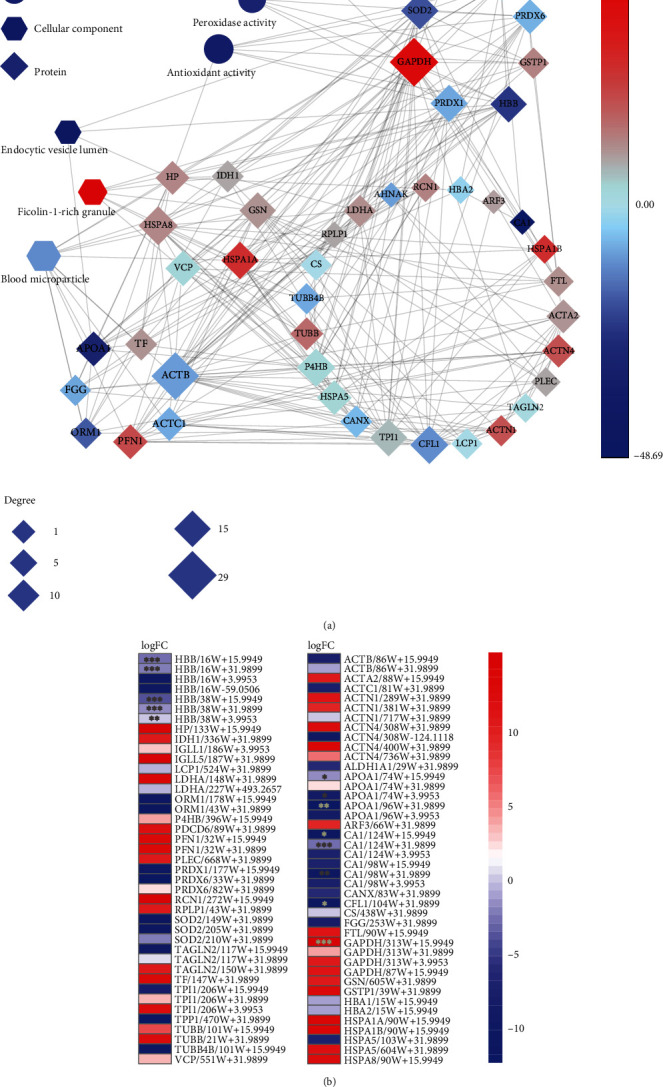
Tryptophan variants associated with antioxidants prone to oxidative stress in NSCLC. (a). Network diagram depicting the interaction of the modified-tryptophan-containing proteins in NSCLC samples that are mapped to the molecular function (MF) and cellular component (CC) categories. Diamonds represent protein, circulars represent MF, and hexagons represent CC. The color of the node corresponds to the fold change (log2) of the modification in each protein site between tumors and the adjacent normal tissues (NATs); red indicates higher expression and blue indicates lower expression in the tumor. The size of the node corresponds to the degree of correlation. (b). Heatmap depicting the modified levels of differentially identified modifications of each protein in lung tumor and adjacent normal tissues. Color bar from blue to red represents the fold change (log2) of modified level from increasing to decreasing. ^∗^*p* < 0.05, ^∗∗^*p* < 0.01, ^∗∗∗^*p* < 0.001.

Reactive species are the byproducts of cellular metabolism known to oxidize proteins and cause cellular oxidative stress [31]. Oxidative stress triggers the antioxidant signaling pathways to eliminate reactive species [45, 46]. We propose that tryptophan variants are associated with oxidative stress induced by reactive species in lung cancer. Indeed, in our study, over 93% of the tryptophan variants was due to reactive species-derived modifications (Supplementary Figure 3(A)) and the majority of the modified proteins were associated with oxidation reactions of hemoglobin in lung cancer (Figure 4(b)). A total of 110 modified tryptophan sites were identified on 53 proteins predominantly in blood microparticle of patients with NSCLC (Supplementary Figure 4(A)). The most commonly modified tryptophan proteins were functionally associated with oxidoreductases, peroxidases, and antioxidant activities (Supplementary Figure 4(A)). Network-based analyses [33, 34] revealed that the occurrence of tryptophan variants in GAPDH, SOD2, HSPA8, ACTB, and HBB was closely connected with the antioxidant network (Figure 5(a)).

**Figure 6:**
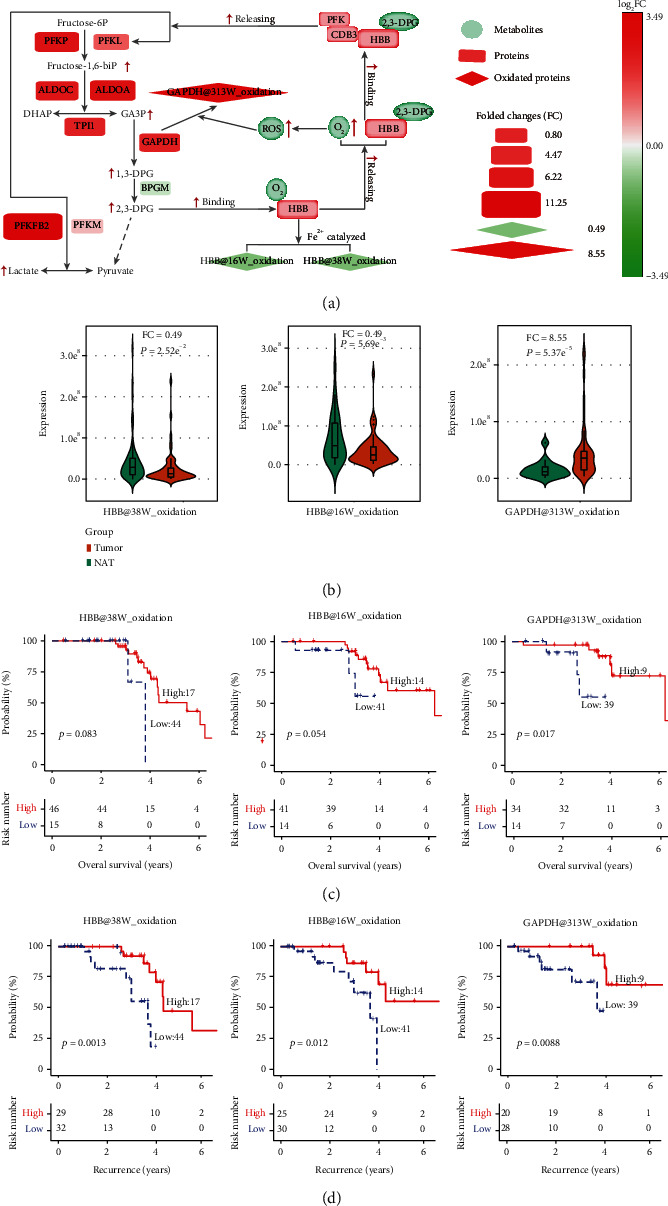
Tryptophan variants associated with the prognosis of NSCLC. (a). Schematic illustration of the mechanism underlying the regulation of various oxidative states of HBB and GAPDH. CDB3: cytoplasmic domain of band 3; 1,3-DPG: glycerate-1,3-diphosphate; 2,3-DPG: glycerate-2,3-diphosphate. (b). Relative expression levels of differentially identified modifications of HBB and GAPDH in tumor samples and adjacent normal tissues (NAT) in an independent cohort of 103 LUAD proteomic dataset (Xu et al., 2020, Cell 182, 245–261). (c). Association of three tryptophan oxidations with overall survival (OS) in an independent cohort of 103 LUAD proteomic dataset (Xu et al., 2020, Cell 182, 245–261), (*p* value from log-rank test). (d). Association of three tryptophan oxidations with disease-free survival (DFS) in an independent cohort of 103 LUAD proteomic dataset (Xu et al., 2020, Cell 182, 245–261), (*p* value from log-rank test).

Consistent with this, the tryptophan modifications at GAPDH at 313 W were found significantly upregulated in tumors compared to that in the adjacent normal tissues (Figure 5(b)). This is consistent with previous studies in which the 313 W of GAPDH was found to be oxidized by the cytosolic ROS during metabolic and oxidative stress [45, 47–49]. The ROS level is typically upregulated in the tumor microenvironment [50]. However, the specific oxidation at 16 W and 38 W of HBB (HBB at 16 W, HBB at 38 W) was slightly reduced in lung cancer (Figure 5(b)), probably because HBB at 16 W and HBB at 38 W were self-oxidized near the heme-coordination site (Figure 4(d)). As GAPDH is typically upregulated in cancers as one of the critical enzymes for glycolysis, it catalyzed the oxidative phosphorylation of glyceraldehyde-3-phosphate (G3P) to generate 1,3-diphospho-glycerate (1,3-DPG), which were subsequently changed to 2,3-diphosphoglycerate (2,3-DPG) in the presence of the enzyme diphosphoglycerate mutase (DPGM) [51]. Upregulation of 2,3-DPG in cancers reduces the oxygen affinity leading to release of oxygen from HBB [51]. This explained our data that, while ROS enhanced oxidation at GAPDH at 313 W, the increase of 2,3-DPG suppressed self-oxidation at HBB 16 W and HBB 38 W, suggesting a negative regulation for the hypoxia environment in lung cancer (Figure 6(a)). Nevertheless, ROS-induced GAPDH at 313 W+15.9949 was localized within the catalytic domain, so that it would suppress the GAPDH activity (Figure 6(a)). The docking simulation studies finally confirmed these speculations (Supplementary Figure 5). The docking scores showed that the binding affinity of GAPDH at 313 W for NAD^+^ was weaker than that of wild type GAPDH (Supplementary Figure 5(A) and 5(B)), which would suppress the conversion of G3P into 1,3-DPG by GAPDH. In addition, the oxidized HBB-W16/W38 weakened the interactions between HBB and 2,3-bisphosphoglycerate (Supplementary Figure 5(C) and (D)), which enhanced the oxygen affinity and retained the oxygen from HBB (Figure 6(a)). In this context, our data suggested that tryptophan variants provided the negative regulation pathways for the aerobic glycolysis and hypoxia, two typical phenotypes for lung cancer. Finally, these data are consistent with the previously reported upregulation of the glycolytic enzymes in lung adenocarcinoma (LUAD) at the proteomic level [52]. In LUAD proteomic data [52], the expressions of PFKP, PFKL, ALDOA, ALDOC, TPI1, GAPDH, and PFKFB2 were significantly upregulated in tumor samples (Supplementary Figure 4(B)).

### 3.5. Tryptophan Variants as Biomarkers for the Prognosis of Lung Cancer

As most tryptophan variants were associated with reactive species in lung cancer, we reasoned whether the specific tryptophan modifications of GAPDH at 313 W oxidation, HBB at 16 W oxidation, and HBB at 38 W oxidations could be developed as prognostic biomarkers. To this end, 103 lung adenocarcinoma samples from an independent cohort were examined [52]. The results further confirmed that oxidation of GAPDH at 313 W was significantly upregulated, while oxidation of HBB at 16 W and HBB at 38 W was downregulated in tumor samples (Figure 6(b)). Moreover, patients with higher levels of GAPDH at 313 W oxidation and lower levels of HBB at 16 W+15.9949 and HBB at 38 W+15.9949 had better prognostic outcomes (Figures 6(c)and 6(d), log-rank test, *p* < 0.05). Therefore, the divergent oxidative tryptophan levels of GAPDH and HBB, mainly attributed to the aerobic glycolysis and hypoxia in lung tumor, presented a biomarker panel with potential prognostic relevance with respect to monitoring tumor progression, evaluating therapeutic response, and developing clinic treatments.

While hypoxia is known to induce ROS accumulation and lead to a diverse array of dysregulated signals, including aerobic glycolysis, oxidative stress, apoptosis, and eventually cell death, it also induces antioxidant measures to counteract ROS-induced damage [50]. Since the observed Trp oxidations are widely and naturally occurring and originate from ROS, it can be assumed that the biological impact of hypoxia may in part be mediated *via* oxidation of cellular proteins. Tryptophan variants are presumed as a negative feedback mechanism to counteract the glycolysis and hypoxia in lung cancer. On one the hand, the upregulation of ROS-induced GAPDH at 313 W oxidation may reduce the GAPDH activity and subsequently suppress GAPDH-mediated glycolytic pathways resulting in downregulation of self-oxidation of HBB. On the other hand, the downregulation of self-oxidation of HBB at 16 W and HBB at 38 W increases the oxygen affinity of HBB and leads to the suppression of ROS-induced hypoxia in cancers. Moreover, tumor hypoxia is an independent marker of poor prognosis for cancer [50, 53, 54], and targeting hypoxia is important for therapeutic outcome. Therefore, tryptophan variants, predominantly oxidative modifications at GAPDH at 313 W, HBB at 16 W, and HBB at 38 W represent a new avenue as potential therapeutic targets and prognostic biomarkers for patients with NSCLC.

## 4. Conclusions

Widespread tryptophan variations or tryptophan variants were identified across the proteome of patients with nonsmall cell lung cancer (NSCLC). These included 25 different tryptophan variants mapped to 2481 sites on 858 proteins, mostly involved in the cascade of oxidation in the blood microparticle, predominantly associated with reactive species in response to anaerobic glycolysis and hypoxia. The divergent Trp oxidation in HBB and GAPDH reflects and regulates the hypoxia and metabolic dysregulation involved in cancer cells, which are of potential prognostic relevance in the context of NSCLC. Overall, the results improve our understanding of the widespread tryptophan variations and highlight the predominant role of tryptophan oxidation at GAPDH at 313 W, HBB at 16 W, and HBB at 38 W in regulating redox balance of cancer cells and their potential role as prognostic biomarkers in patients with NSCLC.

## Figures and Tables

**Table 1:** Trp polymorphisms: list of the delta mass peaks at the Trp residue.

No.^a^	Classification^b^	Delta mass^c^	Frequency	Annotation	Chemical formula	Monoisotopic mass^d^	Error (Da)
1	Reactive species	15.9949	2872	Oxidation	O	15.9949	0.00
2	Reactive species	31.9899	5965	Dihydroxylation	O(2)	31.9898	1.00e^−4^
3	Reactive species	3.9953	2029	Trp->Kynurenin	C(-1)O	3.9949	4.00e^−4^
4	Reactive species	19.9903	45	Trp->Hydroxykynurenin	C(-1)O(2)	19.9998	-9.50e^−3^
5∗	Reactive species	115.9754	2	Trp->Hydroxy-bis-tryptophandione then lactoyl	C(3)O(5)	115.9746	8.00e^−4^
6∗	Reactive species	354.1708	2	Trp->Hydroxy-bis-tryptophandione then docosahexaenoyl	C(22)H(26)O(4)	354.1831	-1.23e^−2^
7∗	Reactive species	368.1698	4	Trp->Hydroxy-bis-tryptophandione then hydroxydecanoyl and histidinyl and replacement of proton with ammonium ion	C(16)H(24)N(4)O(6)	368.1696	2.00e^−4^
8∗	Reactive species	432.2146	1	Trp->Hydroxy-bis-tryptophandione then 3-oxocholoyl	C(24)H(32)O(7)	432.2148	-2.00e^−4^
9∗	Reactive species	92.0277	1	Nitro-hydroxy-tryptophan then methylation then replacement of proton with ammonium ion	C(1)H(4)N(2)O(3)	92.0222	5.50e^−3^
10∗	Reactive species	246.1373	1	Nitro-hydroxy-tryptophan then spermidine adduct and IAA	C(9)H(18)N(4)O(4)	246.1328	4.50e^−3^
11∗	Reactive species	312.1545	1	Trihydroxylation and then 3-hydroxy-OPC6	C(12)H(20)N(6)O(4)	312.1546	-1.00e^−4^
12∗	Chemical derivative	493.2657	35	Hexosamine and levuglandinyl-lysine lactam adduct	C(26)H(39)NO(8)	493.2676	-1.90e^−3^
13	Chemical derivative	103.0088	2	Cysteine	C(3)H(5)NOS	103.0092	-4.00e^−4^
14	AA substitution	-72.9961	22	Trp->Leu/Ile substitution	HC(-5)N(-1)	-72.9953	-8.00e^−4^
15∗	AA substitution	-59.0506	16	Trp->His then aspartylurea	C(-6)H(-5)N(-1)O(2)	-59.0524	1.80e^−3^
16∗	AA substitution	4.9792	32	Trp- > Tyr then formylation	C(-1)H(-1)N(-1)O(2)	4.9790	2.00e^−4^
17∗	AA substitution	-13.0308	11	Trp- > Cys then corotonaldehyde	C(-4)HN(-1)OS	-13.0283	-2.50e^−3^
18∗	AA substitution	32.9748	204	Trp->Asp then benzoyltion	H(-1)N(-1)O(3)	32.9739	9.00e^−4^
19∗	AA substitution	88.9942	20	Trp->Thr then citryltion	C(-1)H(3)N(-1)O(7)	88.9848	9.40e^−3^
20∗	AA substitution	67.0058	16	Trp->Thr then 3-oxo-5,6-dehydrosuberyl semialdehyde	CH(5)N(-1)O(4)	67.0157	-9.90e^−3^
21	Artefact	209.0179	76	Carbamidomethylated DTT modification	H(11)C(6)NO(3)S(2)	209.0180	-1.00e^−4^
22	Unknown	-124.1118	10	Unknown	C(-8)H(-14)N	-124.1126	8.00e^−4^
23	Unknown	14.9827	352	Unknown	OH(-1)	14.9871	-4.40e^−3^
24	Unknown	195.1041	1	Unknown	C(14)H(13)N	195.1048	-7.00e^−4^
25	Unknown	244.0876	5	Unknown	C(10)H(14)NO(6)	244.0821	5.50e^−3^

^a^Newly annotated delta masses are marked with ^“^∗^”^. ^b^The modifications of tryptophan relevant to reactive species account for 10923 of 11725 total modification events (93.16%). ^c^The delta mass peaks are defined by review after clustering of the measured delta masses from the open search results of 139 HRMS proteomics data, which reflecting the potential Trp polymorphisms. ^d^Predicted monoisotopic mass based on the annotation.

## Data Availability

All data used to support the findings of this study are included within the article and the supplementary information file.

## Supplementary Materials

Supplementary Materials Supplementary Figure 1: peptide spectrum matches (PSMs) of the identified 25 Trp variants. Supplementary Figure 2: proposed pathways of chemical reaction with *in vivo* metabolites (A) and tryptophan substitutions (B). The red-colored structures indicate the potential new modifications at tryptophan residue; the structures in grey color show the intermediates of the tryptophan modification pathway; the structures in black color show the preidentified tryptophan modifications. Supplementary Figure 3: proteins with tryptophan variants were largely clustered in blood microparticle, related to Figure 4. (a) Relative frequencies of each tryptophan modification group in the dataset. (b) Relative frequencies of delta mass clusters in the dataset. (c) Relative frequencies of each protein in the cellular components of blood microparticle, ficolin-1-rich granule lumen, and ficolin-1-rich granule. D. Heatmap depicting the correlation of tryptophan modifications in P69892 (HBG2). E. Color bar represents the relative frequency of differentially expressed oxidation modification at the 16 W, 38 W, and 131 W sites of P69892 (HBG2); the graph shows the overall structure of heme-core in P69892 (PDB: 4MQK). The linear distances of the 16 W, 38 W, and 131 W sites from the heme group are shown. Supplementary Figure 4: tryptophan variants associated with antioxidants prone to oxidative stress in NSCLC. A. Gene Ontology enrichment analysis of the modified-tryptophan-containing proteins in NSCLC, related to Figure 5(a); B. Relative expression levels of glycolytic enzymes in tumor samples and adjacent normal tissues in an independent cohort of 103 LUAD proteomic dataset (Xu et al., 2020, Cell 182, 245–261), related to Figure 6. Supplementary Figure 5: molecular docking of GAPDH (PubChem CID: 6 M61) with NAD^+^ and HBB (PubChem CID: 1CBL) with 2,3-diphosphoglycerate before and after oxidation, respectively. A. The 3D binding mode of NAD^+^ with GAPDH-wt; B. The 3D binding mode of NAD^+^ with GAPDH-wt. NAD^+^ is colored in yellow. The surrounding residues in the binding pocket are colored in light blue. The backbone of the receptor is depicted as light blue cartoon. C. The 3D binding mode of 2,3-diphosphoglycerate with HBB-wt. D. The 3D binding mode of 2,3-diphosphoglycerate with HBB-W16/W38. The 2,3-diphosphoglycerate is colored in yellow. The surrounding residues in the binding pocket are colored in light blue. The backbone and residue of HBB-wt alpha chain is colored in light blue, and the backbone and residue of HBB-wt beta chain is colored in white. The hydrogen bonds are depicted as yellow dashed lines. The salt bridges are depicted as magenta dashed lines. Supplementary Tables. Supplementary Table 1: the dataset of tryptophan modification. Supplementary Table 2: the preidentified Trp modifications was constructed based on the Unimod database (http://www.unimod.org/) and references [Bachi A, et al., Chem Rev 2013, 113 : 596-698.], related to Figure 1(b). Supplementary Table 3: the dataset of functionally grouped KEGG pathways of the modified-tryptophan-containing proteins. Supplementary Table 4: the dataset of cellular components of the modified-tryptophan-containing proteins and the corresponding frequencies per site, related to Figure 4.
