# Supplementary material for: Open Search-Based Proteomics Reveals Widespread Tryptophan Modifications Associated with Hypoxia in Lung Cancer
Source: Oxid Med Cell Longev. 2022 Apr 30;2022:2590198. doi: 10.1155/2022/2590198 (PMC9078843; doi:10.1155/2022/2590198)
Supplement: Supplementary Materials — Supplementary Figure 1: peptide spectrum matches (PSMs) of the identified 25 Trp variants. Supplementary Figure 2: proposed pathways of chemical reaction with in vivo metabolites (A) and tryptophan substitutions (B). The red-colored structures indicate the potential new modifications at tryptophan residue; the structures in grey color show the intermediates of the tryptophan modification pathway; the structures in black color show the preidentified tryptophan modifications. Supplementary Figure 3: proteins with tryptophan variants were largely clustered in blood microparticle, related to Figure 4. (a) Relative frequencies of each tryptophan modification group in the dataset. (b) Relative frequencies of delta mass clusters in the dataset. (c) Relative frequencies of each protein in the cellular components of blood microparticle, ficolin-1-rich granule lumen, and ficolin-1-rich granule. D. Heatmap depicting the correlation of tryptophan modifications in P69892 (HBG2). E. Color bar represents the relative frequency of differentially expressed oxidation modification at the 16 W, 38 W, and 131 W sites of P69892 (HBG2); the graph shows the overall structure of heme-core in P69892 (PDB: 4MQK). The linear distances of the 16 W, 38 W, and 131 W sites from the heme group are shown. Supplementary Figure 4: tryptophan variants associated with antioxidants prone to oxidative stress in NSCLC. A. Gene Ontology enrichment analysis of the modified-tryptophan-containing proteins in NSCLC, related to Figure 5(a); B. Relative expression levels of glycolytic enzymes in tumor samples and adjacent normal tissues in an independent cohort of 103 LUAD proteomic dataset (Xu et al., 2020, Cell 182, 245–261), related to Figure 6. Supplementary Figure 5: molecular docking of GAPDH (PubChem CID: 6 M61) with NAD+ and HBB (PubChem CID: 1CBL) with 2,3-diphosphoglycerate before and after oxidation, respectively. A. The 3D binding mode of NAD+ with GAPDH-wt; B. The 3D binding mode of NAD+ with GAPDH-w [file 2590198.f1.zip › Supplemental Table S1_20220226.pdf]

Supplementary Table 1: The dataset of tryptophan modification.

Note 1 : The 25 delta mass (Δmass) peaks are defined by reviewed after clustering of the measured delta masses from the open search results of 139 HRMS proteomics data, which reflecting the potential Trp polymorphisms.
Note 2 : Pro.ID: Protein ID; Pos.: Position in Protein; Total occ.: Total occurrence.

Δ mass	R.Squared	Pro.ID	Pos.	Total Occ.	Original Sequence	Position in Peptide
15.9949	0.9191695	P68871	38	697	R.LLVVYPWTQR.F	W7(+15.982730081)
31.9899	0.9376182	P68871	38	516	R.LLVVYPWTQR.F	W7(+31.977603128)
15.9949	0.9191695	P69892	38	290	R.LLVVYPWTQR.F	W7(+15.98627012)
31.9899	0.9376182	P68871	16	273	K.SAVTALWGK.V	W7(+31.980847685)
15.9949	0.9191695	P69892	16	229	M.GHFTEEDKATITSLWGK.V	W15(+15.986680626)
31.9899	0.9376182	P69892	131	187	K.EFTPEVQASWQK.M	W10(+31.980573221)
31.9899	0.9376182	P69892	16	163	M.GHFTEEDKATITSLWGK.V	W15(+31.977475543)
31.9899	0.9376182	P01857	196	132	R.VVSVLTVLHQDWLNGK.E	W12(+31.984763178)
3.9953	0.9130741	P68871	38	125	R.LLVVYPWTQR.F	W7(+3.984316995)
32.9748	0.6928963	P01857	196	120	R.VVSVLTVLHQDWLNGK.E	W12(+32.965523818)
15.9949	0.9191695	P04406	313	117	K.LISWYDNEFGYSNR.V	W4(+15.982217676)
31.9899	0.9376182	P01857	160	114	K.FNWYVDGVEVHNAK.T	W3(+31.983941094)
15.9949	0.9191695	P68871	16	105	K.SAVTALWGK.V	W7(+15.986646025)
15.9949	0.9191695	P69892	131	103	K.EFTPEVQASWQK.M	W10(+15.985944314)
31.9899	0.9376182	P60174	206	84	K.VVLAYEPVWAIGTGK.T	W9(+31.985431263)
31.9899	0.9376182	P04406	313	81	K.LISWYDNEFGYSNR.V	W4(+31.980508692)
31.9899	0.9376182	P0DOY2	79	72	K.YAASSYLSLTPEQWK.S	W14(+31.982039844)
31.9899	0.9376182	P69892	38	71	R.LLVVYPWTQR.F	W7(+31.980654886)
31.9899	0.9376182	P30086	8	50	K.WSGPLSLQEVDEQPQHPLHVTYAGAAVDELGK.V	W1(+31.980216648)
31.9899	0.9376182	P00915	124	49	K.YSAELHVAHWNSAK.Y	W10(+31.978273999)
3.9953	0.9130741	P01857	196	48	R.VVSVLTVLHQDWLNGK.E	W12(+3.991097548)
15.9949	0.9191695	P02686	250	47	R.FSWGAEGQR.P	W3(+15.992296097)
31.9899	0.9376182	P02647	96	46	K.LREQLGPVTQEFWDNLEK.E	W13(+31.983065125)
3.9953	0.9130741	P69892	131	44	K.EFTPEVQASWQK.M	W10(+3.992414041)
3.9953	0.9130741	P69892	38	44	R.LLVVYPWTQR.F	W7(+3.990895506)
15.9949	0.9191695	P0DOY2	79	42	K.YAASSYLSLTPEQWK.S	W14(+15.985579883)
31.9899	0.9376182	P14618	482	42	K.DPVQEAWAEDVDLR.V	W7(+31.982358015)
31.9899	0.9376182	P02686	250	41	R.FSWGAEGQRPGFGYGGR.A	W3(+31.978856095)
31.9899	0.9376182	P23528	104	40	K.KEDLVFIFWAPESAPLK.S	W9(+31.983500569)
31.9899	0.9376182	P14136	256	40	R.TQYEAMASSNMHEAEEWYR.S	W17(+31.976800296)
31.9899	0.9376182	P01857	264	40	K.GFYPSDIAVEWESNGQPENNYK.T	W11(+31.980489892)
15.9949	0.9191695	P02647	74	39	K.LLDNWDSVTSTFSK.L	W5(+15.989743239)
31.9899	0.9376182	P63261	86	38	K.IWHHTFYNELR.V	W2(+31.983847045)
31.9899	0.9376182	P30041	82	35	K.LIALSIDSVEDHLAWSK.D	W15(+31.985802232)
31.9899	0.9376182	P08263	21	35	R.WLLAAAGVEFEEK.F	W1(+31.989216311)
31.9899	0.9376182	Q16555	291	35	K.GTVVYGEPITASLGTDGSHYWSK.N	W21(+31.983906393)
493.2657	0.1346338	P00338	227	35	K.TLHPDLGTDKDKEQWK.E	W15(+493.259703849)
32.9748	0.6928963	P01857	264	34	K.GFYPSDIAVEWESNGQPENNYK.T	W11(+32.969747705)
15.9949	0.9191695	P30086	55	33	K.NRPTSISWDGLDSGK.L	W8(+15.988677687)
31.9899	0.9376182	P30086	55	33	K.NRPTSISWDGLDSGK.L	W8(+31.985503859)
31.9899	0.9376182	Q9BVA1	21	33	K.FWEVISDEHGIDPTGSYHGDSDLQLER.I	W2(+31.977933576)
3.9953	0.9130741	P23528	104	32	K.KEDLVFIFWAPESAPLK.S	W9(+3.988749592)
31.9899	0.9376182	P02647	74	32	K.LLDNWDSVTSTFSK.L	W5(+31.984128005)
31.9899	0.9376182	O75390	438	32	R.ALGVLAQLIWSR.A	W10(+31.987182426)
31.9899	0.9376182	P07437	21	32	K.FWEVISDEHGIDPTGTYHGDSDLQLDR.I	W2(+31.970683679)
3.9953	0.9130741	P69892	16	31	M.GHFTEEDKATITSLWGK.V	W15(+3.990464806)
15.9949	0.9191695	P30086	8	31	K.WSGPLSLQEVDEQPQHPLHVTYAGAAVDELGK.V	W1(+15.983878757)
31.9899	0.9376182	P00738	398	31	K.VTSIQDWVQK.T	W7(+31.986158361)
31.9899	0.9376182	P01859	192	31	R.VVSVLTVVHQDWLNGK.E	W12(+31.987948784)
31.9899	0.9376182	P04264	212	31	K.WELLQQVDTSTR.T	W1(+31.988346528)
3.9953	0.9130741	P04406	313	30	K.LISWYDNEFGYSNR.V	W4(+3.989541895)
3.9953	0.9130741	P60174	206	29	K.VVLAYEPVWAIGTGK.T	W9(+3.983478138)
15.9949	0.9191695	P60174	206	29	K.VVLAYEPVWAIGTGK.T	W9(+15.99190099)
31.9899	0.9376182	P0DOY3	79	28	K.YAASSYLSLTPEQWK.S	W14(+31.985213672)
15.9949	0.9191695	P14618	482	26	K.DPVQEAWAEDVDLR.V	W7(+15.982846296)
31.9899	0.9376182	P07437	344	26	K.NSSYFVEWIPNNVK.T	W8(+31.987656571)
15.9949	0.9191695	P01857	160	25	K.FNWYVDGVEVHNAK.T	W3(+15.98839666)
31.9899	0.9376182	Q16555	412	25	R.IAVGSDADLVIWDPDSVK.T	W12(+31.981872675)

31.9899	0.9376182	P09543	310	25	R.VELSEQQLQLWPSDVDK.L	W11(+31.983378822)
15.9949	0.9191695	P01857	196	24	R.VVSVLTVLHQDWLNGK.E	W12(+15.987984755)
15.9949	0.9191695	P63261	86	24	K.IWHHTFYNELR.V	W2(+15.988211058)
31.9899	0.9376182	P12814	717	24	R.VGWEQLLTTIAR.T	W3(+31.985104576)
31.9899	0.9376182	P06396	605	24	K.TPSAAYLWVGTGASEAEK.T	W8(+31.986556173)
31.9899	0.9376182	P60709	86	23	K.IWHHTFYNELR.V	W2(+31.979526835)
31.9899	0.9376182	P09543	167	23	K.NQWQLSADDLK.K	W3(+31.985231654)
31.9899	0.9376182	P00338	148	23	K.LLIVSNPVDILTYVAWK.I	W16(+31.986152498)
31.9899	0.9376182	P21333	2262	23	R.AEAGVPAEFSIWTR.E	W12(+31.982747805)
14.9827	0.2716954	P69892	38	23	R.LLVVYPWTQR.F	W7(+14.979922464)
-72.9961	0.0052843	P05060	381	22	R.APRPQSEESWDEEDKR.N	W10(-72.992970166)
3.9953	0.9130741	P02686	250	22	R.FSWGAEGQRPGFGYGGR.A	W3(+3.990941056)
31.9899	0.9376182	P0DOY2	79	22	K.YAASSYLSLTPEQWK.S	W14(+31.983504687)
31.9899	0.9376182	P18669	16	22	R.HGESAWNLENR.F	W6(+31.986636985)
31.9899	0.9376182	Q13813	1640	22	R.LAALADQWQFLVQK.S	W8(+31.98599712)
31.9899	0.9376182	P11021	604	22	K.IEWLESHQDADIEDFK.A	W3(+31.986358317)
31.9899	0.9376182	P30041	33	22	R.FHDFLGDSWGILFSHPR.D	W9(+31.981932589)
3.9953	0.9130741	P21333	2262	21	R.AEAGVPAEFSIWTR.E	W12(+3.99251343)
15.9949	0.9191695	P00915	124	21	K.YSAELHVAHWNSAK.Y	W10(+15.987185132)
31.9899	0.9376182	P60201	212	21	R.MYGVLPWNAFPGK.V	W7(+31.986296347)
31.9899	0.9376182	P37802	117	21	R.YGINTTDIFQTVDLWEGK.N	W15(+31.986771726)
31.9899	0.9376182	P13796	524	21	K.VNDDIIVNWVNETLR.E	W9(+31.986081483)
14.9827	0.2716954	P69892	16	21	M.GHFTEEDKATITSLWGK.V	W15(+14.978879351)
3.9953	0.9130741	P15814	186	20	K.YAASSYLSLTPEQWR.S	W14(+3.9826057)
31.9899	0.9376182	P68371	344	20	K.NSSYFVEWIPNNVK.T	W8(+31.982773759)
31.9899	0.9376182	P00338	227	20	K.TLHPDLGTDKDKEQWK.E	W15(+31.98214047)
31.9899	0.9376182	Q14195	412	20	R.ISVGSDSDLVIWDPDAVK.I	W12(+31.986835983)
31.9899	0.9376182	P09429	133	20	K.LGEMWNNTAADDKQPYEK.K	W5(+31.985502068)
31.9899	0.9376182	P0DML2	112	20	R.ISLLLIESWLEPVR.F	W9(+31.985962009)
31.9899	0.9376182	P68032	88	19	K.IWHHTFYNELR.V	W2(+31.983847045)
3.9953	0.9130741	P30086	55	18	K.NRPTSISWDGLDSGK.L	W8(+3.987823195)
3.9953	0.9130741	P09543	310	18	R.VELSEQQLQLWPSDVDK.L	W11(+3.987651283)
3.9953	0.9130741	P14618	482	18	K.DPVQEAWAEDVDLR.V	W7(+3.992001569)
15.9949	0.9191695	P0DOY2	79	18	K.YAASSYLSLTPEQWK.S	W14(+15.990706836)
31.9899	0.9376182	P68371	21	18	K.FWEVISDEHGIDPTGTYHGDSDLQLER.I	W2(+31.970573068)
31.9899	0.9376182	P40925	218	18	K.EVGVYEALKDDSWLK.G	W13(+31.985169717)
31.9899	0.9376182	O43707	736	18	R.VGWEQLLTTIAR.T	W3(+31.98669149)
31.9899	0.9376182	P50454	176	18	R.SALQSINEWAAQTTDGKLPEVTK.D	W9(+31.988583669)
14.9827	0.2716954	P69892	131	18	K.EFTPEVQASWQK.M	W10(+14.979230447)
3.9953	0.9130741	P60201	212	17	R.MYGVLPWNAFPGK.V	W7(+3.990446738)
31.9899	0.9376182	P01876	162	17	R.DASGVTFTWTPSSGK.S	W9(+31.984147767)
31.9899	0.9376182	O14880	93	17	R.IASGLGLAWIVGR.V	W9(+31.986719209)
3.9953	0.9130741	P63261	86	16	K.IWHHTFYNELR.V	W2(+3.992710429)
3.9953	0.9130741	Q16555	291	16	K.GTVVYGEPITASLGTDGSHYWSK.N	W21(+3.98754399)
15.9949	0.9191695	P23528	104	16	K.KEDLVFIFWAPESAPLK.S	W9(+15.983256428)
31.9899	0.9376182	Q00610	1556	16	K.DTELAEELLQWFLQEEK.R	W11(+31.979471078)
31.9899	0.9376182	P09972	314	16	R.ALQASALNAWR.G	W10(+31.988140426)
31.9899	0.9376182	P27797	219	16	K.IKDPDASKPEDWDER.A	W12(+31.986505753)
209.0179	0.7899121	P69892	131	16	K.EFTPEVQASWQK.M	W10(+209.016095681)
32.9748	0.6928963	P01859	192	16	R.VVSVLTVVHQDWLNGKEYK.C	W12(+32.970108566)
3.9953	0.9130741	P68871	16	15	K.SAVTALWGK.V	W7(+3.988110868)
3.9953	0.9130741	P06396	605	15	K.TPSAAYLWVGTGASEAEK.T	W8(+3.991438985)
15.9949	0.9191695	P60709	86	15	K.IWHHTFYNELR.V	W2(+15.990133666)
31.9899	0.9376182	P07900	320	15	K.SLTNDWEDHLAVK.H	W6(+31.986229758)
31.9899	0.9376182	P30086	122	15	R.YVWLVYEQDRPLK.C	W3(+31.985795124)
31.9899	0.9376182	Q09666	745	15	K.VDIDAPDVDVHGPDWHLK.M	W15(+31.985541292)
31.9899	0.9376182	P01834	41	15	K.VQWKVDNALQSGNSQESVTEQDSK.D	W3(+31.985046336)
209.0179	0.7899121	P69892	38	15	R.LLVVYPWTQR.F	W7(+209.011782816)
3.9953	0.9130741	P30086	8	14	K.WSGPLSLQEVDEQPQHPLHVTYAGAAVDELGK.V	W1(+3.990056594)
3.9953	0.9130741	P00738	398	14	K.VTSIQDWVQK.T	W7(+3.993238439)
15.9949	0.9191695	P09543	310	14	R.VELSEQQLQLWPSDVDK.L	W11(+15.984843665)
15.9949	0.9191695	P00338	227	14	K.TLHPDLGTDKDKEQWK.E	W15(+15.987744479)
15.9949	0.9191695	P18669	25	14	R.FSGWYDADLSPAGHEEAK.R	W4(+15.992543821)
31.9899	0.9376182	P12277	228	14	K.TFLVWVNEEDHLR.V	W5(+31.982439408)
31.9899	0.9376182	P11021	103	14	R.TWNDPSVQQDIK.F	W2(+31.986503375)
31.9899	0.9376182	P07900	162	14	K.HNDDEQYAWESSAGGSFTVR.T	W9(+31.982136091)
31.9899	0.9376182	O43707	400	14	K.GYEEWLLNEIR.R	W5(+31.987450733)
31.9899	0.9376182	P00918	123	14	K.YAAELHLVHWNTK.Y	W10(+31.986263085)
31.9899	0.9376182	Q03135	85	14	K.IDFEDVIAEPEGTHSFDGIWK.A	W20(+31.985454634)

4.9792	0.6640700	P01857	196	13	R.VVSVLTVLHQDWLNGK.E	W12(+4.972542861)
15.9949	0.9191695	P18669	16	13	R.HGESAWNLENR.F	W6(+15.990421165)
15.9949	0.9191695	P07737	32	13	K.DSPSVWAAVPGK.T	W6(+15.986549636)
15.9949	0.9191695	P11021	103	13	R.TWNDPSVQQDIK.F	W2(+15.990653766)
15.9949	0.9191695	P07437	101	13	R.SGPFGQIFRPDNFVFGQSGAGNNWAK.G	W24(+15.989302919)
15.9949	0.9191695	A8K7I4	158	13	R.AFVHEWAHLR.W	W6(+15.992900282)
209.0179	0.7899121	P69892	16	13	M.GHFTEEDKATITSLWGK.V	W15(+209.014476134)
14.9827	0.2716954	P68871	38	13	R.LLVVYPWTQR.F	W7(+14.971621683)
3.9953	0.9130741	Q16555	412	12	R.IAVGSDADLVIWDPDSVK.T	W12(+3.986633417)
3.9953	0.9130741	O75947	54	12	R.LAALPENPPAIDWAYYK.A	W13(+3.988752843)
3.9953	0.9130741	P21333	345	12	R.TFSVWYVPEVTGTHK.V	W5(+3.995014381)
3.9953	0.9130741	Q01995	55	12	R.LGFQVWLK.N	W6(+3.994466482)
15.9949	0.9191695	Q13885	101	12	R.SGPFGQIFRPDNFVFGQSGAGNNWAK.G	W24(+15.983260439)
15.9949	0.9191695	P51911	82	12	K.INESTQNWHQLENIGNFIK.A	W8(+15.988267621)
15.9949	0.9191695	P62736	88	12	K.IWHHSFYNELR.V	W2(+15.993712055)
15.9949	0.9191695	P0DML2	112	12	R.ISLLLIESWLEPVR.F	W9(+15.992065525)
31.9899	0.9376182	P68032	81	12	K.YPIEHGIITNWDDMEK.I	W11(+31.984005067)
31.9899	0.9376182	P40925	184	12	K.NVIIWGNHSSTQYPDVNHAK.V	W5(+31.98678538)
31.9899	0.9376182	P21333	345	12	R.TFSVWYVPEVTGTHK.V	W5(+31.988910866)
31.9899	0.9376182	Q14195	291	12	K.GNVVFGEPITASLGIDGTHYWSK.N	W21(+31.986077389)
31.9899	0.9376182	Q05707	644	12	R.VTWHPLSADEGLHK.L	W3(+31.986925643)
3.9953	0.9130741	Q13813	1640	11	R.LAALADQWQFLVQK.S	W8(+3.989537159)
3.9953	0.9130741	P30041	82	11	K.LIALSIDSVEDHLAWSK.D	W15(+3.992014531)
3.9953	0.9130741	P21796	210	11	K.KLETAVNLAWTAGNSNTR.F	W10(+3.988469569)
3.9953	0.9130741	P00915	124	11	K.YSAELHVAHWNSAK.Y	W10(+3.991091382)
3.9953	0.9130741	P01859	192	11	R.VVSVLTVVHQDWLNGK.E	W12(+3.990891758)
3.9953	0.9130741	P51911	82	11	K.INESTQNWHQLENIGNFIK.A	W8(+3.993882855)
15.9949	0.9191695	P21333	2262	11	R.AEAGVPAEFSIWTR.E	W12(+15.984945071)
31.9899	0.9376182	Q13885	101	11	R.SGPFGQIFRPDNFVFGQSGAGNNWAK.G	W24(+31.96598749)
31.9899	0.9376182	P07437	101	11	R.SGPFGQIFRPDNFVFGQSGAGNNWAK.G	W24(+31.978524209)
31.9899	0.9376182	P21796	75	11	K.WNTDNTLGTEITVEDQLAR.G	W1(+31.97963037)
31.9899	0.9376182	O43707	308	11	K.LASDLLEWIR.R	W8(+31.986149807)
31.9899	0.9376182	O43707	192	11	K.NVNVQNFHISWK.D	W11(+31.988663218)
31.9899	0.9376182	Q99715	2045	11	R.VFGETTNSLSVAWDHADGPVQQYR.I	W13(+31.991651997)
31.9899	0.9376182	P07197	291	11	R.SQLESHSDQNMHQAEEWFK.C	W17(+31.985605626)
31.9899	0.9376182	P62736	88	11	K.IWHHSFYNELR.V	W2(+31.988724404)
31.9899	0.9376182	Q05707	210	11	R.IEWHLNAFSTK.D	W3(+31.989565426)
31.9899	0.9376182	A8K7I4	158	11	R.AFVHEWAHLR.W	W6(+31.987895399)
31.9899	0.9376182	Q9BVA1	344	11	K.NSSYFVEWIPNNVK.T	W8(+31.992783524)
3.9953	0.9130741	P01857	160	10	K.FNWYVDGVEVHNAK.T	W3(+3.99041082)
3.9953	0.9130741	P01857	264	10	K.GFYPSDIAVEWESNGQPENNYK.T	W11(+3.98756997)
3.9953	0.9130741	P0DML2	112	10	R.ISLLLIESWLEPVR.F	W9(+3.990404289)
15.9949	0.9191695	P68371	344	10	K.NSSYFVEWIPNNVK.T	W8(+15.984604813)
15.9949	0.9191695	P09382	69	10	K.DGGAWGTEQR.E	W5(+15.993723506)
15.9949	0.9191695	P07437	344	10	K.NSSYFVEWIPNNVK.T	W8(+15.991806962)
31.9899	0.9376182	P68363	407	10	R.AFVHWYVGEGMEEGEFSEAR.E	W5(+31.973413885)
31.9899	0.9376182	P07737	32	10	K.DSPSVWAAVPGK.T	W6(+31.983375808)
31.9899	0.9376182	P12814	173	10	K.NVNIQNFHISWK.D	W11(+31.989322827)
31.9899	0.9376182	P51911	82	10	K.INESTQNWHQLENIGNFIK.A	W8(+31.987643983)
31.9899	0.9376182	P62753	45	10	R.MATEVAADALGEEWK.G	W14(+31.988131534)
31.9899	0.9376182	P63267	87	10	K.IWHHSFYNELR.V	W2(+31.98760854)
31.9899	0.9376182	P31327	152	10	K.SLGQWLQEEKVPAIYGVDTR.M	W5(+31.987527186)
31.9899	0.9376182	P09382	69	10	K.DGGAWGTEQR.E	W5(+31.987864131)
31.9899	0.9376182	P15144	961	10	K.EVVLQWFTENSK.-	W6(+31.989897442)
-59.0506	0.1389893	P69892	16	9	M.GHFTEEDKATITSLWGKVNVEDAGGETLGR.L	W15(-59.050647383)
3.9953	0.9130741	P13611	287	9	R.LATVGELQAAWR.N	W11(+3.992252164)
3.9953	0.9130741	P68371	344	9	K.NSSYFVEWIPNNVK.T	W8(+3.990097978)
3.9953	0.9130741	P40925	184	9	K.NVIIWGNHSSTQYPDVNHAK.V	W5(+3.992888895)
3.9953	0.9130741	P02647	74	9	K.LLDNWDSVTSTFSK.L	W5(+3.994381911)
3.9953	0.9130741	P68032	88	9	K.IWHHTFYNELR.V	W2(+3.990866088)
3.9953	0.9130741	P35749	591	9	K.VDYNASAWLTK.N	W8(+3.99563827)
15.9949	0.9191695	P40925	184	9	K.NVIIWGNHSSTQYPDVNHAK.V	W5(+15.992791337)
15.9949	0.9191695	P27797	219	9	K.IKDPDASKPEDWDER.A	W12(+15.992792374)
15.9949	0.9191695	P68032	88	9	K.IWHHTFYNELR.V	W2(+15.991140746)
15.9949	0.9191695	P69905	15	9	K.AAWGKVGAHAGEYGAEALER.M	W3(+15.99097352)
15.9949	0.9191695	P13796	524	9	K.VNDDIIVNWVNETLR.E	W9(+15.994138124)
31.9899	0.9376182	P17174	296	9	R.ITWSNPPAQGAR.I	W3(+31.984213252)
31.9899	0.9376182	Q01082	1779	9	R.VDTVNHLADELINSGHSDAATIAEWK.D	W25(+31.985843338)
31.9899	0.9376182	P35609	724	9	R.VGWELLLTTIAR.T	W3(+31.987816349)

31.9899	0.9376182	P02679	253	9	K.EGFGHLSPTGTTEFWLGNEK.I	W15(+31.987919329)
31.9899	0.9376182	P02545	514	9	K.AGQVVTIWAAGAGATHSPPTDLVWK.A	W24(+31.987257142)
31.9899	0.9376182	P07195	325	9	K.SADTLWDIQK.D	W6(+31.987422396)
31.9899	0.9376182	P61586	58	9	K.QVELALWDTAGQEDYDR.L	W7(+31.98781086)
31.9899	0.9376182	P27695	119	9	K.LPAELQELPGLSHQYWSAPSDK.E	W16(+31.987231453)
31.9899	0.9376182	P18669	25	9	R.FSGWYDADLSPAGHEEAKR.G	W4(+31.98585418)
31.9899	0.9376182	P12109	696	9	K.SLQWMAGGTFTGEALQYTR.D	W4(+31.988973141)
31.9899	0.9376182	P12814	381	9	K.GYEEWLLNEIR.R	W5(+31.987450733)
31.9899	0.9376182	P06733	365	9	K.LAQANGWGVMVSHR.S	W7(+31.985566367)
31.9899	0.9376182	P02545	498	9	K.AGQVVTIWAAGAGATHSPPTDLVWK.A	W8(+31.991468568)
31.9899	0.9376182	O75083	584	9	R.LHHVSSLAWLDEHTLVTTSHDASVK.E	W9(+31.985997544)
3.9953	0.9130741	P35749	30	8	K.NFINSPVAQADWAAK.R	W12(+3.992108027)
3.9953	0.9130741	O14880	93	8	R.IASGLGLAWIVGR.V	W9(+3.99229064)
3.9953	0.9130741	P09972	314	8	R.ALQASALNAWR.G	W10(+3.992534957)
3.9953	0.9130741	P27695	119	8	K.LPAELQELPGLSHQYWSAPSDK.E	W16(+3.991503914)
3.9953	0.9130741	Q9BVA1	21	8	K.FWEVISDEHGIDPTGSYHGDSDLQLER.I	W2(+3.991350085)
3.9953	0.9130741	P62736	88	8	K.IWHHSFYNELR.V	W2(+3.993454628)
3.9953	0.9130741	P11021	604	8	K.IEWLESHQDADIEDFK.A	W3(+3.994794455)
3.9953	0.9130741	P18669	25	8	R.FSGWYDADLSPAGHEEAK.R	W4(+3.991628294)
3.9953	0.9130741	P30041	33	8	R.FHDFLGDSWGILFSHPR.D	W9(+3.992430636)
3.9953	0.9130741	P07900	162	8	K.HNDDEQYAWESSAGGSFTVR.T	W9(+3.993610701)
15.9949	0.9191695	Q16555	412	8	R.IAVGSDADLVIWDPDSVK.T	W12(+15.983581659)
15.9949	0.9191695	P01857	264	8	K.GFYPSDIAVEWESNGQPENNYK.T	W11(+15.989767236)
15.9949	0.9191695	P0DOY3	79	8	K.YAASSYLSLTPEQWK.S	W14(+15.994124805)
31.9899	0.9376182	P55072	454	8	R.WALSQSNPSALR.E	W1(+31.979238736)
31.9899	0.9376182	Q9Y277	210	8	K.IETSINLAWTAGSNNTR.F	W9(+31.984366924)
31.9899	0.9376182	P53999	110	8	K.GISLNPEQWSQLK.E	W9(+31.987239342)
31.9899	0.9376182	P14866	283	8	K.NDQDTWDYTNPNLSGQGDPGSNPNKR.Q	W6(+31.989913335)
31.9899	0.9376182	P55072	476	8	R.ETVVEVPQVTWEDIGGLEDVKR.E	W11(+31.986542701)
31.9899	0.9376182	Q14194	412	8	R.IAVGSDADVVIWDPDK.L	W12(+31.98260862)
31.9899	0.9376182	P22314	647	8	K.NFPNAIEHTLQWAR.D	W12(+31.988993506)
31.9899	0.9376182	P21980	404	8	K.YDAPFVFAEVNADVVDWIQQDDGSVHK.S	W17(+31.987063187)
31.9899	0.9376182	P21333	582	8	R.AWGPGLEGGVVGK.S	W2(+31.986043086)
31.9899	0.9376182	Q01082	510	8	R.LWEYLLELLR.A	W2(+31.989295321)
31.9899	0.9376182	Q01995	55	8	R.LGFQVWLK.N	W6(+31.989278494)
31.9899	0.9376182	P00915	98	8	R.LFQFHFHWGSTNEHGSEHTVDGVK.Y	W8(+31.982243172)
31.9899	0.9376182	P98160	991	8	R.LLSGPYFWSLPSR.F	W8(+31.988838627)
14.9827	0.2716954	P14136	256	8	R.TQYEAMASSNMHEAEEWYR.S	W17(+14.971978519)
14.9827	0.2716954	P07900	297	8	K.YIDQEELNKTKPIWTRNPDDITNEEYGEFYK.S	W14(+14.979184431)
14.9827	0.2716954	P30086	8	8	K.WSGPLSLQEVDEQPQHPLHVTYAGAAVDELGK.V	W1(+14.978751804)
3.9953	0.9130741	P05386	43	7	K.AAGVNVEPFWPGLFAK.A	W10(+3.991246143)
3.9953	0.9130741	P07737	32	7	K.DSPSVWAAVPGK.T	W6(+3.990089675)
3.9953	0.9130741	P01876	162	7	R.DASGVTFTWTPSSGK.S	W9(+3.992923543)
3.9953	0.9130741	Q99715	2045	7	R.VFGETTNSLSVAWDHADGPVQQYR.I	W13(+3.990614399)
3.9953	0.9130741	P00338	227	7	K.TLHPDLGTDKDKEQWK.E	W15(+3.98769467)
3.9953	0.9130741	P09543	167	7	K.NQWQLSADDLKK.L	W3(+3.992977518)
3.9953	0.9130741	P31327	152	7	K.SLGQWLQEEKVPAIYGVDTR.M	W5(+3.996194178)
3.9953	0.9130741	P15144	961	7	K.EVVLQWFTENSK.-	W6(+3.991118145)
3.9953	0.9130741	P07900	320	7	K.SLTNDWEDHLAVK.H	W6(+3.992821555)
3.9953	0.9130741	P12883	816	7	R.DSLLVIQWNIR.A	W8(+3.991763012)
3.9953	0.9130741	P07437	344	7	K.NSSYFVEWIPNNVK.T	W8(+3.992783524)
15.9949	0.9191695	P09543	167	7	K.NQWQLSADDLK.K	W3(+15.988527553)
15.9949	0.9191695	P04406	87	7	K.WGDAGAEYVVESTGVFTTMEK.A	W1(+15.992818318)
15.9949	0.9191695	P05093	121	7	K.GIAFADSGAHWQLHR.R	W11(+15.99595918)
15.9949	0.9191695	P02751	2014	7	R.FLATTPNSLLVSWQPPR.A	W13(+15.991472633)
15.9949	0.9191695	P51911	37	7	R.EQELREWIEGVTGR.R	W7(+15.99418295)
15.9949	0.9191695	P04792	16	7	R.GPSWDPFRDWYPHSR.L	W4(+15.991537568)
31.9899	0.9376182	P68371	101	7	R.SGPFGQIFRPDNFVFGQSGAGNNWAK.G	W24(+31.959578798)
31.9899	0.9376182	Q01082	726	7	R.EQWANLEQLSAIR.K	W3(+31.984320591)
31.9899	0.9376182	Q13885	344	7	K.NSSYFVEWIPNNVK.T	W8(+31.97947786)
31.9899	0.9376182	P05386	43	7	K.AAGVNVEPFWPGLFAK.A	W10(+31.985630909)
31.9899	0.9376182	Q9C002	69	7	K.LITINQQWKPIEELQNVQR.V	W8(+31.986509673)
31.9899	0.9376182	P21796	210	7	K.KLETAVNLAWTAGNSNTR.F	W10(+31.985404525)
31.9899	0.9376182	P62942	60	7	R.GWEEGVAQMSVGQR.A	W2(+31.98610504)
31.9899	0.9376182	P12814	289	7	K.LASDLLEWIR.R	W8(+31.984807034)
31.9899	0.9376182	P35749	30	7	K.NFINSPVAQADWAAK.R	W12(+31.984173456)
31.9899	0.9376182	O75947	54	7	R.LAALPENPPAIDWAYYK.A	W13(+31.985420421)
31.9899	0.9376182	P02751	1195	7	K.VVTPLSPPTNLHLEANPDTGVLTVSWER.S	W26(+31.985325874)
31.9899	0.9376182	Q13813	693	7	R.NVEDIELWLYEVEGHLASDDYGK.D	W8(+31.984581869)

31.9899	0.9376182	P98160	1989	7	R.AAGVPSATITWRKEGGSLPPQAR.S	W11(+31.991703295)
31.9899	0.9376182	O75083	242	7	K.AHDGGIYAISWSPDSTHLLSASGDK.T	W11(+31.988206581)
31.9899	0.9376182	P02763	140	7	K.NWGLSVYADKPETTK.E	W2(+31.987523292)
31.9899	0.9376182	P05093	220	7	K.DSLVDLVPWLK.I	W9(+31.989949005)
31.9899	0.9376182	P21980	40	7	R.GQPFWLTLHFEGR.N	W5(+31.987123181)
31.9899	0.9376182	P35609	416	7	K.ASTHETWAYGK.E	W7(+31.988745739)
31.9899	0.9376182	P00352	29	7	K.IFINNEWHDSVSGK.K	W7(+31.990247175)
3.9953	0.9130741	O43707	736	6	R.VGWEQLLTTIAR.T	W3(+3.992428794)
3.9953	0.9130741	Q00610	1556	6	K.DTELAEELLQWFLQEEK.R	W11(+3.997293344)
3.9953	0.9130741	O75390	438	6	R.ALGVLAQLIWSR.A	W10(+3.992265576)
3.9953	0.9130741	P27797	219	6	K.IKDPDASKPEDWDER.A	W12(+3.987665421)
3.9953	0.9130741	P12036	50	6	R.SAAGSSSGFHSWTR.T	W12(+3.988641128)
3.9953	0.9130741	P06396	642	6	R.AQPVQVAEGSEPDGFWEALGGK.A	W16(+3.992317129)
3.9953	0.9130741	P14136	256	6	R.TQYEAMASSNMHEAEEWYR.S	W17(+3.994975486)
3.9953	0.9130741	P45379	247	6	K.ELWQSIYNLEAEKFDLQEK.F	W3(+3.996113394)
3.9953	0.9130741	P00387	246	6	R.APEAWDYGQGFVNEEMIR.D	W5(+3.992337436)
3.9953	0.9130741	P35609	296	6	R.LASELLEWIR.R	W8(+3.989298571)
3.9953	0.9130741	P02545	498	6	K.AGQVVTIWAAGAGATHSPPTDLVWK.A	W8(+3.997388978)
4.9792	0.6640700	P01857	264	6	K.GFYPSDIAVEWESNGQPENNYK.T	W11(+4.973945844)
15.9949	0.9191695	P30041	82	6	K.LIALSIDSVEDHLAWSK.D	W15(+15.982309941)
15.9949	0.9191695	P12814	717	6	R.VGWEQLLTTIAR.T	W3(+15.989621177)
15.9949	0.9191695	O14880	93	6	R.IASGLGLAWIVGR.V	W9(+15.991968233)
15.9949	0.9191695	P07437	21	6	K.FWEVISDEHGIDPTGTYHGDSDLQLDR.I	W2(+15.98915282)
15.9949	0.9191695	P17600	126	6	R.VLLVIDEPHTDWAK.Y	W12(+15.991001381)
15.9949	0.9191695	P21333	582	6	R.AWGPGLEGGVVGK.S	W2(+15.990803828)
15.9949	0.9191695	P27824	287	6	R.KPEDWDERPK.I	W5(+15.993250095)
15.9949	0.9191695	P30086	122	6	R.YVWLVYEQDRPLK.C	W3(+15.993865051)
15.9949	0.9191695	P50440	149	6	R.RPDPIDWSLK.Y	W7(+15.994099727)
15.9949	0.9191695	P04406	196	6	K.TVDGPSGKLWR.D	W10(+15.991452213)
15.9949	0.9191695	P09972	314	6	R.ALQASALNAWR.G	W10(+15.992901168)
15.9949	0.9191695	P26439	230	6	K.FSTVNPVYVGNVAWAHILALR.A	W14(+15.992254046)
15.9949	0.9191695	P11021	604	6	K.IEWLESHQDADIEDFK.A	W3(+15.988982829)
31.9899	0.9376182	P13611	287	6	R.LATVGELQAAWR.N	W11(+31.987809884)
31.9899	0.9376182	P17174	320	6	R.IVASTLSNPELFEEWTGNVK.T	W15(+31.97792371)
31.9899	0.9376182	P13637	896	6	R.TVNDLEDSYGQQWTYEQR.K	W13(+31.985548426)
31.9899	0.9376182	Q01082	1787	6	K.DGLNEAWADLLELIDTR.T	W7(+31.984716293)
31.9899	0.9376182	P02671	295	6	R.NPSSAGSWNSGSSGPGSTGNR.N	W8(+31.987466213)
31.9899	0.9376182	P00367	129	6	R.DDGSWEVIEGYR.A	W5(+31.984130366)
31.9899	0.9376182	Q13813	1106	6	R.EANELQQWINEK.E	W8(+31.983691345)
31.9899	0.9376182	Q16698	322	6	K.EQWDTIEELIR.K	W3(+31.988563222)
31.9899	0.9376182	P04179	210	6	K.AIWNVINWENVTER.Y	W8(+31.987160958)
31.9899	0.9376182	P02763	43	6	K.WFYIASAFR.N	W1(+31.988332328)
31.9899	0.9376182	P06744	380	6	R.VDHQTGPIVWGEPGTNGQHAFYQLIHQGTK.M	W10(+31.961077582)
31.9899	0.9376182	P07686	424	6	K.LAPGTIVEVWKDSAYPEELSR.V	W10(+31.989722958)
31.9899	0.9376182	P15121	112	6	K.TLSDLKLDYLDLYLIHWPTGFKPGK.E	W17(+31.993218196)
31.9899	0.9376182	P06396	642	6	R.AQPVQVAEGSEPDGFWEALGGK.A	W16(+31.987678457)
31.9899	0.9376182	Q14896	711	6	K.AITQGNKAPARPAPDAPEDTGDSDEWVFDKK.L	W26(+31.980837059)
31.9899	0.9376182	P29972	245	6	K.VWTSGQVEEYDLDADDINSR.V	W2(+31.985811725)
31.9899	0.9376182	Q13509	101	6	R.SGAFGHLFRPDNFIFGQSGAGNNWAK.G	W24(+31.985062318)
31.9899	0.9376182	P31146	379	6	R.KSDLFQEDLYPPTAGPDPALTAEEWLGGR.D	W25(+31.988149104)
31.9899	0.9376182	P02751	1378	6	R.VTWAPPPSIDLTNFLVR.Y	W3(+31.985012622)
31.9899	0.9376182	Q99715	2136	6	R.VSWDPSPSPVLGYK.I	W3(+31.986917626)
31.9899	0.9376182	Q8WZ42	28919	6	R.VTWFKDGVEIEKR.M	W3(+31.988908788)
31.9899	0.9376182	Q1KMD3	318	6	R.VGWSVDFSRPQLGEDEFSYGFDGR.G	W3(+31.990180357)
31.9899	0.9376182	P45379	247	6	K.ELWQSIYNLEAEKFDLQEK.F	W3(+31.994295626)
31.9899	0.9376182	P61106	65	6	K.LQIWDTAGQER.F	W4(+31.98536052)
31.9899	0.9376182	P02787	479	6	R.TAGWNIPMGLLYNK.I	W4(+31.988443212)
31.9899	0.9376182	P00387	246	6	R.APEAWDYGQGFVNEEMIR.D	W5(+31.987942905)
31.9899	0.9376182	P29762	88	6	R.SLATWENENK.I	W5(+31.989105155)
31.9899	0.9376182	O75083	110	6	K.IKDIAWTEDSKR.I	W6(+31.985799563)
31.9899	0.9376182	P31930	429	6	R.RIPLAEWESR.I	W7(+31.984142472)
31.9899	0.9376182	P02790	393	6	K.SGAQATWTELPWPHEK.V	W7(+31.989005868)
31.9899	0.9376182	P07384	392	6	R.NYPATFWVNPQFK.I	W7(+31.989641829)
31.9899	0.9376182	P13639	641	6	R.YLAEKYEWDVAEAR.K	W8(+31.988036638)
31.9899	0.9376182	P61106	105	6	R.STYNHLSSWLTDAR.N	W9(+31.988988467)
88.9942	0.1672632	P69892	131	6	K.EFTPEVQASWQK.M	W10(+88.991681619)
14.9827	0.2716954	P68371	344	6	K.NSSYFVEWIPNNVK.T	W8(+14.970566728)
14.9827	0.2716954	P14618	482	6	K.DPVQEAWAEDVDLR.V	W7(+14.973324812)
-124.1118	0.2696443	O43707	308	5	K.LASDLLEWIRR.T	W8(-124.110200478)

3.9953	0.9130741	P60709	86	5	K.IWHHTFYNELR.V	W2(+3.988193827)
3.9953	0.9130741	P12814	717	5	R.VGWEQLLTTIAR.T	W3(+3.989499107)
3.9953	0.9130741	P12814	289	5	K.LASDLLEWIR.R	W8(+3.993474026)
3.9953	0.9130741	P68032	81	5	K.YPIEHGIITNWDDMEK.I	W11(+3.992427919)
3.9953	0.9130741	P02511	60	5	R.APSWFDTGLSEMR.L	W4(+3.989668923)
3.9953	0.9130741	P00918	123	5	K.YAAELHLVHWNTK.Y	W10(+3.991267968)
3.9953	0.9130741	P37802	150	5	R.DDGLFSGDPNWFPK.K	W11(+3.99373589)
3.9953	0.9130741	O75083	242	5	K.AHDGGIYAISWSPDSTHLLSASGDK.T	W11(+3.99417474)
3.9953	0.9130741	P62826	163	5	K.SNYNFEKPFLWLAR.K	W11(+3.994552978)
3.9953	0.9130741	P02647	96	5	K.LREQLGPVTQEFWDNLEK.E	W13(+3.993929383)
3.9953	0.9130741	P98160	1989	5	R.AAGVPSATITWRKEGGSLPPQAR.S	W11(+3.9958404)
3.9953	0.9130741	P13796	387	5	R.YPALHKPENQDIDWGALEGETR.E	W14(+3.991793799)
3.9953	0.9130741	P09960	302	5	K.SLSNVIAHEISHSWTGNLVTNK.T	W14(+3.993383369)
3.9953	0.9130741	P06733	304	5	K.DYPVVSIEDPFDQDDWGAWQK.F	W19(+3.997189302)
3.9953	0.9130741	P07437	21	5	K.FWEVISDEHGIDPTGTYHGDSDLQLDR.I	W2(+3.996843249)
3.9953	0.9130741	P35609	724	5	R.VGWELLLTTIAR.T	W3(+3.992577091)
3.9953	0.9130741	Q05707	644	5	R.VTWHPLSADEGLHK.L	W3(+3.993124656)
3.9953	0.9130741	P62820	65	5	K.LQIWDTAGQER.F	W4(+3.99305095)
3.9953	0.9130741	P07195	325	5	K.SADTLWDIQK.D	W6(+3.993770053)
3.9953	0.9130741	Q8WX93	1329	5	R.LDVYTQWHQQSQSTKPK.K	W7(+3.992820145)
3.9953	0.9130741	P02787	460	5	K.SASDLTWDNLK.G	W7(+3.99435948)
3.9953	0.9130741	Q9NR12	14	5	K.VVLEGPAPWGFR.L	W9(+3.99403133)
15.9949	0.9191695	P12277	228	5	K.TFLVWVNEEDHLR.V	W5(+15.986101517)
15.9949	0.9191695	P07900	320	5	K.SLTNDWEDHLAVK.H	W6(+15.987938742)
15.9949	0.9191695	P07195	325	5	K.SADTLWDIQK.D	W6(+15.992915561)
15.9949	0.9191695	Q9UQM7	237	5	K.AGAYDFPSPEWDTVTPEAK.D	W11(+15.989916945)
15.9949	0.9191695	Q13885	344	5	K.NSSYFVEWIPNNVK.T	W8(+15.991440751)
15.9949	0.9191695	Q12860	845	5	R.YWAAHDKEEAANR.V	W2(+15.988952798)
15.9949	0.9191695	P06733	301	5	K.DYPVVSIEDPFDQDDWGAWQK.F	W16(+15.993296339)
15.9949	0.9191695	P05413	98	5	K.LVHLQKWDGQETTLVR.E	W7(+15.99460814)
15.9949	0.9191695	P07237	396	5	K.NVFVEFYAPWCGHCK.Q	W10(+15.994885796)
15.9949	0.9191695	P15814	186	5	K.YAASSYLSLTPEQWR.S	W14(+15.9826057)
15.9949	0.9191695	P04792	51	5	R.LPEEWSQWLGGSSWPGYVRPLPPAAIESPAVAAPAYSR.A	W14(+15.988065577)
15.9949	0.9191695	P06733	304	5	K.DYPVVSIEDPFDQDDWGAWQK.F	W19(+15.994272901)
15.9949	0.9191695	P08238	312	5	K.SLTNDWEDHLAVK.H	W6(+15.992943625)
15.9949	0.9191695	P04792	45	5	R.LPEEWSQWLGGSSWPGYVRPLPPAAIESPAVAAPAYSR.A	W8(+15.987333155)
15.9949	0.9191695	P05141	71	5	K.EQGVLSFWR.G	W8(+15.993854696)
31.9899	0.9376182	P27797	33	5	K.EQFLDGDGWTSR.W	W9(+31.987213984)
31.9899	0.9376182	P21579	260	5	K.VPMNTVDFGHVTEEWR.D	W15(+31.981395018)
31.9899	0.9376182	Q01082	1713	5	R.EVDDLEQWIAER.E	W8(+31.987468729)
31.9899	0.9376182	P35579	26	5	K.NFINNPLAQADWAAK.K	W12(+31.987409511)
31.9899	0.9376182	P02751	2014	5	R.FLATTPNSLLVSWQPPR.A	W13(+31.988872633)
31.9899	0.9376182	O75083	133	5	K.FGAVFLWDSGSSVGEITGHNK.V	W7(+31.985520689)
31.9899	0.9376182	P05141	71	5	K.EQGVLSFWR.G	W8(+31.986042196)
31.9899	0.9376182	P04004	200	5	R.DVWGIEGPIDAAFTR.I	W3(+31.987315425)
31.9899	0.9376182	P60174	195	5	K.VIADNVKDWSK.V	W9(+31.985665667)
31.9899	0.9376182	P13861	230	5	R.AATIVATSEGSLWGLDR.V	W13(+31.987850311)
31.9899	0.9376182	P08237	132	5	R.SEWSDLLSDLQK.A	W3(+31.98635916)
31.9899	0.9376182	P37837	147	5	K.LSSTWEGIQAGK.E	W5(+31.98748021)
31.9899	0.9376182	Q01082	2032	5	R.DASVAEAWLLGQEPYLSSR.E	W8(+31.989168638)
31.9899	0.9376182	P13639	685	5	K.DSVVAGFQWATK.E	W9(+31.988254207)
31.9899	0.9376182	Q15124	364	5	K.VPVYETPAGWR.F	W10(+31.988850703)
31.9899	0.9376182	P14625	485	5	K.IADDKYNDTFWKEFGTNIK.L	W11(+31.985579678)
31.9899	0.9376182	P29401	86	5	K.GHAAPILYAVWAEAGFLAEAELLNLR.K	W11(+31.999243928)
31.9899	0.9376182	P12036	50	5	R.SAAGSSSGFHSWTR.T	W12(+31.98925148)
31.9899	0.9376182	P0C0L5	116	5	R.GPEVQLVAHSPWLK.D	W12(+31.990495133)
31.9899	0.9376182	P27824	287	5	R.EIEDPEDRKPEDWDERPK.I	W13(+31.987555794)
31.9899	0.9376182	Q05707	50	5	R.YNVISHDSIQISWK.A	W13(+31.988132758)
31.9899	0.9376182	Q99613	121	5	R.ILADLEDYLNELWEDKEGK.K	W13(+31.989782815)
31.9899	0.9376182	P13796	387	5	R.YPALHKPENQDIDWGALEGETR.E	W14(+31.983309913)
31.9899	0.9376182	P09960	302	5	K.SLSNVIAHEISHSWTGNLVTNK.T	W14(+31.990575751)
31.9899	0.9376182	P0DMV9	90	5	K.HWPFQVINDGDKPK.V	W2(+31.984813288)
31.9899	0.9376182	P04004	339	5	R.DWHGVPGQVDAAMAGR.I	W2(+31.987171277)
31.9899	0.9376182	Q05707	755	5	R.VKWDISDSDVQQFR.V	W3(+31.985794877)
31.9899	0.9376182	P62937	121	5	K.TEWLDGKHVVFGK.V	W3(+31.989641829)
31.9899	0.9376182	Q01082	1574	5	K.QLWGLLIEETEKR.H	W3(+31.989745898)
31.9899	0.9376182	P69905	15	5	K.AAWGKVGAHAGEYGAEALER.M	W3(+31.992011118)
31.9899	0.9376182	P26038	175	5	R.IQVWHEEHR.G	W4(+31.987448417)
31.9899	0.9376182	Q92522	79	5	K.KVPWFDQQNGR.T	W4(+31.990243638)

31.9899	0.9376182	P48735	164	5	R.LVPGWTKPITIGR.H	W5(+31.986273393)
31.9899	0.9376182	Q9HBL0	650	5	R.GLNSWQQQQQQQQQPRPPPR.Q	W5(+31.988159204)
31.9899	0.9376182	P35609	388	5	K.GYEEWLLNEIR.R	W5(+31.990380421)
31.9899	0.9376182	O15230	2939	5	K.STGDPWLTDGSYLDGTGFAR.I	W6(+31.986341603)
31.9899	0.9376182	O14521	66	5	K.AASLHWTSER.V	W6(+31.987867676)
31.9899	0.9376182	P17252	223	5	R.STLNPQWNESFTFK.L	W7(+31.985686935)
31.9899	0.9376182	P21333	749	5	K.HTAMVSWGGVSIPNSPFR.V	W7(+31.986620791)
31.9899	0.9376182	P50440	149	5	R.RPDPIDWSLK.Y	W7(+31.987996211)
31.9899	0.9376182	Q8WX93	1329	5	R.LDVYTQWHQQSQSTKPK.K	W7(+31.988364579)
31.9899	0.9376182	P34932	658	5	K.LEDTENWLYEDGEDQPK.Q	W7(+31.988580069)
31.9899	0.9376182	P84077	66	5	K.NISFTVWDVGGQDK.I	W7(+31.989866803)
31.9899	0.9376182	P04792	45	5	R.LPEEWSQWLGGSSWPGYVRPLPPAAIESPAVAAPAYSR.A	W8(+31.982694483)
31.9899	0.9376182	P11047	540	5	R.DGSEASLEWSSER.Q	W9(+31.988609533)
31.9899	0.9376182	P12883	1456	5	R.NFDKILAEWK.Q	W9(+31.988713284)
244.0876	0.2231023	P01860	207	5	K.WYVDGVEVHNAK.T	W1(+244.083441637)
32.9748	0.6928963	P06733	365	5	K.LAQANGWGVMVSHR.S	W7(+32.970233256)
32.9748	0.6928963	P06744	380	5	R.VDHQTGPIVWGEPGTNGQHAFYQLIHQGTK.M	W10(+32.968903368)
14.9827	0.2716954	P21796	75	5	K.WNTDNTLGTEITVEDQLAR.G	W1(+14.973526854)
14.9827	0.2716954	P09543	310	5	R.VELSEQQLQLWPSDVDK.L	W11(+14.97495597)
14.9827	0.2716954	P51911	82	5	K.INESTQNWHQLENIGNFIK.A	W8(+14.980808045)
3.9953	0.9130741	P12277	228	4	K.TFLVWVNEEDHLR.V	W5(+3.983049759)
3.9953	0.9130741	P68371	101	4	R.SGPFGQIFRPDNFVFGQSGAGNNWAK.G	W24(+3.986922548)
3.9953	0.9130741	P61019	100	4	R.DTFNHLTTWLEDAR.Q	W9(+3.996757012)
3.9953	0.9130741	P17174	320	4	R.IVASTLSNPELFEEWTGNVK.T	W15(+3.987689335)
3.9953	0.9130741	O43707	308	4	K.LASDLLEWIR.R	W8(+3.994572659)
3.9953	0.9130741	Q13813	1566	4	R.DVDEIEAWISEK.L	W8(+3.993510709)
3.9953	0.9130741	Q9UQM7	237	4	K.AGAYDFPSPEWDTVTPEAK.D	W11(+3.990649367)
3.9953	0.9130741	Q09666	4184	4	K.VDIDVPDVDVQGPDWHLK.M	W15(+3.993786658)
3.9953	0.9130741	P07686	424	4	K.LAPGTIVEVWKDSAYPEELSR.V	W10(+3.995643368)
3.9953	0.9130741	Q8WX93	1168	4	K.VSGLPTPDLSWQLDGKPVRPDSAHK.M	W11(+3.991608638)
3.9953	0.9130741	P55072	476	4	R.ETVVEVPQVTWEDIGGLEDVKR.E	W11(+3.992377564)
3.9953	0.9130741	Q14195	412	4	R.ISVGSDSDLVIWDPDAVK.I	W12(+3.992329147)
3.9953	0.9130741	P27824	287	4	R.EIEDPEDRKPEDWDERPK.I	W13(+3.99242532)
3.9953	0.9130741	P62753	45	4	R.MATEVAADALGEEWK.G	W14(+3.993624698)
3.9953	0.9130741	P0DMV9	90	4	K.HWPFQVINDGDKPK.V	W2(+3.992381648)
3.9953	0.9130741	Q13509	101	4	R.SGAFGHLFRPDNFIFGQSGAGNNWAK.G	W24(+3.998856264)
3.9953	0.9130741	P01009	218	4	K.GKWERPFEVK.D	W3(+3.993593177)
3.9953	0.9130741	P12109	696	4	K.SLQWMAGGTFTGEALQYTR.D	W4(+3.996785641)
3.9953	0.9130741	O75083	110	4	K.IKDIAWTEDSKR.I	W6(+3.99228652)
3.9953	0.9130741	Q8NBS9	227	4	K.ALAPTWEQLALGLEHSETVK.I	W6(+3.994584868)
3.9953	0.9130741	P00352	29	4	K.IFINNEWHDSVSGK.K	W7(+3.992139264)
3.9953	0.9130741	P31930	429	4	R.RIPLAEWESR.I	W7(+3.992931535)
3.9953	0.9130741	O43707	428	4	K.ASIHEAWTDGK.E	W7(+3.993872692)
3.9953	0.9130741	Q13907	144	4	K.AQSDGIWGEHEIDYILLVR.K	W7(+3.999422547)
3.9953	0.9130741	P02751	65	4	K.HYQINQQWER.T	W8(+3.993282456)
3.9953	0.9130741	P08670	290	4	K.NLQEAEEWYK.S	W8(+3.995336043)
3.9953	0.9130741	P13639	641	4	R.YLAEKYEWDVAEAR.K	W8(+3.995408605)
3.9953	0.9130741	Q9Y277	210	4	K.IETSINLAWTAGSNNTR.F	W9(+3.987540752)
3.9953	0.9130741	Q13228	204	4	R.HNVMISTEWAAPNVLR.D	W9(+3.992947796)
15.9949	0.9191695	P13591	656	4	R.ALSSEWKPEIR.L	W6(+15.989634143)
15.9949	0.9191695	P60880	105	4	K.AWGNNQDGVVASQPAR.V	W2(+15.986833998)
15.9949	0.9191695	P00367	129	4	R.DDGSWEVIEGYR.A	W5(+15.9897456)
15.9949	0.9191695	P46459	213	4	R.QSIINPDWNFEK.M	W8(+15.991377635)
15.9949	0.9191695	O75390	438	4	R.ALGVLAQLIWSR.A	W10(+15.99243145)
15.9949	0.9191695	P00738	398	4	K.VTSIQDWVQK.T	W7(+15.988355626)
15.9949	0.9191695	P35749	591	4	K.VDYNASAWLTK.N	W8(+15.994905848)
15.9949	0.9191695	P01859	192	4	R.VVSVLTVVHQDWLNGK.E	W12(+15.991855034)
15.9949	0.9191695	P63267	87	4	K.IWHHSFYNELR.V	W2(+15.995298969)
15.9949	0.9191695	P45379	247	4	K.ELWQSIYNLEAEKFDLQEK.F	W3(+16.001789664)
15.9949	0.9191695	P02766	61	4	K.AADDTWEPFASGK.T	W6(+15.995437189)
15.9949	0.9191695	Q00610	956	4	R.RKDPELWGSVLLESNPYR.R	W7(+15.994979381)
15.9949	0.9191695	Q8WZ42	18283	4	R.DVASAQWSPLSATSK.K	W7(+15.991875359)
15.9949	0.9191695	P62273	8	4	M.GHQQLYWSHPR.K	W7(+15.995118077)
15.9949	0.9191695	P02749	335	4	K.EHSSLAFWK.T	W8(+15.993304189)
19.9903	0.3691468	P63261	86	4	K.IWHHTFYNELR.V	W2(+19.98929246)
31.9899	0.9376182	Q13813	1566	4	R.DVDEIEAWISEK.L	W8(+31.97268049)
31.9899	0.9376182	P06733	301	4	K.DYPVVSIEDPFDQDDWGAWQK.F	W16(+31.976206495)
31.9899	0.9376182	Q9UQM7	237	4	K.AGAYDFPSPEWDTVTPEAK.D	W11(+31.986743117)
31.9899	0.9376182	P02511	60	4	R.APSWFDTGLSEMR.L	W4(+31.987593728)

31.9899	0.9376182	P07237	128	4	R.EADDIVNWLK.K	W8(+31.98654284)
31.9899	0.9376182	P12814	409	4	K.ASIHEAWTDGK.E	W7(+31.987647106)
31.9899	0.9376182	P25325	15	4	R.ALVSAQWVAEALR.A	W7(+31.988365695)
31.9899	0.9376182	Q15149	1359	4	R.ESADPLGAWLQDAR.R	W9(+31.984511058)
31.9899	0.9376182	P12110	696	4	K.NLEWIAGGTWTPSALK.F	W4(+31.988620168)
31.9899	0.9376182	Q00610	164	4	K.WLLLTGISAQQNR.V	W1(+31.987960291)
31.9899	0.9376182	Q01813	469	4	K.EIGWTDVGGWTGQGGSILGTK.R	W10(+31.991209053)
31.9899	0.9376182	Q9NR12	49	4	K.AAQAGVAVGDWVLSIDGENAGSLTHIEAQNK.I	W11(+32.00173199)
31.9899	0.9376182	Q13813	2294	4	K.YTEHSTVGLAQQWDQLDQLGMR.M	W13(+31.990671566)
31.9899	0.9376182	P27797	200	4	K.IDNSQVESGSLEDDWDFLPPKK.I	W15(+31.986081794)
31.9899	0.9376182	Q09666	3470	4	K.APEVNLNAPDVDVHGPDWNLK.M	W18(+31.983766908)
31.9899	0.9376182	P63244	90	4	R.LWDLTTGTTTR.R	W2(+31.986625718)
31.9899	0.9376182	P12109	86	4	R.NLVWNAGALHYSDEVEIIQGLTR.M	W4(+31.991406069)
31.9899	0.9376182	Q9Y6R7	5349	4	K.AGVQVWLGANGK.V	W6(+31.986441157)
31.9899	0.9376182	P21796	64	4	R.WTEYGLTFTEK.W	W1(+31.986969784)
31.9899	0.9376182	Q99798	657	4	R.WVVIGDENYGEGSSR.E	W1(+31.987963534)
31.9899	0.9376182	P05093	313	4	K.WTLAFLLHNPQVK.K	W1(+31.989393551)
31.9899	0.9376182	P05413	98	4	K.LVHLQKWDGQETTLVR.E	W7(+31.988504624)
31.9899	0.9376182	P04792	22	4	R.GPSWDPFRDWYPHSR.L	W10(+31.986715791)
31.9899	0.9376182	Q14697	939	4	R.KPGINVASDWSIHLR.-	W10(+31.989721168)
31.9899	0.9376182	P19835	71	4	K.ALENPQPHPGWQGTLK.A	W11(+31.985147989)
31.9899	0.9376182	P01624	55	4	R.ASQSVSSNLAWYQQKPGQAPR.L	W11(+31.986170615)
31.9899	0.9376182	Q13554	238	4	K.AGAYDFPSPEWDTVTPEAK.N	W11(+31.98796382)
31.9899	0.9376182	P00751	334	4	K.VSEADSSNADWVTK.Q	W11(+31.988020995)
31.9899	0.9376182	P17600	126	4	R.VLLVIDEPHTDWAK.Y	W12(+31.984165443)
31.9899	0.9376182	P35580	30	4	R.AVIYNPATQADWTAK.K	W12(+31.988999345)
31.9899	0.9376182	Q96EP5	206	4	K.SQAPGQPGASQWGSR.V	W12(+31.989431364)
31.9899	0.9376182	Q13813	2220	4	R.QEFAQHANAFHQWIQETR.T	W13(+31.985738368)
31.9899	0.9376182	Q9UL46	112	4	K.VLSLLALVKPEVWTLK.E	W13(+31.9913036)
31.9899	0.9376182	Q16658	171	4	R.PADEIAVDRDVPWGVDSLITLAFQDQR.Y	W13(+31.994747446)
31.9899	0.9376182	P11142	90	4	R.RFDDAVVQSDMKHWPFMVVNDAGRPK.V	W14(+31.988898925)
31.9899	0.9376182	P08237	459	4	K.GQIEEAGWSYVGGWTGQGGSK.L	W14(+31.989975729)
31.9899	0.9376182	O75083	349	4	K.SYIYSGSHDGHINYWDSETGENDSFAGK.G	W15(+31.984800459)
31.9899	0.9376182	Q09666	2239	4	K.VDIDAPDVDVHGPDWHLK.M	W15(+31.986958387)
31.9899	0.9376182	Q14896	683	4	R.LDVPISGDPAPTVIWQK.A	W15(+31.987983898)
31.9899	0.9376182	Q09666	1708	4	K.VDIDAPDVEVHDPDWHLK.M	W15(+31.991267389)
31.9899	0.9376182	Q01082	202	4	K.TAGYPNVNIHNFTTSWR.D	W16(+31.987432273)
31.9899	0.9376182	P15121	220	4	K.GIVVTAYSPLGSPDRPWAKPEDPSLLEDPR.I	W17(+31.990266517)
31.9899	0.9376182	P06744	228	4	K.TFTTQETITNAETAKEWFLQAAKDPSAVAK.H	W17(+31.998244671)
31.9899	0.9376182	P16435	219	4	R.IFELGLGDDDGNLEEDFITWR.E	W20(+31.976730195)
31.9899	0.9376182	P09211	39	4	R.MLLADQGQSWKEEVVTVETWQEGSLK.A	W20(+31.98995564)
31.9899	0.9376182	P23141	189	4	R.GNWGHLDQVAALR.W	W3(+31.985962502)
31.9899	0.9376182	A8K7I4	273	4	R.STWEVIRDSEDFKK.T	W3(+31.985982094)
31.9899	0.9376182	Q92747	221	4	R.LAWVSHDSTVSVADASK.S	W3(+31.987576373)
31.9899	0.9376182	P61160	89	4	K.HLWDYTFGPEK.L	W3(+31.988376278)
31.9899	0.9376182	P26038	43	4	R.EVWFFGLQYQDTK.G	W3(+31.989013473)
31.9899	0.9376182	Q06520	77	4	R.SPWVESEIGYTALSETESPR.L	W3(+31.991082835)
31.9899	0.9376182	P33241	230	4	K.IDQWLEQYTQAIETAGR.T	W4(+31.988797124)
31.9899	0.9376182	Q13263	351	4	R.FASWALESDNNTALLLSK.K	W4(+31.989224341)
31.9899	0.9376182	Q9GZM7	407	4	K.ITGWGEETLPDGR.T	W4(+31.989433063)
31.9899	0.9376182	P49821	75	4	K.GPDWILGEIK.T	W4(+31.991096876)
31.9899	0.9376182	P46783	69	4	R.HFYWYLTNEGIQYLR.D	W4(+31.993281874)
31.9899	0.9376182	Q8WZ42	21420	4	K.ATLTWTPPLEDGGSPIK.S	W5(+31.986379834)
31.9899	0.9376182	P26583	133	4	K.LGEMWSEQSAK.D	W5(+31.987906538)
31.9899	0.9376182	P10745	824	4	K.VTEVWTLPQVAGQR.Y	W5(+31.989169786)
31.9899	0.9376182	P34949	74	4	K.TLSQWIAENQDSLGSK.V	W5(+31.98962328)
31.9899	0.9376182	P07195	251	4	K.GYTNWAIGLSVADLIESMLK.N	W5(+31.992193515)
31.9899	0.9376182	Q9Y490	351	4	K.EVIQEWNLTNIK.R	W6(+31.987126987)
31.9899	0.9376182	Q6UW68	115	4	R.TTAAMWALQTVEK.E	W6(+31.987569826)
31.9899	0.9376182	Q9HBL0	79	4	K.VLEFGWPDLHTPALEK.I	W6(+31.987745914)
31.9899	0.9376182	Q9Y2J8	49	4	K.HSEHVWVEVVR.D	W6(+31.987929755)
31.9899	0.9376182	Q16643	85	4	K.YVLINWVGEDVPDAR.K	W6(+31.988237446)
31.9899	0.9376182	P55083	111	4	R.ADGEYWLGLQNMHLLTLK.Q	W6(+31.990625477)
31.9899	0.9376182	Q8WZ42	30582	4	K.STVTLAWEKPLYDGGSR.L	W7(+31.9874181)
31.9899	0.9376182	P02787	460	4	K.SASDLTWDNLK.G	W7(+31.989720809)
31.9899	0.9376182	P02671	360	4	R.PGSTGTWNPGSSER.G	W7(+31.990032537)
31.9899	0.9376182	Q9Y678	776	4	K.LNFEAAWDEVGDEFEKEETFTLSTIK.T	W7(+31.994179471)
31.9899	0.9376182	P35609	296	4	R.LASELLEWIR.R	W8(+31.988322009)
31.9899	0.9376182	P08238	157	4	K.HNDDEQYAWESSAGGSFTVR.A	W9(+31.982136091)

31.9899	0.9376182	Q9NX14	55	4	K.RPPEPTTPWQEDPEPEDENLYEK.N	W9(+31.985181041)
31.9899	0.9376182	P15311	445	4	R.RKEDEVEEWQHR.A	W9(+31.985354676)
31.9899	0.9376182	P13797	527	4	K.ANDDIIVNWVNR.T	W9(+31.985784545)
31.9899	0.9376182	Q9UMS6	1086	4	K.KSGVTIQVWKPSVVEE.-	W9(+31.987190993)
31.9899	0.9376182	P09543	192	4	K.DFLPLYFGWFLTK.K	W9(+31.989637357)
88.9942	0.1672632	P69892	38	4	R.LLVVYPWTQR.F	W7(+88.991275003)
368.1698	0.3290409	O43583	182	4	K.WPEVDDDSIEDLGEVKK.-	W1(+368.170097156)
14.9827	0.2716954	P30086	55	4	K.NRPTSISWDGLDSGK.L	W8(+14.971954054)
14.9827	0.2716954	P60174	206	4	K.VVLAYEPVWAIGTGK.T	W9(+14.972003529)
14.9827	0.2716954	Q01082	202	4	K.TAGYPNVNIHNFTTSWR.D	W16(+14.98024135)
-13.0308	0.3243439	P62820	105	3	K.QWLQEIDRYASENVNK.L	W2(-13.027051616)
3.9953	0.9130741	P68363	407	3	R.AFVHWYVGEGMEEGEFSEAR.E	W5(+3.989113207)
3.9953	0.9130741	P12236	71	3	K.EQGVLSFWR.G	W8(+3.992267782)
3.9953	0.9130741	P18669	16	3	R.HGESAWNLENR.F	W6(+3.991886009)
3.9953	0.9130741	P13591	656	3	R.ALSSEWKPEIR.L	W6(+3.993662464)
3.9953	0.9130741	P13637	896	3	R.TVNDLEDSYGQQWTYEQR.K	W13(+3.993849208)
3.9953	0.9130741	P0DOY2	79	3	K.YAASSYLSLTPEQWK.S	W14(+3.995711719)
3.9953	0.9130741	P37802	117	3	R.YGINTTDIFQTVDLWEGK.N	W15(+3.990922117)
3.9953	0.9130741	Q16698	322	3	K.EQWDTIEELIR.K	W3(+3.993323964)
3.9953	0.9130741	O43707	400	3	K.GYEEWLLNEIR.R	W5(+3.993065968)
3.9953	0.9130741	P61586	58	3	K.QVELALWDTAGQEDYDR.L	W7(+3.988823946)
3.9953	0.9130741	Q05707	755	3	K.WDISDSDVQQFR.V	W1(+3.99633978)
3.9953	0.9130741	P19835	391	3	K.TTFDVYTESWAQDPSQENKKK.T	W10(+3.992974655)
3.9953	0.9130741	P04746	149	3	R.DFPAVPYSGWDFNDGK.C	W10(+3.998285811)
3.9953	0.9130741	P63267	80	3	K.YPIEHGIITNWDDMEK.I	W11(+3.992305848)
3.9953	0.9130741	O43707	192	3	K.NVNVQNFHISWK.D	W11(+3.994596914)
3.9953	0.9130741	P43405	284	3	R.PQLPGSHPATWSAGGIISR.I	W11(+3.994770623)
3.9953	0.9130741	P17174	141	3	K.NTPVYVSSPTWENHNAVFSAAGFK.D	W11(+3.995227727)
3.9953	0.9130741	P02790	398	3	K.SGAQATWTELPWPHEK.V	W12(+3.991250882)
3.9953	0.9130741	P35579	26	3	K.NFINNPLAQADWAAK.K	W12(+3.993146816)
3.9953	0.9130741	P09093	40	3	R.VVHGEDAVPYSWPWQVSLQYEK.S	W12(+3.993995769)
3.9953	0.9130741	Q04760	171	3	K.GLAFIQDPDGYWIEILNPNK.M	W12(+3.998576899)
3.9953	0.9130741	Q14315	2338	3	R.GVAGVPAEFSIWTR.E	W12(+4.00028701)
3.9953	0.9130741	Q13813	2220	3	R.QEFAQHANAFHQWIQETR.T	W13(+3.990010829)
3.9953	0.9130741	Q05707	1306	3	R.ILPDTPQEPFALWEILNK.N	W13(+3.996999973)
3.9953	0.9130741	Q9Y490	2389	3	K.VGAIPANALDDGQWSQGLISAAR.M	W14(+3.992388534)
3.9953	0.9130741	P07237	128	3	R.EADDIVNWLK.K	W8(+3.995331903)
3.9953	0.9130741	P08237	459	3	K.GQIEEAGWSYVGGWTGQGGSK.L	W14(+3.997214401)
3.9953	0.9130741	P09093	42	3	R.VVHGEDAVPYSWPWQVSLQYEK.S	W14(+3.997400451)
3.9953	0.9130741	Q6GPI1	47	3	R.IVNGEDAVPGSWPWQVSLQDK.T	W14(+3.999437594)
3.9953	0.9130741	O75083	349	3	K.SYIYSGSHDGHINYWDSETGENDSFAGK.G	W15(+3.991636396)
3.9953	0.9130741	Q09666	2890	3	K.VDIDVPDVNVQGPDWHLK.M	W15(+3.99298671)
3.9953	0.9130741	Q09666	745	3	K.VDIDAPDVDVHGPDWHLK.M	W15(+3.993353792)
3.9953	0.9130741	P21579	260	3	K.VPMNTVDFGHVTEEWR.D	W15(+3.993907225)
3.9953	0.9130741	P00338	148	3	K.LLIVSNPVDILTYVAWK.I	W16(+3.993610013)
3.9953	0.9130741	P06744	228	3	K.TFTTQETITNAETAKEWFLQAAK.D	W17(+3.988466901)
3.9953	0.9130741	P07197	291	3	R.SQLESHSDQNMHQAEEWFK.C	W17(+3.991159826)
3.9953	0.9130741	Q09666	3470	3	K.APEVNLNAPDVDVHGPDWNLK.M	W18(+3.991335267)
3.9953	0.9130741	P11021	103	3	R.TWNDPSVQQDIK.F	W2(+3.994804157)
3.9953	0.9130741	P04004	339	3	R.DWHGVPGQVDAAMAGR.I	W2(+3.994983777)
3.9953	0.9130741	Q03135	85	3	K.IDFEDVIAEPEGTHSFDGIWK.A	W20(+3.992606973)
3.9953	0.9130741	Q16363	460	3	R.ELVDEEADEAYELLSQAESWQR.L	W20(+3.994974951)
3.9953	0.9130741	P31146	379	3	K.SDLFQEDLYPPTAGPDPALTAEEWLGGR.D	W24(+3.994489075)
3.9953	0.9130741	P02545	514	3	K.AGQVVTIWAAGAGATHSPPTDLVWK.A	W24(+3.997022767)
3.9953	0.9130741	P69905	15	3	K.AAWGKVGAHAGEYGAEALER.M	W3(+3.99345145)
3.9953	0.9130741	Q8WZ42	25776	3	R.LSWTQVSTEVQALNYK.V	W3(+3.995240315)
3.9953	0.9130741	P30086	122	3	R.YVWLVYEQDRPLK.C	W3(+3.996794738)
3.9953	0.9130741	Q99623	74	3	R.IPWFQYPIIYDIR.A	W3(+3.997375885)
3.9953	0.9130741	Q05707	210	3	R.IEWHLNAFSTKDEVIEAVR.N	W3(+3.997920544)
3.9953	0.9130741	P11137	14	3	K.APHWTSAPLTEASAHSHPPEIK.D	W4(+3.993038953)
3.9953	0.9130741	P61106	65	3	K.LQIWDTAGQER.F	W4(+3.995370286)
3.9953	0.9130741	P09417	113	3	K.QSIWTSTISSHLATK.H	W4(+3.995573442)
3.9953	0.9130741	Q13263	351	3	R.FASWALESDNNTALLLSK.K	W4(+3.99667063)
3.9953	0.9130741	Q9HBL0	650	3	R.GLNSWQQQQQQQQPRPPPR.Q	W5(+3.99261477)
3.9953	0.9130741	P00367	129	3	R.DDGSWEVIEGYR.A	W5(+3.992675288)
3.9953	0.9130741	P09429	133	3	K.LGEMWNNTAADDKQPYEK.K	W5(+3.993070427)
3.9953	0.9130741	P17931	181	3	K.LDNNWGREER.Q	W5(+3.993961204)
3.9953	0.9130741	P27824	450	3	R.IVDDWANDGWGLK.K	W5(+3.995553089)
3.9953	0.9130741	Q9NVD7	104	3	K.VLIDWINDVLVGER.I	W5(+3.997607655)

3.9953	0.9130741	A8K7I4	158	3	R.AFVHEWAHLR.W	W6(+3.994090467)
3.9953	0.9130741	O75643	267	3	R.DIDAFWLQR.Q	W6(+3.996717744)
3.9953	0.9130741	P06733	365	3	K.LAQANGWGVMVSHR.S	W7(+3.991547812)
3.9953	0.9130741	Q14697	711	3	R.YSLLPFWYTLLYQAHR.E	W7(+3.994197375)
3.9953	0.9130741	P61513	69	3	K.TVAGGAWTYNTTSAVTVK.S	W7(+3.994472475)
3.9953	0.9130741	P12814	409	3	K.ASIHEAWTDGK.E	W7(+3.994483044)
3.9953	0.9130741	P07384	392	3	R.NYPATFWVNPQFK.I	W7(+3.994890852)
3.9953	0.9130741	P09104	365	3	K.LAQENGWGVMVSHR.S	W7(+3.995589988)
3.9953	0.9130741	Q9BZZ5	443	3	K.STVTLSWKPVQK.V	W7(+3.9965889)
3.9953	0.9130741	P07451	47	3	R.HDPSLQPWSVSYDGGSAK.T	W8(+3.991587341)
3.9953	0.9130741	P02671	295	3	R.NPSSAGSWNSGSSGPGSTGNR.N	W8(+3.992959377)
3.9953	0.9130741	P02751	2399	3	K.TYHVGEQWQK.E	W8(+3.993976494)
3.9953	0.9130741	Q9Y2B0	40	3	R.ALVDELEWEIAQVDPKK.T	W8(+3.994566247)
3.9953	0.9130741	Q9Y490	2112	3	K.VGDDPAVWQLK.N	W8(+3.995557775)
3.9953	0.9130741	P09455	9	3	M.PVDFTGYWK.M	W8(+3.99591216)
3.9953	0.9130741	P15311	445	3	R.RKEDEVEEWQHR.A	W9(+3.989810243)
3.9953	0.9130741	P50454	176	3	R.SALQSINEWAAQTTDGKLPEVTK.D	W9(+3.99285613)
3.9953	0.9130741	P12883	1456	3	R.NFDKILAEWK.Q	W9(+3.993107815)
3.9953	0.9130741	O75083	584	3	R.LHHVSSLAWLDEHTLVTTSHDASVK.E	W9(+3.993810044)
3.9953	0.9130741	P11047	540	3	R.DGSEASLEWSSER.Q	W9(+3.994590978)
3.9953	0.9130741	P00352	77	3	R.QAFQIGSPWR.T	W9(+3.995469058)
4.9792	0.6640700	P06733	365	3	K.LAQANGWGVMVSHR.S	W7(+4.978259379)
15.9949	0.9191695	P68371	101	3	R.SGPFGQIFRPDNFVFGQSGAGNNWAK.G	W24(+15.982711123)
15.9949	0.9191695	P18669	167	3	R.ALPFWNEEIVPQIK.E	W5(+15.992287852)
15.9949	0.9191695	P12814	409	3	K.ASIHEAWTDGK.E	W7(+15.991187146)
15.9949	0.9191695	P60201	212	3	R.MYGVLPWNAFPGK.V	W7(+15.989470175)
15.9949	0.9191695	P17174	320	3	R.IVASTLSNPELFEEWTGNVK.T	W15(+15.988177616)
15.9949	0.9191695	P02671	295	3	R.NPSSAGSWNSGSSGPGSTGNR.N	W8(+15.99015176)
15.9949	0.9191695	P21796	75	3	K.WNTDNTLGTEITVEDQLAR.G	W1(+15.987931151)
15.9949	0.9191695	Q01813	469	3	K.EIGWTDVGGWTGQGGSILGTK.R	W10(+15.991209053)
15.9949	0.9191695	P68363	407	3	R.AFVHWYVGEGMEEGEFSEAR.E	W5(+15.988123358)
15.9949	0.9191695	P02671	360	3	R.PGSTGTWNPGSSER.G	W7(+15.992473943)
15.9949	0.9191695	O43707	736	3	R.VGWEQLLTTIAR.T	W3(+15.98693563)
15.9949	0.9191695	P27824	304	3	K.IPDPEAVKPDDWDEDAPAK.I	W12(+15.992482182)
15.9949	0.9191695	P00338	148	3	K.LLIVSNPVDILTYVAWK.I	W16(+15.99445328)
15.9949	0.9191695	P00505	162	3	R.DVFLPKPTWGNHTPIFR.D	W9(+15.993506558)
15.9949	0.9191695	P12814	381	3	K.GYEEWLLNEIR.R	W5(+15.994530811)
15.9949	0.9191695	P33241	267	3	R.WETGEVQAQSAAK.T	W1(+15.991411252)
15.9949	0.9191695	P00918	123	3	K.YAAELHLVHWNTK.Y	W10(+15.992109173)
15.9949	0.9191695	P04792	22	3	R.GPSWDPFRDWYPHSR.L	W10(+15.992636201)
15.9949	0.9191695	Q15746	1842	3	K.IEGYPDPEVVWFKDDQSIR.E	W11(+15.991231436)
15.9949	0.9191695	P19835	71	3	K.ALENPQPHPGWQGTLK.A	W11(+15.993191343)
15.9949	0.9191695	P02790	398	3	K.SGAQATWTELPWPHEK.V	W12(+15.985574613)
15.9949	0.9191695	P13637	896	3	R.TVNDLEDSYGQQWTYEQR.K	W13(+15.997511317)
15.9949	0.9191695	P13796	387	3	R.YPALHKPENQDIDWGALEGETR.E	W14(+15.992892432)
15.9949	0.9191695	P13073	137	3	K.HYVYGPLPQSFDKEWVAK.Q	W15(+15.988905313)
15.9949	0.9191695	P00738	133	3	K.LRTEGDGVYTLNNEKQWINK.A	W17(+15.988926611)
15.9949	0.9191695	P0DMV9	90	3	K.HWPFQVINDGDKPK.V	W2(+15.996226862)
15.9949	0.9191695	P04179	205	3	K.AIWNVINWENVTER.Y	W3(+15.993508614)
15.9949	0.9191695	P35609	724	3	R.VGWELLLTTIAR.T	W3(+15.995506779)
15.9949	0.9191695	P05451	113	3	R.WHWSSGSLVSYK.S	W3(+15.995850014)
15.9949	0.9191695	P14866	283	3	K.NDQDTWDYTNPNLSGQGDPGSNPNKR.Q	W6(+15.992635405)
15.9949	0.9191695	P02675	432	3	K.EDGGGWWYNR.C	W6(+15.992906176)
15.9949	0.9191695	P05109	54	3	K.KGADVWFK.E	W6(+15.995684832)
15.9949	0.9191695	P06396	605	3	K.TPSAAYLWVGTGASEAEK.T	W8(+15.991805196)
15.9949	0.9191695	P12883	816	3	R.DSLLVIQWNIR.A	W8(+15.993716137)
15.9949	0.9191695	P15311	445	3	R.RKEDEVEEWQHR.A	W9(+15.991994222)
15.9949	0.9191695	P50454	176	3	R.SALQSINEWAAQTTDGKLPEVTK.D	W9(+15.99404031)
15.9949	0.9191695	P19237	161	3	K.ERPVEVGDWRK.N	W9(+15.994821124)
19.9903	0.3691468	P60709	86	3	K.IWHHTFYNELR.V	W2(+19.985630351)
19.9903	0.3691468	P68871	38	3	R.LLVVYPWTQR.F	W7(+19.990298441)
19.9903	0.3691468	P09429	133	3	K.LGEMWNNTAADDKQPYEK.K	W5(+19.987516228)
19.9903	0.3691468	P26583	133	3	K.KLGEMWSEQSAK.D	W6(+19.987914223)
19.9903	0.3691468	P13796	524	3	K.VNDDIIVNWVNETLR.E	W9(+19.990720155)
31.9899	0.9376182	P10809	68	3	R.TVIIEQSWGSPK.V	W8(+31.983839914)
31.9899	0.9376182	Q16762	36	3	R.VLDASWYSPGTR.E	W6(+31.985370235)
31.9899	0.9376182	P09417	113	3	K.QSIWTSTISSHLATK.H	W4(+31.98489229)
31.9899	0.9376182	Q00610	1340	3	R.EHLELFWSR.V	W7(+31.985671573)
31.9899	0.9376182	P61204	66	3	K.NISFTVWDVGGQDK.I	W7(+31.980467389)

31.9899	0.9376182	Q9UBB6	402	3	R.ILGAWLAEETSSLR.K	W5(+31.984559098)
31.9899	0.9376182	P13591	656	3	R.ALSSEWKPEIR.L	W6(+31.986094104)
31.9899	0.9376182	P12236	71	3	K.EQGVLSFWR.G	W8(+31.985798056)
31.9899	0.9376182	Q13813	1983	3	K.LDENSAFLQFNWK.A	W12(+31.985122572)
31.9899	0.9376182	P09093	262	3	R.VSAFIDWIEETIASH.-	W7(+31.985116666)
31.9899	0.9376182	P00338	324	3	K.SADTLWGIQK.E	W6(+31.986431978)
31.9899	0.9376182	Q13813	343	3	K.REELITNWEQIR.T	W8(+31.986749902)
31.9899	0.9376182	Q9Y6R7	1300	3	R.FAVLQENVAWGNGR.V	W10(+31.986982567)
31.9899	0.9376182	P17174	141	3	K.NTPVYVSSPTWENHNAVFSAAGFK.D	W11(+31.987207609)
31.9899	0.9376182	O75369	2217	3	R.GEAGVPAEFSIWTR.E	W12(+31.986557049)
31.9899	0.9376182	P02751	1833	3	K.FTQVTPTSLSAQWTPPNVQLTGYR.V	W13(+31.989410498)
31.9899	0.9376182	P35749	591	3	K.VDYNASAWLTK.N	W8(+31.986482996)
31.9899	0.9376182	P01023	178	3	R.IAQWQSFQLEGGLK.Q	W4(+31.989936987)
31.9899	0.9376182	P24534	172	3	R.SIQADGLVWGSSK.L	W9(+31.986010954)
31.9899	0.9376182	Q92777	126	3	K.VLLVVDEPHADWAK.C	W12(+31.987383226)
31.9899	0.9376182	Q09666	1506	3	K.VDINAPDVEVHGPDWHLK.M	W15(+31.984742853)
31.9899	0.9376182	P04179	205	3	K.AIWNVINWENVTER.Y	W3(+31.987771309)
31.9899	0.9376182	P05023	418	3	K.TSATWLALSR.I	W5(+31.986682915)
31.9899	0.9376182	Q5SSJ5	136	3	K.TIPSWATLSASQLAR.A	W5(+31.987638303)
31.9899	0.9376182	P49419	363	3	R.VGNPWDPNVLYGPLHTK.Q	W5(+31.988959603)
31.9899	0.9376182	P04217	101	3	R.SGLSTGWTQLSK.L	W7(+31.985649156)
31.9899	0.9376182	P28331	319	3	K.GLLTYTSWEDALSR.V	W8(+31.962406285)
31.9899	0.9376182	P56470	256	3	R.NSLLNGSWGSEEK.K	W8(+31.985876513)
31.9899	0.9376182	P61019	100	3	R.DTFNHLTTWLEDAR.Q	W9(+31.986699498)
31.9899	0.9376182	E9PAV3	961	3	K.WAPTPPAATPPSPK.G	W1(+31.989681021)
31.9899	0.9376182	P02751	831	3	R.WSRPQAPITGYR.I	W1(+31.989889525)
31.9899	0.9376182	P08758	187	3	K.WGTDEEKFITIFGTR.S	W1(+31.990782032)
31.9899	0.9376182	P33241	267	3	R.WETGEVQAQSAAK.T	W1(+31.9908009)
31.9899	0.9376182	P35606	396	3	K.SFGSAQEFAWAHDSSEYAIR.E	W10(+31.991179285)
31.9899	0.9376182	Q1KMD3	388	3	K.NGEDLGVAFWISKDSLADR.A	W10(+31.991977765)
31.9899	0.9376182	P39019	52	3	K.ELAPYDENWFYTR.A	W9(+31.989800163)
31.9899	0.9376182	Q13557	238	3	K.AGAYDFPSPEWDTVTPEAK.D	W11(+31.987072804)
31.9899	0.9376182	Q16795	233	3	R.FGPIPLGSLGWK.T	W11(+31.99045529)
31.9899	0.9376182	P62244	89	3	R.FDVQLKDLEKWQNNLLPSR.Q	W11(+31.994346046)
31.9899	0.9376182	Q9UGI8	113	3	K.KNVSINTVTYEWAPPVQNQALAR.Q	W12(+31.99706407)
31.9899	0.9376182	Q04760	171	3	K.GLAFIQDPDGYWIEILNPNK.M	W12(+31.983281587)
31.9899	0.9376182	P22314	109	3	K.AVTLHDQGTAQWADLSSQFYLR.E	W12(+31.990656247)
31.9899	0.9376182	Q8WZ42	14435	3	K.FGDITKDSVHLTWEPPDDDGGSPLTGYVVEKR.E	W13(+31.986742458)
31.9899	0.9376182	Q14896	322	3	R.DSKLEAPAEEDVWEILR.Q	W13(+31.997161308)
31.9899	0.9376182	Q9UI47	67	3	R.ASVLLASVEEATWNLLDKGEK.I	W13(+32.010229027)
31.9899	0.9376182	P08238	289	3	K.YIDQEELNKTKPIWTR.N	W14(+31.987252078)
31.9899	0.9376182	P33991	624	3	R.TSVLAAANPIESQWNPK.K	W14(+31.988949583)
31.9899	0.9376182	P07900	297	3	K.YIDQEELNKTKPIWTRNPDDITNEEYGEFYK.S	W14(+31.989194196)
31.9899	0.9376182	B9A064	187	3	K.YAASSYLSLTPEQWK.S	W14(+31.989486133)
31.9899	0.9376182	Q9UQ80	170	3	R.LVKPGNQNTQVTEAWNK.V	W15(+31.98560295)
31.9899	0.9376182	Q09666	2044	3	K.MDIDAPDVDVHGPDWHLK.M	W15(+31.987740339)
31.9899	0.9376182	Q09666	3146	3	K.VDINAPDVDVQGPDWHLK.M	W15(+31.988581272)
31.9899	0.9376182	Q09666	3598	3	K.VDINAPDVDVHGPDWHLK.M	W15(+31.988843987)
31.9899	0.9376182	P15085	53	3	K.VKELEDLEHLQLDFWR.G	W15(+31.989682259)
31.9899	0.9376182	P02787	569	3	K.HQTVPQNTGGKNPDPWAK.N	W16(+31.989783486)
31.9899	0.9376182	P0DPI2	204	3	R.GVEVTVGHEQEEGGKWPYAGTAEAIK.A	W16(+31.994504998)
31.9899	0.9376182	P01023	1069	3	R.AYIFIDEAHITQALIWLSQR.Q	W16(+31.994677448)
31.9899	0.9376182	Q9BPW8	256	3	R.GWDENVYYTVPLVR.H	W2(+31.987387366)
31.9899	0.9376182	Q9UJU6	237	3	R.TWEQQQEVVSR.N	W2(+31.989612057)
31.9899	0.9376182	O15143	86	3	R.TWKPTLVILR.I	W2(+31.990337636)
31.9899	0.9376182	Q01813	141	3	K.EWSGLLEELAR.N	W2(+31.991239326)
31.9899	0.9376182	Q13813	905	3	R.RQDLEDSLQAQQYFADANEAESWMR.E	W23(+31.98707939)
31.9899	0.9376182	O00264	156	3	K.EALKDEYDDLSDLTAAQQETLSDWESQFTFK.Y	W24(+31.988387058)
31.9899	0.9376182	Q08211	176	3	R.KEEQEVQATLESEEVDLNAGLHGNWTLENAK.A	W25(+31.986292168)
31.9899	0.9376182	P27797	347	3	K.SGTIFDNFLITNDEAYAEEFGNETWGVTK.A	W25(+32.009808961)
31.9899	0.9376182	P00505	162	3	K.PTWGNHTPIFR.D	W3(+31.987731603)
31.9899	0.9376182	P35237	173	3	R.GNWDEQFDKENTEER.L	W3(+31.988095646)
31.9899	0.9376182	P01009	218	3	K.GKWERPFEVK.D	W3(+31.988222083)
31.9899	0.9376182	Q8WZ42	8266	3	K.VLWYKDETEIQESSKFR.M	W3(+31.989274489)
31.9899	0.9376182	P12277	218	3	R.GIWHNDNK.T	W3(+31.990043401)
31.9899	0.9376182	P04217	409	3	R.ATWSGAVLAGR.D	W3(+31.990646089)
31.9899	0.9376182	P05451	113	3	R.WHWSSGSLVSYK.S	W3(+31.991198056)
31.9899	0.9376182	O96000	5	3	M.PDSWDKDVYPEPPR.R	W4(+31.98828165)
31.9899	0.9376182	P35749	37	3	K.RLVWVPSEK.Q	W4(+31.990378046)

31.9899	0.9376182	P06732	228	3	K.SFLVWVNEEDHLR.V	W5(+31.986981074)
31.9899	0.9376182	P01023	858	3	R.QTVSWAVTPK.S	W5(+31.987302981)
31.9899	0.9376182	P02751	927	3	K.VTIMWTPPESAVTGYR.V	W5(+31.987771251)
31.9899	0.9376182	Q01844	429	3	K.AAVEWFDGKDFQGSK.L	W5(+31.988018049)
31.9899	0.9376182	O14548	16	3	K.LAGAWASEAYSPQGLKPVVSTEAPPIIFATPTK.L	W5(+31.98859965)
31.9899	0.9376182	O14773	470	3	R.VPIPWVSGTSASTPVFGGILSLINEHR.I	W5(+31.988821963)
31.9899	0.9376182	P01024	1077	3	R.APSTWLTAYVVK.V	W5(+31.989670085)
31.9899	0.9376182	Q9NVD7	104	3	K.VLIDWINDVLVGER.I	W5(+31.992602773)
31.9899	0.9376182	P35637	353	3	K.AAIDWFDGKEFSGNPIKVSFATR.R	W5(+32.000567789)
31.9899	0.9376182	Q8NBS9	227	3	K.ALAPTWEQLALGLEHSETVK.I	W6(+31.986284087)
31.9899	0.9376182	O94875	975	3	K.RVDQNWYEGK.I	W6(+31.987064202)
31.9899	0.9376182	P49458	7	3	M.PQYQTWEEFSR.A	W6(+31.989524766)
31.9899	0.9376182	Q6ZS81	2126	3	K.TVQTLWQQLVAQR.Q	W6(+31.989786933)
31.9899	0.9376182	Q01082	30	3	R.WDVDDWDNENSSAR.L	W6(+31.989882475)
31.9899	0.9376182	P15144	601	3	R.QQQDYWLIDVR.A	W6(+31.991165354)
31.9899	0.9376182	O95573	687	3	R.LSPEPWTPETGLVTDAFK.L	W6(+31.993785437)
31.9899	0.9376182	P61513	69	3	K.TVAGGAWTYNTTSAVTVK.S	W7(+31.986782045)
31.9899	0.9376182	P38646	153	3	R.ASNGDAWVEAHGK.L	W7(+31.988777327)
31.9899	0.9376182	P26038	445	3	K.ESEAVEWQQK.A	W7(+31.989160585)
31.9899	0.9376182	Q09666	15	3	R.ELLLPNWQGSGSHGLTIAQR.D	W7(+31.997290193)
31.9899	0.9376182	P18206	394	3	K.KIDAAQNWLADPNGGPEGEEQIR.G	W8(+31.985700028)
31.9899	0.9376182	Q9GZV7	282	3	K.VGHLYAAWK.F	W8(+31.987203328)
31.9899	0.9376182	P52179	443	3	K.HFQPEIQWYR.N	W8(+31.988624294)
31.9899	0.9376182	P02751	65	3	K.HYQINQQWER.T	W8(+31.988765854)
31.9899	0.9376182	P07099	150	3	K.PLLMVHGWPGSFYEFYK.I	W8(+31.9895675)
31.9899	0.9376182	P14866	549	3	R.SSSGLLEWESK.S	W8(+31.989633518)
88.9942	0.1672632	P68032	81	3	K.YPIEHGIITNWDDMEK.I	W11(+88.989986512)
88.9942	0.1672632	P68871	38	3	R.LLVVYPWTQR.F	W7(+88.991519144)
209.0179	0.7899121	Q9Y490	2112	3	K.VGDDPAVWQLK.N	W8(+209.017408361)
32.9748	0.6928963	Q9BVA1	344	3	K.NSSYFVEWIPNNVK.T	W8(+32.980332353)
32.9748	0.6928963	P02686	250	3	R.GLSLSRFSWGAEGQRPGFGYGGR.A	W9(+32.96942019)
14.9827	0.2716954	P13611	287	3	R.LATVGELQAAWR.N	W11(+14.971696603)
14.9827	0.2716954	P17174	141	3	K.NTPVYVSSPTWENHNAVFSAAGFK.D	W11(+14.972206247)
14.9827	0.2716954	P55072	476	3	R.ETVVEVPQVTWEDIGGLEDVKR.E	W11(+14.973481177)
14.9827	0.2716954	P98160	1989	3	R.AAGVPSATITWRK.E	W11(+14.976738648)
14.9827	0.2716954	P22314	647	3	K.NFPNAIEHTLQWAR.D	W12(+14.972025732)
14.9827	0.2716954	P08238	289	3	K.YIDQEELNKTKPIWTRNPDDITQEEYGEFYK.S	W14(+14.973299992)
14.9827	0.2716954	P63261	86	3	K.IWHHTFYNELR.V	W2(+14.972080546)
14.9827	0.2716954	P21333	582	3	R.AWGPGLEGGVVGK.S	W2(+14.97566711)
14.9827	0.2716954	P09417	113	3	K.QSIWTSTISSHLATK.H	W4(+14.972563188)
14.9827	0.2716954	P04406	313	3	K.LISWYDNEFGYSNR.V	W4(+14.974283106)
14.9827	0.2716954	P60201	212	3	R.MYGVLPWNAFPGK.V	W7(+14.974699667)
14.9827	0.2716954	P06396	605	3	K.TPSAAYLWVGTGASEAEK.T	W8(+14.974715352)
14.9827	0.2716954	P27824	287	3	R.EIEDPEDRKPEDWDERPK.I	W13(+14.979083133)
14.9827	0.2716954	Q16555	291	3	K.GTVVYGEPITASLGTDGSHYWSK.N	W21(+14.979548385)
14.9827	0.2716954	Q9BVA1	21	3	K.FWEVISDEHGIDPTGSYHGDSDLQLER.I	W2(+14.98536864)
-124.1118	0.2696443	P35749	30	2	K.NFINSPVAQADWAAKR.L	W12(-124.107904369)
-59.0506	0.1389893	P69905	15	2	K.AAWGKVGAHAGEYGAEALER.M	W3(-59.052874234)
-59.0506	0.1389893	P68871	16	2	K.SAVTALWGKVNVDEVGGEALGR.L	W7(-59.0499808)
-13.0308	0.3243439	P09417	113	2	K.QSIWTSTISSHLATK.H	W4(-13.026263857)
-13.0308	0.3243439	Q01082	1574	2	K.QLWGLLIEETEKR.H	W3(-13.030443452)
3.9953	0.9130741	P01876	264	2	R.WLQGSQELPR.E	W1(+3.990575037)
3.9953	0.9130741	P06744	182	2	R.VWYVSNIDGTHIAK.T	W2(+3.989962052)
3.9953	0.9130741	P68371	21	2	K.FWEVISDEHGIDPTGTYHGDSDLQLER.I	W2(+3.992057443)
3.9953	0.9130741	Q13813	1983	2	K.LDENSAFLQFNWK.A	W12(+3.993179212)
3.9953	0.9130741	P02763	140	2	K.NWGLSVYADKPETTK.E	W2(+3.992772316)
3.9953	0.9130741	P13796	524	2	K.VNDDIIVNWVNETLR.E	W9(+3.99143929)
3.9953	0.9130741	Q02817	557	2	K.TASGLVEATGAGFANTWK.A	W17(+3.995199594)
3.9953	0.9130741	P05023	418	2	K.TSATWLALSR.I	W5(+3.99278643)
3.9953	0.9130741	Q16762	36	2	R.VLDASWYSPGTR.E	W6(+3.991717892)
3.9953	0.9130741	P02751	831	2	R.WSRPQAPITGYR.I	W1(+3.990146952)
3.9953	0.9130741	P21796	75	2	K.WNTDNTLGTEITVEDQLAR.G	W1(+3.992081542)
3.9953	0.9130741	P37837	296	2	R.WLHNEDQMAVEK.L	W1(+3.995127308)
3.9953	0.9130741	P06744	380	2	R.VDHQTGPIVWGEPGTNGQHAFYQLIHQGTK.M	W10(+3.987543602)
3.9953	0.9130741	P55268	594	2	R.LVTPGETPSWTGSGFVR.L	W10(+3.991920207)
3.9953	0.9130741	P01160	102	2	R.DGGALGRGPWDSSDR.S	W10(+3.994950162)
3.9953	0.9130741	P10644	262	2	K.VSILESLDKWER.L	W10(+3.995170341)
3.9953	0.9130741	Q9Y3I0	318	2	K.GMAAAGNYAWVNR.S	W10(+3.99550622)
3.9953	0.9130741	P07384	614	2	K.LGLVEFNILWNR.I	W10(+3.995803431)

3.9953	0.9130741	P10745	1206	2	R.SVGASDGSSWEGVGVTPHVVVPAEEALAR.A	W10(+3.996902799)
3.9953	0.9130741	P21399	66	2	K.KQDIENILHWNVTQHK.N	W10(+3.997499506)
3.9953	0.9130741	P19835	71	2	K.ALENPQPHPGWQGTLK.A	W11(+3.99209271)
3.9953	0.9130741	Q9NR12	49	2	K.AAQAGVAVGDWVLSIDGENAGSLTHIEAQNK.I	W11(+3.993112746)
3.9953	0.9130741	P14866	283	2	R.LNVFKNDQDTWDYTNPNLSGQGDPGSNPNKR.Q	W11(+3.99521699)
3.9953	0.9130741	Q86UX7	16	2	K.TASGDYIDSSWELR.V	W11(+3.995382376)
3.9953	0.9130741	Q8WZ42	7701	2	R.VSGSAPISVGWFQDGNEIVSGPK.C	W11(+3.995916463)
3.9953	0.9130741	P50213	63	2	K.IFDAAKAPIQWEER.N	W11(+3.996169938)
3.9953	0.9130741	O15075	646	2	R.FSAVQVLEHPWVNDDGLPENEHQLSVAGK.I	W11(+3.996303085)
3.9953	0.9130741	P17600	126	2	R.VLLVIDEPHTDWAK.Y	W12(+3.992100013)
3.9953	0.9130741	P22314	109	2	K.AVTLHDQGTAQWADLSSQFYLR.E	W12(+3.993097653)
3.9953	0.9130741	P27824	304	2	K.IPDPEAVKPDDWDEDAPAK.I	W12(+3.99339771)
3.9953	0.9130741	Q14194	412	2	R.IAVGSDADVVIWDPDK.L	W12(+3.993717018)
3.9953	0.9130741	P22314	647	2	K.NFPNAIEHTLQWAR.D	W12(+3.995510981)
3.9953	0.9130741	O43707	664	2	R.QFASQANVVGPWIQTK.M	W12(+3.996241039)
3.9953	0.9130741	P35613	255	2	K.SESVPPVTDWAWYK.I	W12(+3.99776096)
3.9953	0.9130741	P15085	367	2	K.AIYQASGSTIDWTYSQGIK.Y	W12(+3.997870409)
3.9953	0.9130741	Q9BPU6	405	2	R.IIPGADADVVVWDPEATK.T	W12(+4.002910344)
3.9953	0.9130741	P40925	218	2	K.EVGVYEALKDDSWLK.G	W13(+3.993300679)
3.9953	0.9130741	Q07075	938	2	R.EQVLETVKNNIEWLK.Q	W13(+3.994608124)
3.9953	0.9130741	Q9UL46	112	2	K.VLSLLALVKPEVWTLK.E	W13(+3.994843639)
3.9953	0.9130741	Q5BJF2	65	2	K.EFKDPLLQEPPAWFK.S	W13(+3.996903221)
3.9953	0.9130741	P04114	1981	2	K.VSALLTPAEQTGTWK.L	W14(+3.996308215)
3.9953	0.9130741	Q09666	3598	2	K.VDINAPDVDVHGPDWHLK.M	W15(+3.99178696)
3.9953	0.9130741	Q14896	683	2	R.LDVPISGDPAPTVIWQK.A	W15(+3.99534464)
3.9953	0.9130741	Q09666	4648	2	K.VDIDVPDVDVQGPDWHLK.M	W15(+3.995726497)
3.9953	0.9130741	P02679	253	2	K.EGFGHLSPTGTTEFWLGNEK.I	W15(+3.996952532)
3.9953	0.9130741	Q9UN36	234	2	R.NIITHAPNLDNIELYWNSYNNR.R	W16(+4.000952647)
3.9953	0.9130741	P06733	301	2	K.DYPVVSIEDPFDQDDWGAWQK.F	W16(+4.001766939)
3.9953	0.9130741	P42167	233	2	R.VEHNQSYSQAGITETEWTSGSSK.G	W17(+3.996029391)
3.9953	0.9130741	P12036	292	2	R.AQLEGHAVQSTLQSEEWFR.V	W17(+3.996556304)
3.9953	0.9130741	P17612	31	2	K.KWESPAQNTAHLDQFER.I	W2(+3.994191873)
3.9953	0.9130741	P21980	404	2	K.EGDLSTKYDAPFVFAEVNADVVDWIQQDDGSVHK.S	W24(+4.000242431)
3.9953	0.9130741	Q9ULV4	377	2	R.KSDLFQDDLYPDTAGPEAALEAEEWFEGK.N	W25(+3.997213809)
3.9953	0.9130741	Q01082	1779	2	R.VDTVNHLADELINSGHSDAATIAEWK.D	W25(+4.000173313)
3.9953	0.9130741	P27797	347	2	K.SGTIFDNFLITNDEAYAEEFGNETWGVTK.A	W25(+4.009612569)
3.9953	0.9130741	Q06520	77	2	R.SPWVESEIGYTALSETESPR.L	W3(+3.993121506)
3.9953	0.9130741	Q8WZ42	3272	2	K.ISWYKEEQLLSTGFK.C	W3(+3.993585207)
3.9953	0.9130741	Q99715	1675	2	R.GTWDHGASDVSLYR.I	W3(+3.99368168)
3.9953	0.9130741	P55786	692	2	R.LGWDPKPGEGHLDALLR.G	W3(+3.993868671)
3.9953	0.9130741	A8K7I4	273	2	R.STWEVIRDSEDFKK.T	W3(+3.99611393)
3.9953	0.9130741	P08237	132	2	R.SEWSDLLSDLQK.A	W3(+3.996490996)
3.9953	0.9130741	Q99715	1689	2	R.ITWAPFGSSDK.M	W3(+3.997595808)
3.9953	0.9130741	P16233	419	2	K.FIWYNNVINPTLPR.V	W3(+3.999574372)
3.9953	0.9130741	O95373	976	2	R.NPVWYQALTHGLNEEQR.K	W4(+3.990424204)
3.9953	0.9130741	P33241	230	2	K.IDQWLEQYTQAIETAGR.T	W4(+3.992825444)
3.9953	0.9130741	P01023	178	2	R.IAQWQSFQLEGGLK.Q	W4(+3.99469773)
3.9953	0.9130741	Q8WZ42	18578	2	R.KDTWGVVSSGSSK.T	W4(+3.994783505)
3.9953	0.9130741	P02787	479	2	R.TAGWNIPMGLLYNK.I	W4(+3.995157079)
3.9953	0.9130741	P12110	696	2	K.NLEWIAGGTWTPSALK.F	W4(+3.995578176)
3.9953	0.9130741	P62826	64	2	K.FNVWDTAGQEK.F	W4(+3.996710676)
3.9953	0.9130741	P46783	69	2	R.HFYWYLTNEGIQYLR.D	W4(+3.996943983)
3.9953	0.9130741	P37837	147	2	K.LSSTWEGIQAGK.E	W5(+3.991508531)
3.9953	0.9130741	Q8WZ42	21420	2	K.ATLTWTPPLEDGGSPIK.S	W5(+3.993704052)
3.9953	0.9130741	P35237	137	2	K.HINTWVAEK.T	W5(+3.993976767)
3.9953	0.9130741	Q02252	93	2	R.AFPAWADTSVLSR.Q	W5(+3.995147959)
3.9953	0.9130741	P11766	14	2	K.AAVAWEAGKPLSIEEIEVAPPK.A	W5(+3.995236175)
3.9953	0.9130741	Q5SSJ5	136	2	K.TIPSWATLSASQLAR.A	W5(+3.996061154)
3.9953	0.9130741	P15311	217	2	K.GTDLWLGVDALGLNIYEKDDKLTPK.I	W5(+3.996086253)
3.9953	0.9130741	P35637	353	2	K.AAIDWFDGKEFSGNPIK.V	W5(+3.996087678)
3.9953	0.9130741	Q01105	95	2	K.IPNFWVTTFVNHPQVSALLGEEDEEALHYLTR.V	W5(+3.99943625)
3.9953	0.9130741	P61313	120	2	R.VLNSYWVGEDSTYK.F	W6(+3.992306716)
3.9953	0.9130741	O43488	256	2	R.FFGNSWAETYR.N	W6(+3.992933292)
3.9953	0.9130741	Q9Y6R7	5349	2	K.AGVQVWLGANGK.V	W6(+3.993032954)
3.9953	0.9130741	Q6ZS81	2126	2	K.TVQTLWQQLVAQR.Q	W6(+3.993937324)
3.9953	0.9130741	O15230	2939	2	K.STGDPWLTDGSYLDGTGFAR.I	W6(+3.994068556)
3.9953	0.9130741	P49458	7	2	M.PQYQTWEEFSR.A	W6(+3.99489586)
3.9953	0.9130741	P08514	983	2	R.GEAQVWTQLLR.A	W6(+3.994915027)
3.9953	0.9130741	P00338	324	2	K.SADTLWGIQK.E	W6(+3.996197603)

3.9953	0.9130741	Q15149	681	2	R.VDGAEWGVDLPSVEAQLGSHR.G	W6(+3.998551573)
3.9953	0.9130741	P00488	165	2	R.MYVAVWTPYGVLR.T	W6(+3.999913681)
3.9953	0.9130741	P00846	48	2	R.LITTQQWLIK.L	W7(+3.992804993)
3.9953	0.9130741	P14621	39	2	R.KIGVVGWVK.N	W7(+3.993038801)
3.9953	0.9130741	Q8WZ42	18283	2	R.DVASAQWSPLSATSK.K	W7(+3.993462273)
3.9953	0.9130741	P50440	149	2	R.RPDPIDWSLK.Y	W7(+3.994343867)
3.9953	0.9130741	P00738	284	2	R.VGYVSGWGR.N	W7(+3.995379686)
3.9953	0.9130741	Q8WZ42	27225	2	K.ESAVLSWDVPENDGGAPVK.N	W7(+3.996870289)
3.9953	0.9130741	Q16643	29	2	R.EESAADWALYTYEDGSDDLK.L	W7(+3.999850348)
3.9953	0.9130741	Q09666	15	2	R.ELLLPNWQGSGSHGLTIAQR.D	W7(+4.002844392)
3.9953	0.9130741	Q13813	343	2	K.REELITNWEQIR.T	W8(+3.990486333)
3.9953	0.9130741	P00915	98	2	R.LFQFHFHWGSTNEHGSEHTVDGVK.Y	W8(+3.991520516)
3.9953	0.9130741	P02749	335	2	K.EHSSLAFWK.T	W8(+3.99379247)
3.9953	0.9130741	P20916	196	2	R.LREDEGTWVQVSLLHFVPTR.E	W8(+3.993931769)
3.9953	0.9130741	Q14697	566	2	K.DAQHYGGWEHR.D	W8(+3.994126912)
3.9953	0.9130741	P14866	549	2	R.SSSGLLEWESK.S	W8(+3.994150119)
3.9953	0.9130741	P12883	1371	2	K.ANSEVAQWR.T	W8(+3.994244809)
3.9953	0.9130741	P98160	991	2	R.LLSGPYFWSLPSR.F	W8(+3.995430424)
3.9953	0.9130741	Q14019	26	2	R.DDGSAVIWVTFK.Y	W8(+3.995521502)
3.9953	0.9130741	O15061	76	2	R.QQLDELSWATALAEGER.D	W8(+3.995620963)
3.9953	0.9130741	Q01082	1713	2	R.EVDDLEQWIAER.E	W8(+3.99564744)
3.9953	0.9130741	P18206	394	2	K.KIDAAQNWLADPNGGPEGEEQIR.G	W8(+3.996198075)
3.9953	0.9130741	P08237	453	2	K.GQIEEAGWSYVGGWTGQGGSK.L	W8(+3.996237838)
3.9953	0.9130741	P18124	142	2	R.IVEPYIAWGYPNLK.S	W8(+3.996661732)
3.9953	0.9130741	P98160	669	2	R.QVQFSEEHWVHESGRPVQR.A	W9(+3.988340768)
3.9953	0.9130741	P53999	110	2	K.GISLNPEQWSQLK.E	W9(+3.992732506)
3.9953	0.9130741	P13797	527	2	K.ANDDIIVNWVNR.T	W9(+3.994329467)
3.9953	0.9130741	P52179	1515	2	K.TGVTGEQIWLQINEPTPNDKGK.Y	W9(+3.994503828)
3.9953	0.9130741	P05093	220	2	K.DSLVDLVPWLK.I	W9(+3.995442169)
3.9953	0.9130741	P13639	685	2	K.DSVVAGFQWATK.E	W9(+3.995822567)
3.9953	0.9130741	P30711	101	2	R.ARVDEYLAWQHTTLR.R	W9(+3.997607717)
3.9953	0.9130741	P09211	39	2	K.EEVVTVETWQEGSLK.A	W9(+3.997637124)
3.9953	0.9130741	P09543	192	2	K.DFLPLYFGWFLTK.K	W9(+3.998657274)
4.9792	0.6640700	P21796	210	2	K.KLETAVNLAWTAGNSNTR.F	W10(+4.979070155)
15.9949	0.9191695	P01876	264	2	R.WLQGSQELPR.E	W1(+15.987157068)
15.9949	0.9191695	P08133	192	2	K.WGTDEAQFIYILGNR.S	W1(+15.987373238)
15.9949	0.9191695	Q16891	384	2	R.ELDSITPEVLPGWK.G	W13(+15.989541836)
15.9949	0.9191695	P08247	228	2	K.ETGWAAPFLR.A	W4(+15.987359906)
15.9949	0.9191695	Q00610	1340	2	R.EHLELFWSR.V	W7(+15.990188174)
15.9949	0.9191695	Q01082	1500	2	R.DVEDEILWVGER.M	W8(+15.992794107)
15.9949	0.9191695	P56470	84	2	K.VVFNTLQGGKWGSEER.K	W11(+15.994059691)
15.9949	0.9191695	Q16762	36	2	R.VLDASWYSPGTR.E	W6(+15.991839962)
15.9949	0.9191695	P02787	460	2	K.SASDLTWDNLK.G	W7(+15.99020909)
15.9949	0.9191695	Q14195	412	2	R.ISVGSDSDLVIWDPDAVK.I	W12(+15.994282272)
15.9949	0.9191695	P31930	429	2	R.RIPLAEWESR.I	W7(+15.992443254)
15.9949	0.9191695	Q99627	153	2	K.GILEQGWQADSTTR.M	W7(+15.993564913)
15.9949	0.9191695	P56470	256	2	R.NSLLNGSWGSEEK.K	W8(+15.993200732)
15.9949	0.9191695	P07237	128	2	R.EADDIVNWLK.K	W8(+15.995698113)
15.9949	0.9191695	P04264	212	2	K.WELLQQVDTSTR.T	W1(+15.99274106)
15.9949	0.9191695	Q99798	657	2	R.WVVIGDENYGEGSSR.E	W1(+15.993456698)
15.9949	0.9191695	P37837	296	2	R.WLHNEDQMAVEK.L	W1(+15.993906604)
15.9949	0.9191695	P04746	149	2	R.DFPAVPYSGWDFNDGK.C	W10(+15.989754175)
15.9949	0.9191695	Q8WZ42	19475	2	K.IADASPDEGWKR.C	W10(+15.994766108)
15.9949	0.9191695	P26439	345	2	R.DLAYKPLYSWEEAK.Q	W10(+15.995299792)
15.9949	0.9191695	P10644	262	2	K.VSILESLDKWER.L	W10(+15.995628105)
15.9949	0.9191695	P07686	424	2	K.LAPGTIVEVWKDSAYPEELSR.V	W10(+15.996095126)
15.9949	0.9191695	P10745	1206	2	R.SVGASDGSSWEGVGVTPHVVVPAEEALAR.A	W10(+16.000564909)
15.9949	0.9191695	P55072	476	2	R.ETVVEVPQVTWEDIGGLEDVKR.E	W11(+15.992560669)
15.9949	0.9191695	P61764	478	2	R.ISEQTYQLSRWTPIIK.D	W11(+15.993048338)
15.9949	0.9191695	P15121	112	2	K.LDYLDLYLIHWPTGFKPGK.E	W11(+15.997860073)
15.9949	0.9191695	P35609	652	2	R.QFAAQANAIGPWIQNK.M	W12(+15.994222767)
15.9949	0.9191695	P04746	331	2	R.GHGAGGASILTFWDAR.L	W13(+15.990921246)
15.9949	0.9191695	P40925	218	2	K.EVGVYEALKDDSWLK.G	W13(+15.994935342)
15.9949	0.9191695	P06748	290	2	R.MTDQEAIQDLWQWR.K	W13(+15.995231727)
15.9949	0.9191695	Q5BJF2	65	2	K.EFKDPLLQEPPAWFK.S	W13(+15.997818748)
15.9949	0.9191695	Q00839	811	2	K.NQSQGYNQWQQGQFWGQK.P	W15(+15.988932845)
15.9949	0.9191695	Q09666	4184	2	K.VDIDVPDVDVQGPDWHLK.M	W15(+15.9891347)
15.9949	0.9191695	Q09666	2239	2	K.VDIDAPDVDVHGPDWHLK.M	W15(+15.992878798)
15.9949	0.9191695	Q8WZ42	20856	2	R.IIGYVVDFLTEENKWQR.V	W15(+15.995918751)

15.9949	0.9191695	P11142	90	2	K.HWPFMVVNDAGRPK.V	W2(+15.99405878)
15.9949	0.9191695	Q15293	272	2	R.HWILPQDYDHAQAEAR.H	W2(+15.994427123)
15.9949	0.9191695	P06396	396	2	K.NWRDPDQTDGLGLSYLSSHIANVER.V	W2(+15.995888217)
15.9949	0.9191695	P30086	84	2	R.EWHHFLVVNMK.G	W2(+15.996797799)
15.9949	0.9191695	P31146	379	2	R.KSDLFQEDLYPPTAGPDPALTAEEWLGGR.D	W25(+15.994985042)
15.9949	0.9191695	Q05707	210	2	R.IEWHLNAFSTKDEVIEAVR.N	W3(+15.992684807)
15.9949	0.9191695	P23141	189	2	R.GNWGHLDQVAALR.W	W3(+15.993147419)
15.9949	0.9191695	Q16698	322	2	K.EQWDTIEELIR.K	W3(+15.993934315)
15.9949	0.9191695	P04083	12	2	K.QAWFIENEEQEYVQTVK.S	W3(+15.994166725)
15.9949	0.9191695	P61160	89	2	K.HLWDYTFGPEK.L	W3(+15.994479793)
15.9949	0.9191695	P30038	116	2	R.KEWDLKPIADR.A	W3(+15.99540861)
15.9949	0.9191695	Q96HC4	363	2	K.SPSWQRPNQGVPSTGR.I	W4(+15.991070491)
15.9949	0.9191695	P62820	65	2	K.LQIWDTAGQER.F	W4(+15.992196458)
15.9949	0.9191695	P33241	230	2	K.IDQWLEQYTQAIETAGR.T	W4(+15.995999273)
15.9949	0.9191695	Q15274	130	2	R.GAGWTGHVAGTR.K	W4(+15.997386241)
15.9949	0.9191695	P99999	60	2	K.GIIWGEDTLMEYLENPK.K	W4(+15.997788966)
15.9949	0.9191695	P61106	65	2	K.LQIWDTAGQER.F	W4(+15.998055833)
15.9949	0.9191695	P04792	42	2	R.LPEEWSQWLGGSSWPGYVRPLPPAAIESPAVAAPAYSR.A	W5(+15.985135889)
15.9949	0.9191695	P06732	228	2	K.SFLVWVNEEDHLR.V	W5(+15.989287123)
15.9949	0.9191695	O14773	470	2	R.VPIPWVSGTSASTPVFGGILSLINEHR.I	W5(+15.989334756)
15.9949	0.9191695	P35609	388	2	K.GYEEWLLNEIR.R	W5(+15.991967335)
15.9949	0.9191695	O75874	124	2	R.LVSGWVKPIIIGR.H	W5(+15.993392667)
15.9949	0.9191695	P15085	173	2	K.RPAIWIDTGIHSR.E	W5(+15.995415439)
15.9949	0.9191695	P61313	150	2	R.NPDTQWITKPVHK.H	W6(+15.99004166)
15.9949	0.9191695	P35749	619	2	K.FVADLWKDVDR.I	W6(+15.995266609)
15.9949	0.9191695	P05060	123	2	K.ADTEKWAEGGGHSR.E	W6(+15.995539862)
15.9949	0.9191695	P05108	126	2	R.FLIPPWVAYHQYYQR.P	W6(+15.995809655)
15.9949	0.9191695	P00352	29	2	K.IFINNEWHDSVSGK.K	W7(+15.990857526)
15.9949	0.9191695	P00738	284	2	R.VGYVSGWGR.N	W7(+15.992999315)
15.9949	0.9191695	P00846	48	2	R.LITTQQWLIK.L	W7(+15.994391907)
15.9949	0.9191695	P35609	416	2	K.ASTHETWAYGK.E	W7(+15.994622345)
15.9949	0.9191695	P00918	16	2	K.HNGPEHWHK.D	W7(+15.995023403)
15.9949	0.9191695	O43707	428	2	K.ASIHEAWTDGK.E	W7(+15.995354767)
15.9949	0.9191695	Q13907	144	2	K.AQSDGIWGEHEIDYILLVR.K	W7(+15.996859071)
15.9949	0.9191695	P00915	98	2	R.LFQFHFHWGSTNEHGSEHTVDGVK.Y	W8(+15.98932325)
15.9949	0.9191695	P04179	210	2	K.AIWNVINWENVTER.Y	W8(+15.992898262)
15.9949	0.9191695	Q9Y490	2112	2	K.VGDDPAVWQLK.N	W8(+15.993482579)
15.9949	0.9191695	Q96G03	22	2	R.LDQETAQWLR.W	W8(+15.994495142)
15.9949	0.9191695	P02763	178	2	K.SDVVYTDWKK.D	W8(+15.99603926)
15.9949	0.9191695	P02751	65	2	K.HYQINQQWER.T	W8(+15.997432846)
15.9949	0.9191695	O60234	133	2	R.TTDDLTEAWLQEK.L	W9(+15.995157666)
15.9949	0.9191695	P05093	397	2	K.GTEVIINLWALHHNEK.E	W9(+15.995247968)
15.9949	0.9191695	P53999	110	2	K.GISLNPEQWSQLK.E	W9(+16.000667077)
19.9903	0.3691468	P68371	344	2	K.NSSYFVEWIPNNVK.T	W8(+19.988511063)
19.9903	0.3691468	P14625	621	2	R.EAVEKEFEPLLNWMKDK.A	W13(+19.990672368)
19.9903	0.3691468	P12109	696	2	K.SLQWMAGGTFTGEALQYTR.D	W4(+19.993855953)
19.9903	0.3691468	P69892	38	2	R.LLVVYPWTQR.F	W7(+19.989566019)
19.9903	0.3691468	P60174	206	2	K.VVLAYEPVWAIGTGK.T	W9(+19.991290638)
31.9899	0.9376182	P01876	264	2	R.WLQGSQELPR.E	W1(+31.982762537)
31.9899	0.9376182	P62258	231	2	R.DNLTLWTSDMQGDGEEQNK.E	W6(+31.97794848)
31.9899	0.9376182	P61764	563	2	K.WEVLIGSTHILTPQK.L	W1(+31.98455823)
31.9899	0.9376182	Q6UX06	289	2	K.GLYWVAPLNTDGR.L	W4(+31.98910667)
31.9899	0.9376182	P10515	631	2	R.VVDGAVGAQWLAEFR.K	W10(+31.986143092)
31.9899	0.9376182	O75947	13	2	K.TIDWVAFAEIIPQNQK.A	W4(+31.988743128)
31.9899	0.9376182	P55072	551	2	K.GPELLTMWFGESEANVR.E	W8(+31.978152488)
31.9899	0.9376182	Q13228	409	2	R.LYITTSLYSAWDK.Q	W11(+31.988203475)
31.9899	0.9376182	P00338	250	2	K.GYTSWAIGLSVADLAESIMK.N	W5(+31.986322042)
31.9899	0.9376182	Q00610	956	2	R.KDPELWGSVLLESNPYR.R	W6(+31.987522152)
31.9899	0.9376182	Q15121	45	2	K.SEEITTGSAWFSFLESHNK.L	W10(+31.983543066)
31.9899	0.9376182	P21281	481	2	R.TVFETLDIGWQLLR.I	W10(+31.987084544)
31.9899	0.9376182	P60880	105	2	K.AWGNNQDGVVASQPAR.V	W2(+31.987444349)
31.9899	0.9376182	P06744	182	2	R.VWYVSNIDGTHIAK.T	W2(+31.987568394)
31.9899	0.9376182	P27824	83	2	R.GTLSGWILSK.A	W6(+31.989088321)
31.9899	0.9376182	P62826	64	2	K.FNVWDTAGQEK.F	W4(+31.985358137)
31.9899	0.9376182	Q01082	1500	2	R.DVEDEILWVGER.M	W8(+31.987911295)
31.9899	0.9376182	A8K7I4	85	2	K.NVAILIPETWK.T	W10(+31.98983373)
31.9899	0.9376182	P02751	1559	2	R.DLEVVAATPTSLLISWDAPAVTVR.Y	W16(+31.998330125)
31.9899	0.9376182	P56470	71	2	R.FDGWDKVVFNTLQGGK.W	W4(+31.988042245)
31.9899	0.9376182	Q15149	681	2	R.VDGAEWGVDLPSVEAQLGSHR.G	W6(+31.987870421)

31.9899	0.9376182	Q00610	514	2	K.VGYTPDWIFLLR.N	W7(+31.983543888)
31.9899	0.9376182	P13667	172	2	R.EVSQPDWTPPPEVTLVLTK.E	W7(+31.985936668)
31.9899	0.9376182	P18124	142	2	R.IVEPYIAWGYPNLK.S	W8(+31.991412709)
31.9899	0.9376182	Q9Y6R7	2501	2	R.FAVLQENVAWGNGR.V	W10(+31.9880812)
31.9899	0.9376182	O43707	664	2	R.QFASQANVVGPWIQTK.M	W12(+31.987451976)
31.9899	0.9376182	Q07021	219	2	R.EVSFQSTGESEWK.D	W12(+31.988707701)
31.9899	0.9376182	O75340	89	2	K.AGVNFSEFTGVWK.Y	W12(+31.988713012)
31.9899	0.9376182	O75874	336	2	K.GQETSTNPIASIFAWTR.G	W15(+31.979047121)
31.9899	0.9376182	Q09666	2634	2	K.VDINAPDVGVQGPDWHLK.M	W15(+31.986248081)
31.9899	0.9376182	Q09666	873	2	K.MDIDVPDVEVQGPDWHLK.M	W15(+31.988474492)
31.9899	0.9376182	P46940	742	2	R.EQLWLANEGLITR.L	W4(+31.985689591)
31.9899	0.9376182	P13591	676	2	K.SLDWNAEYEVYVVAENQQGK.S	W4(+31.986837821)
31.9899	0.9376182	P11766	14	2	K.AAVAWEAGKPLSIEEIEVAPPK.A	W5(+31.988400237)
31.9899	0.9376182	P02766	61	2	K.AADDTWEPFASGK.T	W6(+31.987502619)
31.9899	0.9376182	P98160	1806	2	K.SPAYTLVWTR.L	W8(+31.988952122)
31.9899	0.9376182	P23526	17	2	K.VADIGLAAWGR.K	W9(+31.987300062)
31.9899	0.9376182	Q9NR12	14	2	K.VVLEGPAPWGFRLQGGKDFNVPLSISR.L	W9(+31.98822079)
31.9899	0.9376182	Q8WZ42	21024	2	K.WAPPKDDGGSEITNYILEKR.D	W1(+31.987424894)
31.9899	0.9376182	P37837	296	2	R.WLHNEDQMAVEK.L	W1(+31.98841344)
31.9899	0.9376182	P27816	322	2	R.WPTETDVSSAK.N	W1(+31.98892616)
31.9899	0.9376182	P08574	276	2	R.WASEPEHDHR.K	W1(+31.989064065)
31.9899	0.9376182	P02751	1653	2	K.WLPSSSPVTGYR.V	W1(+31.989903348)
31.9899	0.9376182	P50440	210	2	K.WTTAPKPTMADELYNQDYPIHSVEDR.H	W1(+31.990677184)
31.9899	0.9376182	P46782	23	2	K.WSTDDVQINDISLQDYIAVK.E	W1(+31.990800664)
31.9899	0.9376182	Q8N8S7	23	2	K.WVPAGGSTGFSR.V	W1(+31.991479657)
31.9899	0.9376182	P19835	391	2	K.TTFDVYTESWAQDPSQENKKK.T	W10(+31.986773581)
31.9899	0.9376182	P09211	29	2	R.MLLADQGQSWK.E	W10(+31.98778271)
31.9899	0.9376182	Q99627	75	2	K.SANSELGGIWSVGQR.I	W10(+31.988933036)
31.9899	0.9376182	Q13418	208	2	K.LNENHSGELWK.G	W10(+31.989291834)
31.9899	0.9376182	Q9H074	334	2	K.LTGSVLEDAWK.E	W10(+31.98966654)
31.9899	0.9376182	P55268	594	2	R.LVTPGETPSWTGSGFVR.L	W10(+31.990089152)
31.9899	0.9376182	Q9BSJ8	845	2	K.TISQTSAPVWDESASFLIR.K	W10(+31.990339212)
31.9899	0.9376182	P31327	247	2	K.RGAEVHLVPWNHDFTK.M	W10(+31.990704254)
31.9899	0.9376182	P11586	530	2	R.LDIDPETITWQR.V	W10(+31.99104179)
31.9899	0.9376182	Q07954	4046	2	R.ETLVQDNIQWPTGLAVDYHNER.L	W10(+31.992851496)
31.9899	0.9376182	P04746	149	2	R.DFPAVPYSGWDFNDGK.C	W10(+31.993172144)
31.9899	0.9376182	P29401	257	2	R.GITGVEDKESWHGKPLPK.N	W11(+31.985060969)
31.9899	0.9376182	Q9NZU5	120	2	K.DPTFDTITYEWAPPGVTQK.L	W11(+31.985972665)
31.9899	0.9376182	Q8WZ42	6664	2	K.VAGTPELSVEWYK.D	W11(+31.98697747)
31.9899	0.9376182	P37802	150	2	R.DDGLFSGDPNWFPK.K	W11(+31.987388234)
31.9899	0.9376182	Q9UK22	279	2	R.FEHGGQDSVYWK.G	W11(+31.987516211)
31.9899	0.9376182	Q02880	582	2	K.GLLINFIHHNWPSLLK.H	W11(+31.988172899)
31.9899	0.9376182	P50552	82	2	K.YNQATPNFHQWR.D	W11(+31.988823668)
31.9899	0.9376182	Q86UX7	16	2	K.TASGDYIDSSWELR.V	W11(+31.988912649)
31.9899	0.9376182	Q13200	154	2	R.LVGSQEELASWGHEYVR.H	W11(+31.989491497)
31.9899	0.9376182	P14314	449	2	K.LGGSPTSLGTWGSWIGPDHDK.F	W11(+31.990880876)
31.9899	0.9376182	P78352	717	2	R.VIEDLSGPYIWVPAR.E	W11(+31.991234935)
31.9899	0.9376182	P05093	121	2	K.GIAFADSGAHWQLHR.R	W11(+31.991320508)
31.9899	0.9376182	Q8WZ42	1627	2	K.IDSTVSQDSAWYTATAINK.A	W11(+31.992038495)
31.9899	0.9376182	P04746	424	2	R.NVVDGQPFTNWYDNGSNQVAFGR.G	W11(+31.994462295)
31.9899	0.9376182	Q9UH99	582	2	K.TALLSLFGIPLWYHSQSPR.V	W12(+31.986823947)
31.9899	0.9376182	P15085	367	2	K.AIYQASGSTIDWTYSQGIK.Y	W12(+31.987860644)
31.9899	0.9376182	P08473	408	2	K.ALYGTTSETATWR.R	W12(+31.988309366)
31.9899	0.9376182	P02675	470	2	K.HGTDDGVVWMNWK.G	W12(+31.98832323)
31.9899	0.9376182	O75112	263	2	K.SQNKPEDEADEWAR.R	W12(+31.988431762)
31.9899	0.9376182	P01889	84	2	R.APWIEQEGPEYWDR.N	W12(+31.988565332)
31.9899	0.9376182	Q06323	134	2	K.DVIEQLNLVTTWLQLQIPR.I	W12(+31.988705718)
31.9899	0.9376182	P01619	56	2	R.ASQSVSSSYLAWYQQKPGQAPR.L	W12(+31.988794825)
31.9899	0.9376182	Q86UP2	1094	2	K.VSVPSNLSYGEWLHGFEK.K	W12(+31.989487055)
31.9899	0.9376182	P18206	258	2	R.VLQLTSWDEDAWASK.D	W12(+31.989556913)
31.9899	0.9376182	Q03252	501	2	K.NNSDKDQSLGNWR.I	W12(+31.989714425)
31.9899	0.9376182	Q14103	97	2	R.HSEAATAQREEWK.M	W12(+31.989727365)
31.9899	0.9376182	P27824	304	2	K.IPDPEAVKPDDWDEDAPAK.I	W12(+31.990406987)
31.9899	0.9376182	P19971	315	2	R.DLVTTLGGALLWLSGHAGTQAQGAAR.V	W12(+31.990475844)
31.9899	0.9376182	P12814	645	2	K.QFGAQANVIGPWIQTK.M	W12(+31.99091152)
31.9899	0.9376182	P02790	398	2	K.SGAQATWTELPWPHEK.V	W12(+31.991006742)
31.9899	0.9376182	P35613	255	2	K.SESVPPVTDWAWYK.I	W12(+31.992756077)
31.9899	0.9376182	P19835	247	2	R.AISQSGVALSPWVIQK.N	W12(+31.993372357)
31.9899	0.9376182	P26038	168	2	R.VLEQHKLNKDQWEER.I	W12(+31.993844206)

31.9899	0.9376182	Q14108	146	2	R.TLNIPVLTVIEWSQVHFLR.E	W12(+31.995813516)
31.9899	0.9376182	P02751	1925	2	R.VTDATETTITISWR.T	W13(+31.986509568)
31.9899	0.9376182	P20674	68	2	R.WVTYFNKPDIDAWELRK.G	W13(+31.987245269)
31.9899	0.9376182	O14818	156	2	R.LYQTDPSGTYHAWK.A	W13(+31.989427462)
31.9899	0.9376182	P11216	183	2	K.IVNGWQVEEADDWLR.Y	W13(+31.989660191)
31.9899	0.9376182	P18669	78	2	R.TLWTVLDAIDQMWLPVVR.T	W13(+31.98976156)
31.9899	0.9376182	P98088	1065	2	R.SVVGDVLEFGNSWK.L	W13(+31.989796023)
31.9899	0.9376182	P22234	214	2	K.EIVLADVIDNDSWR.L	W13(+31.990136897)
31.9899	0.9376182	Q08211	1087	2	K.VQSDGQIVLVDDWIK.L	W13(+31.990621928)
31.9899	0.9376182	Q16891	384	2	R.ELDSITPEVLPGWK.G	W13(+31.990884609)
31.9899	0.9376182	P31323	245	2	R.AATITATSPGALWGLDR.V	W13(+31.991132354)
31.9899	0.9376182	Q13630	38	2	K.VVADGAGLPGEDWVFVSSK.D	W13(+31.994685394)
31.9899	0.9376182	P14060	231	2	K.FSTVNPVYVGNVAWAHILALR.A	W14(+31.987981585)
31.9899	0.9376182	Q9HBL0	1197	2	R.TVGTNTPPSPGFGWR.A	W14(+31.988684755)
31.9899	0.9376182	P04114	1981	2	K.VSALLTPAEQTGTWK.L	W14(+31.989106066)
31.9899	0.9376182	P19652	140	2	K.TLMFGSYLDDEKNWGLSFYADKPETTK.E	W14(+31.996571008)
31.9899	0.9376182	Q14204	824	2	K.EVQALIAEGIALVWESYK.L	W14(+31.998134256)
31.9899	0.9376182	Q09666	1378	2	K.VDISAPDVDVHGPDWHLK.M	W15(+31.987976525)
31.9899	0.9376182	Q09666	4056	2	K.VDIDAPDVDVHGPDWHLK.M	W15(+31.988423231)
31.9899	0.9376182	Q09666	4312	2	K.VDIDAPDVDVHGPDWHLK.M	W15(+31.989288949)
31.9899	0.9376182	O76062	58	2	R.LLGPPASLPGLEVLWSPR.A	W15(+31.991351725)
31.9899	0.9376182	P04179	149	2	K.LTAASVGVQGSGWGWLGFNK.E	W15(+31.991467058)
31.9899	0.9376182	P00450	454	2	R.GPEEEHLGILGPVIWAEVGDTIR.V	W15(+31.995751949)
31.9899	0.9376182	P21549	288	2	R.ESLALIAEQGLENSWR.Q	W15(+31.997769883)
31.9899	0.9376182	O95954	462	2	R.AVSVPLTLAETVASLWPALQELAR.C	W16(+31.98787807)
31.9899	0.9376182	O95674	327	2	R.LQEYNIPGVIQSVIGWK.T	W16(+31.989340513)
31.9899	0.9376182	P12036	292	2	R.AQLEGHAVQSTLQSEEWFR.V	W17(+31.988011382)
31.9899	0.9376182	Q8WZ42	4101	2	R.ALQAAVASEQPGLFSEWLR.N	W17(+31.988220434)
31.9899	0.9376182	P07585	337	2	K.KASYSGVSLFSNPVQYWEIQPSTFR.C	W17(+31.993390451)
31.9899	0.9376182	P00918	244	2	K.LNFNGEGEPEELMVDNWR.P	W17(+31.995002059)
31.9899	0.9376182	Q13228	191	2	K.GTWERPGGAAPLGYDFWYQPR.H	W17(+31.995260713)
31.9899	0.9376182	O43852	96	2	K.IDGDKDGFVTVDELKDWIK.F	W17(+31.998197415)
31.9899	0.9376182	P42167	233	2	R.RVEHNQSYSQAGITETEWTSGSSK.G	W18(+31.985677641)
31.9899	0.9376182	P61026	195	2	K.EPNSENVDISSGGGVTGWK.S	W18(+31.990583059)
31.9899	0.9376182	P14317	21	2	K.SVVGHDVSVSVETQGDDWDTDPDFVNDISEK.E	W18(+31.990673976)
31.9899	0.9376182	O95373	339	2	R.VLQQTLNYINQGVSHALTWK.N	W19(+31.986600186)
31.9899	0.9376182	Q08211	139	2	K.AENNSEVGASGYGVPGPTWDR.G	W19(+31.991469777)
31.9899	0.9376182	P18206	283	2	K.GWLRDPSASPGDAGEQAIR.Q	W2(+31.987369774)
31.9899	0.9376182	P30086	84	2	R.EWHHFLVVNMK.G	W2(+31.988863228)
31.9899	0.9376182	Q15334	460	2	R.FWDASGVALRPLYK.L	W2(+31.990103858)
31.9899	0.9376182	Q92896	557	2	R.DWKLDPVLYR.K	W2(+31.990471209)
31.9899	0.9376182	P51911	37	2	R.EWIEGVTGR.R	W2(+31.99048006)
31.9899	0.9376182	P84098	23	2	K.VWLDPNETNEIANANSR.Q	W2(+31.990962861)
31.9899	0.9376182	Q8IZ83	775	2	R.AWDQEAEGAGPELGLR.V	W2(+31.991174524)
31.9899	0.9376182	Q9Y262	222	2	K.IWNVHSVLNVLHSLVDK.S	W2(+31.991461729)
31.9899	0.9376182	Q13509	21	2	K.FWEVISDEHGIDPSGNYVGDSDLQLER.I	W2(+31.99502191)
31.9899	0.9376182	P14550	220	2	R.AWRDPDEPVLLEEPVVLALAEK.Y	W2(+31.998687462)
31.9899	0.9376182	Q9GZT8	140	2	R.VGIYSPHTAYDAAPQGVNNWLAK.G	W20(+31.986379645)
31.9899	0.9376182	Q6P2Q9	816	2	K.DGPYITAEEAVAVYTTTVHWLESR.R	W20(+31.988476093)
31.9899	0.9376182	Q6STE5	226	2	K.SLVIELDKDLYGPDNHLVEWHR.T	W20(+31.99139223)
31.9899	0.9376182	P12883	593	2	K.GKPEAHFSLIHYAGIVDYNIIGWLQK.N	W23(+31.988314861)
31.9899	0.9376182	P61158	76	2	K.GVDDLDFFIGDEAIEKPTYATKWPIR.H	W23(+31.99393029)
31.9899	0.9376182	O15230	2550	2	R.ILQAVQAAEDAAGQALQQADHTWATVVR.Q	W23(+31.997584556)
31.9899	0.9376182	O00571	421	2	K.VVWVEESDKR.S	W3(+31.987045282)
31.9899	0.9376182	Q8WZ42	19966	2	R.GDWVTALASVTK.T	W3(+31.987775641)
31.9899	0.9376182	Q9Y281	135	2	K.HEWQVNGLDDIKDR.S	W3(+31.988813918)
31.9899	0.9376182	P39060	1691	2	K.SVWHGSDPNGR.R	W3(+31.989027373)
31.9899	0.9376182	P62837	93	2	R.SQWSPALTISK.V	W3(+31.989062984)
31.9899	0.9376182	P47756	75	2	R.SPWSNKYDPPLEDGAMPSAR.L	W3(+31.989263566)
31.9899	0.9376182	Q05707	849	2	R.ITWDPPSSPVK.G	W3(+31.989586045)
31.9899	0.9376182	Q9Y6Y8	882	2	K.SAWQTLNEFAR.A	W3(+31.990187902)
31.9899	0.9376182	Q05707	658	2	K.LMWIPVYGGK.T	W3(+31.990902542)
31.9899	0.9376182	P02675	448	2	R.YYWGGQYTWDMAK.H	W3(+31.991327779)
31.9899	0.9376182	Q99715	1689	2	R.ITWAPFGSSDK.M	W3(+31.991370222)
31.9899	0.9376182	Q8WZ42	17564	2	K.LTWFSPEDDGGSPITNYVIEKR.E	W3(+31.992579879)
31.9899	0.9376182	P31749	99	2	R.EEWTTAIQTVADGLK.K	W3(+31.99264345)
31.9899	0.9376182	Q14204	1470	2	R.EVWNTYELDLVNYQNK.C	W3(+31.99285848)
31.9899	0.9376182	Q15029	733	2	R.SIWAFGPDATGPNILVDDTLPSEVDKALLGSVK.D	W3(+32.007232177)
31.9899	0.9376182	Q15019	260	2	R.LYPWGVVEVENPEHNDFLK.L	W4(+31.980474841)

31.9899	0.9376182	P02787	147	2	R.SAGWNIPIGLLYCDLPEPR.K	W4(+31.980812103)
31.9899	0.9376182	P37837	196	2	R.ILDWHVANTDKK.S	W4(+31.98676227)
31.9899	0.9376182	P20336	76	2	K.LQIWDTAGQER.Y	W4(+31.988534348)
31.9899	0.9376182	P11137	14	2	K.APHWTSAPLTEASAHSHPPEIK.D	W4(+31.988644421)
31.9899	0.9376182	P63151	203	2	R.INLWHLEITDR.S	W4(+31.989104441)
31.9899	0.9376182	P07942	583	2	R.IPSWTGAGFVR.V	W4(+31.989350193)
31.9899	0.9376182	P04792	16	2	R.GPSWDPFR.D	W4(+31.989910394)
31.9899	0.9376182	O00154	161	2	K.ATLWYVPLSLK.N	W4(+31.991105724)
31.9899	0.9376182	Q9Y490	2295	2	K.GTEWVDPEDPTVIAENELLGAAAAIEAAAK.K	W4(+31.991181097)
31.9899	0.9376182	P17858	70	2	K.QANWLSVSNIIQLGGTIIGSAR.C	W4(+31.996033537)
31.9899	0.9376182	Q5SZK8	643	2	R.TVTEWQQQDITEGR.L	W5(+31.957075332)
31.9899	0.9376182	P26038	217	2	K.GSELWLGVDALGLNIYEQNDR.L	W5(+31.988285035)
31.9899	0.9376182	Q71U36	407	2	R.AFVHWYVGEGMEEGEFSEAR.E	W5(+31.988624926)
31.9899	0.9376182	Q02252	93	2	R.AFPAWADTSVLSR.Q	W5(+31.988922373)
31.9899	0.9376182	P06312	76	2	K.LLIYWASTR.E	W5(+31.988947982)
31.9899	0.9376182	Q03252	554	2	K.GQSSWGTGESFR.T	W5(+31.989106375)
31.9899	0.9376182	Q9Y490	1630	2	R.DPPSWSVLAGHSR.T	W5(+31.989555589)
31.9899	0.9376182	O94875	900	2	K.IDQNWYEGEHHGR.V	W5(+31.99079328)
31.9899	0.9376182	Q7Z6K5	24	2	R.LPGAWDPAAHQGGNGVLLEGELIDVSR.H	W5(+31.991711611)
31.9899	0.9376182	P11216	175	2	K.IVNGWQVEEADDWLR.Y	W5(+31.992101598)
31.9899	0.9376182	P15311	217	2	K.GTDLWLGVDALGLNIYEKDDKLTPK.I	W5(+31.994218674)
31.9899	0.9376182	P35241	217	2	K.GTELWLGVDALGLNIYEHDDKLTPK.I	W5(+32.007345357)
31.9899	0.9376182	Q15404	218	2	K.AENNPWVTPIADQFQLGVSHVFEYIR.S	W6(+31.983403719)
31.9899	0.9376182	P09960	118	2	K.SSALQWLTPEQTSGK.E	W6(+31.987451351)
31.9899	0.9376182	P35222	25	2	K.AAVSHWQQQSYLDSGIHSGATTTAPSLSGK.G	W6(+31.987835736)
31.9899	0.9376182	P51659	116	2	R.ISDEDWDIIHR.V	W6(+31.98862647)
31.9899	0.9376182	P62873	63	2	K.IYAMHWGTDSR.L	W6(+31.989009047)
31.9899	0.9376182	P61313	120	2	R.VLNSYWVGEDSTYK.F	W6(+31.989132888)
31.9899	0.9376182	Q9P258	11	2	K.AAAAAWEEPSSGNGTAR.A	W6(+31.989653088)
31.9899	0.9376182	P49588	590	2	K.VGDQVWLFIDEPR.R	W6(+31.992951583)
31.9899	0.9376182	P78527	3805	2	R.LGLIEWLENTVTLK.D	W6(+31.995648897)
31.9899	0.9376182	Q9UQC9	166	2	R.VFVHEWAHLR.W	W6(+32.002079748)
31.9899	0.9376182	P63208	88	2	R.TDDIPVWDQEFLK.V	W7(+31.987134047)
31.9899	0.9376182	P06737	826	2	K.EYAQNIWNVEPSDLK.I	W7(+31.987136158)
31.9899	0.9376182	Q8WZ42	32069	2	R.DSVNLTWTEPASDGGSKITNYIVEK.C	W7(+31.988301239)
31.9899	0.9376182	Q8WZ42	17368	2	K.SSADLEWSPPLKDGGSK.V	W7(+31.988301999)
31.9899	0.9376182	Q16643	29	2	R.EESAADWALYTYEDGSDDLK.L	W7(+31.988375738)
31.9899	0.9376182	P00918	16	2	K.HNGPEHWHK.D	W7(+31.988414375)
31.9899	0.9376182	Q8WZ42	21221	2	R.NSVTLIWTEPK.Y	W7(+31.988457935)
31.9899	0.9376182	P07741	98	2	K.LPGPTLWASYSLEYGK.A	W7(+31.988475953)
31.9899	0.9376182	Q8WZ42	29715	2	R.ESGTTAWQLVNSSVKR.T	W7(+31.988782379)
31.9899	0.9376182	Q96NY7	328	2	R.SPGELAWDAAEEAEVPGVKGSEEAAPGDAR.A	W7(+31.98898743)
31.9899	0.9376182	P09104	365	2	K.LAQENGWGVMVSHR.S	W7(+31.98929008)
31.9899	0.9376182	Q99627	153	2	K.GILEQGWQADSTTR.M	W7(+31.989292452)
31.9899	0.9376182	P18124	209	2	K.EANNFLWPFK.L	W7(+31.990539036)
31.9899	0.9376182	Q8WZ42	15777	2	K.TKPDSEWIVVTSTLR.H	W7(+31.990562084)
31.9899	0.9376182	Q14315	551	2	K.YVVTITWGGYAIPR.S	W7(+31.991616767)
31.9899	0.9376182	Q8WZ42	27225	2	K.ESAVLSWDVPENDGGAPVK.N	W7(+31.991621266)
31.9899	0.9376182	P35611	161	2	R.LADLFGWSQLIYNHITTR.V	W7(+31.991740377)
31.9899	0.9376182	Q8WZ42	27715	2	K.NSVSLSWQQPAFDGGSK.I	W7(+31.993098172)
31.9899	0.9376182	P08237	453	2	K.GQIEEAGWSYVGGWTGQGGSK.L	W8(+31.986801901)
31.9899	0.9376182	Q13683	606	2	K.HQASGTVWLK.H	W8(+31.987203056)
31.9899	0.9376182	Q15149	668	2	R.YLQDLLAWVEENQHR.V	W8(+31.987524517)
31.9899	0.9376182	Q14103	106	2	K.MFIGGLSWDTTK.K	W8(+31.988677284)
31.9899	0.9376182	P17661	295	2	K.NISEAEEWYK.S	W8(+31.98880409)
31.9899	0.9376182	P20916	196	2	R.LREDEGTWVQVSLLHFVPTR.E	W8(+31.988940173)
31.9899	0.9376182	O15061	76	2	R.QQLDELSWATALAEGER.D	W8(+31.989273307)
31.9899	0.9376182	Q9Y2B0	40	2	R.ALVDELEWEIAQVDPKK.T	W8(+31.989475817)
31.9899	0.9376182	Q13813	1248	2	R.DADETKEWIEEKNQALNTDNYGHDLASVQALQR.K	W8(+31.989659535)
31.9899	0.9376182	Q15020	562	2	R.TEGSLEDWDIAVQK.T	W8(+31.989726728)
31.9899	0.9376182	Q12860	624	2	R.ATSVALTWSR.G	W8(+31.989759737)
31.9899	0.9376182	Q9BTW9	959	2	R.SDVASVNWSAPSQAFPR.I	W8(+31.990016687)
31.9899	0.9376182	Q96G03	22	2	R.LDQETAQWLR.W	W8(+31.990710963)
31.9899	0.9376182	P09455	9	2	M.PVDFTGYWK.M	W8(+31.991151418)
31.9899	0.9376182	Q02978	181	2	R.EEGVLTLWR.G	W8(+31.991478436)
31.9899	0.9376182	Q9Y490	2112	2	K.VGDDPAVWQLK.N	W8(+31.991773595)
31.9899	0.9376182	Q14204	2500	2	R.YLVYAILWSLSGDSR.L	W8(+31.992780274)
31.9899	0.9376182	P00918	97	2	R.LIQFHFHWGSLDGQGSEHTVDK.K	W8(+31.993093542)
31.9899	0.9376182	O00410	156	2	R.EAALHIFWNFPGIFGNQQQHYLDVIK.R	W8(+31.993867262)

31.9899	0.9376182	P52179	1515	2	K.TGVTGEQIWLQINEPTPNDKGK.Y	W9(+31.988217207)
31.9899	0.9376182	Q9Y490	173	2	K.LHTDDELNWLDHGR.T	W9(+31.988680941)
31.9899	0.9376182	Q14204	1523	2	K.VFEEDALSWEDK.L	W9(+31.988696765)
31.9899	0.9376182	P19237	161	2	K.ERPVEVGDWRK.N	W9(+31.988900713)
31.9899	0.9376182	P04181	178	2	K.IVFAAGNFWGR.T	W9(+31.989356989)
31.9899	0.9376182	Q01082	396	2	K.LISDINKAWER.L	W9(+31.989591531)
31.9899	0.9376182	Q8WZ42	5446	2	K.GSLPITVTWLKDSDEITEDDNIR.M	W9(+31.989648426)
31.9899	0.9376182	P08648	447	2	K.PSQVLQPLWAASHTPDFFGSALR.G	W9(+31.990339569)
31.9899	0.9376182	O15230	2360	2	R.VQEQLSSLWEENQALATQTR.D	W9(+31.991932377)
31.9899	0.9376182	Q6P2Q9	293	2	R.DINLQDEDWNEFNDINK.I	W9(+31.993363595)
31.9899	0.9376182	Q13228	204	2	R.HNVMISTEWAAPNVLR.D	W9(+31.996365764)
31.9899	0.9376182	Q92625	704	2	R.TLEQSVGEWLESIGLQQYESK.L	W9(+31.996636735)
67.0058	0.3471842	P02763	43	2	K.WFYIASAFR.N	W1(+67.00468975)
67.0058	0.3471842	P07108	56	2	K.WDAWNELK.G	W1(+67.006206479)
115.9754	0.1090560	P04440	88	2	R.AVTELGRPAAEYWNSQKDILEEKR.A	W13(+115.972319512)
209.0179	0.7899121	Q13554	238	2	K.AGAYDFPSPEWDTVTPEAK.N	W11(+209.018237257)
209.0179	0.7899121	P06733	301	2	K.SFIKDYPVVSIEDPFDQDDWGAWQK.F	W20(+209.024018904)
209.0179	0.7899121	P07437	101	2	R.SGPFGQIFRPDNFVFGQSGAGNNWAK.G	W24(+209.017806337)
209.0179	0.7899121	P07900	320	2	K.SLTNDWEDHLAVK.H	W6(+209.018944601)
354.1708	0.0515655	P09172	431	2	R.EWEIVNQDNHYSPHFQEIR.M	W2(+354.162063598)
32.9748	0.6928963	Q9Y6R7	3702	2	R.FAVLQENVAWGNGR.V	W10(+32.975752098)
32.9748	0.6928963	P69892	131	2	K.EFTPEVQASWQK.M	W10(+32.973737283)
32.9748	0.6928963	Q9Y6R7	1300	2	R.FAVLQENVAWGNGR.V	W10(+32.973921043)
14.9827	0.2716954	P13637	896	2	R.TVNDLEDSYGQQWTYEQR.K	W13(+14.974317958)
14.9827	0.2716954	P39019	52	2	K.HKELAPYDENWFYTR.A	W11(+14.972655259)
14.9827	0.2716954	Q13813	2220	2	R.QEFAQHANAFHQWIQETR.T	W13(+14.971212001)
14.9827	0.2716954	P27695	119	2	K.LPAELQELPGLSHQYWSAPSDK.E	W16(+14.971240242)
14.9827	0.2716954	P07197	291	2	R.SQLESHSDQNMHQAEEWFK.C	W17(+14.975426042)
14.9827	0.2716954	P69905	15	2	K.AAWGKVGAHAGEYGAEALER.M	W3(+14.975470591)
14.9827	0.2716954	P31327	152	2	K.SLGQWLQEEKVPAIYGVDTR.M	W5(+14.977297791)
14.9827	0.2716954	P08238	312	2	K.SLTNDWEDHLAVK.H	W6(+14.977684836)
14.9827	0.2716954	P06733	365	2	K.LAQANGWGVMVSHR.S	W7(+14.970795859)
14.9827	0.2716954	Q8WZ42	18283	2	R.DVASAQWSPLSATSK.K	W7(+14.973320671)
14.9827	0.2716954	P02787	460	2	K.SASDLTWDNLK.G	W7(+14.976171004)
14.9827	0.2716954	Q01082	1713	2	R.EVDDLEQWIAER.E	W8(+14.971355448)
14.9827	0.2716954	P07451	47	2	R.HDPSLQPWSVSYDGGSAK.T	W8(+14.972910583)
14.9827	0.2716954	P12236	71	2	K.EQGVLSFWR.G	W8(+14.975055868)
14.9827	0.2716954	P68371	21	2	K.FWEVISDEHGIDPTGTYHGDSDLQLER.I	W2(+14.979043668)
14.9827	0.2716954	P08758	187	2	K.WGTDEEKFITIFGTR.S	W1(+14.985227833)
14.9827	0.2716954	P01857	264	2	K.GFYPSDIAVEWESNGQPENNYK.T	W11(+14.98341958)
14.9827	0.2716954	Q9Y5U9	33	2	K.NIGWGTDQGIGGFGEEPGIK.S	W4(+14.983536641)
14.9827	0.2716954	P00338	227	2	K.TLHPDLGTDKDKEQWK.E	W15(+14.982798571)
14.9827	0.2716954	P07437	344	2	K.NSSYFVEWIPNNVK.T	W8(+14.979966142)
14.9827	0.2716954	P13639	641	2	R.YLAEKYEWDVAEAR.K	W8(+14.981811052)
14.9827	0.2716954	P68871	16	2	K.SAVTALWGKVNVDEVGGEALGR.L	W7(+14.9786692)
-124.1118	0.2696443	Q8WZ42	19475	1	K.IADASPDEGWKR.C	W10(-124.11038418)
-124.1118	0.2696443	P22695	149	1	R.RWEVADLQPQLK.I	W2(-124.108781623)
-124.1118	0.2696443	P19367	171	1	K.IDEAILITWTKR.F	W9(-124.110740381)
-59.0506	0.1389893	P63261	86	1	K.IWHHTFYNELR.V	W2(-59.055459596)
-59.0506	0.1389893	Q01082	726	1	R.EQWANLEQLSAIR.K	W3(-59.052666714)
-59.0506	0.1389893	P08238	312	1	K.SLTNDWEDHLAVK.H	W6(-59.052344461)
-13.0308	0.3243439	P01857	196	1	R.VVSVLTVLHQDWLNGK.E	W12(-13.028372667)
-13.0308	0.3243439	P15814	186	1	K.YAASSYLSLTPEQWR.S	W14(-13.031554456)
-13.0308	0.3243439	P25311	168	1	K.QKWEAEPVYVQR.A	W3(-13.032870399)
-13.0308	0.3243439	Q86V81	87	1	K.QLPDKWQHDLFDSGFGGGAGVETGGK.L	W6(-13.033189865)
3.9953	0.9130741	P18669	167	1	R.ALPFWNEEIVPQIK.E	W5(+3.992654063)
3.9953	0.9130741	P61764	288	1	K.EVLLDEDDDLWIALR.H	W11(+3.988005668)
3.9953	0.9130741	P14618	515	1	K.KGDVVIVLTGWR.P	W11(+3.992145734)
3.9953	0.9130741	Q9H115	275	1	R.LDQWLTTMLLR.I	W4(+3.99111252)
3.9953	0.9130741	P56470	71	1	R.FDGWDKVVFNTLQGGK.W	W4(+3.991032968)
3.9953	0.9130741	Q01082	1077	1	R.DLDDFQSWLSR.T	W8(+3.988485977)
3.9953	0.9130741	Q9C002	69	1	K.LITINQQWKPIEELQNVQR.V	W8(+3.989683502)
3.9953	0.9130741	Q01082	726	1	R.EQWANLEQLSAIR.K	W3(+3.994452427)
3.9953	0.9130741	Q13509	21	1	K.FWEVISDEHGIDPSGNYVGDSDLQLER.I	W2(+3.989040465)
3.9953	0.9130741	O75874	124	1	R.LVSGWVKPIIIGR.H	W5(+3.992294034)
3.9953	0.9130741	Q9UBB6	402	1	R.ILGAWLAEETSSLR.K	W5(+3.994202653)
3.9953	0.9130741	Q00610	164	1	K.WLLLTGISAQQNR.V	W1(+3.992232752)
3.9953	0.9130741	Q01813	469	1	K.EIGWTDVGGWTGQGGSILGTK.R	W10(+3.992918038)
3.9953	0.9130741	P02751	1833	1	K.FTQVTPTSLSAQWTPPNVQLTGYR.V	W13(+4.002824946)

3.9953	0.9130741	P52565	194	1	R.FTDDDKTDHLSWEWNLTIK.K	W14(+3.99056419)
3.9953	0.9130741	P0DOY2	79	1	K.YAASSYLSLTPEQWK.S	W14(+3.993026172)
3.9953	0.9130741	P25325	15	1	R.ALVSAQWVAEALR.A	W7(+3.994516959)
3.9953	0.9130741	Q13813	2106	1	K.ASAFNSWFENAEEDLTDPVR.C	W7(+3.996459562)
3.9953	0.9130741	P27797	33	1	K.EQFLDGDGWTSR.W	W9(+3.993073359)
3.9953	0.9130741	P46782	23	1	K.WSTDDVQINDISLQDYIAVK.E	W1(+3.991288945)
3.9953	0.9130741	P39656	345	1	K.WVPFDGDDIQLEFVR.I	W1(+3.991466705)
3.9953	0.9130741	P00558	383	1	K.WNTEDKVSHVSTGGGASLELLEGK.V	W1(+3.991609201)
3.9953	0.9130741	Q8IXS6	52	1	K.WLLQGIPAGTAEEEEAR.R	W1(+3.992650612)
3.9953	0.9130741	P05093	313	1	K.WTLAFLLHNPQVK.K	W1(+3.993482906)
3.9953	0.9130741	P55072	454	1	R.WALSQSNPSALR.E	W1(+3.994131314)
3.9953	0.9130741	Q9UH65	467	1	K.WHLEQQQAIQTTEAEKQELENQR.V	W1(+3.996445216)
3.9953	0.9130741	P04439	268	1	K.WAAVVVPSGEEQR.Y	W1(+3.996902659)
3.9953	0.9130741	P10321	268	1	K.WAAVVVPSGQEQR.Y	W1(+3.996909454)
3.9953	0.9130741	Q99798	657	1	R.WVVIGDENYGEGSSR.E	W1(+3.996996737)
3.9953	0.9130741	P08263	21	1	R.WLLAAAGVEFEEK.F	W1(+3.998005373)
3.9953	0.9130741	P35573	680	1	K.WNPEALPSNTGEVNFQSGIIAAR.C	W1(+3.998420998)
3.9953	0.9130741	P25786	13	1	R.NQYDNDVTVWSPQGR.I	W10(+3.990722243)
3.9953	0.9130741	P35221	705	1	K.SKLDAEVSKWDDSGNDIIVLAK.Q	W10(+3.99088061)
3.9953	0.9130741	Q9UKV3	1044	1	R.TGTLVEEAFWIDK.I	W10(+3.992770205)
3.9953	0.9130741	P09211	29	1	R.MLLADQGQSWK.E	W10(+3.993764155)
3.9953	0.9130741	Q15124	364	1	K.VPVYETPAGWR.F	W10(+3.993811782)
3.9953	0.9130741	P06396	71	1	K.AGKEPGLQIWR.V	W10(+3.994796332)
3.9953	0.9130741	P35613	253	1	K.SESVPPVTDWAWYK.I	W10(+3.995672478)
3.9953	0.9130741	Q13418	208	1	K.LNENHSGELWK.G	W10(+3.996005702)
3.9953	0.9130741	Q14204	702	1	K.LNTQEIFDDWAR.K	W10(+3.996209157)
3.9953	0.9130741	A0AVT1	192	1	K.FISADVHGIWSR.L	W10(+3.996209734)
3.9953	0.9130741	P12110	702	1	K.NLEWIAGGTWTPSALK.F	W10(+3.996432668)
3.9953	0.9130741	P26439	345	1	R.DLAYKPLYSWEEAK.Q	W10(+3.996642566)
3.9953	0.9130741	P27824	455	1	R.IVDDWANDGWGLK.K	W10(+3.997017933)
3.9953	0.9130741	P45877	212	1	K.TPFVVEIADW.-	W10(+3.997315667)
3.9953	0.9130741	P08575	1125	1	R.TVYQYQYTNWSVEQLPAEPK.E	W10(+3.998472825)
3.9953	0.9130741	P21281	481	1	R.TVFETLDIGWQLLR.I	W10(+3.998559154)
3.9953	0.9130741	P15085	191	1	R.EWVTQASGVWFAK.K	W10(+3.998637869)
3.9953	0.9130741	P05451	99	1	K.ESGTDDFNVWIGLHDPKK.N	W10(+3.99987895)
3.9953	0.9130741	Q9H8H3	231	1	K.LQHIQAPLSWELVRPHIYGYAVK.-	W10(+4.000571959)
3.9953	0.9130741	Q07954	4046	1	R.ETLVQDNIQWPTGLAVDYHNER.L	W10(+4.000786066)
3.9953	0.9130741	Q8WZ42	6664	1	K.VAGTPELSVEWYK.D	W11(+3.993447196)
3.9953	0.9130741	Q8WZ42	9210	1	R.VAGSQPITVAWYK.N	W11(+3.993928418)
3.9953	0.9130741	P00751	334	1	K.VSEADSSNADWVTK.Q	W11(+3.994856932)
3.9953	0.9130741	P50552	82	1	K.YNQATPNFHQWR.D	W11(+3.995097003)
3.9953	0.9130741	Q8WZ42	1589	1	R.ATGNPNPDIVWLK.N	W11(+3.99509783)
3.9953	0.9130741	P62736	81	1	K.YPIEHGIITNWDDMEK.I	W11(+3.995113466)
3.9953	0.9130741	Q8WZ42	4524	1	K.DGAALSPSPNWR.I	W11(+3.995731194)
3.9953	0.9130741	Q01082	2119	1	K.VSEEAESQQQWDTSKGEQVSQNGLPAEQGSPR.M	W11(+3.996802135)
3.9953	0.9130741	P01624	55	1	R.ASQSVSSNLAWYQQKPGQAPR.L	W11(+3.997584189)
3.9953	0.9130741	P04746	424	1	R.NVVDGQPFTNWYDNGSNQVAFGR.G	W11(+3.998368545)
3.9953	0.9130741	Q13200	154	1	R.LVGSQEELASWGHEYVR.H	W11(+3.999440228)
3.9953	0.9130741	Q07021	219	1	R.EVSFQSTGESEWK.D	W12(+3.993346373)
3.9953	0.9130741	Q96D96	38	1	R.HFTVVGDDYHAWNINYK.K	W12(+3.995235598)
3.9953	0.9130741	Q15181	189	1	R.LKPGYLEATVDWFR.R	W12(+3.995402708)
3.9953	0.9130741	Q9H2U2	237	1	K.FKPGYLEATLNWFR.L	W12(+3.995409503)
3.9953	0.9130741	Q06323	134	1	K.DVIEQLNLVTTWLQLQIPR.I	W12(+3.995493907)
3.9953	0.9130741	P12814	645	1	K.QFGAQANVIGPWIQTK.M	W12(+3.995550192)
3.9953	0.9130741	O75112	263	1	K.SQNKPEDEADEWAR.R	W12(+3.995817016)
3.9953	0.9130741	Q92777	126	1	K.VLLVVDEPHADWAK.C	W12(+3.995914862)
3.9953	0.9130741	P24821	913	1	R.VSQTDNSITLEWR.N	W12(+3.996016034)
3.9953	0.9130741	Q8WVY7	236	1	R.GLIDVKPLGVIWGK.F	W12(+3.996786458)
3.9953	0.9130741	P25789	139	1	K.RPFGVSLLYIGWDK.H	W12(+3.999344359)
3.9953	0.9130741	P19835	247	1	R.AISQSGVALSPWVIQK.N	W12(+4.000574505)
3.9953	0.9130741	Q9UGI8	113	1	K.KNVSINTVTYEWAPPVQNQALAR.Q	W12(+4.001336531)
3.9953	0.9130741	P27797	236	1	K.IDDPTDSKPEDWDKPEHIPDPDAK.K	W12(+4.00245399)
3.9953	0.9130741	P04264	212	1	R.FLEQQNQVLQTKWELLQQVDTSTR.T	W13(+3.987445895)
3.9953	0.9130741	P02751	2014	1	R.FLATTPNSLLVSWQPPR.A	W13(+3.993511305)
3.9953	0.9130741	Q13813	2294	1	K.YTEHSTVGLAQQWDQLDQLGMR.M	W13(+3.993662289)
3.9953	0.9130741	P01911	90	1	R.AVTELGRPDAEYWNSQK.D	W13(+3.993674634)
3.9953	0.9130741	Q6UWR7	327	1	K.FVSPLTLVADEGWFITENR.E	W13(+3.993723564)
3.9953	0.9130741	P31323	245	1	R.AATITATSPGALWGLDR.V	W13(+3.994306182)
3.9953	0.9130741	P10745	1123	1	K.IYSRPDDSVSELWTHAQVVGER.Y	W13(+3.995151924)

3.9953	0.9130741	P13861	230	1	R.AATIVATSEGSLWGLDR.V	W13(+3.995784881)
3.9953	0.9130741	Q14697	623	1	R.FGAVWTGDNTAEWDHLK.I	W13(+3.996417947)
3.9953	0.9130741	P22105	860	1	R.VVAVTPTTLELGWLRPQAEVDR.F	W13(+3.997068454)
3.9953	0.9130741	Q05707	50	1	R.YNVISHDSIQISWK.A	W13(+3.997715277)
3.9953	0.9130741	Q14896	322	1	R.DSKLEAPAEEDVWEILR.Q	W13(+3.997819408)
3.9953	0.9130741	Q08211	1087	1	K.VQSDGQIVLVDDWIK.L	W13(+4.001486186)
3.9953	0.9130741	Q9UI47	67	1	R.ASVLLASVEEATWNLLDKGEK.I	W13(+4.006627952)
3.9953	0.9130741	Q08945	53	1	K.VDNIQAGELTEGIWR.R	W14(+3.994925087)
3.9953	0.9130741	P14060	231	1	K.FSTVNPVYVGNVAWAHILALR.A	W14(+3.995183734)
3.9953	0.9130741	Q9H9B4	69	1	R.QGIVPPGLTENELWR.A	W14(+3.996911234)
3.9953	0.9130741	P26232	906	1	K.VYGTAAVNSPVVSWK.M	W14(+3.996929775)
3.9953	0.9130741	P50502	187	1	R.AIEINPDSAQPYKWR.G	W14(+3.998652647)
3.9953	0.9130741	Q09666	2762	1	K.VDIDAPDVDVHGPDWHLK.M	W15(+3.990132215)
3.9953	0.9130741	Q09666	1378	1	K.VDISAPDVDVHGPDWHLK.M	W15(+3.992785016)
3.9953	0.9130741	P61326	77	1	R.IIDDSEITKEDDALWPPPDR.V	W15(+3.9934697)
3.9953	0.9130741	P09497	128	1	R.LQELDAASKVTEQEWR.E	W15(+3.99363454)
3.9953	0.9130741	O75874	336	1	K.GQETSTNPIASIFAWTR.G	W15(+3.993804342)
3.9953	0.9130741	P35442	149	1	R.HVVSLEDVGLADSQWK.N	W15(+3.994489857)
3.9953	0.9130741	P36957	89	1	K.TPAFAESVTEGDVRWEK.A	W15(+3.995769579)
3.9953	0.9130741	Q09666	3800	1	K.VDINAPDVDVQGPDWHLK.M	W15(+3.995783421)
3.9953	0.9130741	Q9UHG3	405	1	R.EKEDPEPSTDGTYVWK.I	W15(+3.995890705)
3.9953	0.9130741	Q09666	873	1	K.MDIDVPDVEVQGPDWHLK.M	W15(+3.996225957)
3.9953	0.9130741	Q09666	1506	1	K.VDINAPDVEVHGPDWHLK.M	W15(+3.996631422)
3.9953	0.9130741	Q09666	2239	1	K.VDIDAPDVDVHGPDWHLK.M	W15(+3.996724012)
3.9953	0.9130741	Q09666	1708	1	K.VDIDAPDVEVHDPDWHLK.M	W15(+3.996882623)
3.9953	0.9130741	Q8NCW5	223	1	K.GLTVPIASIDIPSGWDVEK.G	W15(+3.997337575)
3.9953	0.9130741	Q09666	1122	1	K.VDIKAPDVEGQGLDWSLKIPK.M	W15(+3.998836932)
3.9953	0.9130741	Q9BY49	280	1	R.SLYTHSYEVPDHDNWPK.G	W15(+4.000236081)
3.9953	0.9130741	P21549	288	1	R.ESLALIAEQGLENSWR.Q	W15(+4.002872325)
3.9953	0.9130741	Q9Y2J2	822	1	R.KPTEFIGGVTSTSQSWVQK.M	W16(+3.988915944)
3.9953	0.9130741	P52566	191	1	K.SFFTDDDKQDHLSWEWNLSIK.K	W16(+3.991674648)
3.9953	0.9130741	Q99584	77	1	K.SLDVNQDSELKFNEYWR.L	W16(+3.992327465)
3.9953	0.9130741	P07996	153	1	K.QHVVSVEEALLATGQWK.S	W16(+3.994172906)
3.9953	0.9130741	P09104	301	1	R.DYPVVSIEDPFDQDDWAAWSK.F	W16(+3.995406314)
3.9953	0.9130741	O95674	327	1	R.LQEYNIPGVIQSVIGWK.T	W16(+3.999398028)
3.9953	0.9130741	P02751	1559	1	R.DLEVVAATPTSLLISWDAPAVTVR.Y	W16(+4.001063421)
3.9953	0.9130741	Q99715	2128	1	R.TVGLLPPQNIHISDEWYTR.F	W16(+4.001967755)
3.9953	0.9130741	Q9Y6D6	1044	1	K.TLITVAHTDGNYLGNSWHEILK.C	W17(+3.996333678)
3.9953	0.9130741	Q9NQG5	29	1	K.LSELSNSQQSVQTLSLWLIHHR.K	W17(+3.996674858)
3.9953	0.9130741	Q13435	27	1	K.AELQLPPPPPPGHYGAWAAQELQAK.L	W17(+3.999312626)
3.9953	0.9130741	P15121	112	1	K.TLSDLKLDYLDLYLIHWPTGFKPGK.E	W17(+3.999321712)
3.9953	0.9130741	O43852	96	1	K.IDGDKDGFVTVDELKDWIK.F	W17(+4.001737454)
3.9953	0.9130741	Q7Z406	50	1	R.GPSAGGGPGSGTSPQVEWTAR.R	W18(+3.989854513)
3.9953	0.9130741	P28068	260	1	R.AGHSSYTPLPGSNYSEGWHIS.-	W18(+3.992155848)
3.9953	0.9130741	P0DJI8	103	1	R.FFGHGAEDSLADQAANEWGR.S	W18(+3.992581363)
3.9953	0.9130741	O75340	57	1	R.SGVISDTELQQALSNGTWTPFNPVTVR.S	W18(+3.996827607)
3.9953	0.9130741	O95372	40	1	R.ETAAVIFLHGLGDTGHSWADALSTIR.L	W18(+4.005277288)
3.9953	0.9130741	Q08211	139	1	K.AENNSEVGASGYGVPGPTWDR.G	W19(+3.992934621)
3.9953	0.9130741	Q13424	392	1	R.HGVDTHLFSVESPQELAAWTR.Q	W19(+3.992949629)
3.9953	0.9130741	Q14896	711	1	K.APARPAPDAPEDTGDSDEWVFDKK.L	W19(+3.999460939)
3.9953	0.9130741	Q86UE4	6	1	R.SWQDELAQQAEEGSAR.L	W2(+3.994844915)
3.9953	0.9130741	Q9UJU6	237	1	R.TWEQQQEVVSR.N	W2(+3.995227292)
3.9953	0.9130741	P01889	157	1	R.SWTAADTAAQITQR.K	W2(+3.995584677)
3.9953	0.9130741	Q01082	510	1	R.LWEYLLELLR.A	W2(+3.996253329)
3.9953	0.9130741	P15085	183	1	R.EWVTQASGVWFAK.K	W2(+3.998515799)
3.9953	0.9130741	Q00610	1150	1	K.ADDPSSYMEVVQAANTSGNWEELVK.Y	W20(+3.997662283)
3.9953	0.9130741	P07942	731	1	K.SLDIFTVGGSGDGVVTNSAWETFQR.Y	W20(+4.000626532)
3.9953	0.9130741	Q14195	291	1	K.GNVVFGEPITASLGIDGTHYWSK.N	W21(+3.996026119)
3.9953	0.9130741	Q99798	765	1	K.HPNGTQETILLNHTFNETQIEWFR.A	W22(+3.996519813)
3.9953	0.9130741	Q9HCN8	82	1	K.YGSGSGQQSVTGVEASDDANSYWR.I	W23(+3.995301412)
3.9953	0.9130741	P33241	84	1	K.QEMLLSLKPSEAPELDEDEGFGDWSQRPEQR.Q	W24(+3.994971531)
3.9953	0.9130741	Q02952	804	1	K.SEDSIAGSGVEHSTPDTEPGKEESWVSIK.K	W25(+3.989774342)
3.9953	0.9130741	Q08211	176	1	R.KEEQEVQATLESEEVDLNAGLHGNWTLENAK.A	W25(+3.992761895)
3.9953	0.9130741	P48449	273	1	R.LSAAEDPLVQSLRQELYVEDFASIDWLAQR.N	W26(+4.00568893)
3.9953	0.9130741	P02751	1195	1	K.VVTPLSPPTNLHLEANPDTGVLTVSWER.S	W26(+4.006444038)
3.9953	0.9130741	P02751	1743	1	K.IAWESPQGQVSR.Y	W3(+3.992380755)
3.9953	0.9130741	Q96G03	178	1	K.VYWDNGAQIISPHDK.G	W3(+3.992794461)
3.9953	0.9130741	P04217	409	1	R.ATWSGAVLAGR.D	W3(+3.993453706)
3.9953	0.9130741	Q01082	1574	1	K.QLWGLLIEETEKR.H	W3(+3.993469043)

3.9953	0.9130741	P17174	296	1	R.ITWSNPPAQGAR.I	W3(+3.993612666)
3.9953	0.9130741	Q99715	1251	1	R.TEWQLNAHR.D	W3(+3.993636841)
3.9953	0.9130741	P50895	485	1	K.LSWSQLGGSPAEPIPGR.Q	W3(+3.993905192)
3.9953	0.9130741	P04179	205	1	K.AIWNVINWENVTER.Y	W3(+3.994241036)
3.9953	0.9130741	P26038	43	1	R.EVWFFGLQYQDTK.G	W3(+3.994750778)
3.9953	0.9130741	Q8WZ42	28919	1	R.VTWFKDGVEIEKR.M	W3(+3.994768163)
3.9953	0.9130741	Q8WZ42	17564	1	K.LTWFSPEDDGGSPITNYVIEKR.E	W3(+3.995021285)
3.9953	0.9130741	Q15424	798	1	R.DGWGGYGSDKR.M	W3(+3.995036803)
3.9953	0.9130741	P46821	2412	1	K.AQWGSNMQVTLIPTHDSEVMR.E	W3(+3.995047183)
3.9953	0.9130741	Q8IZ83	120	1	R.LLWTLESLVTGR.A	W3(+3.995221063)
3.9953	0.9130741	P51970	117	1	K.LGWVRPDLGELSK.V	W3(+3.995279748)
3.9953	0.9130741	P04083	12	1	K.QAWFIENEEQEYVQTVK.S	W3(+3.995387428)
3.9953	0.9130741	O43169	42	1	K.ELWLVIHGR.V	W3(+3.995536859)
3.9953	0.9130741	P06737	175	1	R.DGWQVEEADDWLR.Y	W3(+3.99556137)
3.9953	0.9130741	Q92499	149	1	R.VGWSTMQASLDLGTDK.F	W3(+3.995757707)
3.9953	0.9130741	O15460	386	1	K.SSWLEEDDDPVVAR.V	W3(+3.995926088)
3.9953	0.9130741	Q15029	733	1	R.SIWAFGPDATGPNILVDDTLPSEVDKALLGSVK.D	W3(+3.996123779)
3.9953	0.9130741	P11310	342	1	R.AAWEVDSGRR.N	W3(+3.996267526)
3.9953	0.9130741	P01889	75	1	R.APWIEQEGPEYWDR.N	W3(+3.996499903)
3.9953	0.9130741	P02751	1378	1	R.VTWAPPPSIDLTNFLVR.Y	W3(+3.99677912)
3.9953	0.9130741	P62937	121	1	K.TEWLDGKHVVFGK.V	W3(+3.997044314)
3.9953	0.9130741	P43405	136	1	K.QTWNLQGQALEQAIISQKPQLEK.L	W3(+3.997735155)
3.9953	0.9130741	Q1KMD3	318	1	R.VGWSVDFSRPQLGEDEFSYGFDGR.G	W3(+4.000495298)
3.9953	0.9130741	Q05707	849	1	R.ITWDPPSSPVKGYR.I	W3(+4.00740482)
3.9953	0.9130741	Q14204	1701	1	K.INEWLTLVEK.E	W4(+3.99244054)
3.9953	0.9130741	P10415	30	1	R.GYEWDAGDVGAAPPGAAPAPGIFSSQPGHTPHPAASR.D	W4(+3.993473735)
3.9953	0.9130741	P01903	193	1	R.VEHWGLDEPLLK.H	W4(+3.993507345)
3.9953	0.9130741	Q13938	221	1	R.SGEWTEDEVLR.R	W4(+3.993631199)
3.9953	0.9130741	P35241	175	1	R.IQNWHEEHR.G	W4(+3.993769847)
3.9953	0.9130741	P46940	742	1	R.EQLWLANEGLITR.L	W4(+3.994234513)
3.9953	0.9130741	P20336	125	1	K.TYSWDNAQVLLVGNK.C	W4(+3.994732237)
3.9953	0.9130741	Q92522	79	1	K.KVPWFDQQNGR.T	W4(+3.99488231)
3.9953	0.9130741	P54317	47	1	K.LLPWSPEDIDTR.F	W4(+3.995049459)
3.9953	0.9130741	P07942	583	1	R.IPSWTGAGFVR.V	W4(+3.995087498)
3.9953	0.9130741	P61019	60	1	K.LQIWDTAGQESFR.S	W4(+3.995635968)
3.9953	0.9130741	P09960	222	1	R.TLVWSEKEQVEK.S	W4(+3.996262179)
3.9953	0.9130741	Q15392	337	1	R.SIFWELQDIIPFGNNPIFR.Y	W4(+3.997812353)
3.9953	0.9130741	P04792	16	1	R.GPSWDPFRDWYPHSR.L	W4(+3.998959346)
3.9953	0.9130741	P49588	919	1	K.ASEWVQQVSGLMDGK.G	W4(+3.999334019)
3.9953	0.9130741	O75947	13	1	K.TIDWVAFAEIIPQNQK.A	W4(+4.002048792)
3.9953	0.9130741	O14548	16	1	K.LAGAWASEAYSPQGLKPVVSTEAPPIIFATPTK.L	W5(+3.989942423)
3.9953	0.9130741	P30711	84	1	K.VPDYWYPQDLQAR.A	W5(+3.992656152)
3.9953	0.9130741	Q13813	1673	1	K.DFDFWLSEVEALLASEDYGK.D	W5(+3.993762748)
3.9953	0.9130741	P00491	16	1	K.NTAEWLLSHTK.H	W5(+3.994200873)
3.9953	0.9130741	Q5UCC4	76	1	R.GSLLWNQQDGTLSLSQR.Q	W5(+3.994413852)
3.9953	0.9130741	P48735	164	1	R.LVPGWTKPITIGR.H	W5(+3.994696245)
3.9953	0.9130741	P09382	69	1	K.DGGAWGTEQR.E	W5(+3.994822139)
3.9953	0.9130741	P12814	381	1	K.GYEEWLLNEIR.R	W5(+3.994897022)
3.9953	0.9130741	P10745	824	1	K.VTEVWTLPQVAGQR.Y	W5(+3.995029161)
3.9953	0.9130741	P50583	94	1	K.TVIYWLAEVK.D	W5(+3.995366087)
3.9953	0.9130741	P06732	228	1	K.SFLVWVNEEDHLR.V	W5(+3.995512709)
3.9953	0.9130741	P29762	88	1	R.SLATWENENK.I	W5(+3.995819022)
3.9953	0.9130741	O14773	470	1	R.VPIPWVSGTSASTPVFGGILSLINEHR.I	W5(+3.996024112)
3.9953	0.9130741	P02675	415	1	R.DNDGWLTSDPR.K	W5(+3.996301728)
3.9953	0.9130741	P34949	74	1	K.TLSQWIAENQDSLGSK.V	W5(+3.997191639)
3.9953	0.9130741	P15085	173	1	K.RPAIWIDTGIHSR.E	W5(+3.997429599)
3.9953	0.9130741	O75340	95	1	K.YITDWQNVFR.T	W5(+3.997563411)
3.9953	0.9130741	P61163	341	1	R.LYSTWIGGSILASLDTFKK.M	W5(+3.999672233)
3.9953	0.9130741	P49419	363	1	R.VGNPWDPNVLYGPLHTK.Q	W5(+4.000104525)
3.9953	0.9130741	Q96SL4	159	1	K.VVGAWDPTVSVEEVRPQITALVR.K	W5(+4.001397537)
3.9953	0.9130741	P35222	25	1	K.AAVSHWQQQSYLDSGIHSGATTTAPSLSGK.G	W6(+3.990643353)
3.9953	0.9130741	P08238	312	1	K.SLTNDWEDHLAVK.H	W6(+3.993296549)
3.9953	0.9130741	Q9Y490	351	1	K.EVIQEWNLTNIK.R	W6(+3.993718784)
3.9953	0.9130741	Q9NZN9	278	1	R.AHAEVWNEAEAK.A	W6(+3.993888931)
3.9953	0.9130741	O14521	66	1	K.AASLHWTSER.V	W6(+3.994215332)
3.9953	0.9130741	Q9UJU6	84	1	K.FVLINWTGEGVNDVR.K	W6(+3.994616962)
3.9953	0.9130741	Q9ULV4	138	1	R.VGIVAWHPTAR.N	W6(+3.994868575)
3.9953	0.9130741	O94875	900	1	R.KIDQNWYEGEHHGR.V	W6(+3.994951356)
3.9953	0.9130741	Q96BZ4	265	1	K.TFQTYWVLGVPK.A	W6(+3.994956271)

3.9953	0.9130741	P53674	193	1	K.VSSGTWVGYQYPGYR.G	W6(+3.994996139)
3.9953	0.9130741	O60832	52	1	K.LDTSQWPLLLK.N	W6(+3.995382325)
3.9953	0.9130741	P78527	3805	1	R.LGLIEWLENTVTLK.D	W6(+3.995404756)
3.9953	0.9130741	O43324	74	1	K.AIVQQWLEYR.V	W6(+3.995601733)
3.9953	0.9130741	Q9HB40	425	1	K.NLAFYWILK.A	W6(+3.995688075)
3.9953	0.9130741	Q15029	549	1	R.VPAGNWVLIEGVDQPIVK.T	W6(+3.995907246)
3.9953	0.9130741	Q6UW68	115	1	R.TTAAMWALQTVEK.E	W6(+3.996236818)
3.9953	0.9130741	P05109	54	1	K.KGADVWFK.E	W6(+3.996295184)
3.9953	0.9130741	P13591	54	1	K.DKDISWFSPNGEK.L	W6(+3.996421437)
3.9953	0.9130741	Q9Y224	82	1	R.QEAIDWLLGLAVR.L	W6(+3.996427638)
3.9953	0.9130741	Q92835	597	1	R.VDLPTWEAETIIQK.I	W6(+3.997105783)
3.9953	0.9130741	P08648	744	1	K.AGASLWGGLR.F	W6(+3.997736184)
3.9953	0.9130741	P04746	299	1	K.NWGEGWGFVPSDR.A	W6(+3.998188839)
3.9953	0.9130741	Q15084	200	1	K.NLEPEWAAAASEVK.E	W6(+3.998202364)
3.9953	0.9130741	P43320	151	1	R.VQSGTWVGYQYPGYR.G	W6(+3.998720475)
3.9953	0.9130741	O95573	687	1	R.LSPEPWTPETGLVTDAFK.L	W6(+4.001109656)
3.9953	0.9130741	Q9HBL0	79	1	K.VLEFGWPDLHTPALEK.I	W6(+4.002014854)
3.9953	0.9130741	P13010	675	1	K.QLNHFWEIVVQDGITLITK.E	W6(+4.002773281)
3.9953	0.9130741	P05108	126	1	R.FLIPPWVAYHQYYQRPIGVLLK.K	W6(+4.007708903)
3.9953	0.9130741	Q13813	1991	1	K.ADVVESWIGEK.E	W7(+3.992213505)
3.9953	0.9130741	P18206	253	1	R.VLQLTSWDEDAWASK.D	W7(+3.99260867)
3.9953	0.9130741	Q8WZ42	29886	1	R.STVSLIWSAPAYDGGSK.V	W7(+3.994292591)
3.9953	0.9130741	P09874	246	1	K.AQNDLIWNIKDELKK.V	W7(+3.99441908)
3.9953	0.9130741	P13591	555	1	R.AVGEEVWHSK.W	W7(+3.994748108)
3.9953	0.9130741	P04114	1141	1	R.SEILAHWSPAK.L	W7(+3.994832149)
3.9953	0.9130741	P22234	410	1	R.ASILNTWISLK.Q	W7(+3.994863677)
3.9953	0.9130741	Q13491	197	1	R.QYGIIPWNAFPGK.I	W7(+3.995522127)
3.9953	0.9130741	P02671	360	1	R.PGSTGTWNPGSSER.G	W7(+3.995525701)
3.9953	0.9130741	P0C0L5	1106	1	K.LQETSNWLLSQQQADGSFQDLSPVIHR.S	W7(+3.995639683)
3.9953	0.9130741	P84095	56	1	R.TVNLNLWDTAGQEEYDR.L	W7(+3.995869244)
3.9953	0.9130741	P34932	658	1	K.LEDTENWLYEDGEDQPK.Q	W7(+3.995904288)
3.9953	0.9130741	P08134	58	1	K.QVELALWDTAGQEDYDR.L	W7(+3.996148165)
3.9953	0.9130741	Q8WZ42	24297	1	R.DTTTTVWDVVSATVAR.T	W7(+3.996291431)
3.9953	0.9130741	P53396	49	1	R.LLQDHPWLLSQNLVVKPDQLIK.R	W7(+3.996800414)
3.9953	0.9130741	P07741	98	1	K.LPGPTLWASYSLEYGK.A	W7(+3.997020875)
3.9953	0.9130741	Q8WZ42	25063	1	K.DSAFLVWEPPIIDGGAK.V	W7(+3.99862844)
3.9953	0.9130741	P06737	826	1	K.EYAQNIWNVEPSDLK.I	W7(+3.998854908)
3.9953	0.9130741	P42768	252	1	K.HVSHVGWDPQNGFDVNNLDPDLR.S	W7(+3.998967993)
3.9953	0.9130741	P63208	88	1	R.TDDIPVWDQEFLK.V	W7(+3.999707289)
3.9953	0.9130741	P61204	66	1	K.NISFTVWDVGGQDK.I	W7(+3.999754498)
3.9953	0.9130741	P17252	223	1	R.STLNPQWNESFTFK.L	W7(+4.000091232)
3.9953	0.9130741	P04179	210	1	K.AIWNVINWENVTER.Y	W8(+3.990016324)
3.9953	0.9130741	Q13813	1106	1	R.EANELQQWINEK.E	W8(+3.99345697)
3.9953	0.9130741	Q13813	270	1	R.DVDETISWIK.E	W8(+3.993468988)
3.9953	0.9130741	Q14204	1261	1	R.TTDLLTDWEK.T	W8(+3.994037764)
3.9953	0.9130741	Q13683	606	1	K.HQASGTVWLK.H	W8(+3.994038993)
3.9953	0.9130741	P02763	178	1	K.SDVVYTDWKK.D	W8(+3.994574416)
3.9953	0.9130741	P26640	417	1	R.EAFLQEVWK.W	W8(+3.99478405)
3.9953	0.9130741	Q16352	286	1	K.NLQSAEEWYK.S	W8(+3.995158542)
3.9953	0.9130741	P98160	1806	1	K.SPAYTLVWTR.L	W8(+3.995421849)
3.9953	0.9130741	Q9UMS6	1086	1	K.SGVTIQVWKPSVVEE.-	W8(+3.9958503)
3.9953	0.9130741	O94804	36	1	R.DLDPNEVWEIVGELGDGAFGK.V	W8(+3.996226299)
3.9953	0.9130741	P09496	146	1	K.AIKELEEWYAR.Q	W8(+3.996670266)
3.9953	0.9130741	Q96G03	22	1	R.LDQETAQWLR.W	W8(+3.996692408)
3.9953	0.9130741	P46459	213	1	R.QSIINPDWNFEK.M	W8(+3.996748728)
3.9953	0.9130741	Q13813	799	1	R.DVEDEETWIREKEPIAASTNR.G	W8(+3.996977388)
3.9953	0.9130741	P28331	319	1	K.GLLTYTSWEDALSR.V	W8(+3.997440465)
3.9953	0.9130741	Q13162	88	1	K.ISKPAPYWEGTAVIDGEFK.E	W8(+3.997924372)
3.9953	0.9130741	Q01082	2032	1	R.DASVAEAWLLGQEPYLSSR.E	W8(+3.998934263)
3.9953	0.9130741	Q01082	1893	1	R.ENEVLEAWK.S	W8(+3.999023489)
3.9953	0.9130741	P07814	1027	1	K.KEENLADWYSQVITK.S	W8(+3.999267996)
3.9953	0.9130741	P50440	104	1	K.ANTYEKYWPFYQK.Q	W8(+4.000964269)
3.9953	0.9130741	P39019	52	1	K.ELAPYDENWFYTR.A	W9(+3.992851921)
3.9953	0.9130741	P05091	93	1	R.AAFQLGSPWR.R	W9(+3.993373211)
3.9953	0.9130741	Q15149	3167	1	R.GANVIAGVWLEEAGQK.L	W9(+3.993700243)
3.9953	0.9130741	P23526	17	1	K.VADIGLAAWGR.K	W9(+3.993891859)
3.9953	0.9130741	P11047	635	1	R.LHEATDYPWRPALTPFEFQK.L	W9(+3.994161749)
3.9953	0.9130741	P51812	332	1	R.HSFFSTIDWNK.L	W9(+3.995568711)
3.9953	0.9130741	Q15149	1359	1	R.ESADPLGAWLQDAR.R	W9(+3.995741527)

3.9953	0.9130741	Q8N126	62	1	K.DHEDSSLQWSNPAQQTLYFGEK.R	W9(+3.996392653)
3.9953	0.9130741	Q9BUR5	181	1	R.GYIVIEDLWK.E	W9(+3.996692349)
3.9953	0.9130741	P04181	178	1	K.IVFAAGNFWGR.T	W9(+3.997047418)
3.9953	0.9130741	O43301	207	1	R.WVITVPAIWK.Q	W9(+3.99814158)
3.9953	0.9130741	Q9NX14	55	1	K.RPPEPTTPWQEDPEPEDENLYEK.N	W9(+3.99960985)
3.9953	0.9130741	P02675	454	1	R.YYWGGQYTWDMAK.H	W9(+3.99962856)
3.9953	0.9130741	P00505	162	1	R.DVFLPKPTWGNHTPIFR.D	W9(+4.000647671)
3.9953	0.9130741	O15230	2360	1	R.VQEQLSSLWEENQALATQTR.D	W9(+4.001453862)
4.9792	0.6640700	Q9Y6R7	2501	1	R.FAVLQENVAWGNGR.V	W10(+4.976118309)
4.9792	0.6640700	P69892	131	1	K.EFTPEVQASWQK.M	W10(+4.978742166)
4.9792	0.6640700	P06744	380	1	R.VDHQTGPIVWGEPGTNGQHAFYQLIHQGTK.M	W10(+4.982172509)
4.9792	0.6640700	P01859	192	1	R.VVSVLTVVHQDWLNGK.E	W12(+4.979525933)
4.9792	0.6640700	P45379	247	1	K.ELWQSIYNLEAEKFDLQEK.F	W3(+4.996784781)
4.9792	0.6640700	P18206	394	1	K.KIDAAQNWLADPNGGPEGEEQIR.G	W8(+4.978925126)
4.9792	0.6640700	P02671	295	1	R.NPSSAGSWNSGSSGPGSTGNR.N	W8(+4.994546291)
4.9792	0.6640700	P06396	605	1	K.TPSAAYLWVGTGASEAEK.T	W8(+4.998274923)
15.9949	0.9191695	P01876	162	1	R.DASGVTFTWTPSSGK.S	W9(+15.987931946)
15.9949	0.9191695	Q01082	726	1	R.EQWANLEQLSAIR.K	W3(+15.988959263)
15.9949	0.9191695	P62873	82	1	K.LIIWDSYTTNK.V	W4(+15.991565124)
15.9949	0.9191695	Q13885	21	1	K.FWEVISDEHGIDPTGSYHGDSDLQLER.I	W2(+15.984758288)
15.9949	0.9191695	P68371	21	1	K.FWEVISDEHGIDPTGTYHGDSDLQLER.I	W2(+15.988761544)
15.9949	0.9191695	P20336	76	1	K.LQIWDTAGQER.Y	W4(+15.986825364)
15.9949	0.9191695	P10809	68	1	R.TVIIEQSWGSPK.V	W8(+15.991530344)
15.9949	0.9191695	P13591	555	1	R.AVGEEVWHSK.W	W7(+15.990597718)
15.9949	0.9191695	Q92752	1061	1	R.QSALISWQPPR.A	W7(+15.987779518)
15.9949	0.9191695	P27797	33	1	K.EQFLDGDGWTSR.W	W9(+15.992096796)
15.9949	0.9191695	P12110	696	1	K.NLEWIAGGTWTPSALK.F	W4(+15.992892629)
15.9949	0.9191695	Q8NCW5	223	1	K.GLTVPIASIDIPSGWDVEK.G	W15(+15.988304371)
15.9949	0.9191695	Q13813	237	1	K.TKQDEVNAAWQR.L	W10(+15.9917147)
15.9949	0.9191695	Q9BWM7	68	1	R.AGVVTPGITEDQLWR.A	W14(+15.993700243)
15.9949	0.9191695	P68032	81	1	K.YPIEHGIITNWDDMEK.I	W11(+15.991085145)
15.9949	0.9191695	Q01813	463	1	K.EIGWTDVGGWTGQGGSILGTK.R	W4(+15.991941475)
15.9949	0.9191695	P18206	258	1	R.VLQLTSWDEDAWASK.D	W12(+15.995416288)
15.9949	0.9191695	P12814	289	1	K.LASDLLEWIR.R	W8(+15.994694729)
15.9949	0.9191695	P21796	64	1	R.WTEYGLTFTEK.W	W1(+15.991364316)
15.9949	0.9191695	Q00610	164	1	K.WLLLTGISAQQNR.V	W1(+15.994307948)
15.9949	0.9191695	P29401	257	1	R.GITGVEDKESWHGKPLPK.N	W11(+15.987332556)
15.9949	0.9191695	P17174	141	1	K.NTPVYVSSPTWENHNAVFSAAGFK.D	W11(+15.998254973)
15.9949	0.9191695	P02751	1378	1	R.VTWAPPPSIDLTNFLVR.Y	W3(+15.996975512)
15.9949	0.9191695	O43707	400	1	K.GYEEWLLNEIR.R	W5(+15.995995655)
15.9949	0.9191695	P21333	345	1	R.TFSVWYVPEVTGTHK.V	W5(+15.99831028)
15.9949	0.9191695	P61626	52	1	K.WESGYNTR.A	W1(+15.993046788)
15.9949	0.9191695	P10321	268	1	K.WAAVVVPSGQEQR.Y	W1(+15.993125275)
15.9949	0.9191695	P02763	43	1	K.WFYIASAFR.N	W1(+15.994924125)
15.9949	0.9191695	P05093	313	1	K.WTLAFLLHNPQVK.K	W1(+15.997328121)
15.9949	0.9191695	P55072	454	1	R.WALSQSNPSALR.E	W1(+15.997549283)
15.9949	0.9191695	P23141	74	1	R.FTPPQPAEPWSFVK.N	W10(+15.989681911)
15.9949	0.9191695	P09211	29	1	R.MLLADQGQSWK.E	W10(+15.994130366)
15.9949	0.9191695	P05062	314	1	R.ALQASALAAWGGK.A	W10(+15.994894375)
15.9949	0.9191695	P27824	455	1	R.IVDDWANDGWGLK.K	W10(+15.996163441)
15.9949	0.9191695	Q8NC51	319	1	R.KPNEGADGQWK.K	W10(+15.996279319)
15.9949	0.9191695	P05060	381	1	R.APRPQSEESWDEEDKR.N	W10(+15.996653857)
15.9949	0.9191695	A8K7I4	85	1	K.NVAILIPETWK.T	W10(+15.996913808)
15.9949	0.9191695	P15085	191	1	R.EWVTQASGVWFAK.K	W10(+15.99875994)
15.9949	0.9191695	P12110	702	1	K.NLEWIAGGTWTPSALK.F	W10(+15.998874074)
15.9949	0.9191695	P06748	288	1	R.MTDQEAIQDLWQWR.K	W11(+15.991935828)
15.9949	0.9191695	P14625	485	1	K.IADDKYNDTFWK.E	W11(+15.992591542)
15.9949	0.9191695	P24462	126	1	K.NAISIAEDEEWKR.I	W11(+15.993988588)
15.9949	0.9191695	P00751	334	1	K.VSEADSSNADWVTK.Q	W11(+15.999739745)
15.9949	0.9191695	P09093	40	1	R.VVHGEDAVPYSWPWQVSLQYEK.S	W12(+15.992639709)
15.9949	0.9191695	Q14103	97	1	R.HSEAATAQREEWK.M	W12(+15.993084298)
15.9949	0.9191695	P22314	109	1	K.AVTLHDQGTAQWADLSSQFYLR.E	W12(+15.993280758)
15.9949	0.9191695	Q04760	171	1	K.GLAFIQDPDGYWIEILNPNK.M	W12(+15.995403071)
15.9949	0.9191695	P0C0L5	116	1	R.GPEVQLVAHSPWLK.D	W12(+15.996367794)
15.9949	0.9191695	P35579	26	1	K.NFINNPLAQADWAAK.K	W12(+15.996564785)
15.9949	0.9191695	P07099	33	1	R.DKEETLPLEDGWWGPGTR.S	W12(+16.002915705)
15.9949	0.9191695	P02671	321	1	R.NPGSSGTGGTATWK.P	W13(+15.992773787)
15.9949	0.9191695	P05060	359	1	K.GYPGVQAPEDLEWER.Y	W13(+15.994235998)
15.9949	0.9191695	P07099	34	1	R.DKEETLPLEDGWWGPGTR.S	W13(+15.995835627)

15.9949	0.9191695	P52566	189	1	K.SFFTDDDKQDHLSWEWNLSIK.K	W14(+15.988561856)
15.9949	0.9191695	P14060	231	1	K.FSTVNPVYVGNVAWAHILALR.A	W14(+15.991521624)
15.9949	0.9191695	P62753	45	1	R.MATEVAADALGEEWK.G	W14(+15.995089542)
15.9949	0.9191695	P15085	53	1	K.VKELEDLEHLQLDFWR.G	W15(+15.993771614)
15.9949	0.9191695	P48735	375	1	K.GRPTSTNPIASIFAWTR.G	W15(+15.995543429)
15.9949	0.9191695	P37802	117	1	R.YGINTTDIFQTVDLWEGK.N	W15(+15.99629321)
15.9949	0.9191695	Q9UQ80	170	1	R.LVKPGNQNTQVTEAWNK.V	W15(+15.996650313)
15.9949	0.9191695	P21549	288	1	R.ESLALIAEQGLENSWR.Q	W15(+16.000810416)
15.9949	0.9191695	P04179	149	1	K.LTAASVGVQGSGWGWLGFNK.E	W15(+16.001232683)
15.9949	0.9191695	Q09666	745	1	K.VDIDAPDVDVHGPDWHLK.M	W15(+16.00290185)
15.9949	0.9191695	Q9BSF4	232	1	K.YKPVVLTDDQVDQALWEEQVLQK.E	W16(+15.994526782)
15.9949	0.9191695	Q01082	202	1	K.TAGYPNVNIHNFTTSWR.D	W16(+15.997123576)
15.9949	0.9191695	P0DPI2	204	1	R.GVEVTVGHEQEEGGKWPYAGTAEAIK.A	W16(+15.997861932)
15.9949	0.9191695	P06744	228	1	K.TFTTQETITNAETAKEWFLQAAK.D	W17(+15.996706647)
15.9949	0.9191695	Q14896	711	1	K.APARPAPDAPEDTGDSDEWVFDKK.L	W19(+15.994248439)
15.9949	0.9191695	O43707	623	1	K.LSGSNPYTTVTPQIINSKWEK.V	W19(+15.995913764)
15.9949	0.9191695	P04746	295	1	K.NWGEGWGFVPSDR.A	W2(+15.989765988)
15.9949	0.9191695	P31327	833	1	K.EWPSNLDLR.K	W2(+15.992062562)
15.9949	0.9191695	Q9BVA1	21	1	K.FWEVISDEHGIDPTGSYHGDSDLQLER.I	W2(+15.992582014)
15.9949	0.9191695	P21579	391	1	R.HWSDMLANPR.R	W2(+15.992585358)
15.9949	0.9191695	P04004	339	1	R.DWHGVPGQVDAAMAGR.I	W2(+15.992786511)
15.9949	0.9191695	Q13813	2065	1	K.RWSQLLANSAAR.K	W2(+15.993433135)
15.9949	0.9191695	Q14315	577	1	R.AWGPGLETGQVGK.S	W2(+15.993956733)
15.9949	0.9191695	P05108	263	1	K.TWKDHVAAWDVIFSK.A	W2(+15.995449615)
15.9949	0.9191695	P30084	120	1	K.HWDHLTQVK.K	W2(+15.995494122)
15.9949	0.9191695	Q9UJU6	237	1	R.TWEQQQEVVSR.N	W2(+15.995837643)
15.9949	0.9191695	Q03135	85	1	K.IDFEDVIAEPEGTHSFDGIWK.A	W20(+15.995817324)
15.9949	0.9191695	Q9H4B7	101	1	K.LGALFQPDSFVHGNSGAGNNWAK.G	W21(+15.993006553)
15.9949	0.9191695	Q16555	291	1	K.GTVVYGEPITASLGTDGSHYWSK.N	W21(+15.994685103)
15.9949	0.9191695	P07099	412	1	K.VYVPTGFSAFPFELLHTPEKWVR.F	W21(+15.999962144)
15.9949	0.9191695	O75390	136	1	K.GGEEPLPEGLFWLLVTGHIPTEEQVSWLSK.E	W27(+15.994374985)
15.9949	0.9191695	Q06520	77	1	R.SPWVESEIGYTALSETESPR.L	W3(+15.992791819)
15.9949	0.9191695	P01889	75	1	R.APWIEQEGPEYWDR.N	W3(+15.994912989)
15.9949	0.9191695	Q01082	437	1	R.ETWLSENQR.L	W3(+15.995875946)
15.9949	0.9191695	Q9P2U7	497	1	K.QPWAEPEEMSEEK.C	W3(+15.996281635)
15.9949	0.9191695	A8K7I4	273	1	R.STWEVIRDSEDFKK.T	W3(+15.996466854)
15.9949	0.9191695	Q8WZ42	17564	1	K.LTWFSPEDDGGSPITNYVIEKR.E	W3(+16.000514449)
15.9949	0.9191695	P08686	405	1	R.TTRPSSISGYDIPEGTVIIPNLQGAHLDETVWERPHEFWPDR.F	W39(+15.998854042)
15.9949	0.9191695	Q9GZM7	407	1	K.ITGWGEETLPDGR.T	W4(+15.990165485)
15.9949	0.9191695	O60684	400	1	K.EAAWAITNATSGGTPEQIR.Y	W4(+15.992718723)
15.9949	0.9191695	P38919	292	1	R.KVDWLTEK.M	W4(+15.99368983)
15.9949	0.9191695	P61457	25	1	R.AVGWNELEGR.D	W4(+15.994738393)
15.9949	0.9191695	P11233	149	1	R.AEQWNVNYVETSAK.T	W4(+15.995094903)
15.9949	0.9191695	P26439	355	1	K.TVEWVGSLVDR.H	W4(+15.996799129)
15.9949	0.9191695	P35749	37	1	K.RLVWVPSEK.Q	W4(+15.997702265)
15.9949	0.9191695	P01023	178	1	R.IAQWQSFQLEGGLK.Q	W4(+15.997871558)
15.9949	0.9191695	P12109	86	1	R.NLVWNAGALHYSDEVEIIQGLTR.M	W4(+16.000073061)
15.9949	0.9191695	P63267	341	1	K.YSVWIGGSILASLSTFQQMWISKPEYDEAGPSIVHR.K	W4(+16.005010204)
15.9949	0.9191695	P51149	102	1	K.TLDSWRDEFLIQASPR.D	W5(+15.990728711)
15.9949	0.9191695	P00387	246	1	R.APEAWDYGQGFVNEEMIR.D	W5(+15.990872592)
15.9949	0.9191695	Q07021	109	1	K.MSGGWELELNGTEAK.L	W5(+15.992150139)
15.9949	0.9191695	P35237	137	1	K.HINTWVAEK.T	W5(+15.992756064)
15.9949	0.9191695	P11177	178	1	K.VVSPWNSEDAK.G	W5(+15.993571628)
15.9949	0.9191695	P49419	363	1	R.VGNPWDPNVLYGPLHTK.Q	W5(+15.994611361)
15.9949	0.9191695	P05023	418	1	K.TSATWLALSR.I	W5(+15.994739555)
15.9949	0.9191695	P27824	450	1	R.IVDDWANDGWGLK.K	W5(+15.995308949)
15.9949	0.9191695	P19440	554	1	R.TAGGWAAASDSR.K	W5(+15.996013365)
15.9949	0.9191695	Q06520	65	1	K.GDAKWIQSVPIWER.S	W5(+15.997166583)
15.9949	0.9191695	P11216	175	1	K.IVNGWQVEEADDWLR.Y	W5(+15.997509215)
15.9949	0.9191695	Q9HBL0	650	1	R.GLNSWQQQQQQQQPRPPPR.Q	W5(+15.997741723)
15.9949	0.9191695	P31327	152	1	K.SLGQWLQEEKVPAIYGVDTR.M	W5(+16.001040467)
15.9949	0.9191695	Q14019	81	1	K.FALITWIGENVSGLQR.A	W6(+15.993358229)
15.9949	0.9191695	P22352	181	1	K.VHDIRWNFEK.F	W6(+15.994361599)
15.9949	0.9191695	P49458	7	1	M.PQYQTWEEFSR.A	W6(+15.99477379)
15.9949	0.9191695	P50993	1013	1	R.RYPGGWVEK.E	W6(+15.995019969)
15.9949	0.9191695	Q01995	55	1	R.LGFQVWLK.N	W6(+15.995320975)
15.9949	0.9191695	Q9UQC9	166	1	R.VFVHEWAHLR.W	W6(+15.998709528)
15.9949	0.9191695	P02792	90	1	K.KPAEDEWGKTPDAMK.A	W7(+15.990806177)
15.9949	0.9191695	P06737	826	1	K.EYAQNIWNVEPSDLK.I	W7(+15.993483814)

15.9949	0.9191695	P61586	58	1	K.QVELALWDTAGQEDYDR.L	W7(+15.993706758)
15.9949	0.9191695	P26038	445	1	K.ESEAVEWQQK.A	W7(+15.99477582)
15.9949	0.9191695	P18124	209	1	K.EANNFLWPFK.L	W7(+15.995421849)
15.9949	0.9191695	Q00610	514	1	K.VGYTPDWIFLLR.N	W7(+15.995750919)
15.9949	0.9191695	P18206	253	1	R.VLQLTSWDEDAWASK.D	W7(+15.99639285)
15.9949	0.9191695	O75083	133	1	K.FGAVFLWDSGSSVGEITGHNK.V	W7(+15.996568052)
15.9949	0.9191695	P84077	66	1	K.NISFTVWDVGGQDK.I	W7(+15.996824811)
15.9949	0.9191695	Q9Y3E1	86	1	K.GFNEGLWEIENNPGVK.F	W7(+16.002060596)
15.9949	0.9191695	P61026	103	1	K.SFENISKWLR.N	W8(+15.992086758)
15.9949	0.9191695	P14866	549	1	R.SSSGLLEWESK.S	W8(+15.993051486)
15.9949	0.9191695	P17661	295	1	K.NISEAEEWYK.S	W8(+15.993076551)
15.9949	0.9191695	Q14103	106	1	K.MFIGGLSWDTTK.K	W8(+15.993560096)
15.9949	0.9191695	Q14697	566	1	K.DAQHYGGWEHR.D	W8(+15.994676229)
15.9949	0.9191695	O43707	308	1	K.LASDLLEWIR.R	W8(+15.9948168)
15.9949	0.9191695	Q13813	1248	1	R.DADETKEWIEEK.N	W8(+15.995267638)
15.9949	0.9191695	P35609	296	1	R.LASELLEWIR.R	W8(+15.996134509)
15.9949	0.9191695	P26439	171	1	K.AVLAANGWNLK.N	W8(+15.997259699)
15.9949	0.9191695	P33121	120	1	R.KPDQPYEWLSYK.Q	W8(+15.998295229)
15.9949	0.9191695	P00918	97	1	R.LIQFHFHWGSLDGQGSEHTVDK.K	W8(+16.001136896)
15.9949	0.9191695	P18124	142	1	R.IVEPYIAWGYPNLK.S	W8(+16.002521107)
15.9949	0.9191695	P11047	540	1	R.DGSEASLEWSSER.Q	W9(+15.992271642)
15.9949	0.9191695	Q9H9H5	164	1	R.VITTHTSGWDSSPGAGFQVPEVR.K	W9(+15.993426701)
15.9949	0.9191695	P49821	311	1	K.HAGGVTGGWDNLLAVIPGGSSTPLIPK.S	W9(+15.994399145)
15.9949	0.9191695	P39019	52	1	K.ELAPYDENWFYTR.A	W9(+15.994560906)
15.9949	0.9191695	P60174	195	1	K.VIADNVKDWSK.V	W9(+15.994838171)
15.9949	0.9191695	P00352	77	1	R.QAFQIGSPWR.T	W9(+15.995346987)
15.9949	0.9191695	P12883	1456	1	R.NFDKILAEWK.Q	W9(+15.995671292)
15.9949	0.9191695	Q06830	177	1	K.HGEVCPAGWKPGSDTIKPDVQK.S	W9(+15.9959285)
15.9949	0.9191695	P30041	33	1	R.FHDFLGDSWGILFSHPR.D	W9(+15.996092745)
15.9949	0.9191695	P05093	220	1	K.DSLVDLVPWLK.I	W9(+15.996296661)
15.9949	0.9191695	Q9NR12	14	1	K.VVLEGPAPWGFR.L	W9(+15.998303791)
15.9949	0.9191695	Q00839	805	1	K.NQSQGYNQWQQGQFWGQK.P	W9(+15.999186751)
19.9903	0.3691468	Q01082	1362	1	K.MWEVLESTTQTK.A	W2(+19.986569956)
19.9903	0.3691468	P01011	217	1	K.WEMPFDPQDTHQSR.F	W1(+19.989667492)
19.9903	0.3691468	P69892	131	1	K.EFTPEVQASWQK.M	W10(+19.995099588)
19.9903	0.3691468	P68032	81	1	K.YPIEHGIITNWDDMEKIWHHTFYNELR.V	W11(+19.984630686)
19.9903	0.3691468	P01857	264	1	K.GFYPSDIAVEWESNGQPENNYK.T	W11(+20.003683251)
19.9903	0.3691468	P40939	80	1	K.ELHSEFSEVMNEIWASDQIR.S	W14(+20.000315407)
19.9903	0.3691468	P62736	88	1	K.IWHHSFYNELR.V	W2(+19.99175893)
19.9903	0.3691468	P02686	250	1	R.FSWGAEGQRPGFGYGGR.A	W3(+19.98862172)
19.9903	0.3691468	P68363	407	1	R.AFVHWYVGEGMEEGEFSEAR.E	W5(+19.987404223)
19.9903	0.3691468	Q07021	109	1	K.MSGGWELELNGTEAK.L	W5(+19.99276049)
19.9903	0.3691468	P61158	86	1	R.HGIVEDWDLMER.F	W7(+19.992685901)
19.9903	0.3691468	P07437	344	1	K.NSSYFVEWIPNNVK.T	W8(+19.989975907)
19.9903	0.3691468	O43707	308	1	K.LASDLLEWIR.R	W8(+19.992497464)
19.9903	0.3691468	P51911	82	1	K.INESTQNWHQLENIGNFIK.A	W8(+19.993394574)
19.9903	0.3691468	P60981	104	1	R.KEELMFFLWAPELAPLK.S	W9(+19.991962018)
19.9903	0.3691468	P23528	104	1	K.KEDLVFIFWAPESAPLK.S	W9(+19.992411702)
31.9899	0.9376182	P01877	149	1	R.DASGATFTWTPSSGK.S	W9(+31.981512348)
31.9899	0.9376182	P61764	288	1	K.EVLLDEDDDLWIALR.H	W11(+31.98092559)
31.9899	0.9376182	P18669	167	1	R.ALPFWNEEIVPQIK.E	W5(+31.985329844)
31.9899	0.9376182	P19367	171	1	K.IDEAILITWTK.R	W9(+31.987318889)
31.9899	0.9376182	Q13885	21	1	K.FWEVISDEHGIDPTGSYHGDSDLQLER.I	W2(+31.989078498)
31.9899	0.9376182	P14618	515	1	K.KGDVVIVLTGWR.P	W11(+31.987873273)
31.9899	0.9376182	O60716	830	1	R.AAALVLQTIWGYK.E	W10(+31.986936792)
31.9899	0.9376182	Q13813	456	1	R.AALLELWELR.R	W7(+31.987020811)
31.9899	0.9376182	P02675	415	1	R.DNDGWLTSDPR.K	W5(+31.987512666)
31.9899	0.9376182	Q02817	557	1	K.TASGLVEATGAGFANTWK.A	W17(+31.9882283)
31.9899	0.9376182	Q93050	479	1	K.SLNIFGSSWSVR.P	W9(+31.984985756)
31.9899	0.9376182	P08133	192	1	K.WGTDEAQFIYILGNR.S	W1(+31.98810566)
31.9899	0.9376182	Q13491	197	1	R.QYGIIPWNAFPGK.I	W7(+31.988075838)
31.9899	0.9376182	Q92752	1093	1	K.ELIVDAEDTWIR.L	W10(+31.987902742)
31.9899	0.9376182	P12110	702	1	K.NLEWIAGGTWTPSALK.F	W10(+31.988009816)
31.9899	0.9376182	Q01082	2119	1	K.VSEEAESQQQWDTSK.G	W11(+31.986362756)
31.9899	0.9376182	P04746	34	1	R.TSIVHLFEWR.W	W9(+31.985675838)
31.9899	0.9376182	Q13813	237	1	K.TKQDEVNAAWQR.L	W10(+31.985855325)
31.9899	0.9376182	P52565	192	1	R.FTDDDKTDHLSWEWNLTIK.K	W12(+31.980615459)
31.9899	0.9376182	Q12860	845	1	R.YWAAHDKEEAANR.V	W2(+31.985046548)
31.9899	0.9376182	Q01082	1926	1	R.DLMLWMEDVIR.Q	W5(+31.987810098)

31.9899	0.9376182	P05023	906	1	R.WINDVEDSYGQQWTYEQR.K	W13(+31.985408088)
31.9899	0.9376182	P09327	22	1	K.GSLNITTPGLQIWR.I	W13(+31.989455502)
31.9899	0.9376182	P49327	896	1	R.VLFPATGYLSIVWK.T	W13(+31.992848348)
31.9899	0.9376182	Q9Y490	2389	1	K.VGAIPANALDDGQWSQGLISAAR.M	W14(+31.986454838)
31.9899	0.9376182	P36578	67	1	R.QPYAVSELAGHQTSAESWGTGR.A	W18(+31.985459411)
31.9899	0.9376182	P17858	132	1	R.SEWGSLLEELVAEGK.I	W3(+31.985964362)
31.9899	0.9376182	O75390	333	1	R.DYIWNTLNSGR.V	W4(+31.987826991)
31.9899	0.9376182	O60832	52	1	K.LDTSQWPLLLK.N	W6(+31.991109864)
31.9899	0.9376182	Q9UQM7	403	1	R.FYFENLWSR.N	W7(+31.990345024)
31.9899	0.9376182	O75489	239	1	K.FDLNSPWEAFPVYR.Q	W7(+31.991604397)
31.9899	0.9376182	P18206	253	1	R.VLQLTSWDEDAWASK.D	W7(+31.994683866)
31.9899	0.9376182	Q16851	333	1	K.IFNTNNLWISLAAVK.R	W8(+31.986211784)
31.9899	0.9376182	P04899	259	1	K.WFTDTSIILFLNKK.D	W1(+31.9830441)
31.9899	0.9376182	P35573	680	1	K.WNPEALPSNTGEVNFQSGIIAAR.C	W1(+31.987922951)
31.9899	0.9376182	P05023	894	1	R.WINDVEDSYGQQWTYEQR.K	W1(+31.989314338)
31.9899	0.9376182	Q8WZ42	15031	1	K.WTVPEKDGGSPITNYIVEKR.D	W1(+31.989857747)
31.9899	0.9376182	Q15257	122	1	R.WIDETPPVDQPSR.F	W1(+31.989891867)
31.9899	0.9376182	P01011	217	1	K.WEMPFDPQDTHQSR.F	W1(+31.990033703)
31.9899	0.9376182	Q8IXS6	52	1	K.WLLQGIPAGTAEEEEAR.R	W1(+31.990209206)
31.9899	0.9376182	Q9C0C2	1532	1	R.WSDQGPAQTSR.R	W1(+31.990271648)
31.9899	0.9376182	Q8WZ42	31798	1	K.WISVTTEEIR.E	W1(+31.990364182)
31.9899	0.9376182	Q86YV0	403	1	R.WFPLLGAPAGAALR.A	W1(+31.990540551)
31.9899	0.9376182	A8K7I4	163	1	R.WGVFDEYNNDEK.F	W1(+31.990655523)
31.9899	0.9376182	Q15149	1325	1	R.WQAVLAQTDVR.Q	W1(+31.9907897)
31.9899	0.9376182	P21333	2632	1	K.WGDEHIPGSPYR.V	W1(+31.99121018)
31.9899	0.9376182	P31040	121	1	R.WHFYDTVK.G	W1(+31.991330412)
31.9899	0.9376182	P25098	517	1	R.WQQEVAETVFDTINAETDRLEAR.K	W1(+31.99161012)
31.9899	0.9376182	Q9Y5K5	58	1	K.WQPGEEPAGSVVQDSR.L	W1(+31.991708521)
31.9899	0.9376182	P09210	21	1	R.WLLAAAGVEFEEK.F	W1(+31.992268068)
31.9899	0.9376182	Q8WZ42	15749	1	K.WEPPLDDGGSEIINYTLEK.K	W1(+31.993274216)
31.9899	0.9376182	P48643	324	1	R.WVGGPEIELIAIATGGR.I	W1(+31.994028424)
31.9899	0.9376182	P00558	383	1	K.WNTEDKVSHVSTGGGASLELLEGK.V	W1(+31.99539338)
31.9899	0.9376182	Q05707	463	1	K.WDAVPGASGYLILYAPLTEGLAGDEK.E	W1(+31.995494788)
31.9899	0.9376182	Q9Y2J8	348	1	R.WIQDEIEFGYIEAPHK.G	W1(+31.99642322)
31.9899	0.9376182	P55265	1043	1	R.WNVLGLQGALLTHFLQPIYLK.S	W1(+31.99703216)
31.9899	0.9376182	P50579	166	1	K.ALDQASEEIWNDFR.E	W10(+31.982445306)
31.9899	0.9376182	Q96KP4	34	1	K.WVAIQSVSAWPEKR.G	W10(+31.985415087)
31.9899	0.9376182	P02100	131	1	K.EFTPEVQAAWQK.L	W10(+31.987572804)
31.9899	0.9376182	P06396	71	1	K.AGKEPGLQIWR.V	W10(+31.988692816)
31.9899	0.9376182	P40616	172	1	K.GTGLDEAMEWLVETLK.S	W10(+31.989033793)
31.9899	0.9376182	P02545	467	1	K.SNEDQSMGNWQIK.R	W10(+31.989133492)
31.9899	0.9376182	Q96S55	521	1	R.GSDQNASLYWLAR.M	W10(+31.989283009)
31.9899	0.9376182	P07954	141	1	K.LNDHFPLVVWQTGSGTQTNMNVNEVISNR.A	W10(+31.989753486)
31.9899	0.9376182	P0C0L5	1288	1	K.AEMADQAAAWLTR.Q	W10(+31.989822853)
31.9899	0.9376182	O15061	290	1	K.ATLTLAMADWLR.D	W10(+31.989941945)
31.9899	0.9376182	Q9Y2S2	207	1	R.LQYAIISEAWR.L	W10(+31.990016865)
31.9899	0.9376182	P28838	186	1	K.LYGSGDQEAWQK.G	W10(+31.990072891)
31.9899	0.9376182	Q8WZ42	15864	1	K.DNGSPILGYWLEKR.E	W10(+31.990091487)
31.9899	0.9376182	O75438	57	1	R.ELQPSEEVTWK.-	W10(+31.990189328)
31.9899	0.9376182	P10644	262	1	K.VSILESLDKWER.L	W10(+31.990257011)
31.9899	0.9376182	O00159	284	1	K.VSSINDKSDWK.V	W10(+31.990271316)
31.9899	0.9376182	P27824	455	1	R.IVDDWANDGWGLK.K	W10(+31.990304066)
31.9899	0.9376182	O43776	501	1	R.EGIDPTPYYWYTDQR.K	W10(+31.990415545)
31.9899	0.9376182	P26640	33	1	R.YGEAGEGPGWGGAHPR.I	W10(+31.990516711)
31.9899	0.9376182	P20701	410	1	R.AGYLGYTVTWLPSR.Q	W10(+31.990526688)
31.9899	0.9376182	Q15181	189	1	K.PGYLEATVDWFR.R	W10(+31.990910277)
31.9899	0.9376182	Q9Y5B9	902	1	K.YTEGVQSLNWTK.I	W10(+31.991071267)
31.9899	0.9376182	P43487	67	1	R.FASENDLPEWK.E	W10(+31.991160588)
31.9899	0.9376182	Q9Y6N5	315	1	K.TSPVADAAGWVDVDK.E	W10(+31.991446921)
31.9899	0.9376182	Q9NTI5	137	1	R.YFYLLENIAWVK.S	W10(+31.991640611)
31.9899	0.9376182	O00203	639	1	K.ATGYLELSNWPEVAPDPSVR.N	W10(+31.991666114)
31.9899	0.9376182	Q9Y3I0	318	1	K.GMAAAGNYAWVNR.S	W10(+31.991722041)
31.9899	0.9376182	Q9H8Y8	90	1	R.ETSVTPSNLWGGQGLLGVSIR.F	W10(+31.991746595)
31.9899	0.9376182	P25786	13	1	R.NQYDNDVTVWSPQGR.I	W10(+31.991820876)
31.9899	0.9376182	P05108	286	1	K.ADIYTQNFYWELR.Q	W10(+31.991832026)
31.9899	0.9376182	Q9UKV3	1044	1	R.TGTLVEEAFWIDK.I	W10(+31.992159854)
31.9899	0.9376182	O76094	474	1	K.EAISDLQQLWK.Q	W10(+31.99228783)
31.9899	0.9376182	P28838	114	1	K.AAGIDEQENWHEGKENIR.A	W10(+31.993458539)
31.9899	0.9376182	P15085	191	1	R.EWVTQASGVWFAK.K	W10(+31.993877127)

31.9899	0.9376182	P33121	535	1	K.TAEALDKDGWLHTGDIGK.W	W10(+31.993939715)
31.9899	0.9376182	P52179	759	1	R.NTDTSVVVSWEESKDAK.E	W10(+31.993983934)
31.9899	0.9376182	P10745	1206	1	R.SVGASDGSSWEGVGVTPHVVVPAEEALAR.A	W10(+31.997940397)
31.9899	0.9376182	Q99798	574	1	R.LQLLEPFDKWDGKDLEDLQILIK.V	W10(+31.997960045)
31.9899	0.9376182	O60610	179	1	R.VSLNNNPVSWVQTFGAEGLASLLDILKR.L	W10(+31.998751851)
31.9899	0.9376182	Q53GS9	325	1	K.QGDGVDFLSWFLNALHSALGGTK.K	W10(+31.999109737)
31.9899	0.9376182	O95782	462	1	R.IAGDYVSEEVWYR.V	W11(+31.987093965)
31.9899	0.9376182	P98082	364	1	R.TGKQEAQAGPWPFSSSQTQPAVR.T	W11(+31.987583314)
31.9899	0.9376182	P62736	81	1	K.YPIEHGIITNWDDMEK.I	W11(+31.987911317)
31.9899	0.9376182	Q16851	75	1	R.FLQEKGPSVDWGK.I	W11(+31.988120932)
31.9899	0.9376182	Q8N8S7	80	1	K.YNQATQTFHQWR.D	W11(+31.988499601)
31.9899	0.9376182	P63267	80	1	K.YPIEHGIITNWDDMEK.I	W11(+31.988521669)
31.9899	0.9376182	P04433	55	1	R.ASQSVSSYLAWYQQKPGQAPR.L	W11(+31.989329094)
31.9899	0.9376182	P35609	180	1	R.NVNIQNFHTSWK.D	W11(+31.98945345)
31.9899	0.9376182	Q8IY81	529	1	K.AVLQEEQANLWFSK.G	W11(+31.989584632)
31.9899	0.9376182	Q08426	531	1	R.VSDLAGLDVGWK.S	W11(+31.989728766)
31.9899	0.9376182	Q9BV20	37	1	R.YEAVGSVHQAWEAIR.A	W11(+31.990554733)
31.9899	0.9376182	Q8WZ42	20734	1	K.EISKDSAYVTWEPPIIDGGSPIINYVVQKR.D	W11(+31.990562838)
31.9899	0.9376182	Q12860	829	1	K.VLSSSEISVHWEHVLEK.I	W11(+31.990669408)
31.9899	0.9376182	Q8N163	108	1	K.AAYNPGQAVPWNAVK.V	W11(+31.990995538)
31.9899	0.9376182	Q8WZ42	1589	1	R.ATGNPNPDIVWLK.N	W11(+31.99119158)
31.9899	0.9376182	Q8WZ42	9210	1	R.VAGSQPITVAWYK.N	W11(+31.991242871)
31.9899	0.9376182	O94973	461	1	R.IAGDYVSEEVWYR.V	W11(+31.991366426)
31.9899	0.9376182	Q15392	303	1	K.LNSIGNYYKPWFFK.H	W11(+31.991574004)
31.9899	0.9376182	P43405	284	1	R.PQLPGSHPATWSAGGIISR.I	W11(+31.991596795)
31.9899	0.9376182	P24043	512	1	K.SGFFNLQEDNWK.G	W11(+31.991819038)
31.9899	0.9376182	P55157	69	1	R.ISSNVDVALLWR.N	W11(+31.992209718)
31.9899	0.9376182	Q6P2Q9	927	1	K.ITDAYLDQYLWYEADKR.R	W11(+31.993401004)
31.9899	0.9376182	Q99715	2332	1	K.ADIVFLTDASWSIGDDNFNK.V	W11(+31.995829721)
31.9899	0.9376182	P09327	710	1	K.QGHEPPTFTGWFLAWDPFK.W	W11(+31.997196379)
31.9899	0.9376182	Q9BUQ8	452	1	K.TAAFLIPLLVWITTLPK.I	W11(+31.997370274)
31.9899	0.9376182	Q8N5N7	86	1	K.EVFGSSLPSNWQDISLEDSR.L	W11(+31.998936387)
31.9899	0.9376182	Q8WX93	1168	1	K.VSGLPTPDLSWQLDGKPVRPDSAHK.M	W11(+32.002350825)
31.9899	0.9376182	O15075	646	1	R.FSAVQVLEHPWVNDDGLPENEHQLSVAGK.I	W11(+32.006753384)
31.9899	0.9376182	Q9UPR5	691	1	K.TNLALVIGTHSWR.E	W12(+31.984511956)
31.9899	0.9376182	Q96PQ0	980	1	K.ELDAYNPNTPEWR.E	W12(+31.988864014)
31.9899	0.9376182	P32418	744	1	K.TNLALVVGTNSWR.E	W12(+31.988924756)
31.9899	0.9376182	P27797	236	1	K.IDDPTDSKPEDWDKPEHIPDPDAK.K	W12(+31.989941783)
31.9899	0.9376182	Q9H2U2	237	1	K.FKPGYLEATLNWFR.L	W12(+31.990404621)
31.9899	0.9376182	Q8WVY7	236	1	R.GLIDVKPLGVIWGK.F	W12(+31.990805012)
31.9899	0.9376182	P56556	97	1	K.GKIELEETIKVWK.Q	W12(+31.991454853)
31.9899	0.9376182	P07814	1103	1	K.THVADFAPEVAWVTR.S	W12(+31.991826727)
31.9899	0.9376182	Q15393	1011	1	R.VIVSDVQESFIWVR.Y	W12(+31.991870351)
31.9899	0.9376182	Q9BPU6	405	1	R.IIPGADADVVVWDPEATK.T	W12(+31.992412297)
31.9899	0.9376182	Q53GG5	13	1	M.PQTVILPGPAPWGFR.L	W12(+31.992662352)
31.9899	0.9376182	P00403	163	1	R.MMITSQDVLHSWAVPTLGLK.T	W12(+31.994939804)
31.9899	0.9376182	Q8NCN5	329	1	K.NQLEIQNLQEDWDHFEPLLSSLLR.R	W12(+31.996319866)
31.9899	0.9376182	Q14315	2338	1	R.GVAGVPAEFSIWTR.E	W12(+31.997479393)
31.9899	0.9376182	P24821	913	1	R.VSQTDNSITLEWR.N	W12(+31.997725018)
31.9899	0.9376182	Q9ULU8	443	1	K.LQTDQAEASKPTWGTQGDFSTTHALPAVK.V	W13(+31.985192304)
31.9899	0.9376182	P04746	331	1	R.GHGAGGASILTFWDAR.L	W13(+31.98646568)
31.9899	0.9376182	P15259	78	1	R.TLWAILDGTDQMWLPVVR.T	W13(+31.987151206)
31.9899	0.9376182	P37235	30	1	R.ENTEFTDHELQEWYK.G	W13(+31.98803371)
31.9899	0.9376182	Q8WUP2	63	1	K.TPEAGLAGRPSPWTTPGR.A	W13(+31.98902122)
31.9899	0.9376182	P01911	90	1	R.AVTELGRPDAEYWNSQK.D	W13(+31.989280103)
31.9899	0.9376182	Q9H0U3	104	1	K.QADEEFQILANSWR.Y	W13(+31.989507115)
31.9899	0.9376182	P10745	1123	1	K.IYSRPDDSVSELWTHAQVVGER.Y	W13(+31.990147041)
31.9899	0.9376182	Q02880	140	1	K.VSIDPESNIISIWNNGK.G	W13(+31.990317671)
31.9899	0.9376182	P61619	324	1	R.FSGNLLVSLLGTWSDTSSGGPAR.A	W13(+31.99068247)
31.9899	0.9376182	Q15063	142	1	K.GSFTYFAPSNEAWDNLDSDIR.R	W13(+31.990925462)
31.9899	0.9376182	Q14697	623	1	R.FGAVWTGDNTAEWDHLK.I	W13(+31.99106014)
31.9899	0.9376182	P07384	187	1	K.LVFVHSAEGNEFWSALLEK.A	W13(+31.992067932)
31.9899	0.9376182	Q92530	146	1	R.IVSGIITPIHEQWEK.A	W13(+31.992376904)
31.9899	0.9376182	Q9H479	289	1	R.LLLYQLFNYLNHWNHFGR.E	W13(+31.993085839)
31.9899	0.9376182	P27797	261	1	K.KPEDWDEEMDGEWEPPVIQNPEYK.G	W13(+31.995024183)
31.9899	0.9376182	Q99715	2597	1	R.LLPETPSDPFAIWQITDR.D	W13(+31.996657664)
31.9899	0.9376182	Q8WZ42	27604	1	K.LPYTTPGPPSTPWVTNVTR.E	W13(+32.002756516)
31.9899	0.9376182	Q9NYU2	764	1	K.EIYDDSFIRPVTFWIVGDFDSPSGR.Q	W14(+31.986348101)
31.9899	0.9376182	Q12840	342	1	K.NTASVNLELTAEQWK.K	W14(+31.987813149)

31.9899	0.9376182	P04792	51	1	R.LPEEWSQWLGGSSWPGYVRPLPPAAIESPAVAAPAYSR.A	W14(+31.988187647)
31.9899	0.9376182	P62256	51	1	K.FYGPQGTPYEGGVWK.V	W14(+31.989125828)
31.9899	0.9376182	P17858	458	1	K.GQVQEVGWHDVAGWLGR.G	W14(+31.990176777)
31.9899	0.9376182	Q9BWM7	68	1	R.AGVVTPGITEDQLWR.A	W14(+31.990404345)
31.9899	0.9376182	P52565	194	1	R.FTDDDKTDHLSWEWNLTIK.K	W14(+31.990869366)
31.9899	0.9376182	Q15149	507	1	K.VPPGYHPLDVEKEWGK.L	W14(+31.992495432)
31.9899	0.9376182	P42224	539	1	K.LLGPNASPDGLIPWTR.F	W14(+31.992899424)
31.9899	0.9376182	P50502	187	1	R.AIEINPDSAQPYKWR.G	W14(+31.992928629)
31.9899	0.9376182	P09093	42	1	R.VVHGEDAVPYSWPWQVSLQYEK.S	W14(+31.993494201)
31.9899	0.9376182	P26439	230	1	K.FSTVNPVYVGNVAWAHILALR.A	W14(+31.996587542)
31.9899	0.9376182	P34932	587	1	K.TSTVDLPIENQLLWQIDREMLNLYIENEGK.M	W14(+32.010509807)
31.9899	0.9376182	P61326	77	1	R.IIDDSEITKEDDALWPPPDR.V	W15(+31.986389621)
31.9899	0.9376182	Q00325	160	1	K.VLYSNMLGEENTYLWR.T	W15(+31.986891422)
31.9899	0.9376182	Q96A72	79	1	R.IIDDSEITKEDDALWPPPDRVGR.Q	W15(+31.986988248)
31.9899	0.9376182	Q09666	3800	1	K.VDINAPDVDVQGPDWHLK.M	W15(+31.988215061)
31.9899	0.9376182	Q09666	1250	1	K.MDIDAPDVEVQGPDWHLK.M	W15(+31.988782048)
31.9899	0.9376182	Q8WXD2	218	1	K.EANNYEEDPNKPTSWTENQAGK.I	W15(+31.989426688)
31.9899	0.9376182	Q09666	2762	1	K.VDIDAPDVDVHGPDWHLK.M	W15(+31.989447542)
31.9899	0.9376182	Q09666	624	1	K.VDVSAPDVEAHGPEWNLK.M	W15(+31.990826922)
31.9899	0.9376182	P19835	508	1	K.TGDPNMGDSAVPTHWEPYTTENSGYLEITK.K	W15(+31.990836409)
31.9899	0.9376182	Q14839	790	1	K.GPFLVSAPLSTIINWER.E	W15(+31.991751553)
31.9899	0.9376182	Q92616	1511	1	K.LVLPSLLAALEEESWR.T	W15(+31.991901607)
31.9899	0.9376182	Q09666	4776	1	K.VDINTPDVDVHGPDWHLK.M	W15(+31.991951175)
31.9899	0.9376182	Q13813	1004	1	K.KGDILTLLNSTNKDWWK.V	W15(+31.993421914)
31.9899	0.9376182	Q8NCW5	223	1	K.GLTVPIASIDIPSGWDVEK.G	W15(+31.993431325)
31.9899	0.9376182	P36957	89	1	K.TPAFAESVTEGDVRWEK.A	W15(+31.994060594)
31.9899	0.9376182	Q15029	709	1	K.GLAEDIENEVVQITWNR.K	W15(+31.994704248)
31.9899	0.9376182	Q5VYK3	1206	1	R.GRPLDDIIDKLPEIWETLFR.V	W15(+31.994763246)
31.9899	0.9376182	Q09666	4184	1	K.VDIDVPDVDVQGPDWHLK.M	W15(+31.995116145)
31.9899	0.9376182	Q9BY49	280	1	R.SLYTHSYEVPDHDNWPK.G	W15(+31.995156877)
31.9899	0.9376182	Q09666	2890	1	K.VDIDVPDVNVQGPDWHLK.M	W15(+31.997686417)
31.9899	0.9376182	O43895	247	1	K.VPTAVLLSALEETAWLFNLR.A	W15(+31.998729612)
31.9899	0.9376182	Q99832	271	1	R.VHTVEDYQAIVDAEWNILYDKLEK.I	W15(+31.999327083)
31.9899	0.9376182	P20916	59	1	R.FDFPDELRPAVVHGVWYFNSPYPK.N	W16(+31.986805722)
31.9899	0.9376182	Q96SK2	273	1	K.LGSPDSTSPSSSPTFWNYSR.S	W16(+31.987582958)
31.9899	0.9376182	P51178	121	1	R.NTLDLIAPSPADAQHWVLGLHK.I	W16(+31.990732877)
31.9899	0.9376182	P00403	65	1	K.LTNTNISDAQEMETVWTILPAIILVLIALPSLR.I	W16(+31.990899519)
31.9899	0.9376182	Q9Y3Z3	285	1	K.EQIVGPLESPVEDSLWPYK.G	W16(+31.991731783)
31.9899	0.9376182	P80365	66	1	R.LLPPPAALAVLAAAGWIALSR.L	W16(+31.992023435)
31.9899	0.9376182	Q93052	326	1	R.NDSDPTYGQQGHPNTWK.R	W16(+31.992186815)
31.9899	0.9376182	P52566	191	1	K.SFFTDDDKQDHLSWEWNLSIK.K	W16(+31.993444668)
31.9899	0.9376182	Q99715	2128	1	R.TVGLLPPQNIHISDEWYTR.F	W16(+31.995131818)
31.9899	0.9376182	Q9H7Z7	314	1	R.LQDNVREDLYEAADKWVAAVGK.D	W16(+31.995969864)
31.9899	0.9376182	Q13813	1005	1	K.KGDILTLLNSTNKDWWK.V	W16(+31.997450235)
31.9899	0.9376182	P06744	513	1	K.IFVQGIIWDINSFDQWGVELGK.Q	W16(+32.002217217)
31.9899	0.9376182	Q9BY11	305	1	R.STSGPGMPMNWPQFEEWNPDLPHTTTK.K	W17(+31.987611843)
31.9899	0.9376182	P52907	120	1	K.EASDPQPEEADGGLKSWR.E	W17(+31.988667415)
31.9899	0.9376182	P00738	133	1	K.LRTEGDGVYTLNNEKQWINK.A	W17(+31.991795263)
31.9899	0.9376182	P07954	500	1	K.ETAIELGYLTAEQFDEWVKPK.D	W17(+31.991821172)
31.9899	0.9376182	Q9Y6D6	1044	1	K.TLITVAHTDGNYLGNSWHEILK.C	W17(+31.99315985)
31.9899	0.9376182	P55786	321	1	K.IDLIAIADFAAGAMENWGLVTYR.E	W17(+31.997456747)
31.9899	0.9376182	P49588	215	1	R.DAAHLVNQDDPNVLEIWNLVFIQYNR.E	W17(+31.997971389)
31.9899	0.9376182	Q14204	278	1	K.LDRDPASGTALQEISFWLNLER.A	W17(+31.998693125)
31.9899	0.9376182	P39060	1654	1	R.AAVPIVNLKDELLFPSWEALFSGSEGPLKPGAR.I	W17(+32.000552828)
31.9899	0.9376182	O75569	140	1	K.NQLNPIGSLQELAIHHGWR.L	W18(+31.990504689)
31.9899	0.9376182	Q9UBG0	1315	1	R.AGGAVLSILDEMENVFVWEHLQSYEGQSR.G	W18(+31.99202323)
31.9899	0.9376182	O60664	416	1	R.EALDHMVEYVAQNTPVTWLVGPFAPGITEK.A	W18(+31.992977053)
31.9899	0.9376182	Q7L0J3	416	1	K.TIHQEDELIEIQSDTGTWYQR.W	W18(+31.993143633)
31.9899	0.9376182	P16885	145	1	K.ILHQEAMNASTPTIIESWLR.K	W18(+31.993343501)
31.9899	0.9376182	O95372	40	1	R.ETAAVIFLHGLGDTGHSWADALSTIR.L	W18(+31.996427191)
31.9899	0.9376182	Q641Q2	23	1	R.TTPDQELAPASEPVWERPWSVEEIR.R	W19(+31.971460855)
31.9899	0.9376182	Q9BQI5	694	1	K.YQVSAQGIQSTPLNLAVNWR.C	W19(+31.986591658)
31.9899	0.9376182	Q13424	392	1	R.HGVDTHLFSVESPQELAAWTR.Q	W19(+31.98940959)
31.9899	0.9376182	Q14247	22	1	K.ASAGHAVSIAQDDAGADDWETDPDFVNDVSEKEQR.W	W19(+31.995848871)
31.9899	0.9376182	P11532	2738	1	K.QWQDLQGEIEAHTDVYHNLDENSQK.I	W2(+31.984668949)
31.9899	0.9376182	Q27J81	1047	1	R.GWDLVDAVTPGPQPTLEQLEEGGPRPLER.R	W2(+31.985095066)
31.9899	0.9376182	Q15293	272	1	R.HWILPQDYDHAQAEAR.H	W2(+31.985662572)
31.9899	0.9376182	P04217	475	1	R.SWVPHTFESELSDPVELLVAES.-	W2(+31.988017256)
31.9899	0.9376182	O00151	89	1	K.VWSPLVTEEGKR.H	W2(+31.988065501)

31.9899	0.9376182	Q86UE4	6	1	R.SWQDELAQQAEEGSAR.L	W2(+31.98898554)
31.9899	0.9376182	Q15436	313	1	R.SWHDIDKDNAK.Y	W2(+31.988987379)
31.9899	0.9376182	P46940	1098	1	K.SWVNQMESQTGEASK.L	W2(+31.989336947)
31.9899	0.9376182	P49888	27	1	K.YWDNVEAFQARPDDLVIATYPK.S	W2(+31.989423753)
31.9899	0.9376182	P43034	135	1	K.VWDYETGDFER.T	W2(+31.989480808)
31.9899	0.9376182	Q9Y3Z3	598	1	K.EWNDSTSVQNPTR.L	W2(+31.989595274)
31.9899	0.9376182	Q9H0U4	102	1	K.QWLQEIDR.Y	W2(+31.989687795)
31.9899	0.9376182	Q00839	816	1	K.PWSQHYHQGYY.-	W2(+31.989714969)
31.9899	0.9376182	P27694	414	1	R.GWFDAEGQALDGVSISDLK.S	W2(+31.989887217)
31.9899	0.9376182	P14314	198	1	K.LWEEQLAAAK.A	W2(+31.989921889)
31.9899	0.9376182	Q9BZK7	196	1	R.IWNLSENSTSGSTQLVLR.H	W2(+31.989932919)
31.9899	0.9376182	Q8WZ42	17590	1	R.AWTPVTYTVTR.Q	W2(+31.989979703)
31.9899	0.9376182	Q14204	2275	1	R.EWTDGLFTHVLR.K	W2(+31.990008911)
31.9899	0.9376182	O15382	221	1	R.AWVGGVGNYK.L	W2(+31.990074929)
31.9899	0.9376182	O43399	97	1	R.SWHDVQVSSAYVK.T	W2(+31.990122152)
31.9899	0.9376182	P30084	120	1	K.HWDHLTQVK.K	W2(+31.990367169)
31.9899	0.9376182	Q15393	720	1	R.SWLSYSYQSR.F	W2(+31.99055173)
31.9899	0.9376182	P15924	1342	1	R.RWEYENELSK.V	W2(+31.990602748)
31.9899	0.9376182	P20273	585	1	K.AWTLEVLYAPR.R	W2(+31.990703571)
31.9899	0.9376182	P01889	157	1	R.SWTAADTAAQITQR.K	W2(+31.990823935)
31.9899	0.9376182	P46776	79	1	K.LWTLVSEQTR.V	W2(+31.99134754)
31.9899	0.9376182	Q02543	45	1	R.FWYFVSQLK.K	W2(+31.991521174)
31.9899	0.9376182	Q8WZ42	16065	1	K.VWTKEEGELDKDR.V	W2(+31.991760653)
31.9899	0.9376182	O60701	300	1	R.YWQQVIDMNDYQR.R	W2(+31.991965305)
31.9899	0.9376182	P15085	183	1	R.EWVTQASGVWFAK.K	W2(+31.992534354)
31.9899	0.9376182	Q8IYI6	273	1	R.EWLEVLEDTKR.A	W2(+31.992608053)
31.9899	0.9376182	P09455	107	1	R.GWTQWIEGDELHLEMR.V	W2(+31.993231425)
31.9899	0.9376182	Q92922	43	1	K.FWESPETVSQLDSVR.V	W2(+31.995095792)
31.9899	0.9376182	P11277	396	1	R.AWESLEEAEYRR.E	W2(+32.000893544)
31.9899	0.9376182	Q13576	1011	1	K.AWVNQLETQTGEASKLPYDVTTEQALTYPEVK.N	W2(+32.00282831)
31.9899	0.9376182	Q8TED1	181	1	K.FWKPEEPIEVIRPDIAALVR.Q	W2(+32.006789827)
31.9899	0.9376182	Q15084	412	1	R.GSTAPVGGGAFPTIVEREPWDGR.D	W20(+31.988213398)
31.9899	0.9376182	P07942	731	1	K.SLDIFTVGGSGDGVVTNSAWETFQR.Y	W20(+31.989029852)
31.9899	0.9376182	Q16363	460	1	R.ELVDEEADEAYELLSQAESWQR.L	W20(+32.005534034)
31.9899	0.9376182	Q14194	291	1	K.GPLVFGEPIAASLGTDGTHYWSK.N	W21(+31.98300498)
31.9899	0.9376182	O00468	1454	1	R.FDTGSGPAVLTSAVPVEPGQWHR.L	W21(+31.991556727)
31.9899	0.9376182	Q9H4G4	109	1	K.NYNFQQPGFTSGTGHFTAMVWK.N	W21(+31.99367377)
31.9899	0.9376182	Q8WXX5	97	1	R.AVYDEQGTVDEDSPVLTQDRDWEAYWR.L	W22(+31.992543708)
31.9899	0.9376182	Q14697	888	1	R.FSFSGNTLVSSSADPEGHFETPIWIER.V	W24(+32.005415861)
31.9899	0.9376182	P31939	508	1	R.AEISNAIDQYVTGTIGEDEDLIKWK.A	W24(+32.007141341)
31.9899	0.9376182	Q9BVA1	101	1	R.SGPFGQIFRPDNFVFGQSGAGNNWAK.G	W24(+32.012387495)
31.9899	0.9376182	Q02952	804	1	K.SEDSIAGSGVEHSTPDTEPGKEESWVSIK.K	W25(+31.986709298)
31.9899	0.9376182	Q9Y4W6	779	1	K.STYEEFVEGTGSLDEDTSLPEGLKDWNKER.E	W26(+31.990589996)
31.9899	0.9376182	Q92523	392	1	R.ILDDPSPPQPGEEKLAALTAGGRVEWAQAR.Q	W26(+31.991559493)
31.9899	0.9376182	P48449	273	1	R.LSAAEDPLVQSLRQELYVEDFASIDWLAQR.N	W26(+32.006495673)
31.9899	0.9376182	P46821	475	1	K.DKAEFILPNGQEVDLPISYLTSVSSLIVWHPANPAEK.I	W29(+32.019198961)
31.9899	0.9376182	P78559	1751	1	R.SPWASDFKDFQESSPQKGLEVER.W	W3(+31.985757191)
31.9899	0.9376182	Q8WZ42	1874	1	K.VNWYLNGQLIR.K	W3(+31.985838491)
31.9899	0.9376182	Q01082	437	1	R.ETWLSENQR.L	W3(+31.987575165)
31.9899	0.9376182	Q99715	1675	1	R.GTWDHGASDVSLYR.I	W3(+31.987700235)
31.9899	0.9376182	Q96G03	178	1	K.VYWDNGAQIISPHDK.G	W3(+31.988277859)
31.9899	0.9376182	Q8WZ42	25776	1	R.LSWTQVSTEVQALNYK.V	W3(+31.988526448)
31.9899	0.9376182	Q8WZ42	22529	1	R.LAWTNVASEVQVTK.L	W3(+31.988587418)
31.9899	0.9376182	P50895	485	1	K.LSWSQLGGSPAEPIPGR.Q	W3(+31.989022379)
31.9899	0.9376182	Q15424	798	1	R.DGWGGYGSDKR.M	W3(+31.989072588)
31.9899	0.9376182	P15311	43	1	R.EVWYFGLHYVDNK.G	W3(+31.989167403)
31.9899	0.9376182	O43169	42	1	K.ELWLVIHGR.V	W3(+31.989189203)
31.9899	0.9376182	Q9UHB6	532	1	R.IAWPPPTELGSSGSALEEGIK.M	W3(+31.989244138)
31.9899	0.9376182	P46821	2412	1	K.AQWGSNMQVTLIPTHDSEVMR.E	W3(+31.989309878)
31.9899	0.9376182	Q99715	1251	1	R.TEWQLNAHR.D	W3(+31.989534199)
31.9899	0.9376182	Q14697	687	1	R.EPWLLPSQHNDIIR.D	W3(+31.989666624)
31.9899	0.9376182	Q9NYU2	815	1	R.AIWAALQTQTSNAAK.N	W3(+31.989763627)
31.9899	0.9376182	P55087	30	1	K.GVWTQAFWK.A	W3(+31.989822236)
31.9899	0.9376182	O15061	865	1	R.YSWQDEIVQGTR.R	W3(+31.990008635)
31.9899	0.9376182	P12532	251	1	R.GIWHNNEK.S	W3(+31.990384547)
31.9899	0.9376182	Q04446	596	1	R.YGWLAAPQAYVSEK.H	W3(+31.990422886)
31.9899	0.9376182	Q6UVY6	145	1	R.VIWAYHHEDAGEAGPK.Y	W3(+31.990635228)
31.9899	0.9376182	P09466	166	1	R.HLWYLLDLK.Q	W3(+31.990703844)
31.9899	0.9376182	P01889	75	1	R.APWIEQEGPEYWDR.N	W3(+31.990762598)

31.9899	0.9376182	Q8IZ83	120	1	R.LLWTLESLVTGR.A	W3(+31.990948602)
31.9899	0.9376182	P49588	156	1	K.QIWQNLGLDDTK.I	W3(+31.991255564)
31.9899	0.9376182	P49675	241	1	K.LTWLLSIDLK.G	W3(+31.991409708)
31.9899	0.9376182	Q9BSJ8	1043	1	R.FEWELPLDEAQR.R	W3(+31.991433066)
31.9899	0.9376182	Q07954	3190	1	K.ITWPNGLTLDYVTER.I	W3(+31.991929922)
31.9899	0.9376182	Q8NB12	28	1	K.EFWAADIIFAER.A	W3(+31.991983846)
31.9899	0.9376182	P04083	12	1	K.QAWFIENEEQEYVQTVK.S	W3(+31.9922136)
31.9899	0.9376182	P50895	502	1	R.QGWVSSSLTLK.V	W3(+31.992480953)
31.9899	0.9376182	P43405	136	1	K.QTWNLQGQALEQAIISQKPQLEK.L	W3(+31.992913378)
31.9899	0.9376182	Q9UIA9	495	1	R.LTWLVYIIGAVIGGR.V	W3(+31.993670443)
31.9899	0.9376182	Q99623	74	1	R.IPWFQYPIIYDIR.A	W3(+31.993713776)
31.9899	0.9376182	Q8WZ42	17779	1	K.VSWFKDEADVLEDDRTHIK.T	W3(+31.99400018)
31.9899	0.9376182	O43815	70	1	R.AQWEVERAELQAQIAFLQGER.K	W3(+31.994019423)
31.9899	0.9376182	P14927	81	1	K.EQWTKYEEENFYLEPYLKEVIR.E	W3(+31.994471368)
31.9899	0.9376182	P18669	68	1	R.TLWTVLDAIDQMWLPVVR.T	W3(+31.995706482)
31.9899	0.9376182	Q99715	354	1	K.LNWNPSPSPVTGYK.V	W3(+31.995906334)
31.9899	0.9376182	Q13228	177	1	K.GTWERPGGAAPLGYDFWYQPR.H	W3(+31.996359346)
31.9899	0.9376182	P27487	563	1	R.LNWATYLASTENIIVASFDGR.G	W3(+31.99694606)
31.9899	0.9376182	P16233	419	1	K.FIWYNNVINPTLPR.V	W3(+31.997621247)
31.9899	0.9376182	P00488	665	1	R.NVWVHLDGPGVTRPMK.K	W3(+31.997900472)
31.9899	0.9376182	O00178	237	1	R.TSSVGNDILGFDSEGNVVNKPDSHGGSLEWTK.I	W30(+31.990189846)
31.9899	0.9376182	P27797	319	1	K.GTWIHPEIDNPEYSPDPSIYAYDNFGVLGLDLWQVK.S	W33(+32.003816936)
31.9899	0.9376182	P68133	342	1	K.YSVWIGGSILASLSTFQQMWITKQEYDEAGPSIVHR.K	W4(+31.971998824)
31.9899	0.9376182	P11233	149	1	R.AEQWNVNYVETSAK.T	W4(+31.982033379)
31.9899	0.9376182	O14594	1158	1	R.DFQWTDNTGLQFENWR.E	W4(+31.987611532)
31.9899	0.9376182	P26373	25	1	R.VATWFNQPAR.K	W4(+31.988388979)
31.9899	0.9376182	P62304	34	1	R.IQVWLYEQVNMR.I	W4(+31.988433496)
31.9899	0.9376182	Q8WZ42	16473	1	K.KAEWEEVTNTAVEKR.Y	W4(+31.989286549)
31.9899	0.9376182	P20036	199	1	R.VEHWGLDQPLLK.H	W4(+31.989377037)
31.9899	0.9376182	P51148	75	1	K.FEIWDTAGQER.Y	W4(+31.98965125)
31.9899	0.9376182	P62491	65	1	K.AQIWDTAGQER.Y	W4(+31.989952314)
31.9899	0.9376182	P62820	65	1	K.LQIWDTAGQER.F	W4(+31.989999192)
31.9899	0.9376182	Q8WZ42	18578	1	R.KDTWGVVSSGSSK.T	W4(+31.990266904)
31.9899	0.9376182	Q9NUU7	347	1	K.TASWLAAELSK.E	W4(+31.990343531)
31.9899	0.9376182	P60842	287	1	R.KVDWLTEK.M	W4(+31.990516002)
31.9899	0.9376182	P68400	176	1	R.LIDWGLAEFYHPGQEYNVR.V	W4(+31.990604309)
31.9899	0.9376182	Q9HC52	42	1	K.YSTWEPEENILDAR.L	W4(+31.990811233)
31.9899	0.9376182	Q9P258	354	1	R.VFSWGFGGYGR.L	W4(+31.990890229)
31.9899	0.9376182	P12036	376	1	R.NTKWEMAAQLR.E	W4(+31.991071276)
31.9899	0.9376182	Q13683	123	1	K.ENQWLGVSVR.S	W4(+31.991088382)
31.9899	0.9376182	P36405	66	1	K.LNVWDIGGQR.K	W4(+31.991277092)
31.9899	0.9376182	O60488	502	1	K.LKDWQEGGYTINDKPNPR.G	W4(+31.991888746)
31.9899	0.9376182	Q9Y5U9	33	1	K.NIGWGTDQGIGGFGEEPGIK.S	W4(+31.992081563)
31.9899	0.9376182	Q15181	92	1	K.GYIWNYGAIPQTWEDPGHNDK.H	W4(+31.992334861)
31.9899	0.9376182	P05455	303	1	K.EVTWEVLEGEVEK.E	W4(+31.992986626)
31.9899	0.9376182	Q15008	63	1	K.SLDWQIDVDLLNK.M	W4(+31.993097224)
31.9899	0.9376182	Q9C0C9	1145	1	R.IESWLETHALLEK.A	W4(+31.993100449)
31.9899	0.9376182	P63261	340	1	K.YSVWIGGSILASLSTFQQMWISKQEYDESGPSIVHR.K	W4(+32.001035047)
31.9899	0.9376182	P30711	84	1	K.VPDYWYPQDLQAR.A	W5(+31.987407128)
31.9899	0.9376182	P13726	57	1	K.TILEWEPKPVNQVYTVQISTK.S	W5(+31.988719272)
31.9899	0.9376182	Q96AC1	252	1	K.INQGWLDSSR.S	W5(+31.98914381)
31.9899	0.9376182	P02768	238	1	R.AFKAWAVAR.L	W5(+31.989210998)
31.9899	0.9376182	P26641	419	1	R.EYFSWEGAFQHVGK.A	W5(+31.989263247)
31.9899	0.9376182	Q9UJU6	28	1	K.SPTDWALFTYEGNSNDIR.V	W5(+31.989826212)
31.9899	0.9376182	P33176	360	1	R.NTIQWLENELNR.W	W5(+31.989985954)
31.9899	0.9376182	Q15661	44	1	R.SKWPWQVSLR.V	W5(+31.99026027)
31.9899	0.9376182	P31327	143	1	K.DYNHWLATK.S	W5(+31.990542287)
31.9899	0.9376182	P48681	898	1	R.SLGAWNLENLR.S	W5(+31.990570624)
31.9899	0.9376182	P00491	16	1	K.NTAEWLLSHTK.H	W5(+31.990769618)
31.9899	0.9376182	O95208	411	1	K.NSDPWAASQQPASSAGKR.A	W5(+31.990817538)
31.9899	0.9376182	Q01105	95	1	K.IPNFWVTTFVNHPQVSALLGEEDEEALHYLTR.V	W5(+31.990891328)
31.9899	0.9376182	O95219	265	1	R.VFSEWSAIEK.E	W5(+31.990891656)
31.9899	0.9376182	O15231	487	1	K.VGEAWQDRPGAPR.G	W5(+31.990920356)
31.9899	0.9376182	Q9NRN5	387	1	R.QLYAWDDGYQIVYK.L	W5(+31.991017616)
31.9899	0.9376182	P17174	351	1	K.TPGTWNHITDQIGMFSFTGLNPK.Q	W5(+31.991120898)
31.9899	0.9376182	P09327	177	1	K.LIIQWNGPESTR.M	W5(+31.991181865)
31.9899	0.9376182	P55884	320	1	R.TSIFWNDVKDPVSIEER.A	W5(+31.99127093)
31.9899	0.9376182	P22914	163	1	R.KPIDWGAASPAVQSFR.R	W5(+31.99144993)
31.9899	0.9376182	Q8IZ83	593	1	R.AALLWALAAALER.R	W5(+31.991510318)

31.9899	0.9376182	P23141	547	1	K.EVAFWTNLFAK.K	W5(+31.991739978)
31.9899	0.9376182	Q96P48	883	1	R.AQEGWFSLSGSELR.A	W5(+31.991810213)
31.9899	0.9376182	Q6P2Q9	1375	1	R.YIQPWESEFIDSQR.V	W5(+31.991988603)
31.9899	0.9376182	Q96SL4	159	1	K.VVGAWDPTVSVEEVRPQITALVR.K	W5(+31.99208367)
31.9899	0.9376182	Q03518	221	1	K.LGSLWVPGGQGGSGNPVR.R	W5(+31.992462492)
31.9899	0.9376182	Q8WXH0	3800	1	K.STEAWIENTSHLLANPADYDSLR.T	W5(+31.992925195)
31.9899	0.9376182	Q6UWY5	383	1	K.QLYAWNEGNQIIYK.L	W5(+31.993064827)
31.9899	0.9376182	Q14624	793	1	K.NPLVWVHASPEHVVVTR.N	W5(+31.993399837)
31.9899	0.9376182	P14625	333	1	K.TVWDWELMNDIKPIWQRPSK.E	W5(+31.993928791)
31.9899	0.9376182	Q14152	393	1	K.DLYNWLEVEFNPLK.L	W5(+31.994116251)
31.9899	0.9376182	P09455	110	1	R.GWTQWIEGDELHLEMR.V	W5(+31.994879375)
31.9899	0.9376182	O43674	168	1	R.GDGPWYYYETIDKELIDHSPK.A	W5(+31.99528628)
31.9899	0.9376182	Q8NBJ5	462	1	R.EGLDWDLIYVGR.K	W5(+31.995360239)
31.9899	0.9376182	Q96N66	176	1	R.TYLDWLEQPFPGAVPSLRPLLR.R	W5(+31.999334712)
31.9899	0.9376182	P24043	2208	1	K.VSFLWDVGSGVGR.V	W5(+31.999487059)
31.9899	0.9376182	P13591	54	1	K.DKDISWFSPNGEK.L	W6(+31.986655812)
31.9899	0.9376182	Q92835	597	1	R.VDLPTWEAETIIQK.I	W6(+31.987462228)
31.9899	0.9376182	P08238	312	1	K.SLTNDWEDHLAVK.H	W6(+31.988182883)
31.9899	0.9376182	Q9UPY8	23	1	R.HDMLAWVNDSLHLNYTK.I	W6(+31.988482781)
31.9899	0.9376182	Q9NZN9	278	1	R.AHAEVWNEAEAK.A	W6(+31.988639908)
31.9899	0.9376182	Q02487	826	1	R.YTYSEWHSFTQPR.L	W6(+31.988649354)
31.9899	0.9376182	O43324	74	1	K.AIVQQWLEYR.V	W6(+31.989009936)
31.9899	0.9376182	Q13087	428	1	K.EMAPAWEALAEK.Y	W6(+31.989026984)
31.9899	0.9376182	P05108	126	1	R.FLIPPWVAYHQYYQRPIGVLLK.K	W6(+31.989154215)
31.9899	0.9376182	Q9ULV4	138	1	R.VGIVAWHPTAR.N	W6(+31.989222824)
31.9899	0.9376182	P15259	16	1	R.HGESTWNQENR.F	W6(+31.989351737)
31.9899	0.9376182	Q15029	549	1	R.VPAGNWVLIEGVDQPIVK.T	W6(+31.98943752)
31.9899	0.9376182	P26639	50	1	R.AELNPWPEYIYTR.L	W6(+31.989711115)
31.9899	0.9376182	Q15084	200	1	K.NLEPEWAAAASEVKEQTK.G	W6(+31.989814087)
31.9899	0.9376182	Q15382	141	1	K.ALAESWNAAFLESSAK.E	W6(+31.989867693)
31.9899	0.9376182	P54289	752	1	K.EAGENWQENPETYEDSFYKR.S	W6(+31.989873666)
31.9899	0.9376182	P09172	302	1	R.HVLAAWALGAK.A	W6(+31.990018631)
31.9899	0.9376182	P08514	983	1	R.GEAQVWTQLLR.A	W6(+31.990154284)
31.9899	0.9376182	P50993	1013	1	R.RYPGGWVEK.E	W6(+31.990259226)
31.9899	0.9376182	P14317	303	1	K.ISSEAWPPVGTPPSSESEPVR.T	W6(+31.990276713)
31.9899	0.9376182	Q13418	355	1	R.MYAPAWVAPEALQK.K	W6(+31.990309046)
31.9899	0.9376182	P02675	432	1	K.EDGGGWWYNR.C	W6(+31.9903427)
31.9899	0.9376182	Q14019	81	1	K.FALITWIGENVSGLQR.A	W6(+31.990428541)
31.9899	0.9376182	P05165	284	1	K.HGNALWLNER.E	W6(+31.991110527)
31.9899	0.9376182	Q9UL25	73	1	R.VNLAIWDTAGQER.F	W6(+31.991308076)
31.9899	0.9376182	Q9UMY4	77	1	R.YSDFEWLKNELER.D	W6(+31.991344314)
31.9899	0.9376182	P0DJI9	71	1	R.GPGGAWAAEVISNAR.E	W6(+31.99160914)
31.9899	0.9376182	Q9NQR4	219	1	K.ASYVAWGHSTVVNPWGEVLAK.A	W6(+31.99193853)
31.9899	0.9376182	Q9UJU6	84	1	K.FVLINWTGEGVNDVR.K	W6(+31.992419696)
31.9899	0.9376182	Q9HA64	193	1	R.EALQLWSALQLK.I	W6(+31.993145867)
31.9899	0.9376182	Q5QJ74	242	1	K.SGLQSWEDIDKLNSFPKLEEVR.L	W6(+31.993820045)
31.9899	0.9376182	Q99459	739	1	K.QLNDLWDQIEQAHLELR.T	W6(+31.995440244)
31.9899	0.9376182	P05108	270	1	K.DHVAAWDVIFSK.A	W6(+31.99610387)
31.9899	0.9376182	Q16853	335	1	R.VASSLWTFSFGLGAFSGPR.I	W6(+31.996486069)
31.9899	0.9376182	P13010	675	1	K.QLNHFWEIVVQDGITLITK.E	W6(+31.996852871)
31.9899	0.9376182	Q9P2R3	693	1	K.GNPPLWLALANNLEDIASTLVR.H	W6(+31.998379669)
31.9899	0.9376182	Q9BWS9	155	1	R.LLFEDWTYDDFRNVLDSEDEIEELSK.T	W6(+32.000460215)
31.9899	0.9376182	Q09666	5597	1	K.GSGGEWKGPQVSSALNLDTSKFAGGLHFSGPK.V	W6(+32.007149075)
31.9899	0.9376182	Q8WZ42	30485	1	R.DSVTITWEIPTIDGGAPVNNYIVEKR.E	W7(+31.983912636)
31.9899	0.9376182	P35609	531	1	R.AAPFNNWMEGAMEDLQDMFIVHSIEEIQSLITAHEQFK.A	W7(+31.984004064)
31.9899	0.9376182	Q7Z3D6	558	1	R.APGDQAWTQALPSVIKEEK.M	W7(+31.987340255)
31.9899	0.9376182	P27694	361	1	K.VVTATLWGEDADKFDGSR.Q	W7(+31.987342867)
31.9899	0.9376182	Q8WZ42	24297	1	R.DTTTTVWDVVSATVAR.T	W7(+31.987746509)
31.9899	0.9376182	Q92736	2290	1	K.GYPDIGWNPVEGER.Y	W7(+31.987806956)
31.9899	0.9376182	P21397	472	1	K.VTEKDIWVQEPESK.D	W7(+31.987857643)
31.9899	0.9376182	P62273	8	1	M.GHQQLYWSHPR.K	W7(+31.988099034)
31.9899	0.9376182	P38606	209	1	K.FTMVQVWPVR.Q	W7(+31.988290157)
31.9899	0.9376182	P00846	48	1	R.LITTQQWLIK.L	W7(+31.988654602)
31.9899	0.9376182	O95741	54	1	K.LYSDEQWVEVER.T	W7(+31.988664368)
31.9899	0.9376182	Q14515	293	1	K.HIQETEWQSQEGK.T	W7(+31.988787659)
31.9899	0.9376182	Q8WZ42	29886	1	R.STVSLIWSAPAYDGGSK.V	W7(+31.988921497)
31.9899	0.9376182	Q5XKP0	110	1	R.EYSKEGWEYVK.A	W7(+31.988968625)
31.9899	0.9376182	Q8NFD5	1499	1	R.INHESQWPSHVSQR.Q	W7(+31.989011486)
31.9899	0.9376182	Q16134	297	1	R.VDHTVGWPLDR.H	W7(+31.989178593)

31.9899	0.9376182	P13591	555	1	R.AVGEEVWHSK.W	W7(+31.989254944)
31.9899	0.9376182	Q9P2R7	294	1	K.IFDLQDWTQEDERDKDAAK.A	W7(+31.989286911)
31.9899	0.9376182	O43707	428	1	K.ASIHEAWTDGK.E	W7(+31.989478161)
31.9899	0.9376182	Q8WZ42	29493	1	K.SSAVLAWLKPDHDGGSR.I	W7(+31.989886299)
31.9899	0.9376182	P98172	37	1	K.NLEPVSWSSLNPK.F	W7(+31.989984733)
31.9899	0.9376182	Q9BZZ5	443	1	K.STVTLSWKPVQK.V	W7(+31.990119174)
31.9899	0.9376182	Q99798	373	1	K.VAEKEGWPLDIR.V	W7(+31.990628977)
31.9899	0.9376182	Q8WZ42	30806	1	R.DAHRPGWLPVSESVTR.S	W7(+31.990638803)
31.9899	0.9376182	P30711	101	1	R.VDEYLAWQHTTLR.R	W7(+31.990690932)
31.9899	0.9376182	Q9NTX5	290	1	R.DLLGTVWGGPANLEAIAK.K	W7(+31.990696253)
31.9899	0.9376182	Q8WZ42	26146	1	K.DSVTLSWEPPLIDGGAK.I	W7(+31.990757854)
31.9899	0.9376182	Q8WZ42	22899	1	K.TSVTLTWDPPLLDGGSK.I	W7(+31.99085486)
31.9899	0.9376182	Q8WZ42	18283	1	R.DVASAQWSPLSATSK.K	W7(+31.991142937)
31.9899	0.9376182	Q96S19	54	1	R.AFPLAEWQPSDVDQR.C	W7(+31.991410112)
31.9899	0.9376182	P09936	26	1	R.LGVAGQWR.F	W7(+31.991520615)
31.9899	0.9376182	Q8WZ42	28410	1	K.TSVILSWTKPDFDGGSVITEYVVER.K	W7(+31.991591491)
31.9899	0.9376182	P50148	263	1	R.TIITYPWFQNSSVILFLNKK.D	W7(+31.991733574)
31.9899	0.9376182	O15537	122	1	K.FQDSSQWLQIDLK.E	W7(+31.991744735)
31.9899	0.9376182	Q01082	151	1	R.LTLGLIWTIILR.F	W7(+31.991899505)
31.9899	0.9376182	P53396	49	1	R.LLQDHPWLLSQNLVVKPDQLIK.R	W7(+31.991917602)
31.9899	0.9376182	P09874	246	1	K.AQNDLIWNIKDELKK.V	W7(+31.991941151)
31.9899	0.9376182	P61158	86	1	R.HGIVEDWDLMER.F	W7(+31.991953479)
31.9899	0.9376182	Q8N4P3	149	1	R.VQEYFEWAAQVVK.G	W7(+31.991984736)
31.9899	0.9376182	Q9H4M9	238	1	R.VYGALMWSLGK.I	W7(+31.992102321)
31.9899	0.9376182	Q14204	4185	1	R.LYFLLAWFHAIIQER.L	W7(+31.992209743)
31.9899	0.9376182	Q9H4A4	283	1	K.LFGPYVWGR.Y	W7(+31.992549358)
31.9899	0.9376182	O43707	147	1	K.MTLGMIWTIILR.F	W7(+31.993120753)
31.9899	0.9376182	Q02487	517	1	K.LTDPTGWVTIDENTGSIK.V	W7(+31.993229354)
31.9899	0.9376182	Q13907	144	1	K.AQSDGIWGEHEIDYILLVR.K	W7(+31.993319032)
31.9899	0.9376182	Q9NZN3	258	1	R.VYIGSFWSHPLLIPDNR.K	W7(+31.993988044)
31.9899	0.9376182	Q9C0C2	1281	1	R.GVGQADWTPDLGLR.N	W7(+31.994237763)
31.9899	0.9376182	O75147	749	1	R.VDFPATWYKDGQKVEESELLVVK.M	W7(+31.994471641)
31.9899	0.9376182	P45985	95	1	K.ISPEQHWDFTAEDLKDLGEIGR.G	W7(+31.994921929)
31.9899	0.9376182	Q9BQG0	40	1	R.EFLDFFWDIAKPEQETR.L	W7(+31.997446388)
31.9899	0.9376182	Q9NZN4	258	1	R.VYIGSFWSQPLLVPDNRR.L	W7(+31.998705181)
31.9899	0.9376182	Q14697	711	1	R.YSLLPFWYTLLYQAHR.E	W7(+32.005391321)
31.9899	0.9376182	Q14204	4308	1	R.REEFVQWVELLPDTQTPSWLGLPNNAER.V	W7(+32.010829221)
31.9899	0.9376182	P61204	66	1	K.NISFTVWDVGGQDK.I	W7(+32.020750592)
31.9899	0.9376182	O43865	428	1	R.SQVDHVIWPDGK.R	W8(+31.986164884)
31.9899	0.9376182	P07814	1027	1	K.KEENLADWYSQVITK.S	W8(+31.986816824)
31.9899	0.9376182	Q13813	799	1	R.DVEDEETWIREKEPIAASTNR.G	W8(+31.987028658)
31.9899	0.9376182	P02749	335	1	K.EHSSLAFWK.T	W8(+31.987444814)
31.9899	0.9376182	P12235	71	1	K.EQGFLSFWR.G	W8(+31.988117392)
31.9899	0.9376182	Q92598	675	1	R.LLTETEDWLYEEGEDQAK.Q	W8(+31.988143968)
31.9899	0.9376182	Q59GN2	26	1	K.QNRPIPQWIR.M	W8(+31.988599535)
31.9899	0.9376182	P12883	1371	1	K.ANSEVAQWR.T	W8(+31.988751645)
31.9899	0.9376182	P98160	2663	1	R.QPQAIITWYK.R	W8(+31.98887693)
31.9899	0.9376182	P17858	382	1	R.GGSFENNWNIYK.L	W8(+31.988981054)
31.9899	0.9376182	P08670	290	1	K.NLQEAEEWYK.S	W8(+31.989110457)
31.9899	0.9376182	Q14697	566	1	K.DAQHYGGWEHR.D	W8(+31.989305135)
31.9899	0.9376182	P17858	452	1	K.GQVQEVGWHDVAGWLGR.G	W8(+31.989444355)
31.9899	0.9376182	Q9Y315	14	1	R.GTELDLSWISK.I	W8(+31.98947783)
31.9899	0.9376182	P49368	435	1	K.AMTGVEQWPYR.A	W8(+31.989481979)
31.9899	0.9376182	P10515	498	1	K.VPEANSSWMDTVIR.Q	W8(+31.990123314)
31.9899	0.9376182	P07942	160	1	R.SSDFGKTWGVYR.Y	W8(+31.990296535)
31.9899	0.9376182	O15020	1709	1	R.ELDDLEQWIQER.E	W8(+31.990516074)
31.9899	0.9376182	Q9NP72	176	1	K.IIQTPGLWESENQNK.G	W8(+31.990682992)
31.9899	0.9376182	Q9UDY2	754	1	K.LANELPDWFQTAK.T	W8(+31.990814161)
31.9899	0.9376182	O94804	36	1	R.DLDPNEVWEIVGELGDGAFGK.V	W8(+31.991343486)
31.9899	0.9376182	P02751	2399	1	K.TYHVGEQWQK.E	W8(+31.991779229)
31.9899	0.9376182	P09496	146	1	K.AIKELEEWYAR.Q	W8(+31.991848489)
31.9899	0.9376182	P26640	417	1	R.EAFLQEVWK.W	W8(+31.991854363)
31.9899	0.9376182	P27701	140	1	R.EDSLQDAWDYVQAQVK.C	W8(+31.992200612)
31.9899	0.9376182	P26639	512	1	K.FLGDIEVWDQAEK.Q	W8(+31.992230634)
31.9899	0.9376182	Q9NR12	155	1	R.LMENTEDWRPRPGTGQSR.S	W8(+31.992298578)
31.9899	0.9376182	P07451	47	1	R.HDPSLQPWSVSYDGGSAK.T	W8(+31.992930114)
31.9899	0.9376182	Q13162	88	1	K.ISKPAPYWEGTAVIDGEFK.E	W8(+31.993932575)
31.9899	0.9376182	P42224	616	1	R.EGAITFTWVER.S	W8(+31.994113582)
31.9899	0.9376182	P07814	1273	1	K.QFAYQNSWGLTTR.T	W8(+31.994202984)

31.9899	0.9376182	O60784	124	1	K.VLNLIQSWADAFR.S	W8(+31.994831008)
31.9899	0.9376182	P13796	496	1	R.TLTLALIWQLMR.R	W8(+31.995266456)
31.9899	0.9376182	O95831	579	1	K.VVVGIVLWNIFNR.M	W8(+31.995343759)
31.9899	0.9376182	P11277	2024	1	R.DASVAEAWLIAQEPYLASGDFGHTVDSVEK.L	W8(+31.996086071)
31.9899	0.9376182	Q12768	1073	1	R.KPTDPVDWPPLVLGLLTLLK.Q	W8(+31.997536297)
31.9899	0.9376182	P00966	74	1	R.EFVEEFIWPAIQSSALYEDR.Y	W8(+32.000180065)
31.9899	0.9376182	O43707	387	1	K.MVSDINNGWQHLEQAEK.G	W9(+31.985606171)
31.9899	0.9376182	P48735	284	1	K.TDFDKNKIWYEHR.L	W9(+31.988039005)
31.9899	0.9376182	Q14697	226	1	K.AEKDEPGAWEETFK.T	W9(+31.988761944)
31.9899	0.9376182	O60234	133	1	R.TTDDLTEAWLQEK.L	W9(+31.98905415)
31.9899	0.9376182	P46937	503	1	K.LDKESFLTWL.-	W9(+31.989082415)
31.9899	0.9376182	P0C0L5	1069	1	R.KADGSYAAWLSR.G	W9(+31.989186275)
31.9899	0.9376182	P78347	564	1	R.TNTPVKEDWNVR.I	W9(+31.989275176)
31.9899	0.9376182	P35609	375	1	K.MVSDIAGAWQR.L	W9(+31.989329542)
31.9899	0.9376182	A0A0C4DH38	102	1	K.SISTAYLQWSSLK.A	W9(+31.989359032)
31.9899	0.9376182	Q9P2E9	1156	1	K.SVEEEEQVWR.A	W9(+31.989425377)
31.9899	0.9376182	Q8WZ42	27147	1	R.GRPVPNVLWSKPDTDLR.T	W9(+31.989457632)
31.9899	0.9376182	Q15819	33	1	K.GVGDGTVSWGLEDDEDMTLTR.W	W9(+31.989525398)
31.9899	0.9376182	P35241	445	1	K.KKEEEATEWQHK.A	W9(+31.989660696)
31.9899	0.9376182	Q13268	233	1	K.VFHGNESLWK.N	W9(+31.989699893)
31.9899	0.9376182	Q8N126	62	1	K.DHEDSSLQWSNPAQQTLYFGEK.R	W9(+31.989922927)
31.9899	0.9376182	Q9UNF0	62	1	K.AYAQQLTEWAR.R	W9(+31.990162838)
31.9899	0.9376182	P22234	140	1	K.DDANNDPQWSEEQLIAAK.F	W9(+31.990744688)
31.9899	0.9376182	Q15149	3167	1	R.GANVIAGVWLEEAGQK.L	W9(+31.990770556)
31.9899	0.9376182	O75531	62	1	K.KDEDLFREWLK.D	W9(+31.990775961)
31.9899	0.9376182	P98160	669	1	R.QVQFSEEHWVHESGR.P	W9(+31.991619185)
31.9899	0.9376182	Q9UKS6	60	1	K.AYAQQLADWAR.K	W9(+31.991841455)
31.9899	0.9376182	Q13308	547	1	R.ADGSSLPEWVTDNAGTLHFAR.V	W9(+31.992192458)
31.9899	0.9376182	P00352	77	1	R.QAFQIGSPWR.T	W9(+31.992783511)
31.9899	0.9376182	P46821	441	1	K.EMQYFMQQWTGTNK.D	W9(+31.992901727)
31.9899	0.9376182	Q14204	4387	1	R.TLHTTASNWLHLIPQTLSHLK.R	W9(+31.99300888)
31.9899	0.9376182	Q9NXG2	187	1	K.YAETFLEPWFK.A	W9(+31.993228392)
31.9899	0.9376182	O75643	1222	1	K.VHGSSEAFWILVEDVDSEVILHHEYFLLK.A	W9(+31.999759048)
31.9899	0.9376182	Q15063	531	1	K.ELLTQPGDWTLFVPTNDAFK.G	W9(+32.004307408)
67.0058	0.3471842	O94906	619	1	K.WLAGDVPAAR.S	W1(+67.003931101)
67.0058	0.3471842	Q9UQM7	462	1	K.WQIVHFHR.S	W1(+67.004482065)
67.0058	0.3471842	P60201	145	1	K.WLGHPDK.F	W1(+67.004658022)
67.0058	0.3471842	P04406	87	1	K.WGDAGAEYVVESTGVFTTMEK.A	W1(+67.005025349)
67.0058	0.3471842	P02689	98	1	R.WDGKETTIK.R	W1(+67.005094258)
67.0058	0.3471842	P08133	192	1	K.WGTDEAQFIYILGNR.S	W1(+67.005439645)
67.0058	0.3471842	P01889	268	1	K.WAAVVVPSGEEQR.Y	W1(+67.005447581)
67.0058	0.3471842	P09543	380	1	R.WMLTLAK.N	W1(+67.005614346)
67.0058	0.3471842	P07355	213	1	K.WISIMTER.S	W1(+67.005659789)
67.0058	0.3471842	P01876	264	1	R.WLQGSQELPR.E	W1(+67.006200037)
67.0058	0.3471842	P50453	171	1	K.WNEPFDETYTR.E	W1(+67.007731511)
67.0058	0.3471842	P33241	267	1	R.WETGEVQAQSAAK.T	W1(+67.008012815)
88.9942	0.1672632	P05386	43	1	K.AAGVNVEPFWPGLFAK.A	W10(+88.9975938)
88.9942	0.1672632	P63261	86	1	K.IWHHTFYNELR.V	W2(+88.988241576)
88.9942	0.1672632	P00738	284	1	R.VGYVSGWGR.N	W7(+88.9914124)
88.9942	0.1672632	P00738	398	1	K.VTSIQDWVQK.T	W7(+88.992017736)
92.0277	0.0010505	P00338	227	1	K.TLHPDLGTDKDKEQWK.E	W15(+92.025437633)
103.0088	0.1239659	O15498	110	1	K.QVDRIDWPVGSPATIHYPALDGHLSR.Y	W7(+103.010529569)
103.0088	0.1239659	Q15021	757	1	K.PAVTQLLWER.A	W8(+103.003881863)
195.1041	0.1317696	Q6V1X1	383	1	R.AGWTPEGK.Y	W3(+195.1043849)
209.0179	0.7899121	Q9H1V8	63	1	K.AVEEELDAEDRPAWNSK.L	W14(+209.018756237)
209.0179	0.7899121	P63261	86	1	K.IWHHTFYNELR.V	W2(+209.017538451)
209.0179	0.7899121	Q9BVA1	21	1	K.FWEVISDEHGIDPTGSYHGDSDLQLER.I	W2(+209.033586413)
209.0179	0.7899121	P35237	173	1	R.GNWDEQFDKENTEER.L	W3(+209.014645939)
209.0179	0.7899121	Q9P1Y5	923	1	R.KQWQEVEKEQR.R	W3(+209.017260986)
209.0179	0.7899121	P04406	313	1	K.LISWYDNEFGYSNR.V	W4(+209.015664942)
209.0179	0.7899121	Q9UH03	93	1	K.ASSWNREEK.I	W4(+209.016963743)
209.0179	0.7899121	P83731	51	1	R.QINWTVLYR.R	W4(+209.020208918)
209.0179	0.7899121	O00499	571	1	K.ESDWNQHKELEK.C	W4(+209.020383219)
209.0179	0.7899121	P21333	345	1	R.TFSVWYVPEVTGTHK.V	W5(+209.022493488)
209.0179	0.7899121	P07738	16	1	R.HGEGAWNKENR.F	W6(+209.017478313)
209.0179	0.7899121	P13591	555	1	R.AVGEEVWHSK.W	W7(+209.016842835)
209.0179	0.7899121	P68871	38	1	R.LLVVYPWTQR.F	W7(+209.02142637)
209.0179	0.7899121	P68871	16	1	K.SAVTALWGKVNVDEVGGEALGR.L	W7(+209.033343413)

209.0179	0.7899121	P07437	344	1	K.NSSYFVEWIPNNVK.T	W8(+209.019516923)
209.0179	0.7899121	P30086	55	1	K.NRPTSISWDGLDSGK.L	W8(+209.020341647)
209.0179	0.7899121	O15394	639	1	R.SKDKEDQWLEK.K	W8(+209.022035552)
209.0179	0.7899121	P18124	142	1	R.IVEPYIAWGYPNLK.S	W8(+209.024737904)
209.0179	0.7899121	P05093	220	1	K.DSLVDLVPWLK.I	W9(+209.019856232)
209.0179	0.7899121	P15311	445	1	R.RKEDEVEEWQHR.A	W9(+209.020680745)
209.0179	0.7899121	P60174	206	1	K.VVLAYEPVWAIGTGK.T	W9(+209.021564076)
246.1373	0.0980538	P05455	303	1	K.EVTWEVLEGEVEKEALKK.I	W4(+246.131536201)
312.1545	0.0060943	P30086	55	1	R.PTSISWDGLDSGK.L	W6(+312.154078467)
432.2146	0.0503961	P40925	218	1	K.EVGVYEALKDDSWLK.G	W13(+432.215724014)
32.9748	0.6928963	P00915	124	1	K.YSAELHVAHWNSAK.Y	W10(+32.972658765)
32.9748	0.6928963	P02100	131	1	K.EFTPEVQAAWQK.L	W10(+32.974877491)
32.9748	0.6928963	P07197	291	1	R.SQLESHSDQNMHQAEEWFK.C	W17(+32.975181901)
32.9748	0.6928963	P07437	344	1	K.NSSYFVEWIPNNVK.T	W8(+32.973252274)
32.9748	0.6928963	P12814	524	1	R.AAPFNNWMEGAMEDLQDTFIVHTIEEIQGLTTAHEQFK.A	W7(+32.981743137)
32.9748	0.6928963	P18206	394	1	K.KIDAAQNWLADPNGGPEGEEQIR.G	W8(+32.977399247)
32.9748	0.6928963	P21796	210	1	K.KLETAVNLAWTAGNSNTR.F	W10(+32.970647303)
32.9748	0.6928963	P60880	105	1	K.AWGNNQDGVVASQPAR.V	W2(+32.968767592)
32.9748	0.6928963	Q01082	2119	1	K.VSEEAESQQQWDTSKGEQVSQNGLPAEQGSPR.M	W11(+32.975073619)
32.9748	0.6928963	Q13885	344	1	K.NSSYFVEWIPNNVK.T	W8(+32.969101884)
32.9748	0.6928963	Q14896	683	1	R.LDVPISGDPAPTVIWQK.A	W15(+32.977852062)
32.9748	0.6928963	Q99715	2045	1	R.VFGETTNSLSVAWDHADGPVQQYR.I	W13(+32.975111469)
14.9827	0.2716954	P01876	162	1	R.DASGVTFTWTPSSGK.S	W9(+14.975846985)
14.9827	0.2716954	P33241	267	1	R.WETGEVQAQSAAK.T	W1(+14.972368283)
14.9827	0.2716954	Q15257	122	1	R.WIDETPPVDQPSR.F	W1(+14.974633078)
14.9827	0.2716954	P35611	370	1	K.WQIGEQEFEALMR.M	W1(+14.97466196)
14.9827	0.2716954	A8K7I4	163	1	R.WGVFDEYNNDEKFYLSNGR.I	W1(+14.975010271)
14.9827	0.2716954	P55072	454	1	R.WALSQSNPSALR.E	W1(+14.975210416)
14.9827	0.2716954	P21796	64	1	R.WTEYGLTFTEK.W	W1(+14.975617245)
14.9827	0.2716954	Q13367	644	1	K.ATGYQELPDWPEEAPDPSVR.N	W10(+14.971730579)
14.9827	0.2716954	P21796	210	1	K.KLETAVNLAWTAGNSNTR.F	W10(+14.97333285)
14.9827	0.2716954	Q01813	469	1	K.EIGWTDVGGWTGQGGSILGTK.R	W10(+14.97436335)
14.9827	0.2716954	P00918	123	1	K.YAAELHLVHWNTK.Y	W10(+14.974666405)
14.9827	0.2716954	P09972	314	1	R.ALQASALNAWR.G	W10(+14.974834762)
14.9827	0.2716954	Q13813	237	1	K.TKQDEVNAAWQR.L	W10(+14.9760897)
14.9827	0.2716954	P12110	702	1	K.NLEWIAGGTWTPSALK.F	W10(+14.976168996)
14.9827	0.2716954	O75390	438	1	R.ALGVLAQLIWSR.A	W10(+14.976318169)
14.9827	0.2716954	P27824	455	1	R.IVDDWANDGWGLK.K	W10(+14.976510121)
14.9827	0.2716954	P04746	149	1	R.DFPAVPYSGWDFNDGK.C	W10(+14.978523707)
14.9827	0.2716954	P78352	717	1	R.VIEDLSGPYIWVPAR.E	W11(+14.971825755)
14.9827	0.2716954	P06737	183	1	R.DGWQVEEADDWLR.Y	W11(+14.973466644)
14.9827	0.2716954	Q03395	96	1	R.ASLNAALYPPWR.G	W11(+14.974057514)
14.9827	0.2716954	P29401	257	1	R.GITGVEDKESWHGKPLPK.N	W11(+14.977370539)
14.9827	0.2716954	P14618	515	1	K.KGDVVIVLTGWR.P	W11(+14.977497296)
14.9827	0.2716954	P01624	55	1	R.ASQSVSSNLAWYQQKPGQAPR.L	W11(+14.977503623)
14.9827	0.2716954	O95741	191	1	K.TNEDQSDQLVWR.T	W11(+14.978197019)
14.9827	0.2716954	Q96PQ0	980	1	K.ELDAYNPNTPEWR.E	W12(+14.972506592)
14.9827	0.2716954	P12036	50	1	R.SAAGSSSGFHSWTR.T	W12(+14.974620274)
14.9827	0.2716954	Q96KP4	140	1	R.GSTDDKGPVAGWINALEAYQK.T	W12(+14.975513001)
14.9827	0.2716954	P27824	304	1	K.IPDPEAVKPDDWDEDAPAK.I	W12(+14.976613042)
14.9827	0.2716954	P01857	196	1	R.VVSVLTVLHQDWLNGK.E	W12(+14.976693251)
14.9827	0.2716954	P26038	168	1	R.VLEQHKLNKDQWEER.I	W12(+14.978097136)
14.9827	0.2716954	Q92530	146	1	R.IVSGIITPIHEQWEK.A	W13(+14.973042046)
14.9827	0.2716954	P37235	30	1	R.ENTEFTDHELQEWYK.G	W13(+14.973385273)
14.9827	0.2716954	P40925	218	1	K.EVGVYEALKDDSWLK.G	W13(+14.977553609)
14.9827	0.2716954	P13796	387	1	R.YPALHKPENQDIDWGALEGETR.E	W14(+14.975009131)
14.9827	0.2716954	P08237	459	1	K.GQIEEAGWSYVGGWTGQGGSK.L	W14(+14.97743901)
14.9827	0.2716954	P07237	128	1	K.EYTAGREADDIVNWLK.K	W14(+14.97839345)
14.9827	0.2716954	P61326	77	1	R.IIDDSEITKEDDALWPPPDR.V	W15(+14.97418259)
14.9827	0.2716954	P30041	82	1	K.LIALSIDSVEDHLAWSK.D	W15(+14.975962285)
14.9827	0.2716954	P17174	320	1	R.IVASTLSNPELFEEWTGNVK.T	W15(+14.976300272)
14.9827	0.2716954	P12036	292	1	R.AQLEGHAVQSTLQSEEWFR.V	W17(+14.977269194)
14.9827	0.2716954	Q7L0J3	416	1	K.TIHQEDELIEIQSDTGTWYQR.W	W18(+14.973673418)
14.9827	0.2716954	P61026	195	1	K.EPNSENVDISSGGGVTGWK.S	W18(+14.974103566)
14.9827	0.2716954	Q7Z406	50	1	R.GPSAGGGPGSGTSPQVEWTAR.R	W18(+14.974229513)
14.9827	0.2716954	P07437	21	1	K.FWEVISDEHGIDPTGTYHGDSDLQLDR.I	W2(+14.971452624)
14.9827	0.2716954	P06744	228	1	K.EWFLQAAK.D	W2(+14.97523245)
14.9827	0.2716954	P06396	396	1	K.NWRDPDQTDGLGLSYLSSHIANVER.V	W2(+14.975393691)
14.9827	0.2716954	P02675	296	1	R.KWDPYKQGFGNVATNTDGK.N	W2(+14.976848394)

14.9827	0.2716954	Q14315	577	1	R.AWGPGLETGQVGK.S	W2(+14.977965522)
14.9827	0.2716954	P18206	283	1	K.GWLRDPSASPGDAGEQAIR.Q	W2(+14.978347796)
14.9827	0.2716954	P37059	276	1	K.VASIQPGGFLTNIAGTSDKWEK.L	W20(+14.975985937)
14.9827	0.2716954	Q14195	291	1	K.GNVVFGEPITASLGIDGTHYWSK.N	W21(+14.97283276)
14.9827	0.2716954	Q13813	905	1	R.RQDLEDSLQAQQYFADANEAESWMR.E	W23(+14.97713066)
14.9827	0.2716954	Q02952	804	1	K.SEDSIAGSGVEHSTPDTEPGKEESWVSIK.K	W25(+14.974990548)
14.9827	0.2716954	P31146	379	1	R.KSDLFQEDLYPPTAGPDPALTAEEWLGGR.D	W25(+14.975087581)
14.9827	0.2716954	Q6L8Q7	225	1	K.EAKPGAAEPEVGVPSSLSPSSPSSSWTETDVEER.V	W26(+14.971466494)
14.9827	0.2716954	P12814	717	1	R.VGWEQLLTTIAR.T	W3(+14.97509481)
14.9827	0.2716954	Q01082	726	1	R.EQWANLEQLSAIR.K	W3(+14.975287388)
14.9827	0.2716954	P11021	604	1	K.IEWLESHQDADIEDFK.A	W3(+14.976728049)
14.9827	0.2716954	Q02252	56	1	K.SDKWIDIHNPATNEVIGR.V	W4(+14.972179995)
14.9827	0.2716954	P01023	178	1	R.IAQWQSFQLEGGLK.Q	W4(+14.972480933)
14.9827	0.2716954	Q9HC52	42	1	K.YSTWEPEENILDAR.L	W4(+14.974942092)
14.9827	0.2716954	P02511	60	1	R.APSWFDTGLSEMR.L	W4(+14.975020485)
14.9827	0.2716954	Q13263	351	1	R.FASWALESDNNTALLLSK.K	W4(+14.976651099)
14.9827	0.2716954	P35237	137	1	K.HINTWVAEK.T	W5(+14.973713095)
14.9827	0.2716954	P29762	88	1	R.SLATWENENK.I	W5(+14.974090506)
14.9827	0.2716954	Q71U36	407	1	R.AFVHWYVGEGMEEGEFSEAR.E	W5(+14.974220629)
14.9827	0.2716954	P05023	418	1	K.TSATWLALSR.I	W5(+14.974231743)
14.9827	0.2716954	Q02252	93	1	R.AFPAWADTSVLSR.Q	W5(+14.974396006)
14.9827	0.2716954	Q5SSJ5	136	1	K.TIPSWATLSASQLAR.A	W5(+14.974576779)
14.9827	0.2716954	P68363	407	1	R.AFVHWYVGEGMEEGEFSEAR.E	W5(+14.975916327)
14.9827	0.2716954	P00338	324	1	K.SADTLWGIQK.E	W6(+14.973004244)
14.9827	0.2716954	P13591	656	1	R.ALSSEWKPEIR.L	W6(+14.974253284)
14.9827	0.2716954	P07900	320	1	K.SLTNDWEDHLAVK.H	W6(+14.974511008)
14.9827	0.2716954	P18669	16	1	R.HGESAWNLENR.F	W6(+14.975650657)
14.9827	0.2716954	P63208	88	1	R.TDDIPVWDQEFLK.V	W7(+14.971753188)
14.9827	0.2716954	P55809	413	1	K.YGDLANWMIPGK.M	W7(+14.973450355)
14.9827	0.2716954	O43707	428	1	K.ASIHEAWTDGK.E	W7(+14.974707653)
14.9827	0.2716954	P13591	555	1	R.AVGEEVWHSK.W	W7(+14.974850648)
14.9827	0.2716954	P00738	398	1	K.VTSIQDWVQK.T	W7(+14.975416173)
14.9827	0.2716954	P06737	826	1	K.EYAQNIWNVEPSDLK.I	W7(+14.97639397)
14.9827	0.2716954	P04179	210	1	K.AIWNVINWENVTER.Y	W8(+14.971047676)
14.9827	0.2716954	Q15020	562	1	R.TEGSLEDWDIAVQK.T	W8(+14.972026533)
14.9827	0.2716954	P24539	218	1	K.EQEHMINWVEK.H	W8(+14.97337814)
14.9827	0.2716954	Q13813	1640	1	R.LAALADQWQFLVQK.S	W8(+14.973668018)
14.9827	0.2716954	P10809	68	1	R.TVIIEQSWGSPK.V	W8(+14.9744405)
14.9827	0.2716954	Q13813	1106	1	R.EANELQQWINEK.E	W8(+14.975024353)
14.9827	0.2716954	Q13813	1566	1	R.DVDEIEAWISEK.L	W8(+14.975243966)
14.9827	0.2716954	Q92598	675	1	R.LLTETEDWLYEEGEDQAK.Q	W8(+14.975692796)
14.9827	0.2716954	Q01082	2032	1	R.DASVAEAWLLGQEPYLSSR.E	W8(+14.975985044)
14.9827	0.2716954	Q96G03	22	1	R.LDQETAQWLR.W	W8(+14.976184595)
14.9827	0.2716954	P07196	279	1	K.NMQNAEEWFK.S	W8(+14.976488852)
14.9827	0.2716954	P08237	453	1	K.GQIEEAGWSYVGGWTGQGGSK.L	W8(+14.976706588)
14.9827	0.2716954	Q15819	33	1	K.GVGDGTVSWGLEDDEDMTLTR.W	W9(+14.973412116)
14.9827	0.2716954	P05091	93	1	R.AAFQLGSPWR.R	W9(+14.973719891)
14.9827	0.2716954	Q9Y277	210	1	K.IETSINLAWTAGSNNTR.F	W9(+14.974845439)
14.9827	0.2716954	P61019	100	1	R.DTFNHLTTWLEDAR.Q	W9(+14.975102818)
14.9827	0.2716954	P53999	110	1	K.GISLNPEQWSQLK.E	W9(+14.975398522)
14.9827	0.2716954	P23528	104	1	K.KEDLVFIFWAPESAPLK.S	W9(+14.975688069)
14.9827	0.2716954	O95831	351	1	K.ILPEYLSNWTMEK.V	W9(+14.976008815)
14.9827	0.2716954	Q13228	204	1	R.HNVMISTEWAAPNVLR.D	W9(+14.976834514)
14.9827	0.2716954	P68371	101	1	R.SGPFGQIFRPDNFVFGQSGAGNNWAK.G	W24(+14.980025576)
14.9827	0.2716954	P01857	160	1	K.FNWYVDGVEVHNAK.T	W3(+14.981316582)
14.9827	0.2716954	P35573	680	1	K.WNPEALPSNTGEVNFQSGIIAAR.C	W1(+14.979133888)
14.9827	0.2716954	P10745	1206	1	R.SVGASDGSSWEGVGVTPHVVVPAEEALAR.A	W10(+14.980301237)
14.9827	0.2716954	P68032	81	1	K.YPIEHGIITNWDDMEK.I	W11(+14.98107538)
14.9827	0.2716954	Q16555	412	1	R.IAVGSDADLVIWDPDSVK.T	W12(+14.978576777)
14.9827	0.2716954	P35613	255	1	K.SESVPPVTDWAWYK.I	W12(+14.979084202)
14.9827	0.2716954	Q9BY49	280	1	R.SLYTHSYEVPDHDNWPK.G	W15(+14.982535886)
14.9827	0.2716954	P46977	687	1	K.DFELDVLEEAYTTEHWLVR.I	W16(+14.984530872)
14.9827	0.2716954	P0DPI2	204	1	R.GVEVTVGHEQEEGGKWPYAGTAEAIK.A	W16(+14.98683908)
14.9827	0.2716954	P06396	642	1	R.AQPVQVAEGSEPDGFWEALGGK.A	W16(+14.986946035)
14.9827	0.2716954	P0DOY2	79	1	K.QSNNKYAASSYLSLTPEQWK.S	W19(+14.98056122)
14.9827	0.2716954	Q9BPW8	256	1	R.GWDENVYYTVPLVR.H	W2(+14.982260413)
14.9827	0.2716954	Q13509	101	1	R.SGAFGHLFRPDNFIFGQSGAGNNWAK.G	W24(+14.980423647)
14.9827	0.2716954	Q14697	687	1	R.REPWLLPSQHNDIIR.D	W4(+14.981048369)
14.9827	0.2716954	Q9GZM7	407	1	K.ITGWGEETLPDGR.T	W4(+14.982719196)

14.9827	0.2716954	P00387	246	1	R.APEAWDYGQGFVNEEMIR.D	W5(+14.979397983)
14.9827	0.2716954	P40925	184	1	K.NVIIWGNHSSTQYPDVNHAK.V	W5(+14.979668778)
14.9827	0.2716954	P09429	133	1	K.LGEMWNNTAADDKQPYEK.K	W5(+14.981595818)
14.9827	0.2716954	P49458	7	1	M.PQYQTWEEFSR.A	W6(+14.979515001)
14.9827	0.2716954	P07237	407	1	K.QLAPIWDKLGETYKDHENIVIAK.M	W6(+14.982365492)
14.9827	0.2716954	Q8NBS9	227	1	K.ALAPTWEQLALGLEHSETVK.I	W6(+14.984941314)
14.9827	0.2716954	P00352	29	1	K.IFINNEWHDSVSGK.K	W7(+14.981471398)
14.9827	0.2716954	P07099	150	1	K.PLLMVHGWPGSFYEFYK.I	W8(+14.983830195)
14.9827	0.2716954	P02675	454	1	R.YYWGGQYTWDMAK.H	W9(+14.982050435)
