# Supplementary material for: Open Search-Based Proteomics Reveals Widespread Tryptophan Modifications Associated with Hypoxia in Lung Cancer
Source: Oxid Med Cell Longev. 2022 Apr 30;2022:2590198. doi: 10.1155/2022/2590198 (PMC9078843; doi:10.1155/2022/2590198)
Supplement: Supplementary Materials — Supplementary Figure 1: peptide spectrum matches (PSMs) of the identified 25 Trp variants. Supplementary Figure 2: proposed pathways of chemical reaction with in vivo metabolites (A) and tryptophan substitutions (B). The red-colored structures indicate the potential new modifications at tryptophan residue; the structures in grey color show the intermediates of the tryptophan modification pathway; the structures in black color show the preidentified tryptophan modifications. Supplementary Figure 3: proteins with tryptophan variants were largely clustered in blood microparticle, related to Figure 4. (a) Relative frequencies of each tryptophan modification group in the dataset. (b) Relative frequencies of delta mass clusters in the dataset. (c) Relative frequencies of each protein in the cellular components of blood microparticle, ficolin-1-rich granule lumen, and ficolin-1-rich granule. D. Heatmap depicting the correlation of tryptophan modifications in P69892 (HBG2). E. Color bar represents the relative frequency of differentially expressed oxidation modification at the 16 W, 38 W, and 131 W sites of P69892 (HBG2); the graph shows the overall structure of heme-core in P69892 (PDB: 4MQK). The linear distances of the 16 W, 38 W, and 131 W sites from the heme group are shown. Supplementary Figure 4: tryptophan variants associated with antioxidants prone to oxidative stress in NSCLC. A. Gene Ontology enrichment analysis of the modified-tryptophan-containing proteins in NSCLC, related to Figure 5(a); B. Relative expression levels of glycolytic enzymes in tumor samples and adjacent normal tissues in an independent cohort of 103 LUAD proteomic dataset (Xu et al., 2020, Cell 182, 245–261), related to Figure 6. Supplementary Figure 5: molecular docking of GAPDH (PubChem CID: 6 M61) with NAD+ and HBB (PubChem CID: 1CBL) with 2,3-diphosphoglycerate before and after oxidation, respectively. A. The 3D binding mode of NAD+ with GAPDH-wt; B. The 3D binding mode of NAD+ with GAPDH-w [file 2590198.f1.zip › Supplemental Table S2_20220310.pdf]

Supplementary Table 2: The pre-identified Trp modifications was constructed based on the Unimod database (http://www.unimod.org/) and references [Bachi A, et al., Chem Rev 2013, 113:596-698.],related to Figure 1B.

Note: Mono. mass: Monoisotopic mass.

Mono.mass	Description	Classifications	Composition	Positions
-186.07931	N_CID_Trp	CID	C(11)H(10)N(2O)	N-term
-129.05785	Trp->Gly substitution	AA substitution,mutation	H(-7)C(-9)N(-1)	Anywhere
-115.04220	Trp->Ala substitution	AA substitution	H(-5)C(-8)N(-1)	Anywhere
-99.04729	Trp->Ser substitution	AA substitution,mutation	H(-5)C(-8)N(-1)O	Anywhere
-89.02655	Trp->Pro substitution	AA substitution	H(-3)C(-6)N(-1)	Anywhere
-87.01090	Trp->Val substitution	AA substitution	H(-1)C(-6)N(-1)	Anywhere
-85.03163	Trp->Thr substitution	AA substitution	H(-3)C(-7)N(-1)O	Anywhere
-83.07013	Trp->Cys substitution	AA substitution,mutation	H(-5)C(-8)N(-1)S	Anywhere
-72.99525	Trp->Leu/Ile substitution	AA substitution,mutation	HC(-5)N(-1)	Anywhere
-72.03639	Trp->Asn substitution	AA substitution	H(-4)C(-7)O	Anywhere
-71.05237	Trp->Asp substitution	AA substitution	H(-5)C(-7)N(-1)O(2)	Anywhere
-58.02074	Trp->Gln substitution	AA substitution	H(-2)C(-6)O	Anywhere
-57.98435	Trp->Lys substitution	AA substitution	H(2)C(-5)	Anywhere
-57.03672	Trp->Glu substitution	AA substitution	H(-3)C(-6)N(-1)O(2)	Anywhere
-55.03883	Trp->Met substitution	AA substitution	H(-1)C(-6)N(-1)S	Anywhere
-49.02040	Trp->His substitution	AA substitution	H(-3)C(-5)N	Anywhere
-39.01090	Trp->Phe substitution	AA substitution	H(-1)C(-2)N(-1)	Anywhere
-29.97820	Trp->Arg substitution	AA substitution,mutation	H(2)C(-5)N(2)	Anywhere
-23.01598	Trp->Tyr substitution	AA substitution	H(-1)C(-2)N(-1)O	Anywhere
3.99492	Tryptophan oxidation to kynurenin	ROS/RNS, oxidation	C(-1)O	Anywhere
9.98435	Dihydro-b-carboline	ROS/RNS	H(-2)C	Anywhere
12.00000	Formaldehyde adduct	Chemical derivative	C	Anywhere
13.97927	Tryptophan oxidation to oxolactone	ROS/RNS, oxidation	H(-2)O	Anywhere
15.99492	Oxidation or Hydroxylation	ROS/RNS, oxidation	O	Anywhere
17.99058	Fluorination	Non-standard residue	H(-1)F	Anywhere
19.98983	Tryptophan oxidation to hydroxykynurenin	ROS/RNS, oxidation	C(-1)O(2)	Anywhere
27.95853	Tryptophan oxidation to b-unsaturated-2,4-bis-tryptophandione	ROS/RNS, oxidation	H(-4)O(2)	Anywhere
29.97418	Quinone	ROS/RNS, oxidation	H(-2)O(2)	Anywhere
30.01057	Formaldehyde induced modifications	Chemical derivative,ROS/RNS	H(2)CO	Anywhere
31.97207	Persulfide	Post-translational	S	Anywhere
31.98983	Dihydroxy	ROS/RNS, oxidation	O(2)	Anywhere
33.96103	Chlorination of tyrosine residues	ROS/RNS	H(-1)Cl	Anywhere
41.02655	Amidination of lysines or N-terminal amines with methyl acetimidate	Chemical derivative	H(3)C(2)N	Anywhere
43.95344	Tryptophan oxidation to hydroxy-bis-tryptophandione	Chemical derivative,ROS/RNS	H(-4)O(3)	Anywhere
43.98983	Carboxylation	Post-translational	CO(2)	Anywhere
44.98508	Oxidation to nitro	ROS/RNS, oxidation	H(-1)NO(2)	Anywhere
45.98772	β-Methylthiolation	Multiple	H(2)CS	Anywhere
47.98474	Trihydroxy	ROS/RNS, oxidation	O(3)	Anywhere
54.01057	Methylglyoxal-derived hydroimidazolone	Chemical derivative,ROS/RNS	H(2)C(3)O	Anywhere
55.98982	Glyoxylate	HMR,ROS/RNS	C(2)O(2)	Anywhere
58.00548	Iodoacetic acid derivative	Artefact	H(2)C(2)O(2)	Anywhere
60.98000	Nitro-hydroxy-tryptophan	ROS/RNS	H(-1)NO(3)	Anywhere
63.97966	Tryptophan oxidation to dihydroxy-N-formaylkynurenine	ROS/RNS, oxidation	O(4)	Anywhere
68.02622	Crotonylation	Post-translational	H(4)C(4)O	Anywhere
70.04187	Crotonaldehyde	Other	H(6)C(4)O	Anywhere
70.04187	(Iso)Butyryl	Post-translational	H(6)C(4)O	Anywhere
72.02113	Lactoyl	CoA	H(4)C(3)O(2)	Anywhere
72.02113	Carboxyethyl	Post-translational	H(4)C(3)O(2)	Anywhere
77.91051	Bromination	Post-translational,ROS/RNS	H(-1)Br	Anywhere
79.96633	Phosphorylation	Post-translational	HO(3)P	Anywhere

87.03203	Glycidamide adduct	Chemical derivative	H(5)C(3)NO(2)	Anywhere
100.01604	Methylmalonylation on Serine	Chemical derivative	H(4)C(4)O(3)	Anywhere
100.01604	Succiniyl	Chemical derivative	H(4)C(4)O(3)	Anywhere
106.00885	Reaction with methyl vinyl sulfone	Chemical derivative	H(6)C(3)O(2)S	Anywhere
119.00410	Cysteinylation	Multiple	H(5)C(3)NO(2)S	Anywhere
132.05752	Propiophenone	Chemical derivative	H(8)C(9)O	Anywhere
161.06881	Hexosamine	Other glycosylation	HexN	Anywhere
162.05282	Hexose	Other glycosylation	C(6)H(10)O(5)	Anywhere
180.08992	3-Hydroxy-L-kynurenine	AA incoperation	C(9)H(12)N(2)O(2)	Anywhere
183.03540	Aminoethylbenzenesulfonylation	Artefact	H(9)C(8)NO(2)S	Anywhere
185.11643	Add Lys-Gly	AA insertion	C(8)H(15)N(3)O(2)	Anywhere
198.98135	2,4-Dinitrobenzenesulfenyl	Chemical derivative	H(3)C(6)N(2)O(4)S	Anywhere
