# Supplementary material for: Open Search-Based Proteomics Reveals Widespread Tryptophan Modifications Associated with Hypoxia in Lung Cancer
Source: Oxid Med Cell Longev. 2022 Apr 30;2022:2590198. doi: 10.1155/2022/2590198 (PMC9078843; doi:10.1155/2022/2590198)
Supplement: Supplementary Materials — Supplementary Figure 1: peptide spectrum matches (PSMs) of the identified 25 Trp variants. Supplementary Figure 2: proposed pathways of chemical reaction with in vivo metabolites (A) and tryptophan substitutions (B). The red-colored structures indicate the potential new modifications at tryptophan residue; the structures in grey color show the intermediates of the tryptophan modification pathway; the structures in black color show the preidentified tryptophan modifications. Supplementary Figure 3: proteins with tryptophan variants were largely clustered in blood microparticle, related to Figure 4. (a) Relative frequencies of each tryptophan modification group in the dataset. (b) Relative frequencies of delta mass clusters in the dataset. (c) Relative frequencies of each protein in the cellular components of blood microparticle, ficolin-1-rich granule lumen, and ficolin-1-rich granule. D. Heatmap depicting the correlation of tryptophan modifications in P69892 (HBG2). E. Color bar represents the relative frequency of differentially expressed oxidation modification at the 16 W, 38 W, and 131 W sites of P69892 (HBG2); the graph shows the overall structure of heme-core in P69892 (PDB: 4MQK). The linear distances of the 16 W, 38 W, and 131 W sites from the heme group are shown. Supplementary Figure 4: tryptophan variants associated with antioxidants prone to oxidative stress in NSCLC. A. Gene Ontology enrichment analysis of the modified-tryptophan-containing proteins in NSCLC, related to Figure 5(a); B. Relative expression levels of glycolytic enzymes in tumor samples and adjacent normal tissues in an independent cohort of 103 LUAD proteomic dataset (Xu et al., 2020, Cell 182, 245–261), related to Figure 6. Supplementary Figure 5: molecular docking of GAPDH (PubChem CID: 6 M61) with NAD+ and HBB (PubChem CID: 1CBL) with 2,3-diphosphoglycerate before and after oxidation, respectively. A. The 3D binding mode of NAD+ with GAPDH-wt; B. The 3D binding mode of NAD+ with GAPDH-w [file 2590198.f1.zip › Supplemental Table S3_20220215.pdf]

Supplementary Table 3: The dataset of functionally grouped KEGG pathways of the modified-tryptophan-containing proteins, related to Figure 3.

GO ID	GO Term	Term P value	Group P value	GO Groups	% Associated Genes	Nr. Genes
KEGG:00020	Citrate cycle (TCA cycle)	0	0	Group00	50	15
KEGG:00071	Fatty acid degradation	0.03	0	Group01	25.58	11
KEGG:00330	Arginine and proline metabolism	0	0	Group02	27.45	14
KEGG:00480	Glutathione metabolism	0.01	0	Group03	24.56	14
KEGG:04141	Protein processing in endoplasmic reticulum	0.01	0	Group04	16.37	28
KEGG:04144	Endocytosis	0	0	Group05	15.54	39
KEGG:05110	Vibrio cholerae infection	0.02	0	Group06	24	12
KEGG:05132	Salmonella infection	0	0	Group07	16.47	41
KEGG:04145	Phagosome	0	0	Group08	22.37	34
KEGG:04612	Antigen processing and presentation	0	0	Group08	24.36	19
KEGG:05416	Viral myocarditis	0	0	Group08	26.67	16
KEGG:04510	Focal adhesion	0	0	Group09	18.91	38
KEGG:04512	ECM-receptor interaction	0	0	Group09	25	22
KEGG:05146	Amoebiasis	0	0	Group09	19.61	20
KEGG:04530	Tight junction	0.01	0	Group10	15.98	27
KEGG:04810	Regulation of actin cytoskeleton	0.04	0	Group10	14.22	31
KEGG:05100	Bacterial invasion of epithelial cells	0	0	Group10	31.17	24
KEGG:00010	Glycolysis / Gluconeogenesis	0	0	Group11	37.31	25
KEGG:00030	Pentose phosphate pathway	0	0	Group11	33.33	10
KEGG:00051	Fructose and mannose metabolism	0.01	0	Group11	30.3	10
KEGG:00620	Pyruvate metabolism	0.01	0	Group11	28.21	11
KEGG:04066	HIF-1 signaling pathway	0	0	Group11	21.1	23
KEGG:00190	Oxidative phosphorylation	0	0	Group12	21.05	28
KEGG:04260	Cardiac muscle contraction	0.01	0	Group12	20.69	18
KEGG:05410	Hypertrophic cardiomyopathy	0.01	0	Group12	20	18
KEGG:05412	Arrhythmogenic right ventricular cardiomyopathy	0	0	Group12	25.97	20
KEGG:05414	Dilated cardiomyopathy	0.01	0	Group12	19.79	19
KEGG:04740	Olfactory transduction	0	1	Group13	1.81	8
KEGG:04918	Thyroid hormone synthesis	0.03	1	Group13	20	15
KEGG:04961	Endocrine and other factor-regulated calcium reabsorption	0	1	Group13	26.42	14
KEGG:04971	Gastric acid secretion	0.04	1	Group13	19.74	15
KEGG:04972	Pancreatic secretion	0.04	1	Group13	17.65	18
KEGG:00190	Oxidative phosphorylation	0	0	Group14	21.05	28
KEGG:04260	Cardiac muscle contraction	0.01	0	Group14	20.69	18
KEGG:04714	Thermogenesis	0	0	Group14	16.02	37
KEGG:05010	Alzheimer disease	0	0	Group14	14.09	52
KEGG:05012	Parkinson disease	0	0	Group14	21.29	53
KEGG:05014	Amyotrophic lateral sclerosis	0	0	Group14	15.38	56
KEGG:05016	Huntington disease	0	0	Group14	18.63	57
KEGG:05020	Prion disease	0	0	Group14	19.78	54
KEGG:05022	Pathways of neurodegeneration	0	0	Group14	14.53	69

Note 1: Ontology Source: KEGG_14.02.2021.
Note 2: Term and group P values were corrected with Bonferroni step down.
