# Supplementary material for: Open Search-Based Proteomics Reveals Widespread Tryptophan Modifications Associated with Hypoxia in Lung Cancer
Source: Oxid Med Cell Longev. 2022 Apr 30;2022:2590198. doi: 10.1155/2022/2590198 (PMC9078843; doi:10.1155/2022/2590198)
Supplement: Supplementary Materials — Supplementary Figure 1: peptide spectrum matches (PSMs) of the identified 25 Trp variants. Supplementary Figure 2: proposed pathways of chemical reaction with in vivo metabolites (A) and tryptophan substitutions (B). The red-colored structures indicate the potential new modifications at tryptophan residue; the structures in grey color show the intermediates of the tryptophan modification pathway; the structures in black color show the preidentified tryptophan modifications. Supplementary Figure 3: proteins with tryptophan variants were largely clustered in blood microparticle, related to Figure 4. (a) Relative frequencies of each tryptophan modification group in the dataset. (b) Relative frequencies of delta mass clusters in the dataset. (c) Relative frequencies of each protein in the cellular components of blood microparticle, ficolin-1-rich granule lumen, and ficolin-1-rich granule. D. Heatmap depicting the correlation of tryptophan modifications in P69892 (HBG2). E. Color bar represents the relative frequency of differentially expressed oxidation modification at the 16 W, 38 W, and 131 W sites of P69892 (HBG2); the graph shows the overall structure of heme-core in P69892 (PDB: 4MQK). The linear distances of the 16 W, 38 W, and 131 W sites from the heme group are shown. Supplementary Figure 4: tryptophan variants associated with antioxidants prone to oxidative stress in NSCLC. A. Gene Ontology enrichment analysis of the modified-tryptophan-containing proteins in NSCLC, related to Figure 5(a); B. Relative expression levels of glycolytic enzymes in tumor samples and adjacent normal tissues in an independent cohort of 103 LUAD proteomic dataset (Xu et al., 2020, Cell 182, 245–261), related to Figure 6. Supplementary Figure 5: molecular docking of GAPDH (PubChem CID: 6 M61) with NAD+ and HBB (PubChem CID: 1CBL) with 2,3-diphosphoglycerate before and after oxidation, respectively. A. The 3D binding mode of NAD+ with GAPDH-wt; B. The 3D binding mode of NAD+ with GAPDH-w [file 2590198.f1.zip › Supplemental Table S4 20220215.pdf]

Supplementary Table 4: The dataset of cellular components of the modified-tryptophan-containing proteins and the corresponding frequencies per site, related to Figure 4.

ID	Description	Gene Ratio	Bg Ratio	pvalue	p.adjust	qvalue	Count	Frequency per site
GO:0072562	blood microparticle	38/829	205/30524	6.37E-21	4.54E-18	2.78E-18	38	18.32
GO:1904813	ficolin-1-rich granule lumen	37/829	249/30524	4.43E-17	1.58E-14	9.64E-15	37	13.18
GO:0044449	contractile fiber part	49/829	444/30524	1.04E-16	2.09E-14	1.28E-14	49	2.98
GO:0101002	ficolin-1-rich granule	44/829	364/30524	1.36E-16	2.09E-14	1.28E-14	44	12.45
GO:0043292	contractile fiber	50/829	465/30524	1.47E-16	2.09E-14	1.28E-14	50	3.15
GO:0030016	myofibril	48/829	448/30524	6.92E-16	8.21E-14	5.03E-14	48	3.07
GO:0030017	sarcomere	43/829	410/30524	4.86E-14	4.94E-12	3.02E-12	43	3.10
GO:0042470	melanosome	30/829	214/30524	2.01E-13	1.57E-11	9.64E-12	30	3.62
GO:0048770	pigment granule	30/829	214/30524	2.01E-13	1.57E-11	9.64E-12	30	3.62
GO:0044448	cell cortex part	39/829	358/30524	2.21E-13	1.57E-11	9.64E-12	39	3.74
GO:0032432	actin filament bundle	26/829	164/30524	4.44E-13	2.87E-11	1.76E-11	26	3.56
GO:0042641	actomyosin	26/829	173/30524	1.59E-12	9.41E-11	5.76E-11	26	3.98
GO:0031674	I band	34/829	298/30524	2.10E-12	1.15E-10	7.03E-11	34	3.20
GO:0030018	Z disc	32/829	278/30524	7.38E-12	3.75E-10	2.30E-10	32	3.06
GO:0098800	inner mitochondrial membrane protein complex	28/829	217/30524	9.84E-12	4.67E-10	2.86E-10	28	2.44
GO:0030027	lamellipodium	38/829	397/30524	2.32E-11	9.90E-10	6.06E-10	38	4.02
GO:0030863	cortical cytoskeleton	28/829	225/30524	2.36E-11	9.90E-10	6.06E-10	28	3.90
GO:0001725	stress fiber	23/829	152/30524	2.65E-11	9.92E-10	6.07E-10	23	3.67
GO:0097517	contractile actin filament bundle	23/829	152/30524	2.65E-11	9.92E-10	6.07E-10	23	3.67
GO:0005884	actin filament	27/829	221/30524	8.21E-11	2.92E-09	1.79E-09	27	4.57
GO:0030864	cortical actin cytoskeleton	23/829	180/30524	8.41E-10	2.85E-08	1.74E-08	23	3.36
GO:0044445	cytosolic part	36/829	413/30524	1.03E-09	3.33E-08	2.04E-08	36	25.52
GO:0044455	mitochondrial membrane part	34/829	378/30524	1.34E-09	4.06E-08	2.48E-08	34	2.28
GO:0070469	respiratory chain	22/829	169/30524	1.37E-09	4.06E-08	2.48E-08	22	2.23
GO:0098803	respiratory chain complex	20/829	140/30524	1.47E-09	4.19E-08	2.56E-08	20	2.25
GO:0005775	vacuolar lumen	32/829	347/30524	2.21E-09	6.06E-08	3.71E-08	32	4.49
GO:0042383	sarcolemma	29/829	295/30524	2.94E-09	7.75E-08	4.74E-08	29	2.02
GO:0005746	mitochondrial respiratory chain	20/829	150/30524	5.01E-09	1.27E-07	7.79E-08	20	2.32
GO:0035578	azurophil granule lumen	21/829	177/30524	1.72E-08	4.23E-07	2.59E-07	21	4.72
GO:0071682	endocytic vesicle lumen	11/829	46/30524	3.11E-08	7.38E-07	4.52E-07	11	30.62
GO:0031093	platelet alpha granule lumen	17/829	124/30524	4.63E-08	1.06E-06	6.51E-07	17	3.26
GO:0031091	platelet alpha granule	20/829	176/30524	7.75E-08	1.72E-06	1.06E-06	20	3.16
GO:0030662	coated vesicle membrane	30/829	367/30524	1.05E-07	2.27E-06	1.39E-06	30	2.04
GO:0005913	cell-cell adherens junction	24/829	251/30524	1.13E-07	2.37E-06	1.45E-06	24	2.45
GO:0098798	mitochondrial protein complex	32/829	423/30524	2.34E-07	4.75E-06	2.91E-06	32	2.69
GO:0022626	cytosolic ribosome	19/829	172/30524	2.55E-07	5.04E-06	3.08E-06	19	2.66
GO:1990204	oxidoreductase complex	20/829	190/30524	2.73E-07	5.26E-06	3.22E-06	20	2.12
GO:0098858	actin-based cell projection	31/829	409/30524	3.45E-07	6.46E-06	3.96E-06	31	3.74
GO:0005766	primary lysosome	25/829	292/30524	5.09E-07	9.06E-06	5.55E-06	25	4.56
GO:0042582	azurophil granule	25/829	292/30524	5.09E-07	9.06E-06	5.55E-06	25	4.56
GO:0030666	endocytic vesicle membrane	27/829	335/30524	5.96E-07	1.04E-05	6.34E-06	27	2.55
GO:0030665	clathrin-coated vesicle membrane	22/829	240/30524	7.77E-07	1.32E-05	8.06E-06	22	2.22
GO:0014704	intercalated disc	13/829	94/30524	1.58E-06	2.62E-05	1.60E-05	13	2.70
GO:0030175	filopodium	20/829	216/30524	2.09E-06	3.38E-05	2.07E-05	20	3.27
GO:0045335	phagocytic vesicle	22/829	255/30524	2.14E-06	3.38E-05	2.07E-05	22	3.69
GO:0032982	myosin filament	8/829	35/30524	3.52E-06	5.44E-05	3.33E-05	8	3.10
GO:0005840	ribosome	26/829	351/30524	4.54E-06	6.88E-05	4.21E-05	26	2.68
GO:0002102	podosome	10/829	63/30524	7.19E-06	1.07E-04	6.53E-05	10	3.71
GO:0001726	ruffle	25/829	340/30524	7.83E-06	1.14E-04	6.97E-05	25	3.95

GO:0005902	microvillus	15/829	147/30524	1.21E-05	1.72E-04	1.06E-04	15	3.90
GO:0043034	costamere	8/829	42/30524	1.49E-05	2.08E-04	1.27E-04	8	1.97
GO:0005790	smooth endoplasmic reticulum	10/829	69/30524	1.65E-05	2.25E-04	1.38E-04	10	3.52
GO:0005811	lipid droplet	15/829	151/30524	1.67E-05	2.25E-04	1.38E-04	15	8.07
GO:1904724	tertiary granule lumen	12/829	100/30524	1.75E-05	2.31E-04	1.41E-04	12	22.75
GO:0045334	clathrin-coated endocytic vesicle	13/829	120/30524	2.41E-05	3.12E-04	1.91E-04	13	2.17
GO:0044291	cell-cell contact zone	14/829	138/30524	2.50E-05	3.18E-04	1.95E-04	14	2.52
GO:0030427	site of polarized growth	25/829	365/30524	2.60E-05	3.25E-04	1.99E-04	25	3.04
GO:0043209	myelin sheath	12/829	105/30524	2.88E-05	3.53E-04	2.16E-04	12	7.23
GO:0098685	Schaffer collateral - CA1 synapse	15/829	160/30524	3.32E-05	4.00E-04	2.45E-04	15	5.18
GO:0005916	fascia adherens	6/829	24/30524	3.49E-05	4.14E-04	2.53E-04	6	3.23
GO:0030136	clathrin-coated vesicle	25/829	372/30524	3.56E-05	4.15E-04	2.54E-04	25	2.27
GO:0005903	brush border	16/829	183/30524	4.32E-05	4.89E-04	2.99E-04	16	3.33
GO:0005798	Golgi-associated vesicle	23/829	331/30524	4.33E-05	4.89E-04	2.99E-04	23	2.24
GO:0016528	sarcoplasm	15/829	165/30524	4.75E-05	5.28E-04	3.23E-04	15	3.14
GO:0005577	fibrinogen complex	5/829	16/30524	4.97E-05	5.44E-04	3.33E-04	5	2.53
GO:0070069	cytochrome complex	8/829	50/30524	5.60E-05	6.04E-04	3.70E-04	8	2.58
GO:0031594	neuromuscular junction	15/829	168/30524	5.84E-05	6.21E-04	3.80E-04	15	1.81
GO:0120111	neuron projection cytoplasm	16/829	188/30524	5.98E-05	6.26E-04	3.83E-04	16	2.02
GO:0071013	catalytic step 2 spliceosome	14/829	150/30524	6.31E-05	6.51E-04	3.99E-04	14	1.22
GO:0005844	polysome	13/829	133/30524	7.12E-05	7.24E-04	4.43E-04	13	3.03
GO:0016460	myosin II complex	7/829	39/30524	7.66E-05	7.68E-04	4.70E-04	7	3.00
GO:0030658	transport vesicle membrane	25/829	391/30524	7.94E-05	7.85E-04	4.81E-04	25	2.05
GO:0005604	basement membrane	15/829	174/30524	8.71E-05	8.49E-04	5.20E-04	15	2.40
GO:0022627	cytosolic small ribosomal subunit	9/829	68/30524	9.05E-05	8.71E-04	5.33E-04	9	3.14
GO:0071556	integral component of lumenal side of endoplasmic reticulum membrane	9/829	69/30524	1.02E-04	9.52E-04	5.82E-04	9	2.42
GO:0098553	lumenal side of endoplasmic reticulum membrane	9/829	69/30524	1.02E-04	9.52E-04	5.82E-04	9	2.42
GO:0005747	mitochondrial respiratory chain complex I	10/829	86/30524	1.14E-04	1.03E-03	6.28E-04	10	1.92
GO:0030964	NADH dehydrogenase complex	10/829	86/30524	1.14E-04	1.03E-03	6.28E-04	10	1.92
GO:0045271	respiratory chain complex I	10/829	86/30524	1.14E-04	1.03E-03	6.28E-04	10	1.92
GO:0070820	tertiary granule	21/829	309/30524	1.25E-04	1.11E-03	6.81E-04	21	19.02
GO:0044391	ribosomal subunit	20/829	287/30524	1.28E-04	1.13E-03	6.91E-04	20	2.62
GO:0031941	filamentous actin	8/829	57/30524	1.46E-04	1.26E-03	7.68E-04	8	5.63
GO:0030426	growth cone	23/829	359/30524	1.47E-04	1.26E-03	7.68E-04	23	3.25
GO:0032154	cleavage furrow	11/829	106/30524	1.48E-04	1.26E-03	7.68E-04	11	2.28
GO:0005833	hemoglobin complex	5/829	20/30524	1.61E-04	1.32E-03	8.07E-04	5	67.09
GO:0031838	haptoglobin-hemoglobin complex	5/829	20/30524	1.61E-04	1.32E-03	8.07E-04	5	60.23
GO:0034663	endoplasmic reticulum chaperone complex	5/829	20/30524	1.61E-04	1.32E-03	8.07E-04	5	4.57
GO:0030669	clathrin-coated endocytic vesicle membrane	10/829	91/30524	1.83E-04	1.48E-03	9.06E-04	10	2.42
GO:0008091	spectrin	4/829	12/30524	2.25E-04	1.80E-03	1.10E-03	4	2.59
GO:0030120	vesicle coat	11/829	112/30524	2.41E-04	1.91E-03	1.17E-03	11	2.48
GO:0005793	endoplasmic reticulum-Golgi intermediate compartment	17/829	235/30524	2.59E-04	2.03E-03	1.24E-03	17	3.93
GO:0022625	cytosolic large ribosomal subunit	10/829	96/30524	2.84E-04	2.20E-03	1.35E-03	10	2.15
GO:0098862	cluster of actin-based cell projections	19/829	282/30524	2.87E-04	2.20E-03	1.35E-03	19	3.21
GO:0031430	M band	7/829	48/30524	2.97E-04	2.24E-03	1.37E-03	7	2.17
GO:0030660	Golgi-associated vesicle membrane	16/829	216/30524	2.99E-04	2.24E-03	1.37E-03	16	2.05
GO:1905368	peptidase complex	13/829	159/30524	4.22E-04	3.13E-03	1.92E-03	13	2.51
GO:0000502	proteasome complex	11/829	120/30524	4.39E-04	3.22E-03	1.97E-03	11	2.61
GO:1905369	endopeptidase complex	11/829	121/30524	4.72E-04	3.43E-03	2.10E-03	11	2.61
GO:0031143	pseudopodium	6/829	38/30524	5.18E-04	3.73E-03	2.28E-03	6	4.92
GO:0097512	cardiac myofibril	4/829	15/30524	5.80E-04	4.05E-03	2.48E-03	4	2.88
GO:0099571	postsynaptic cytoskeleton	4/829	15/30524	5.80E-04	4.05E-03	2.48E-03	4	5.08

GO:0032155	cell division site part	11/829	124/30524	5.81E-04	4.05E-03	2.48E-03	11	2.28
GO:0005682	U5 snRNP	5/829	26/30524	5.98E-04	4.13E-03	2.53E-03	5	1.44
GO:0030532	small nuclear ribonucleoprotein complex	10/829	106/30524	6.30E-04	4.31E-03	2.64E-03	10	1.30
GO:1904115	axon cytoplasm	11/829	126/30524	6.64E-04	4.50E-03	2.76E-03	11	1.94
GO:0005852	eukaryotic translation initiation factor 3 complex	5/829	27/30524	7.17E-04	4.79E-03	2.93E-03	5	2.20
GO:0005681	spliceosomal complex	20/829	328/30524	7.20E-04	4.79E-03	2.93E-03	20	1.40
GO:0071006	U2-type catalytic step 1 spliceosome	4/829	16/30524	7.57E-04	4.95E-03	3.03E-03	4	1.50
GO:0071012	catalytic step 1 spliceosome	4/829	16/30524	7.57E-04	4.95E-03	3.03E-03	4	1.50
GO:0048786	presynaptic active zone	12/829	149/30524	7.93E-04	5.13E-03	3.14E-03	12	1.97
GO:0071437	invadopodium	5/829	28/30524	8.54E-04	5.48E-03	3.35E-03	5	2.42
GO:0032994	protein-lipid complex	8/829	74/30524	8.88E-04	5.64E-03	3.45E-03	8	9.25
GO:0032153	cell division site	11/829	131/30524	9.19E-04	5.79E-03	3.54E-03	11	2.28
GO:0034358	plasma lipoprotein particle	7/829	58/30524	9.58E-04	5.93E-03	3.63E-03	7	9.80
GO:1990777	lipoprotein particle	7/829	58/30524	9.58E-04	5.93E-03	3.63E-03	7	9.80
GO:0005883	neurofilament	4/829	17/30524	9.69E-04	5.95E-03	3.64E-03	4	2.85
GO:0030479	actin cortical patch	5/829	29/30524	1.01E-03	6.09E-03	3.72E-03	5	1.67
GO:0061645	endocytic patch	5/829	29/30524	1.01E-03	6.09E-03	3.72E-03	5	1.67
GO:0034364	high-density lipoprotein particle	6/829	43/30524	1.02E-03	6.10E-03	3.73E-03	6	10.43
GO:0030125	clathrin vesicle coat	7/829	59/30524	1.06E-03	6.30E-03	3.86E-03	7	2.63
GO:0030496	midbody	19/829	317/30524	1.18E-03	6.95E-03	4.26E-03	19	3.33
GO:0005750	mitochondrial respiratory chain complex III	4/829	18/30524	1.22E-03	7.06E-03	4.32E-03	4	2.67
GO:0045275	respiratory chain complex III	4/829	18/30524	1.22E-03	7.06E-03	4.32E-03	4	2.67
GO:0097525	spliceosomal snRNP complex	9/829	97/30524	1.31E-03	7.48E-03	4.58E-03	9	1.33
GO:0032838	plasma membrane bounded cell projection cytoplasm	22/829	395/30524	1.31E-03	7.48E-03	4.58E-03	22	2.11
GO:0071005	U2-type precatalytic spliceosome	8/829	79/30524	1.37E-03	7.71E-03	4.72E-03	8	1.29
GO:0045277	respiratory chain complex IV	5/829	31/30524	1.38E-03	7.74E-03	4.74E-03	5	2.29
GO:0044420	extracellular matrix component	10/829	118/30524	1.44E-03	8.03E-03	4.91E-03	10	2.55
GO:0071011	precatalytic spliceosome	8/829	80/30524	1.48E-03	8.18E-03	5.00E-03	8	1.29
GO:0030117	membrane coat	13/829	183/30524	1.56E-03	8.47E-03	5.18E-03	13	2.37
GO:0048475	coated membrane	13/829	183/30524	1.56E-03	8.47E-03	5.18E-03	13	2.37
GO:0030132	clathrin coat of coated pit	6/829	47/30524	1.64E-03	8.71E-03	5.33E-03	6	2.72
GO:0046540	U4/U6 x U5 tri-snRNP complex	6/829	47/30524	1.64E-03	8.71E-03	5.33E-03	6	1.36
GO:0097526	spliceosomal tri-snRNP complex	6/829	47/30524	1.64E-03	8.71E-03	5.33E-03	6	1.36
GO:0005865	striated muscle thin filament	7/829	64/30524	1.72E-03	9.08E-03	5.56E-03	7	1.85
GO:0031258	lamellipodium membrane	5/829	33/30524	1.84E-03	9.47E-03	5.80E-03	5	9.60
GO:0034361	very-low-density lipoprotein particle	5/829	33/30524	1.84E-03	9.47E-03	5.80E-03	5	11.15
GO:0034385	triglyceride-rich plasma lipoprotein particle	5/829	33/30524	1.84E-03	9.47E-03	5.80E-03	5	11.15
GO:0005885	Arp2/3 protein complex	4/829	20/30524	1.85E-03	9.47E-03	5.80E-03	4	2.43
GO:0016529	sarcoplasmic reticulum	11/829	143/30524	1.87E-03	9.50E-03	5.82E-03	11	2.87
GO:0036379	myofilament	7/829	66/30524	2.06E-03	1.04E-02	6.37E-03	7	1.85
GO:0019814	immunoglobulin complex	3/829	10/30524	2.08E-03	1.04E-02	6.37E-03	3	4.38
GO:0055038	recycling endosome membrane	11/829	146/30524	2.20E-03	1.10E-02	6.71E-03	11	1.63
GO:0005751	mitochondrial respiratory chain complex IV	4/829	21/30524	2.24E-03	1.11E-02	6.77E-03	4	2.80
GO:0120114	Sm-like protein family complex	10/829	127/30524	2.49E-03	1.22E-02	7.49E-03	10	1.30
GO:0042788	polysomal ribosome	6/829	51/30524	2.51E-03	1.22E-02	7.49E-03	6	2.38
GO:0012507	ER to Golgi transport vesicle membrane	10/829	128/30524	2.64E-03	1.28E-02	7.83E-03	10	1.20
GO:0031672	A band	7/829	70/30524	2.89E-03	1.39E-02	8.51E-03	7	2.17
GO:0016459	myosin complex	9/829	110/30524	3.12E-03	1.47E-02	9.03E-03	9	3.05
GO:0042611	MHC protein complex	8/829	90/30524	3.13E-03	1.47E-02	9.03E-03	8	1.13
GO:0005905	clathrin-coated pit	11/829	153/30524	3.17E-03	1.47E-02	9.03E-03	11	2.53
GO:0030130	clathrin coat of trans-Golgi network vesicle	4/829	23/30524	3.17E-03	1.47E-02	9.03E-03	4	2.94
GO:0060198	clathrin-sculpted vesicle	4/829	23/30524	3.17E-03	1.47E-02	9.03E-03	4	2.22

GO:0031304	intrinsic component of mitochondrial inner membrane	7/829	72/30524	3.39E-03	1.56E-02	9.53E-03	7	1.25
GO:0031305	integral component of mitochondrial inner membrane	7/829	72/30524	3.39E-03	1.56E-02	9.53E-03	7	1.25
GO:0035749	myelin sheath adaxonal region	3/829	12/30524	3.66E-03	1.67E-02	1.02E-02	3	8.00
GO:0043256	laminin complex	4/829	24/30524	3.72E-03	1.68E-02	1.03E-02	4	2.00
GO:0015935	small ribosomal subunit	9/829	113/30524	3.73E-03	1.68E-02	1.03E-02	9	3.14
GO:0043197	dendritic spine	19/829	352/30524	3.81E-03	1.71E-02	1.04E-02	19	2.50
GO:0043202	lysosomal lumen	13/829	203/30524	3.84E-03	1.71E-02	1.05E-02	13	3.83
GO:0044309	neuron spine	19/829	355/30524	4.17E-03	1.85E-02	1.13E-02	19	2.50
GO:0030134	COPII-coated ER to Golgi transport vesicle	12/829	182/30524	4.26E-03	1.87E-02	1.15E-02	12	1.42
GO:0036020	endolysosome membrane	4/829	25/30524	4.34E-03	1.88E-02	1.15E-02	4	2.94
GO:0043218	compact myelin	4/829	25/30524	4.34E-03	1.88E-02	1.15E-02	4	10.23
GO:0030118	clathrin coat	8/829	95/30524	4.36E-03	1.88E-02	1.15E-02	8	2.55
GO:0031256	leading edge membrane	18/829	333/30524	4.71E-03	2.02E-02	1.24E-02	18	3.46
GO:0005859	muscle myosin complex	4/829	26/30524	5.02E-03	2.11E-02	1.29E-02	4	3.10
GO:0012510	trans-Golgi network transport vesicle membrane	4/829	26/30524	5.02E-03	2.11E-02	1.29E-02	4	2.94
GO:0033290	eukaryotic 48S preinitiation complex	4/829	26/30524	5.02E-03	2.11E-02	1.29E-02	4	2.25
GO:0030315	T-tubule	9/829	119/30524	5.24E-03	2.20E-02	1.34E-02	9	2.08
GO:0098563	intrinsic component of synaptic vesicle membrane	7/829	79/30524	5.67E-03	2.36E-02	1.45E-02	7	2.21
GO:0099738	cell cortex region	7/829	80/30524	6.07E-03	2.51E-02	1.54E-02	7	2.84
GO:0071203	WASH complex	4/829	28/30524	6.58E-03	2.71E-02	1.66E-02	4	1.25
GO:0005684	U2-type spliceosomal complex	10/829	146/30524	6.69E-03	2.74E-02	1.68E-02	10	1.26
GO:0090734	site of DNA damage	9/829	124/30524	6.83E-03	2.78E-02	1.70E-02	9	2.21
GO:0043198	dendritic shaft	7/829	82/30524	6.94E-03	2.81E-02	1.72E-02	7	5.35
GO:0030670	phagocytic vesicle membrane	10/829	147/30524	7.01E-03	2.81E-02	1.72E-02	10	2.41
GO:0005890	sodium:potassium-exchanging ATPase complex	3/829	15/30524	7.11E-03	2.81E-02	1.72E-02	3	2.08
GO:0014731	spectrin-associated cytoskeleton	3/829	15/30524	7.11E-03	2.81E-02	1.72E-02	3	2.36
GO:0072669	tRNA-splicing ligase complex	3/829	15/30524	7.11E-03	2.81E-02	1.72E-02	3	1.25
GO:0005952	cAMP-dependent protein kinase complex	4/829	29/30524	7.47E-03	2.92E-02	1.79E-02	4	2.00
GO:0016282	eukaryotic 43S preinitiation complex	4/829	29/30524	7.47E-03	2.92E-02	1.79E-02	4	2.25
GO:0030867	rough endoplasmic reticulum membrane	5/829	46/30524	7.97E-03	3.10E-02	1.90E-02	5	2.40
GO:0005777	peroxisome	13/829	225/30524	8.91E-03	3.41E-02	2.09E-02	13	1.81
GO:0042579	microbody	13/829	225/30524	8.91E-03	3.41E-02	2.09E-02	13	1.81
GO:0044853	plasma membrane raft	13/829	225/30524	8.91E-03	3.41E-02	2.09E-02	13	2.09
GO:0070993	translation preinitiation complex	4/829	31/30524	9.49E-03	3.61E-02	2.21E-02	4	2.25
GO:0101031	chaperone complex	5/829	48/30524	9.53E-03	3.61E-02	2.21E-02	5	2.23
GO:0030176	integral component of endoplasmic reticulum membrane	16/829	305/30524	9.74E-03	3.67E-02	2.25E-02	16	3.16
GO:0061617	MICOS complex	3/829	17/30524	1.02E-02	3.83E-02	2.34E-02	3	1.50
GO:0030137	COPI-coated vesicle	5/829	49/30524	1.04E-02	3.87E-02	2.37E-02	5	2.50
GO:0015934	large ribosomal subunit	11/829	180/30524	1.04E-02	3.88E-02	2.37E-02	11	2.10
GO:0019897	extrinsic component of plasma membrane	17/829	336/30524	1.10E-02	4.06E-02	2.49E-02	17	2.06
GO:0005802	trans-Golgi network	22/829	476/30524	1.16E-02	4.23E-02	2.59E-02	22	2.22
GO:0032587	ruffle membrane	11/829	183/30524	1.17E-02	4.23E-02	2.59E-02	11	4.66
GO:0005915	zonula adherens	3/829	18/30524	1.20E-02	4.23E-02	2.59E-02	3	1.00
GO:0019773	proteasome core complex, alpha-subunit complex	3/829	18/30524	1.20E-02	4.23E-02	2.59E-02	3	1.25
GO:0043220	Schmidt-Lanterman incisure	3/829	18/30524	1.20E-02	4.23E-02	2.59E-02	3	2.38
GO:0044233	Mitochondria-associated ER Membrane	3/829	18/30524	1.20E-02	4.23E-02	2.59E-02	3	2.24
GO:0061200	clathrin-sculpted gamma-aminobutyric acid transport vesicle	3/829	18/30524	1.20E-02	4.23E-02	2.59E-02	3	2.38
GO:0061202	clathrin-sculpted gamma-aminobutyric acid transport vesicle membrane	3/829	18/30524	1.20E-02	4.23E-02	2.59E-02	3	2.38

GO:0097441	basal dendrite	3/829	18/30524	1.20E-02	4.23E-02	2.59E-02	3	1.43
GO:0035580	specific granule lumen	8/829	113/30524	1.21E-02	4.23E-02	2.59E-02	8	3.01
GO:0016328	lateral plasma membrane	8/829	114/30524	1.27E-02	4.42E-02	2.71E-02	8	1.31
GO:0030128	clathrin coat of endocytic vesicle	4/829	34/30524	1.31E-02	4.56E-02	2.79E-02	4	1.00
GO:0030140	trans-Golgi network transport vesicle	5/829	52/30524	1.32E-02	4.58E-02	2.80E-02	5	3.20
GO:0031901	early endosome membrane	14/829	263/30524	1.33E-02	4.58E-02	2.81E-02	14	2.07
GO:0032839	dendrite cytoplasm	6/829	72/30524	1.35E-02	4.62E-02	2.83E-02	6	2.08
GO:0008250	oligosaccharyltransferase complex	3/829	19/30524	1.40E-02	4.71E-02	2.88E-02	3	1.00
GO:0008290	F-actin capping protein complex	3/829	19/30524	1.40E-02	4.71E-02	2.88E-02	3	1.50
GO:0033018	sarcoplasmic reticulum lumen	3/829	19/30524	1.40E-02	4.71E-02	2.88E-02	3	3.32
GO:0031227	intrinsic component of endoplasmic reticulum membrane	16/829	318/30524	1.40E-02	4.71E-02	2.88E-02	16	3.16
GO:0035861	site of double-strand break	7/829	94/30524	1.42E-02	4.74E-02	2.90E-02	7	2.45
GO:0099092	postsynaptic density, intracellular component	4/829	35/30524	1.45E-02	4.83E-02	2.95E-02	4	3.00
