# Supplementary material for: Open Search-Based Proteomics Reveals Widespread Tryptophan Modifications Associated with Hypoxia in Lung Cancer
Source: Oxid Med Cell Longev. 2022 Apr 30;2022:2590198. doi: 10.1155/2022/2590198 (PMC9078843; doi:10.1155/2022/2590198)

Supplementary Materials list

Open Search-based Proteomics Reveal Widespread Tryptophan Modifications Associated with Hypoxia in Lung Cancer

Jinfeng Chen, [1] Lei Zhang, [1] Zhao Sun, [2] Hongyi Li, [2] Jingyi Li, [1] Xinli Xue, [1] Qingqing Zhu, [1] Bowen Dong, [1] Yuanyuan Wang, [1] Yang Yang, [1] Yongqiang Dong, [2] Guangyu Guo, [1] Hongqiang Jiang, [2] An Zhang, [1] Guoqing Zhang, [2] Zhichao Hou, [2] Xiangnan Li, [2] and Jing-Hua Yang [1,*]

[1] Clinical Systems Biology Key Laboratories of Henan, Translational Medicine Center, the First Affiliated Hospital of Zhengzhou University, Zhengzhou, 450052, Henan, China.

[2] Departments of Surgery, the First Affiliated Hospital of Zhengzhou University, Zhengzhou, Henan, 450052, China.

***** Correspondence: Jing-Hua Yang; E-mail address: jyang@bu.edu

CONTENTS

Supplementary Figures

Supplementary Figure 1: Peptide spectrum matches (PSMs) of the identified 25 Trp variants.

Supplementary Figure 2: Proposed pathways of chemical reaction with *in vivo* metabolites (A) and tryptophan substitutions (B). The red-colored structures indicate the potential new modifications at tryptophan residue; the structures in grey color show the intermediates of the tryptophan modification pathway; the structures in black color show the pre-identified tryptophan modifications.

Supplementary Figure 3: Proteins with tryptophan variants were largely clustered in blood microparticle, related to Figure 4. A: Relative frequencies of each tryptophan modification group in the dataset. B: Relative frequencies of delta mass clusters in the dataset. C: Relative frequencies of each protein in the cellular components of blood microparticle, ficolin-1-rich granule lumen, and ficolin-1-rich granule. D. Heatmap depicting the correlation of tryptophan modifications in P69892 (HBG2). E. Color bar represents the relative frequency of differentially expressed oxidation modification at the 16W, 38W and 131W sites of P69892 (HBG2); the graph shows the overall structure of heme-core in P69892 (PDB: 4MQK). The linear distances of the 16W, 38W and 131W sites from the heme group are shown.

Supplementary Figure 4: Tryptophan variants associated with antioxidants prone to oxidative stress in NSCLC. A. Gene ontology enrichment analysis of the modified-tryptophan-containing proteins in NSCLC, related to Figure 5A; B. Relative expression levels of glycolytic enzymes in tumor samples and adjacent normal tissues in an independent cohort of 103 LUAD proteomic dataset (Xu et al., 2020, Cell 182, 245–261), related to Figure 6.

Supplementary Figure 5: Molecular docking of GAPDH (PubChem CID: 6M61) with NAD^+ and HBB (PubChem CID: 1CBL) with 2,3-diphosphoglycerate before and after oxidation, respectively. A. The 3D binding mode of NAD^+ with GAPDH-wt; B. The 3D binding mode of NAD^+ with GAPDH-wt. NAD^+ is colored in yellow. The surrounding residues in the binding pocket are colored in light blue. The backbone of the receptor is depicted as light blue cartoon. C. The 3D binding mode of 2,3-diphosphoglycerate with HBB-wt. D. The 3D binding mode of 2,3-diphosphoglycerate with HBB-W16/W38. The 2,3-diphosphoglycerate is colored in yellow.

The surrounding residues in the binding pocket are colored in light blue. The backbone and residue of HBB-wt alpha chain is colored in light blue, the backbone and residue of HBB-wt beta chain is colored in white. The hydrogen bonds are depicted as yellow dashed lines. The salt bridges are depicted as magenta dashed lines.

Supplementary Tables

Supplementary Table 1: The dataset of tryptophan modification.

Supplementary Table 2: The pre-identified Trp modifications was constructed based on the Unimod database(http://www.unimod.org/) and references [Bachi A, et al., Chem Rev 2013, 113:596-698.], related to Figure 1B.

Supplementary Table 3: The dataset of functionally grouped KEGG pathways of the modified-tryptophan-containing proteins.

Supplementary Table 4: The dataset of cellular components of the modified-tryptophan-containing proteins and the corresponding frequencies per site, related to Figure 4.

Supplementary Figure 1: Peptide spectrum matches (PSMs) of the identified 25 Trp variants. (1) – (4)

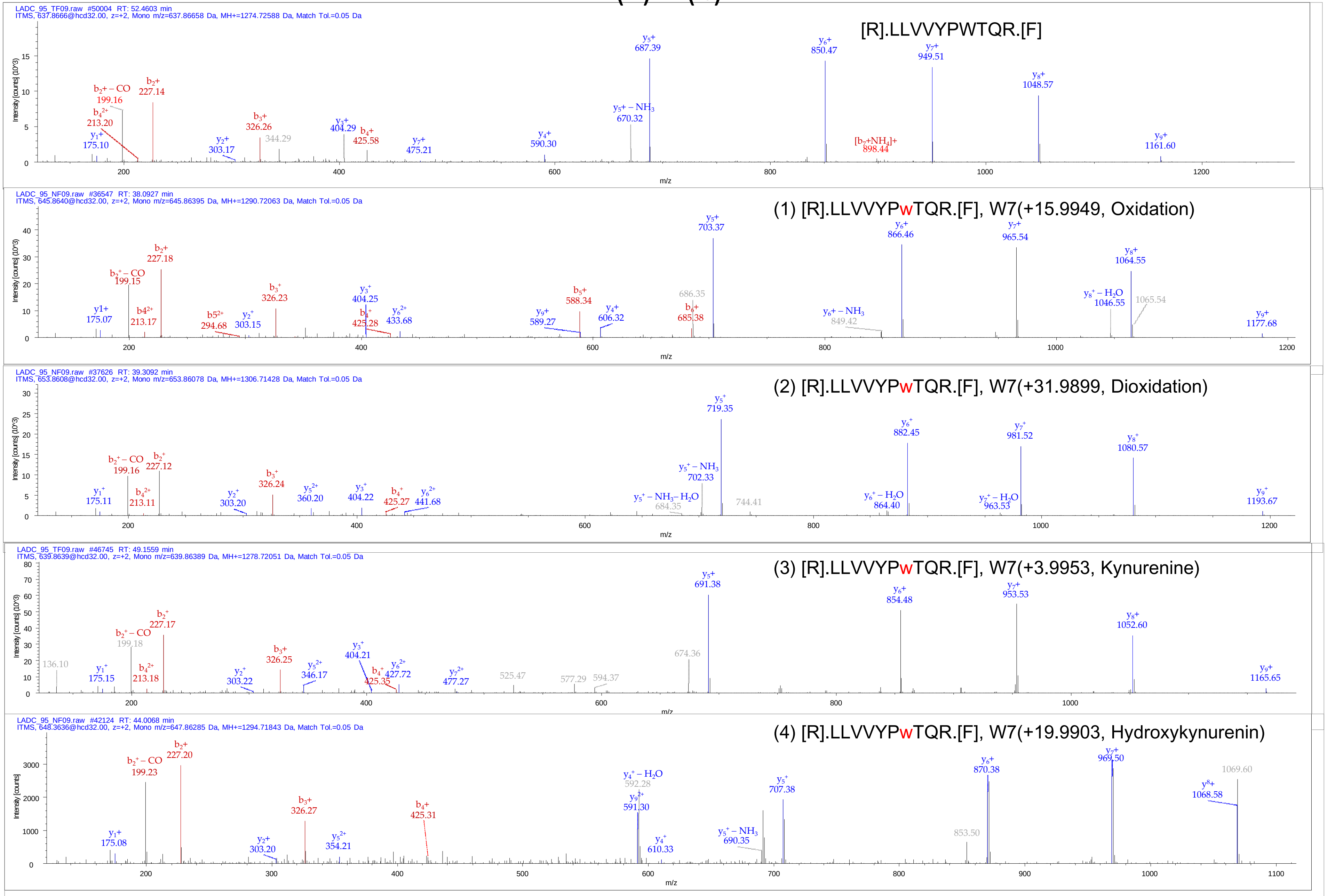

(5)

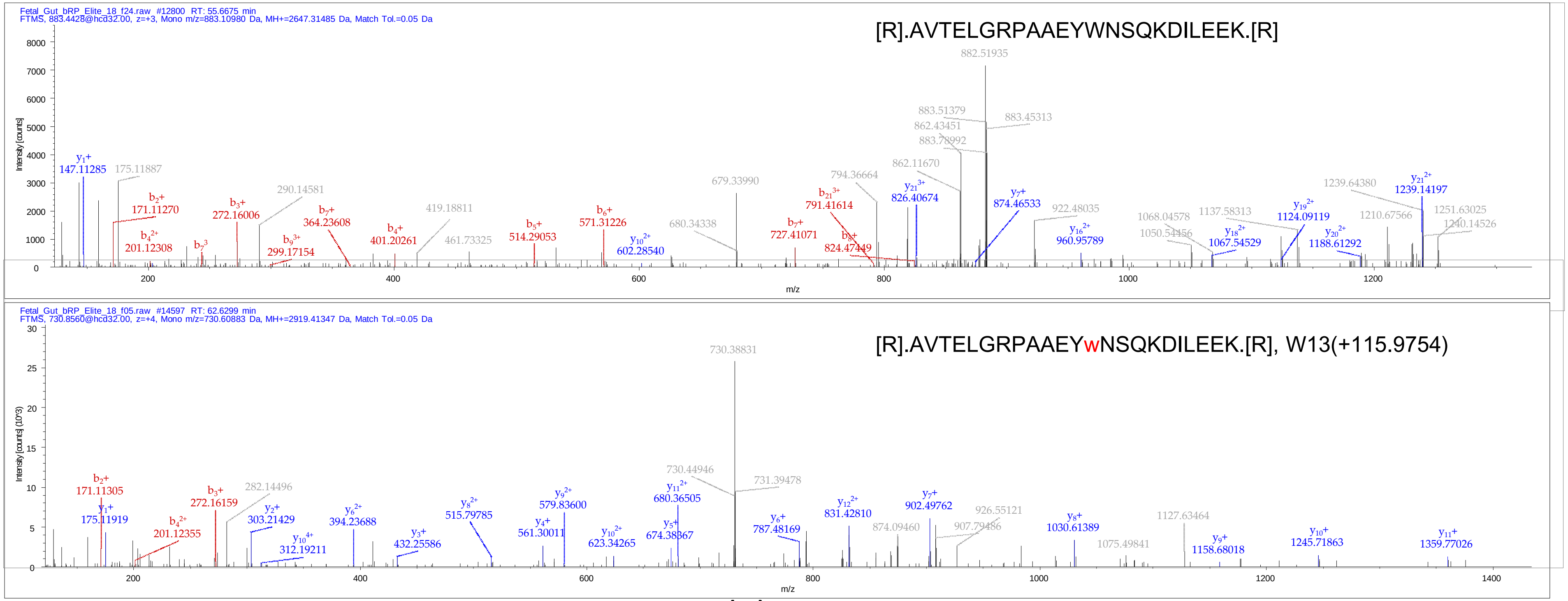
Fetal_Gut_bRP_Elite_18_f24.raw #12800 RT: 55.6675 min
FTMS, 883.4428@hcd32.00, z=+3, Mono m/z=883.10980 Da, MH+=2647.31485 Da, Match Tol.=0.05 Da
[R].AVTELGRPAAEYWNSQKDILEEK.[R]
Intensity [counts]
m/z
Fetal_Gut_bRP_Elite_18_f05.raw #14597 RT: 62.6299 min
FTMS, 730.8560@hcd32.00, z=+4, Mono m/z=730.60883 Da, MH+=2919.41347 Da, Match Tol.=0.05 Da
[R].AVTELGRPAAEYwNSQKDILEEK.[R], W13(+115.9754)
Intensity [counts] (10^3)
m/z

(6)

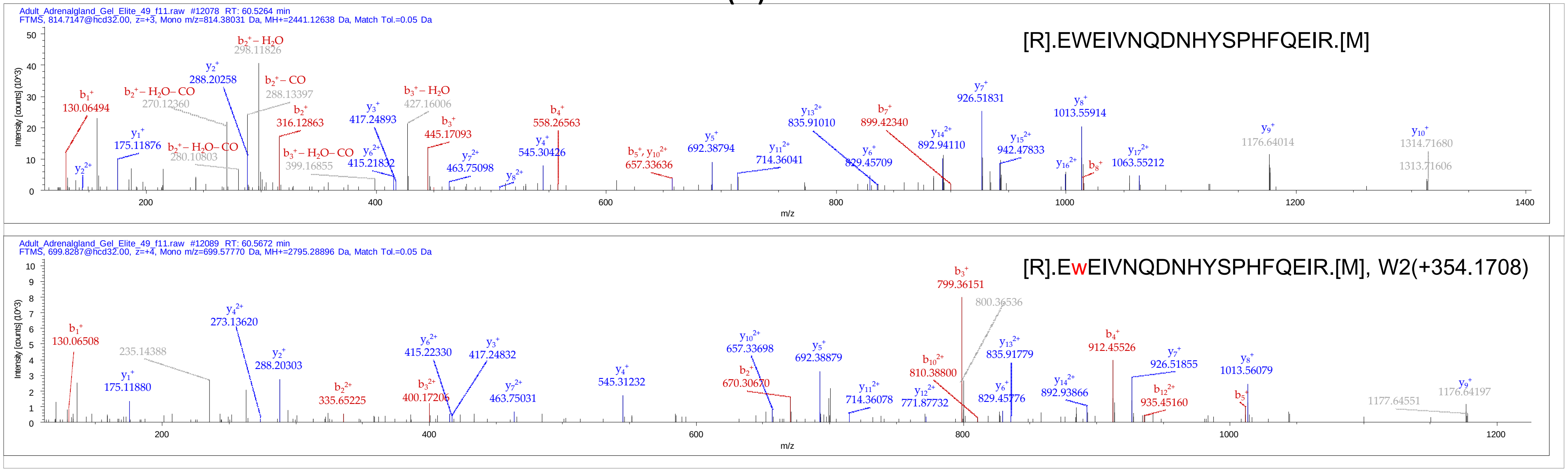
Adult_Adrenalgland_Gel_Elite_49_f11.raw #12078 RT: 60.5264 min
FTMS, 814.7147@hcd32.00, z=+3, Mono m/z=814.38031 Da, MH+=2441.12638 Da, Match Tol.=0.05 Da
[R].EWEIVNQDNHYSPHFQEIR.[M]
Intensity [counts] (10^3)
m/z
Adult_Adrenalgland_Gel_Elite_49_f11.raw #12089 RT: 60.5672 min
FTMS, 699.8287@hcd32.00, z=+4, Mono m/z=699.57770 Da, MH+=2795.28896 Da, Match Tol.=0.05 Da
[R].EwEIVNQDNHYSPHFQEIR.[M], W2(+354.1708)
Intensity [counts] (10^3)
m/z

(7)

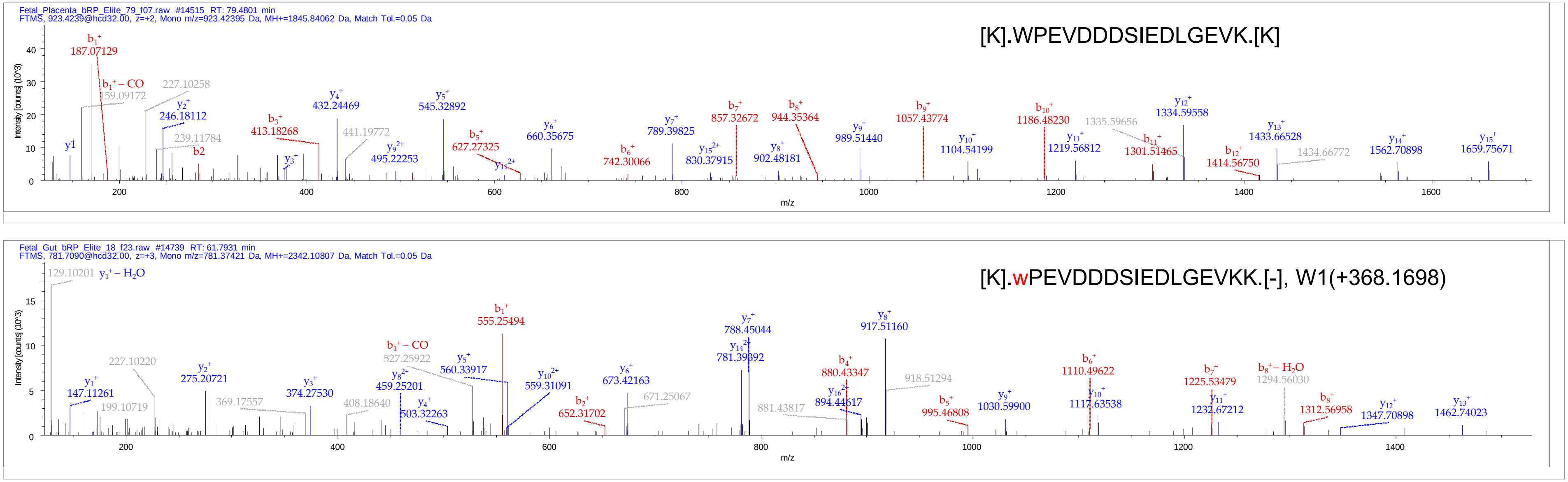
Fetal_Placenta_bRP_Elite_79_f07.raw #14515 RT: 79.4801 min
FTMS, 923.4239@hcd32.00, z=+2, Mono m/z=923.42395 Da, MH+=1845.84062 Da, Match Tol.=0.05 Da
[K].WPEVDDDSIEDLGEVK.[K]
Fetal_Gut_bRP_Elite_18_f23.raw #14739 RT: 61.7931 min
FTMS, 781.7090@hcd32.00, z=+3, Mono m/z=781.37421 Da, MH+=2342.10807 Da, Match Tol.=0.05 Da
[K].wPEVDDDSIEDLGEVKK.[-], W1(+368.1698)

(8)

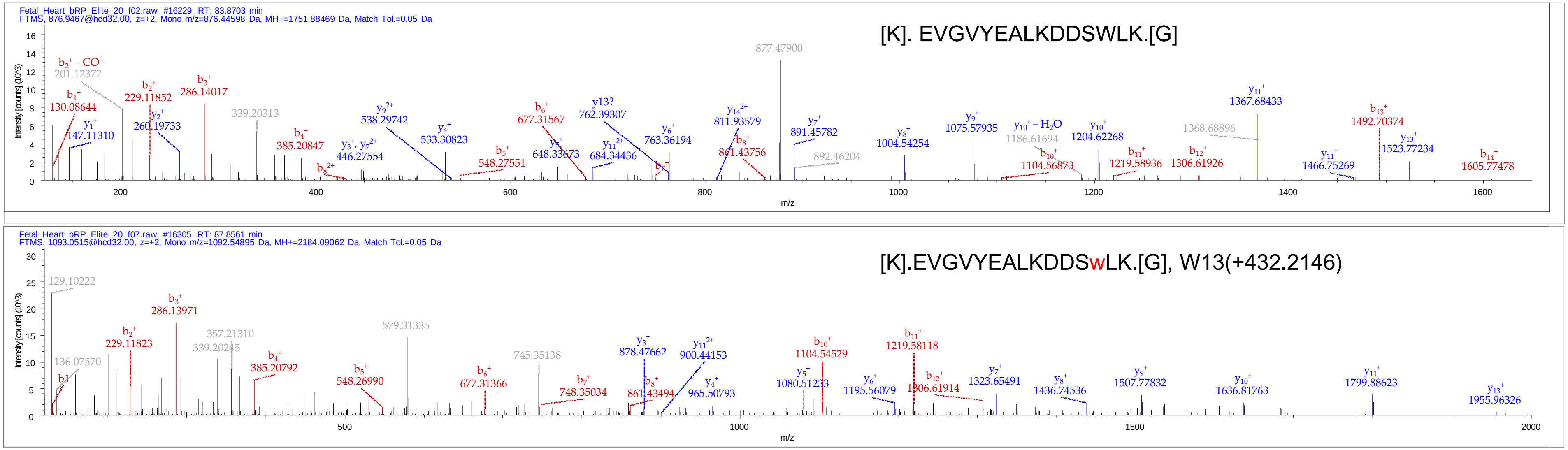
Fetal_Heart_bRP_Elite_20_f02.raw #16229 RT: 83.8703 min
FTMS, 876.9467@hcd32.00, z=+2, Mono m/z=876.44598 Da, MH+=1751.88469 Da, Match Tol.=0.05 Da
[K]. EVGVYEALKDDSWLK.[G]
Fetal_Heart_bRP_Elite_20_f07.raw #16305 RT: 87.8561 min
FTMS, 1093.0515@hcd32.00, z=+2, Mono m/z=1092.54895 Da, MH+=2184.09062 Da, Match Tol.=0.05 Da
[K].EVGVYEALKDDSwLK.[G], W13(+432.2146)

(9)

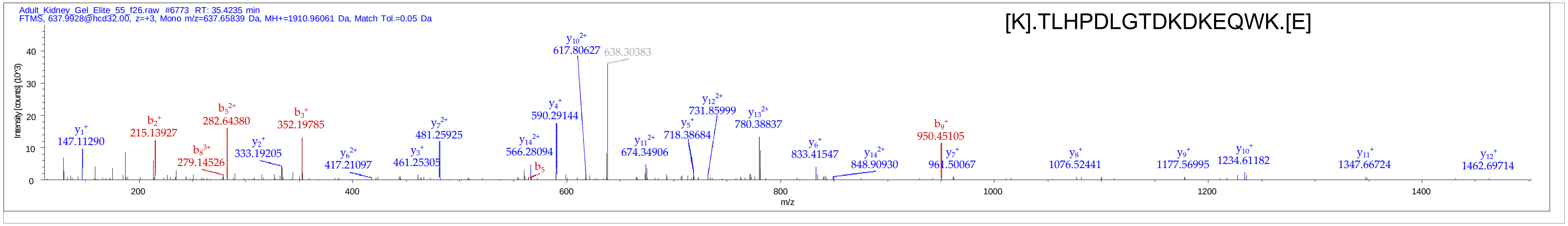
Adult_Kidney_Gel_Elite_55_f26.raw #6773 RT: 35.4235 min
FTMS, 637.9928@hcd32.00, z=+3, Mono m/z=637.65839 Da, MH+=1910.96061 Da, Match Tol.=0.05 Da
[K].TLHPDLGTDKDKEQWK.[E]
Intensity [counts] (10^3)
m/z

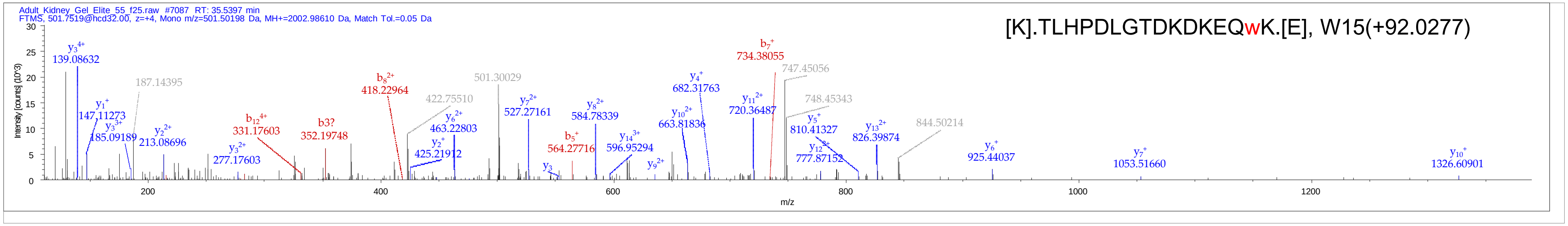
Adult_Kidney_Gel_Elite_55_f25.raw #7087 RT: 35.5397 min
FTMS, 501.7519@hcd32.00, z=+4, Mono m/z=501.50198 Da, MH+=2002.98610 Da, Match Tol.=0.05 Da
[K].TLHPDLGTDKDKEQwK.[E], W15(+92.0277)
Intensity [counts] (10^3)
m/z

(10)

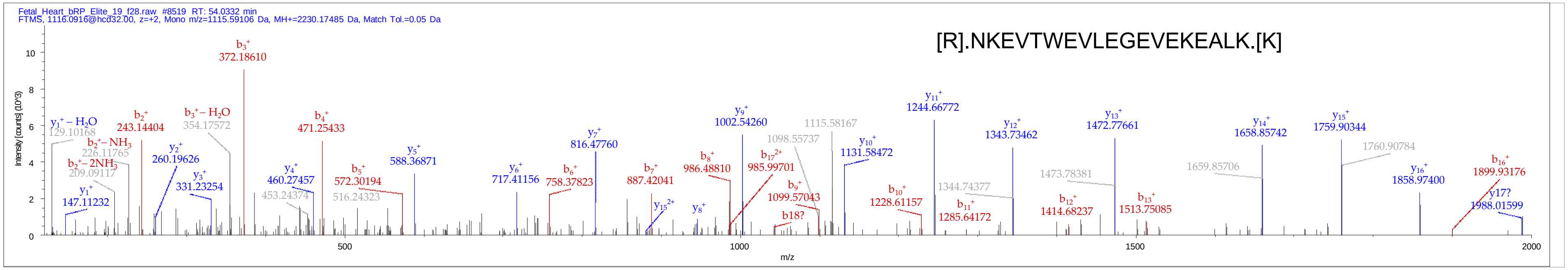
Fetal_Heart_bRP_Elite_19_f28.raw #8519 RT: 54.0332 min
FTMS, 1116.0916@hcd32.00, z=+2, Mono m/z=1115.59106 Da, MH+=2230.17485 Da, Match Tol.=0.05 Da
[R].NKEVTWEVLEGEVEKEALK.[K]
Intensity [counts] (10^3)
m/z

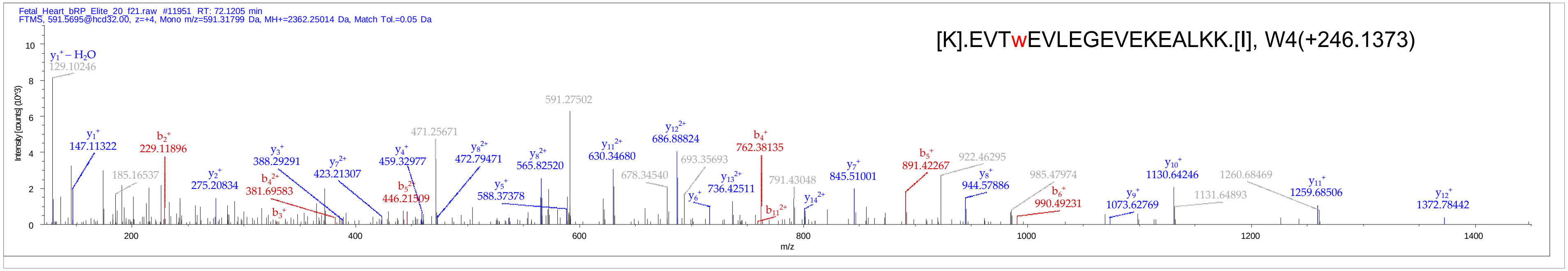
Fetal_Heart_bRP_Elite_20_f21.raw #11951 RT: 72.1205 min
FTMS, 591.5695@hcd32.00, z=+4, Mono m/z=591.31799 Da, MH+=2362.25014 Da, Match Tol.=0.05 Da
[K].EVTwEVLEGEVEKEALKK.[I], W4(+246.1373)
Intensity [counts] (10^3)
m/z

(11)

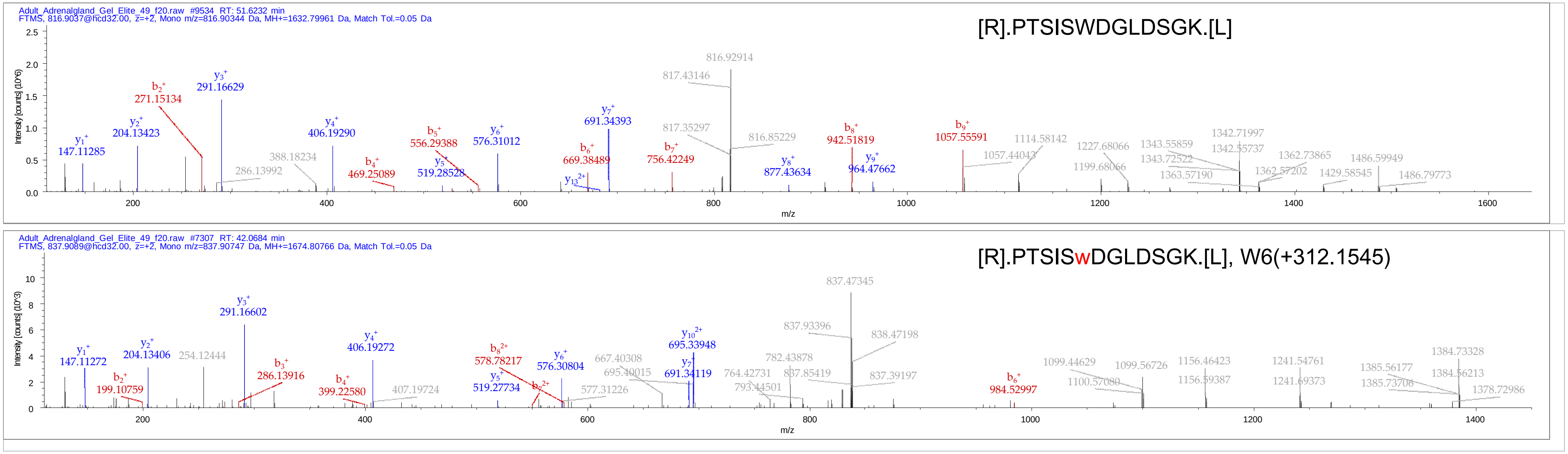

Adult_Adrenalgland_Gel_Elite_49_f20.raw #9534 RT: 51.6232 min
FTMS, 816.9037@hcd32.00, z=+2, Mono m/z=816.90344 Da, MH+=1632.79961 Da, Match Tol.=0.05 Da
[R].PTSISWDGLDSGK.[L]
Intensity [counts] (10^6)
m/z
Adult_Adrenalgland_Gel_Elite_49_f20.raw #7307 RT: 42.0684 min
FTMS, 837.9089@hcd32.00, z=+2, Mono m/z=837.90747 Da, MH+=1674.80766 Da, Match Tol.=0.05 Da
[R].PTSISwDGLDSGK.[L], W6(+312.1545)
Intensity [counts] (10^3)
m/z

(12)

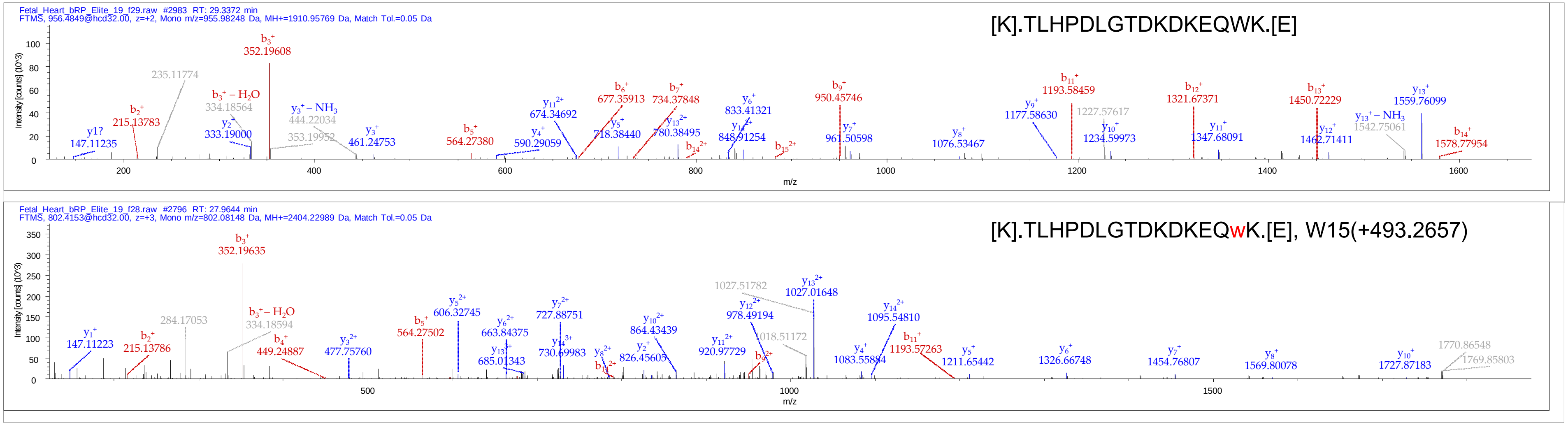

Fetal_Heart_bRP_Elite_19_f29.raw #2983 RT: 29.3372 min
FTMS, 956.4849@hcd32.00, z=+2, Mono m/z=955.98248 Da, MH+=1910.95769 Da, Match Tol.=0.05 Da
[K].TLHPDLGTDKDKEQWK.[E]
Intensity [counts] (10^3)
m/z
Fetal_Heart_bRP_Elite_19_f28.raw #2796 RT: 27.9644 min
FTMS, 802.4153@hcd32.00, z=+3, Mono m/z=802.08148 Da, MH+=2404.22989 Da, Match Tol.=0.05 Da
[K].TLHPDLGTDKDKEQwK.[E], W15(+493.2657)
Intensity [counts] (10^3)
m/z

(13)

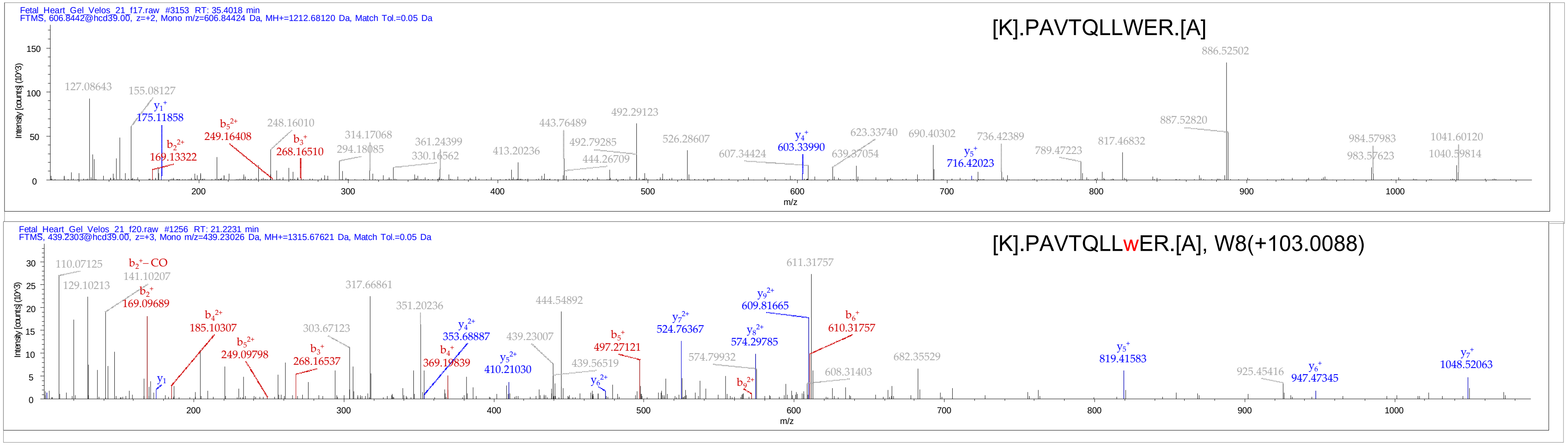

Fetal_Heart_Gel_Velos_21_f17.raw #3153 RT: 35.4018 min
FTMS, 606.8442@hcd39.00, z=+2, Mono m/z=606.84424 Da, MH+=1212.68120 Da, Match Tol.=0.05 Da
[K].PAVTQLLWER.[A]
Intensity [counts] (10^3)
m/z
Fetal_Heart_Gel_Velos_21_f20.raw #1256 RT: 21.2231 min
FTMS, 439.2303@hcd39.00, z=+3, Mono m/z=439.23026 Da, MH+=1315.67621 Da, Match Tol.=0.05 Da
[K].PAVTQLLwER.[A], W8(+103.0088)
Intensity [counts] (10^3)
m/z

(14)

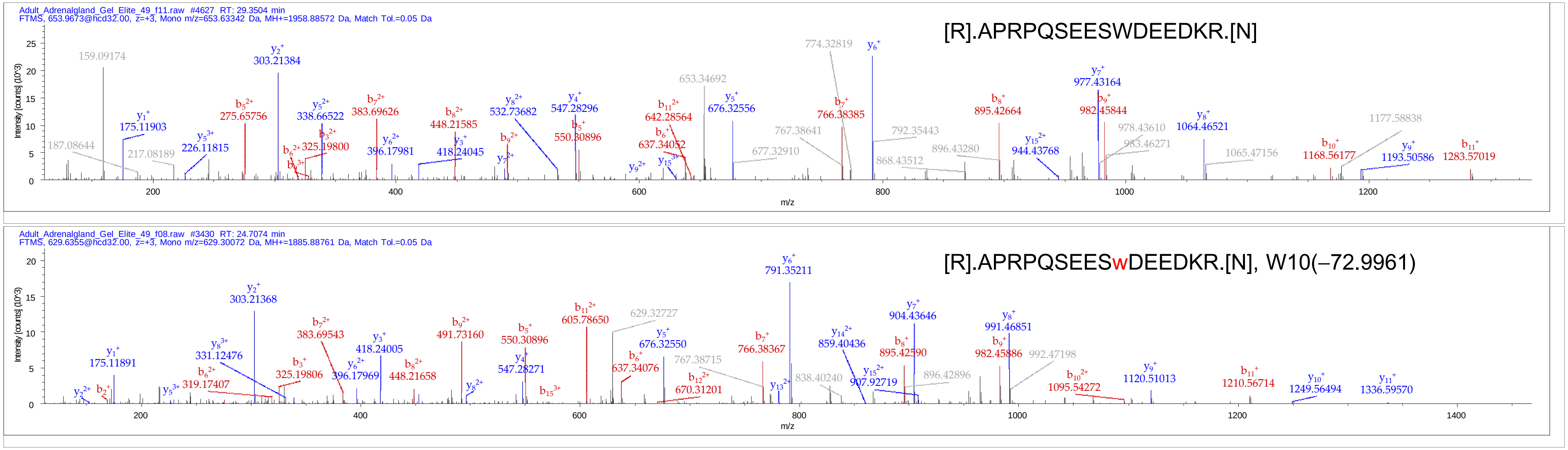

Adult_Adrenalgland_Gel_Elite_49_f11.raw #4627 RT: 29.3504 min
FTMS, 653.9673@hcd32.00, z=+3, Mono m/z=653.63342 Da, MH+=1958.88572 Da, Match Tol.=0.05 Da
[R].APRPQSEESWDEEDKR.[N]
Intensity [counts] (10^3)
m/z
Adult_Adrenalgland_Gel_Elite_49_f08.raw #3430 RT: 24.7074 min
FTMS, 629.6355@hcd32.00, z=+3, Mono m/z=629.30072 Da, MH+=1885.88761 Da, Match Tol.=0.05 Da
[R].APRPQSEESwDEEDKR.[N], W10(–72.9961)
Intensity [counts] (10^3)
m/z

(15)

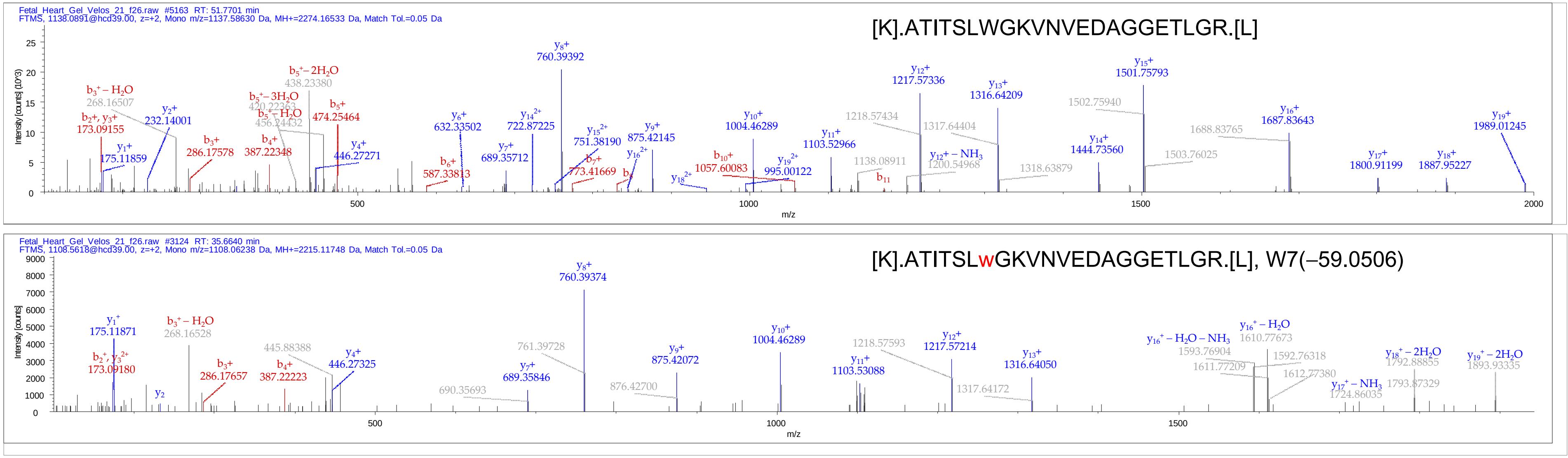
Fetal_Heart_Gel_Velos_21_f26.raw #5163 RT: 51.7701 min
FTMS, 1138.0891@hcd39.00, z=+2, Mono m/z=1137.58630 Da, MH+=2274.16533 Da, Match Tol.=0.05 Da
[K].ATITSLWGKVNVEDAGGETLGR.[L]
Intensity [counts] (10^3)
m/z
Fetal_Heart_Gel_Velos_21_f26.raw #3124 RT: 35.6640 min
FTMS, 1108.5618@hcd39.00, z=+2, Mono m/z=1108.06238 Da, MH+=2215.11748 Da, Match Tol.=0.05 Da
[K].ATITSLwGKVNVEDAGGETLGR.[L], W7(–59.0506)
Intensity [counts]
m/z

(16)

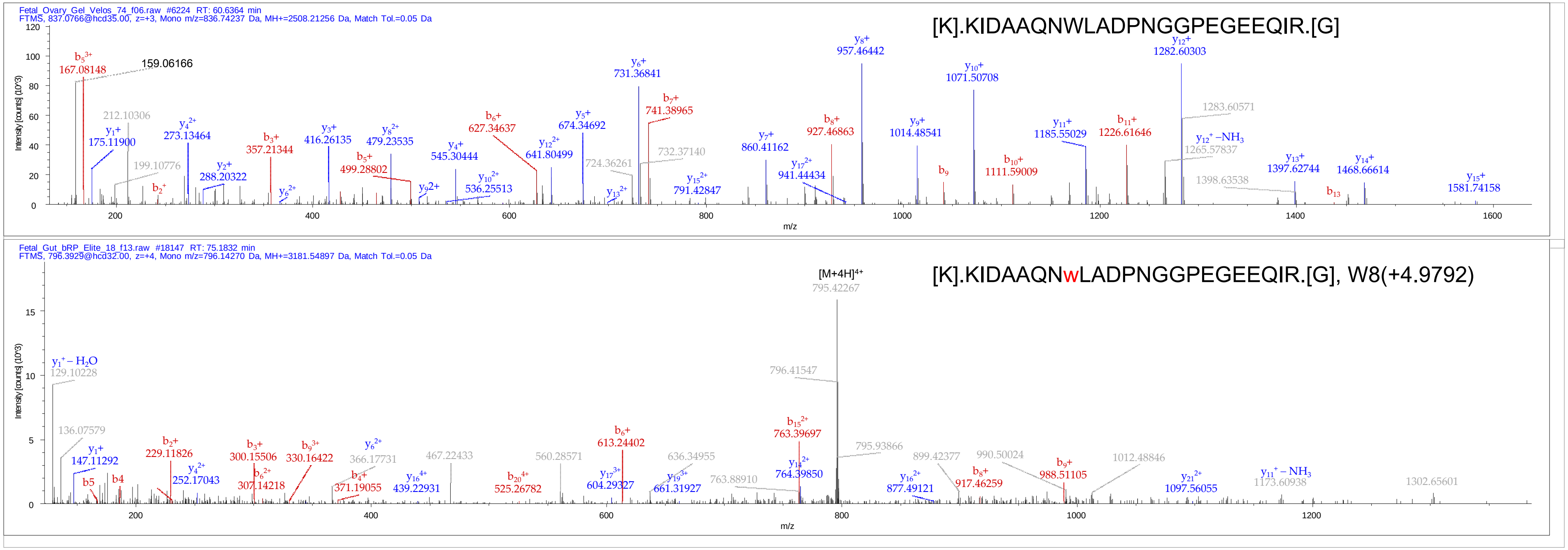
Fetal_Ovary_Gel_Velos_74_f06.raw #6224 RT: 60.6364 min
FTMS, 837.0766@hcd35.00, z=+3, Mono m/z=836.74237 Da, MH+=2508.21256 Da, Match Tol.=0.05 Da
[K].KIDAAQNWLADPNGGPEGEEQIR.[G]
Intensity [counts] (10^3)
m/z
Fetal_Gut_bRP_Elite_18_f13.raw #18147 RT: 75.1832 min
FTMS, 796.3929@hcd32.00, z=+4, Mono m/z=796.14270 Da, MH+=3181.54897 Da, Match Tol.=0.05 Da
[K].KIDAAQNwLADPNGGPEGEEQIR.[G], W8(+4.9792)
[M+4H]4+
795.42267
Intensity [counts] (10^3)
m/z

(17)

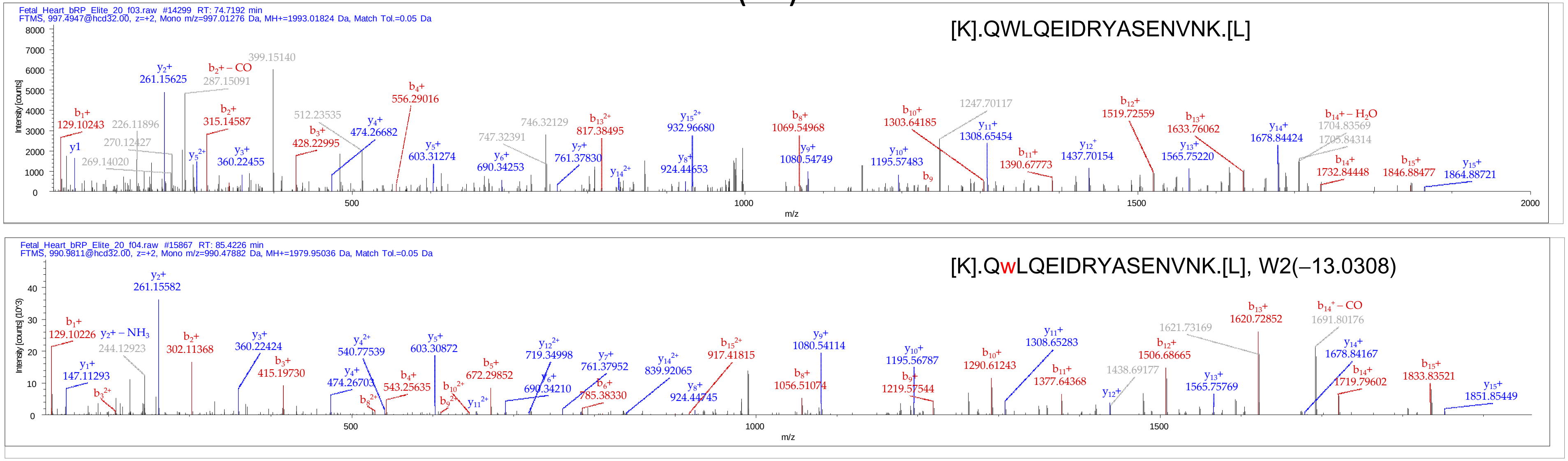

Fetal_Heart_bRP_Elite_20_f03.raw #14299 RT: 74.7192 min
FTMS, 997.4947@hcd32.00, z=+2, Mono m/z=997.01276 Da, MH+=1993.01824 Da, Match Tol.=0.05 Da
[K].QWLQEIDRYASENVNK.[L]
Intensity [counts]
m/z
b1+ 129.10243
y1
226.11896
270.12427
269.14020
y2+ 261.15625
b2+ – CO 287.15091
399.15140
y5 2+
b2+ 315.14587
y3+ 360.22455
b3+ 428.22995
512.23535
y4+ 474.26682
b4+ 556.29016
y5+ 603.31274
y6+ 690.34253
747.32391
746.32129
y7+ 761.37830
b13 2+ 817.38495
y14 2+
y15 2+ 932.96680
y8+ 924.44653
b8+ 1069.54968
y9+ 1080.54749
y10+ 1195.57483
b9
b10+ 1303.64185
1247.70117
y11+ 1308.65454
b11+ 1390.67773
y12+ 1437.70154
b12+ 1519.72559
y13+ 1565.75220
b13+ 1633.76062
y14+ 1678.84424
b14+ – H2O
1704.83569
1705.84314
b14+ 1732.84448
b15+ 1846.88477
y15+ 1864.88721
Fetal_Heart_bRP_Elite_20_f04.raw #15867 RT: 85.4226 min
FTMS, 990.9811@hcd32.00, z=+2, Mono m/z=990.47882 Da, MH+=1979.95036 Da, Match Tol.=0.05 Da
[K].QwLQEIDRYASENVNK.[L], W2(–13.0308)
Intensity [counts] (10^3)
m/z
b1+ 129.10226
y2+ – NH3 244.12923
y1+ 147.11293
b3 2+
y2+ 261.15582
b2+ 302.11368
y3+ 360.22424
b3+ 415.19730
y4 2+ 540.77539
y4+ 474.26703
b8 2+
b4+ 543.25635
y5+ 603.30872
b9 2+
b10 2+
y11 2+
b5+ 672.29852
y12 2+ 719.34998
y6+ 690.34210
y7+ 761.37952
b6+ 785.38330
y14 2+ 839.92065
y8+ 924.44745
b15 2+ 917.41815
b8+ 1056.51074
y9+ 1080.54114
y10+ 1195.56787
b9+ 1219.57544
b10+ 1290.61243
y11+ 1308.65283
b11+ 1377.64368
1438.69177
y12+
1621.73169
b12+ 1506.68665
y13+ 1565.75769
b13+ 1620.72852
b14+ – CO 1691.80176
y14+ 1678.84167
b14+ 1719.79602
b15+ 1833.83521
y15+ 1851.85449

(18)

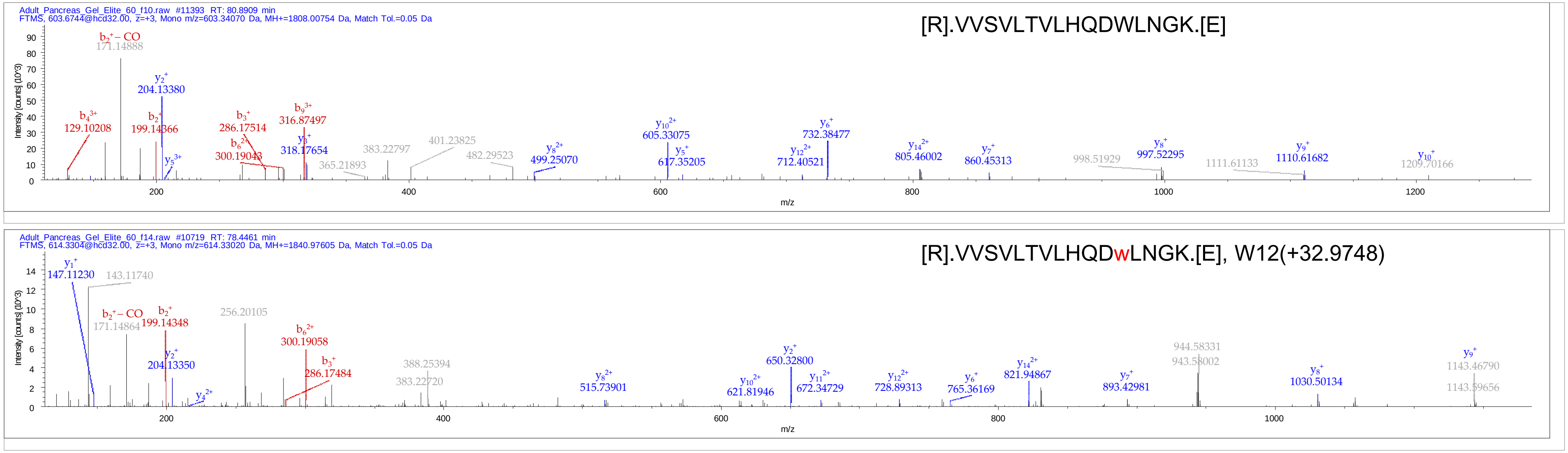

Adult_Pancreas_Gel_Elite_60_f10.raw #11393 RT: 80.8909 min
FTMS, 603.6744@hcd32.00, z=+3, Mono m/z=603.34070 Da, MH+=1808.00754 Da, Match Tol.=0.05 Da
[R].VVSVLTVLHQDWLNGK.[E]
Intensity [counts] (10^3)
m/z
b2+ – CO 171.14888
b4 3+ 129.10208
y2+ 204.13380
b2+ 199.14366
y5 3+
b3+ 286.17514
b6 2+ 300.19043
b9 3+ 316.87497
y3+ 318.17654
365.21893
383.22797
401.23825
482.29523
y8 2+ 499.25070
y10 2+ 605.33075
y5+ 617.35205
y12 2+ 712.40521
y6+ 732.38477
y14 2+ 805.46002
y7+ 860.45313
998.51929
y8+ 997.52295
1111.61133
y9+ 1110.61682
y10+ 1209.70166
Adult_Pancreas_Gel_Elite_60_f14.raw #10719 RT: 78.4461 min
FTMS, 614.3304@hcd32.00, z=+3, Mono m/z=614.33020 Da, MH+=1840.97605 Da, Match Tol.=0.05 Da
[R].VVSVLTVLHQDwLNGK.[E], W12(+32.9748)
Intensity [counts] (10^3)
m/z
y1+ 147.11230
143.11740
b2+ – CO 171.14864
b2+ 199.14348
y2+ 204.13350
y4 2+
256.20105
b6 2+ 300.19058
b3+ 286.17484
388.25394
383.22720
y8 2+ 515.73901
y10 2+ 621.81946
y2+ 650.32800
y11 2+ 672.34729
y12 2+ 728.89313
y6+ 765.36169
y14 2+ 821.94867
y7+ 893.42981
944.58331
943.58002
y8+ 1030.50134
y9+ 1143.46790
1143.59656

(19)

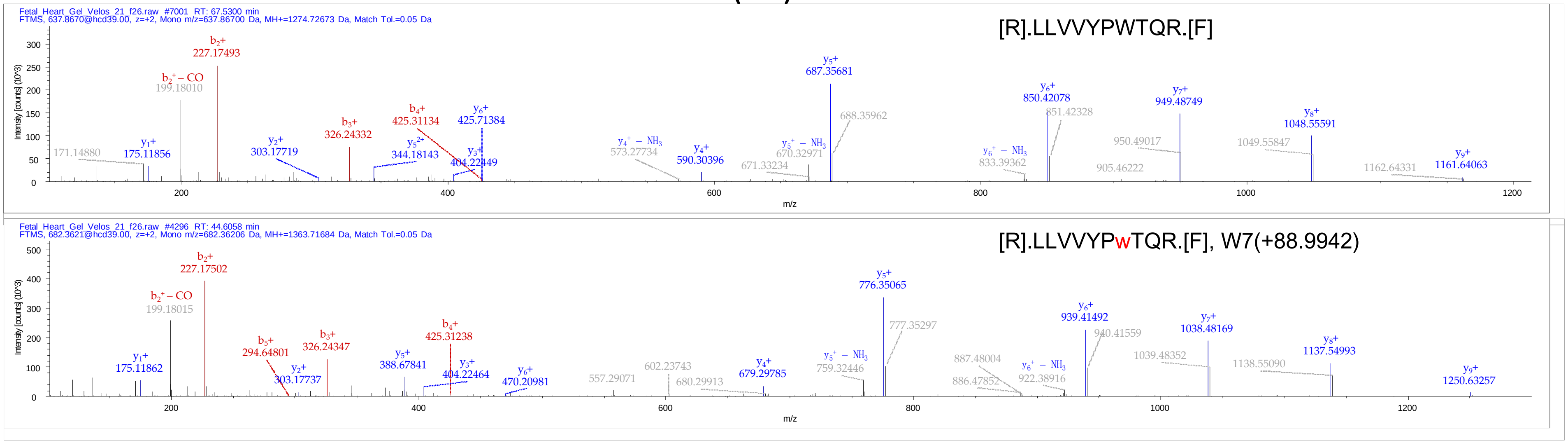
Fetal_Heart_Gel_Velos_21_f26.raw #7001 RT: 67.5300 min
FTMS, 637.8670@hcd39.00, z=+2, Mono m/z=637.86700 Da, MH+=1274.72673 Da, Match Tol.=0.05 Da
[R].LLVVYPWTQR.[F]
Intensity [counts] (10^3)
m/z
b2+ 227.17493
b2+ – CO 199.18010
y1+ 175.11856
171.14880
y2+ 303.17719
b3+ 326.24332
y5 2+ 344.18143
b4+ 425.31134
y6+ 425.71384
y3+ 404.22449
y4+ – NH3 573.27734
y4+ 590.30396
y5+ – NH3 670.32971
671.33234
y5+ 687.35681
688.35962
y6+ – NH3 833.39362
y6+ 850.42078
851.42328
950.49017
905.46222
y7+ 949.48749
1049.55847
y8+ 1048.55591
1162.64331
y9+ 1161.64063
Fetal_Heart_Gel_Velos_21_f26.raw #4296 RT: 44.6058 min
FTMS, 682.3621@hcd39.00, z=+2, Mono m/z=682.36206 Da, MH+=1363.71684 Da, Match Tol.=0.05 Da
[R].LLVVYPwTQR.[F], W7(+88.9942)
b2+ 227.17502
b2+ – CO 199.18015
y1+ 175.11862
b5+ 294.64801
y2+ 303.17737
b3+ 326.24347
y5+ 388.67841
b4+ 425.31238
y3+ 404.22464
y6+ 470.20981
557.29071
602.23743
680.29913
y4+ 679.29785
y5+ – NH3 759.32446
y5+ 776.35065
777.35297
887.48004
886.47852
y6+ – NH3 922.38916
y6+ 939.41492
940.41559
1039.48352
y7+ 1038.48169
1138.55090
y8+ 1137.54993
y9+ 1250.63257

(20)

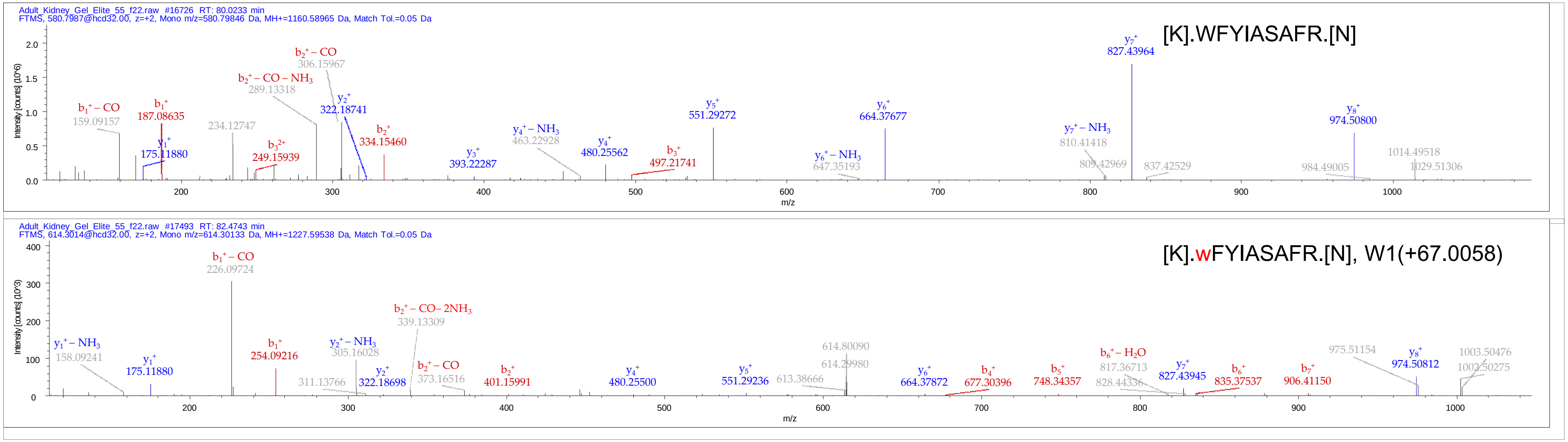
Adult_Kidney_Gel_Elite_55_f22.raw #16726 RT: 80.0233 min
FTMS, 580.7987@hcd32.00, z=+2, Mono m/z=580.79846 Da, MH+=1160.58965 Da, Match Tol.=0.05 Da
[K].WFYIASAFR.[N]
Intensity [counts] (10^6)
m/z
b1+ – CO 159.09157
b1+ 187.08635
y1+ 175.11880
234.12747
b2+ – CO – NH3 289.13318
b2+ – CO 306.15967
y2+ 322.18741
b3 2+ 249.15939
b2+ 334.15460
y3+ 393.22287
y4+ – NH3 463.22928
y4+ 480.25562
b3+ 497.21741
y5+ 551.29272
y6+ – NH3 647.35193
y6+ 664.37677
y7+ – NH3 810.41418
809.42969
y7+ 827.43964
837.42529
984.49005
y8+ 974.50800
1014.49518
1029.51306
Adult_Kidney_Gel_Elite_55_f22.raw #17493 RT: 82.4743 min
FTMS, 614.3014@hcd32.00, z=+2, Mono m/z=614.30133 Da, MH+=1227.59538 Da, Match Tol.=0.05 Da
[K].wFYIASAFR.[N], W1(+67.0058)
y1+ – NH3 158.09241
y1+ 175.11880
b1+ – CO 226.09724
b1+ 254.09216
311.13766
y2+ – NH3 305.16028
y2+ 322.18698
b2+ – CO– 2NH3 339.13309
b2+ – CO 373.16516
b2+ 401.15991
y4+ 480.25500
y5+ 551.29236
613.38666
614.80090
614.29980
y6+ 664.37872
b4+ 677.30396
b5+ 748.34357
b6+ – H2O 817.36713
828.44336
y7+ 827.43945
b6+ 835.37537
b7+ 906.41150
975.51154
y8+ 974.50812
1003.50476
1002.50275

(21)

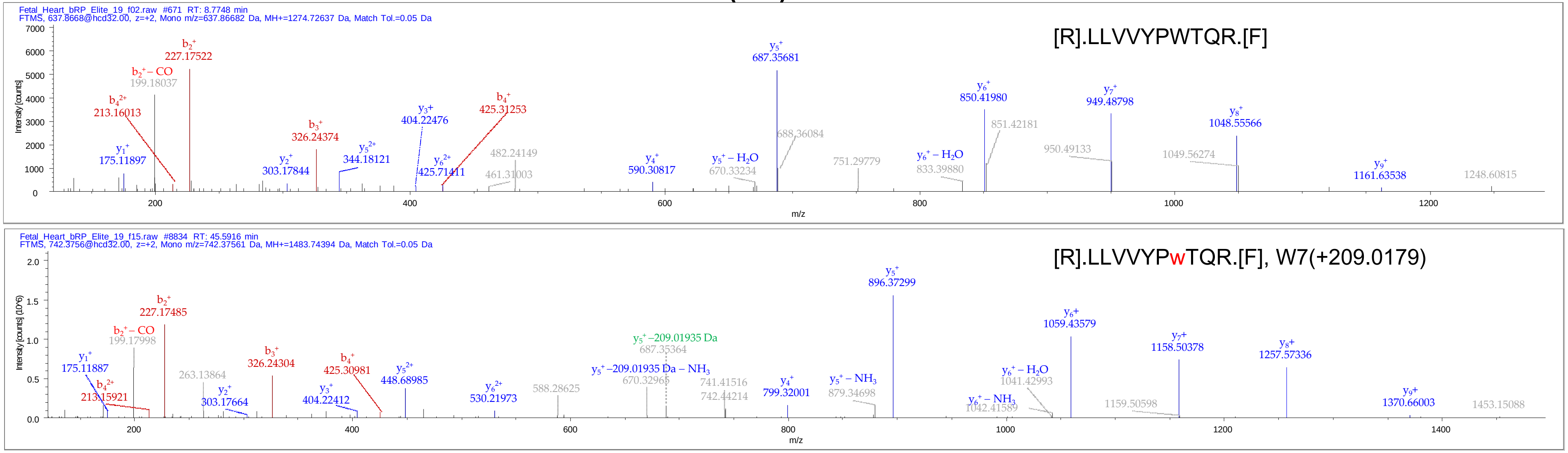
Fetal_Heart_bRP_Elite_19_f02.raw #671 RT: 8.7748 min
FTMS, 637.8668@hcd32.00, z=+2, Mono m/z=637.86682 Da, MH+=1274.72637 Da, Match Tol.=0.05 Da
[R].LLVVYPWTQR.[F]
Intensity [counts]
m/z
Fetal_Heart_bRP_Elite_19_f15.raw #8834 RT: 45.5916 min
FTMS, 742.3756@hcd32.00, z=+2, Mono m/z=742.37561 Da, MH+=1483.74394 Da, Match Tol.=0.05 Da
[R].LLVVYPwTQR.[F], W7(+209.0179)
Intensity [counts] (10^6)
m/z

(22)

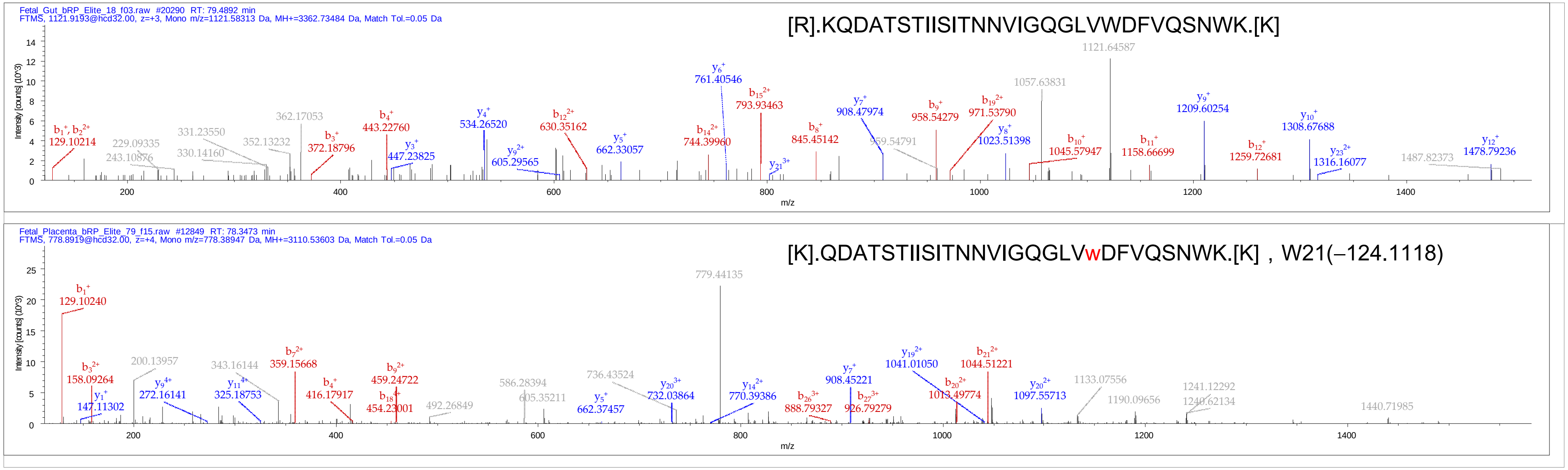
Fetal_Gut_bRP_Elite_18_f03.raw #20290 RT: 79.4892 min
FTMS, 1121.9193@hcd32.00, z=+3, Mono m/z=1121.58313 Da, MH+=3362.73484 Da, Match Tol.=0.05 Da
[R].KQDATSTIISITNNVIGQGLVWDFVQSNWK.[K]
Intensity [counts] (10^3)
m/z
Fetal_Placenta_bRP_Elite_79_f15.raw #12849 RT: 78.3473 min
FTMS, 778.8919@hcd32.00, z=+4, Mono m/z=778.38947 Da, MH+=3110.53603 Da, Match Tol.=0.05 Da
[K].QDATSTIISITNNVIGQGLVwDFVQSNWK.[K] , W21(–124.1118)
Intensity [counts] (10^3)
m/z

(23)

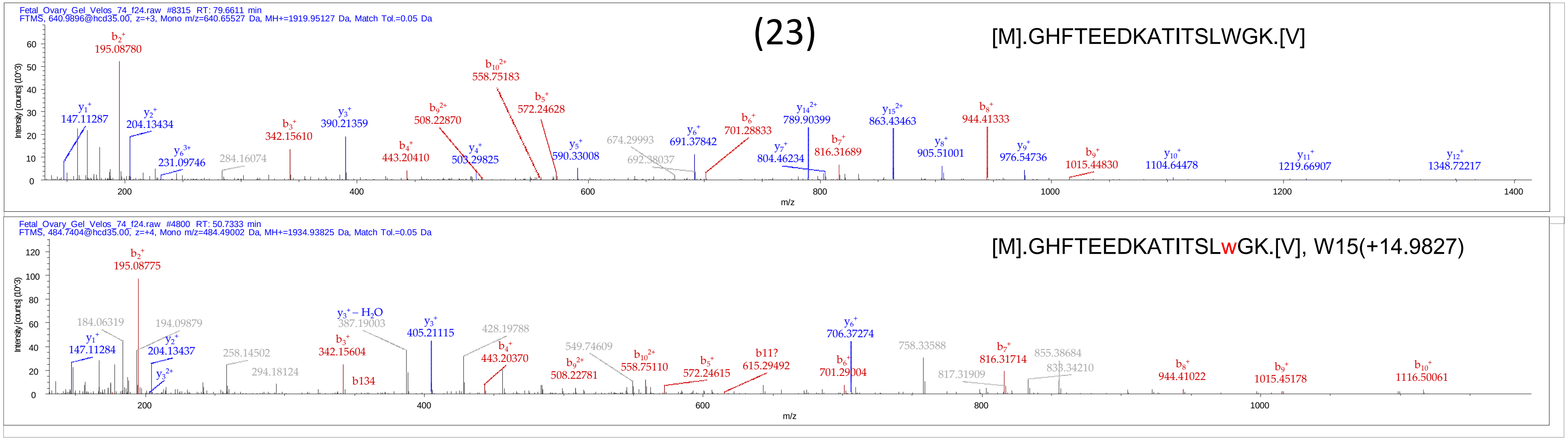
Fetal_Ovary_Gel_Velos_74_f24.raw #8315 RT: 79.6611 min
FTMS, 640.9896@hcd35.00, z=+3, Mono m/z=640.65527 Da, MH+=1919.95127 Da, Match Tol.=0.05 Da
[M].GHFTEEDKATITSLWGK.[V]
Intensity [counts] (10^3)
m/z
Fetal_Ovary_Gel_Velos_74_f24.raw #4800 RT: 50.7333 min
FTMS, 484.7404@hcd35.00, z=+4, Mono m/z=484.49002 Da, MH+=1934.93825 Da, Match Tol.=0.05 Da
[M].GHFTEEDKATITSLwGK.[V], W15(+14.9827)
Intensity [counts] (10^3)
m/z

(24)

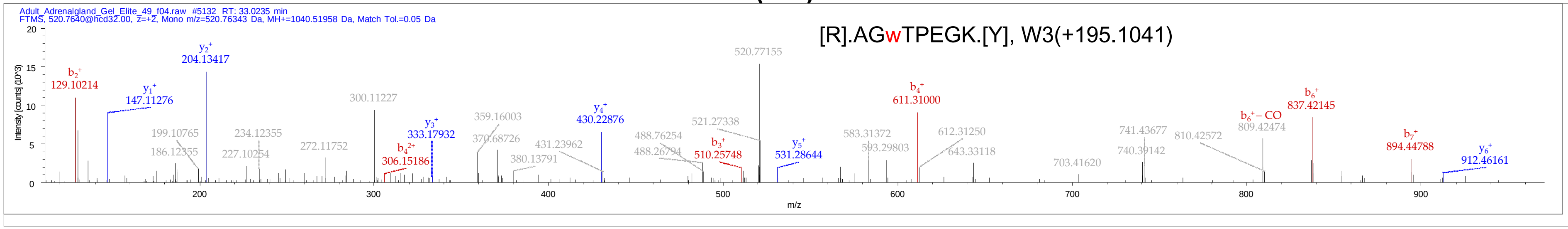
Adult_Adrenalgland_Gel_Elite_49_f04.raw #5132 RT: 33.0235 min
FTMS, 520.7640@hcd32.00, z=+2, Mono m/z=520.76343 Da, MH+=1040.51958 Da, Match Tol.=0.05 Da
[R].AGwTPEGK.[Y], W3(+195.1041)
Intensity [counts] (10^3)
m/z

(25)

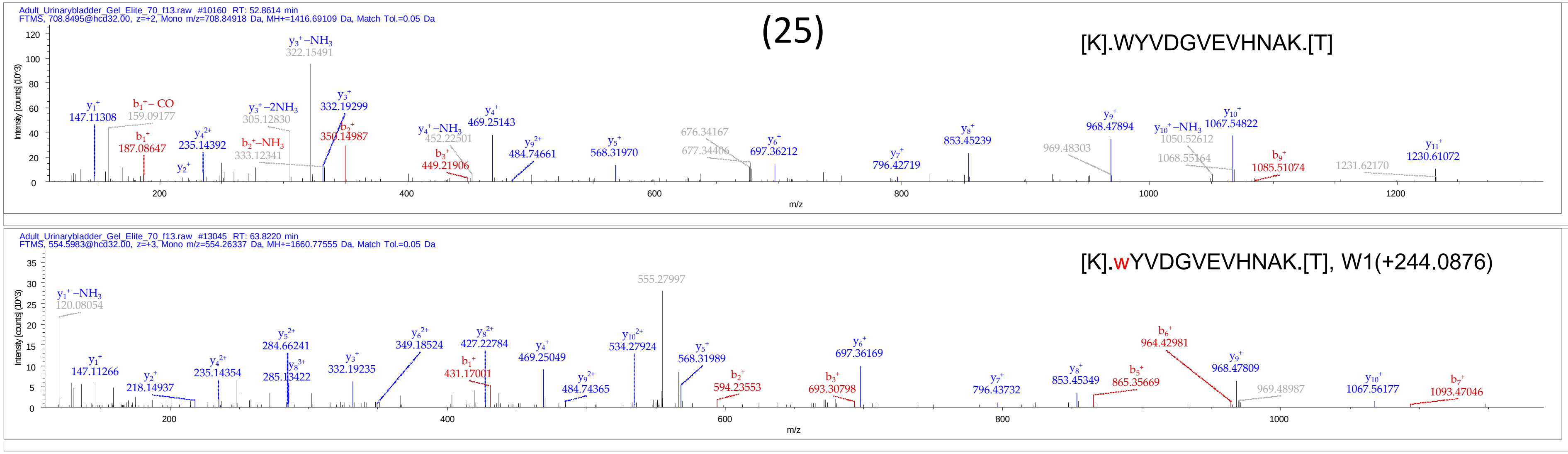
Adult_Urinarybladder_Gel_Elite_70_f13.raw #10160 RT: 52.8614 min
FTMS, 708.8495@hcd32.00, z=+2, Mono m/z=708.84918 Da, MH+=1416.69109 Da, Match Tol.=0.05 Da
[K].WYVDGVEVHNAK.[T]
Intensity [counts] (10^3)
m/z
Adult_Urinarybladder_Gel_Elite_70_f13.raw #13045 RT: 63.8220 min
FTMS, 554.5983@hcd32.00, z=+3, Mono m/z=554.26337 Da, MH+=1660.77555 Da, Match Tol.=0.05 Da
[K].wYVDGVEVHNAK.[T], W1(+244.0876)
Intensity [counts] (10^3)
m/z

Supplementary Figure 2

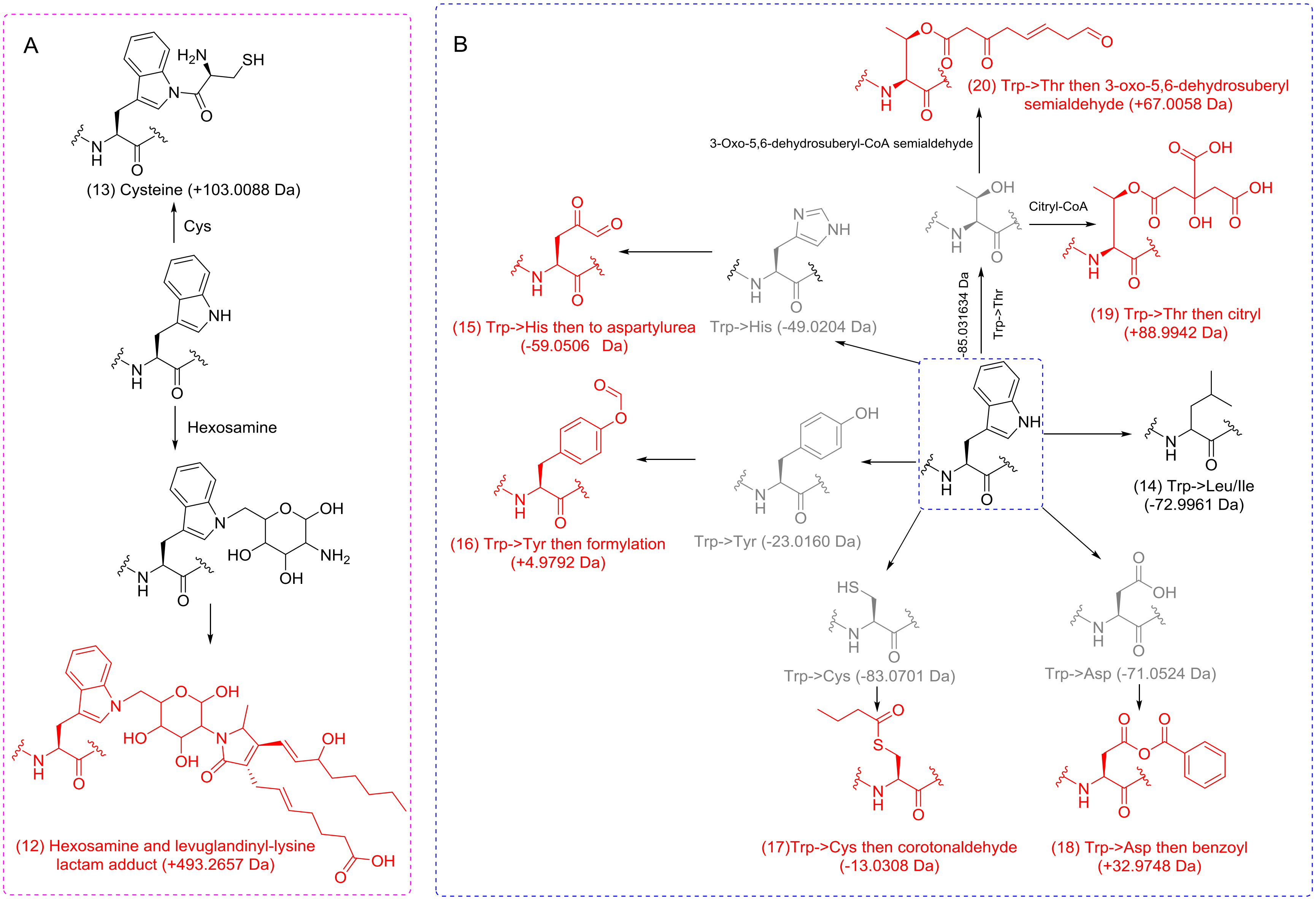

Supplemental Figure 3

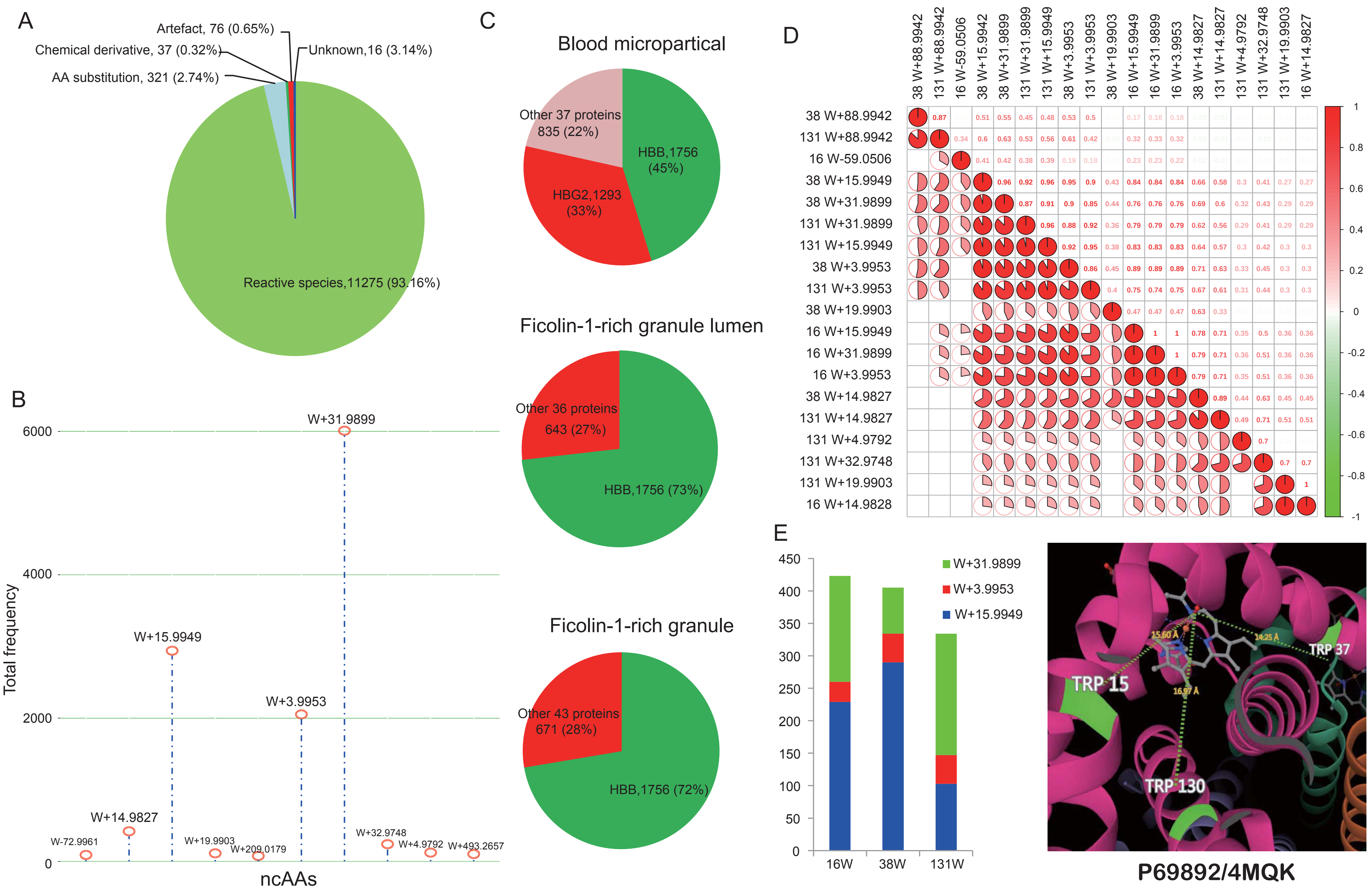

Supplementary Figure 4

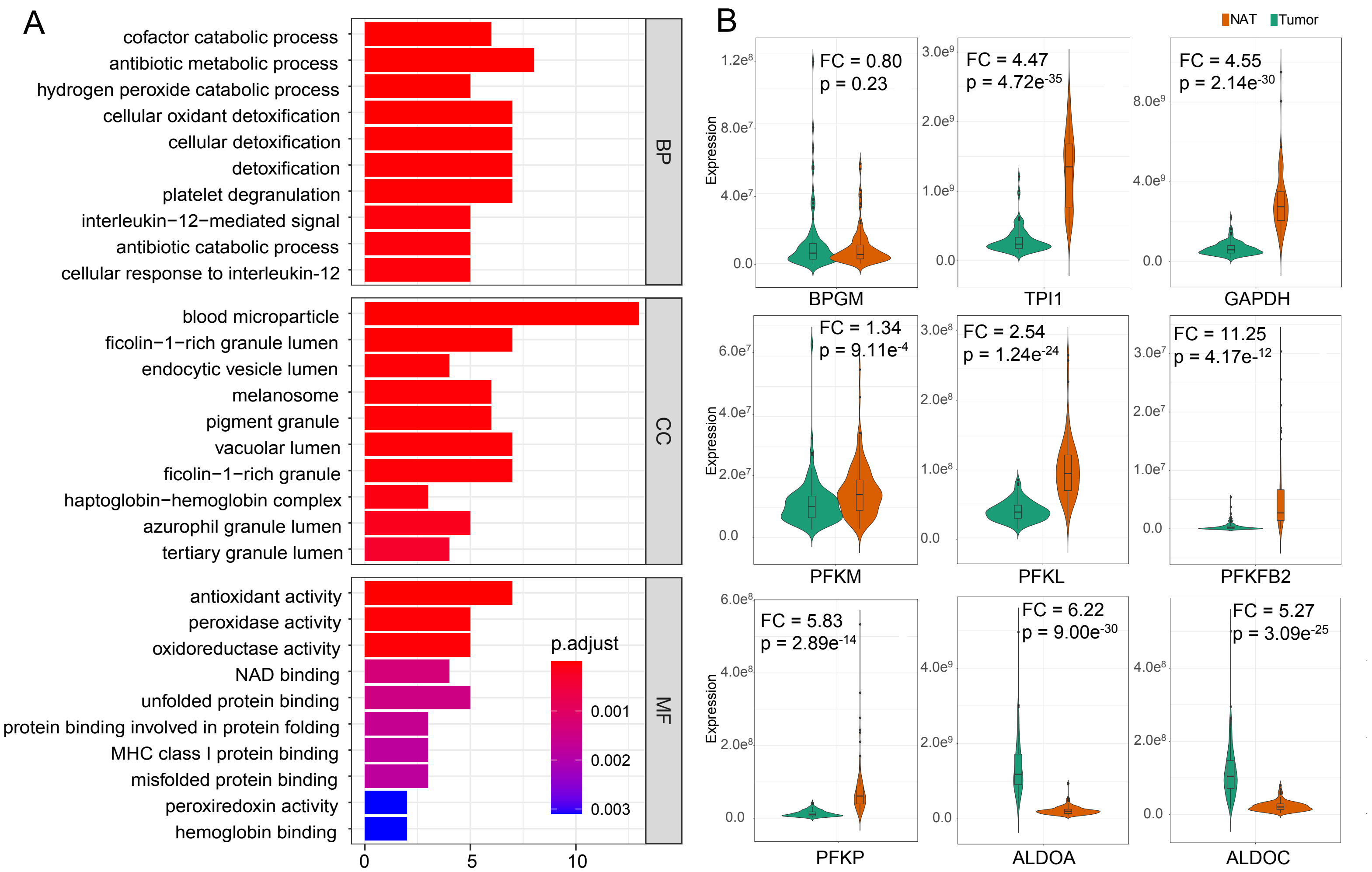

Supplementary Figure 5

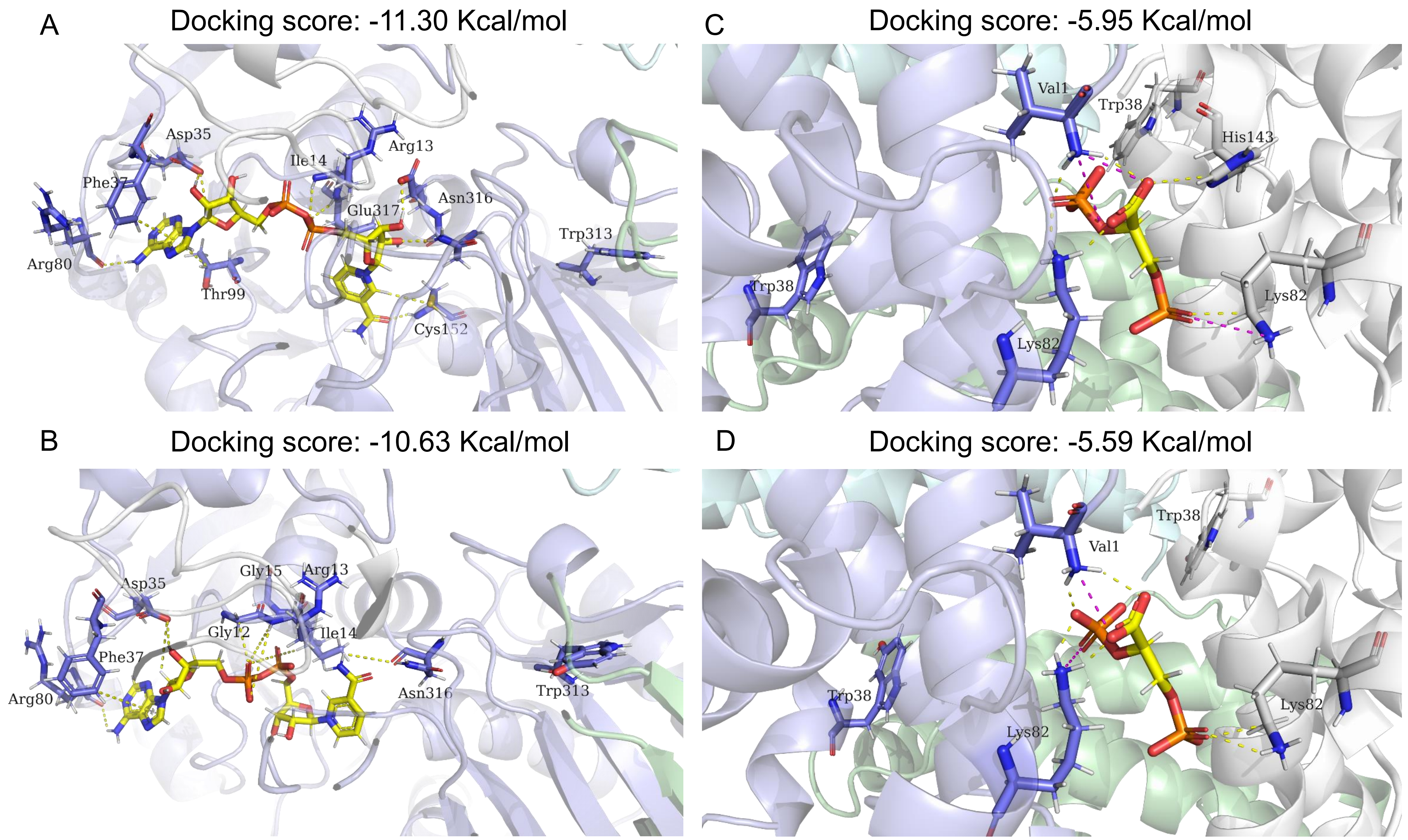

Supplement: Supplementary Materials — Supplementary Figure 1: peptide spectrum matches (PSMs) of the identified 25 Trp variants. Supplementary Figure 2: proposed pathways of chemical reaction with in vivo metabolites (A) and tryptophan substitutions (B). The red-colored structures indicate the potential new modifications at tryptophan residue; the structures in grey color show the intermediates of the tryptophan modification pathway; the structures in black color show the preidentified tryptophan modifications. Supplementary Figure 3: proteins with tryptophan variants were largely clustered in blood microparticle, related to Figure 4. (a) Relative frequencies of each tryptophan modification group in the dataset. (b) Relative frequencies of delta mass clusters in the dataset. (c) Relative frequencies of each protein in the cellular components of blood microparticle, ficolin-1-rich granule lumen, and ficolin-1-rich granule. D. Heatmap depicting the correlation of tryptophan modifications in P69892 (HBG2). E. Color bar represents the relative frequency of differentially expressed oxidation modification at the 16 W, 38 W, and 131 W sites of P69892 (HBG2); the graph shows the overall structure of heme-core in P69892 (PDB: 4MQK). The linear distances of the 16 W, 38 W, and 131 W sites from the heme group are shown. Supplementary Figure 4: tryptophan variants associated with antioxidants prone to oxidative stress in NSCLC. A. Gene Ontology enrichment analysis of the modified-tryptophan-containing proteins in NSCLC, related to Figure 5(a); B. Relative expression levels of glycolytic enzymes in tumor samples and adjacent normal tissues in an independent cohort of 103 LUAD proteomic dataset (Xu et al., 2020, Cell 182, 245–261), related to Figure 6. Supplementary Figure 5: molecular docking of GAPDH (PubChem CID: 6 M61) with NAD+ and HBB (PubChem CID: 1CBL) with 2,3-diphosphoglycerate before and after oxidation, respectively. A. The 3D binding mode of NAD+ with GAPDH-wt; B. The 3D binding mode of NAD+ with GAPDH-w [file 2590198.f1.zip › Supplementary Materials_20220306 (1).pdf]
